# Supplementary material for: Electrochemical C−H deuteration of pyridine derivatives with D2O
Source: Nat Commun. 2024 May 7;15:3832. doi: 10.1038/s41467-024-48262-9 (PMC11076510; doi:10.1038/s41467-024-48262-9)
Supplement: Supplementary file 1 — Supplementary Information [file 41467_2024_48262_MOESM1_ESM.pdf]

# Supplementary Information

## Electrochemical C−H deuteration of Pyridine Derivatives with $D_2O$

**Zhiwei Zhao, Ranran Zhang, Yaowen Liu, Zile Zhu, Qiuyan Wang,* Youai Qiu***

**State Key Laboratory and Institute of Elemento-Organic Chemistry, Frontiers Science Center for New Organic Matter, College of Chemistry, Nankai University, 94 Weijin Road, Tianjin, 300071, China**

***qiuyouai@nankai.edu.cn**

# Table of Contents

**1. Supplementary notes .......... 3**
**1.1 Methods.......... 3**
**1.2 Materials and reagents .......... 3**
**1.3 Instrumentation.......... 3**
**1.4 General remarks .......... 4**
**2. Supplementary methods .......... 5**
**2.1 Supplementart Table 1: Optimization of the reaction conditions .......... 5**
**2.2 Supplementart Table 2: The effect of the electrolyte .......... 6**
**2.3 Supplementart Table 3: Optimization of the reaction time .......... 7**
**2.4 Supplementart Table 4: The effect of the electrode.......... 8**
**2.5 General procedure of electrochemical C–H deuteration of pyridine derivatives .......... 9**
**2.6 General procedure of observation of intermediate S1-a .......... 11**
**2.7 General procedure of the preparation of *N*-alkyl-2-phenylpyridinium iodide salts... 12**
**3. Supplementary discussion .......... 13**
**3.1 Mechanism research .......... 13**
**3.2 Cyclic voltammety.......... 31**
**3.3 Characterization of products .......... 33**
**3.4 General information of DFT computational studies.......... 68**
**3.5 NMR spectra.......... 74**
**4. Supplemental references .......... 156**

# 1. Supplementary notes

## 1.1 Methods

All reactions were performed in standard, oven-dried glassware under air atmosphere. Catalytic reactions were carried out in undivided electrochemical cells (15 mL) using pre-dried glassware, if not noted otherwise. Flash chromatography was performed using Silica gel (200-300 mesh) purchased from Qingdao Haiyang Chemical Co, China. Thin layer chromatography was used for product detection using silicone plates, After ultraviolet irradiation ($\lambda ex$ = 254nm), the result was visible.

## 1.2 Materials and reagents:

Most chemical reagents such as (2-phenylpyridine; CAS = 1008-89-5), (2,6-diphenylpyridine; CAS = 3558-69-8) and pyridine, quinoline derivatives were purchased from *J&K* Scientific, TCI, Bidepharm, Energy Chemical, Alfa Aesar, and used as received. The starting organic arenes were obtained from commercial sources or synthesized according to literature methods.[1-2]. Anhydrous solvents (diethyl ether, toluene, tetrahydrofuran (THF), dichloromethane (DCM), dimethyl sulfoxide (DMSO) and dimethylformamide (DMF) *N*,*N*-dimethylacetamide (DMA) were purchased from Energy Chemical and *J&K* Scientific, and dried using anhydrous $MgSO_4$. Deuterated solvents were purchased from *J&K* Scientific.

## 1.3 Instrumentation:

Nuclear magnetic resonance (NMR) spectra were recorded on AVANCE AV 400 spectrometer. Proton NMR spectra are reported in parts per million (ppm) downfield from tetramethylsilane and are referenced using the NMR solvent ($CDCl_3$: 7.26 ppm, DMSO-$d_6$: 2.50 ppm). Proton-decoupled $^{13}C$ NMR spectra are reported in ppm downfield from tetramethylsilane, and are referenced using the NMR solvent ($CDCl_3$: 77.00 ppm). $^{19}F$ NMR spectra are reported in ppm downfield from chlorotrifluoromethane. High-resolution Mass Spectrometry (HRMS) data were acquired by Nankai university of Science Molecule Mass Spectrometry facility. Graphite felt electrodes (10 mm × 15 mm × 5 mm), Jinglong company, Beijing, China) were connected using stainless steel adapters. Electrocatalysis was conducted using an HSPY-36-03 potentiostat in constant current mode. Cyclic Voltammetry studies were performed using a Shanghai Chenhua CHI760E workstation. High-resolution mass spectrometry (HRMS) was done on a

FTICR-mass spectrometer. Melting points were recorded on Shanghai ShenGuang WRS–2 apparatus. Visualization was achieved under a UV lamp (254 nm and 365 nm).

### 1.4 General remarks

NMR spectra were recorded on Bruker AVANCE AV 400 in the solvent indicated; using $CDCl_3$ or DMSO-$d_6$ as the solvent with tetramethylsilane (TMS) as the internal standard at room temperature, chemical shifts are given in ppm relative to the residual solvent peak, coupling constants (J) are reported in Hertz (Hz). Multiplicities are recorded as: s = singlet, d = doublet, t = triplet, dd = doublet of doublets, m = multiplet. Coupling constants are measured in Hertz (Hz). Ar = aryl, Bn = benzyl, c = concentration, Bz = Benzoyl, Cbz = benzyloxycarbonyl, DCM = dichloromethane, DMF = dimethylformamide, DMA = *N*,*N*-dimethylacetamide. PE = petroleum ether, EA = ethyl acetate, DCM = dichloromethane. equiv. = equivalents, $Et_2O$ = diethyl ether, EtOAc = ethyl acetate, EtOH = ethanol, g = grams, h = hours, HPLC = high-performance liquid chromatography, HRMS = high-resolution mass spectrometry, mg = milligrams, Hz = Hertz, $^{i}$Am = isoamyl, $^{i}$Bu = isobutyl, $^{i}$Pr = isopropyl, $^{i}$PrOH = isopropyl alcohol, MeOH = methanol, Me = methyl, min = minutes, m/z = mass to charge ratio, $^{n}$Bu = *n*-butyl, NMR = nuclear magnetic resonance, Ph = phenyl, ppm = parts per million, *p*-TsOH = para-toluenesulfonic acid, TFA = trifluoroacetic acid, THF = tetrahydrofuran, TMS = trimethylsilyl, Ts = para-tolylsulfonyl.

## 2. Supplementary methods

**2.1 Supplementary Table 1:** Optimization of the reaction conditions[a]

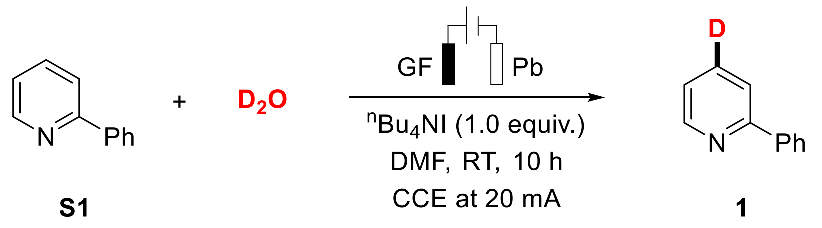


| Entry | Variation from standard conditions | Yield of **1** or recovered **S1** (%)[b] | **1** (D%)[c] |
|---|---|---|---|
| **1** | **$^{n}Bu_4NI$** | **99** | **>99** |
| 2 | NaOAc (1.0 equiv.) | 99 | 16 |
| 3 | DMA as solvent | 99 | 82 |
| 4 | MeCN | 99 | <5 |
| 5 | CF(+) \| Pb(−) | 90 | 80 |
| 6 | GF(+) \| GF(−) | 99 | 35 |
| 7 | GF(+) \| Pt(−) | 99 | 53 |
| 8 | 10 mA | 99 | 60 |
| 9 | 15 mA | 99 | 81 |
| 10 | T = 50 °C | 99 | >99 |
| 11 | Ar | 99 | 99 |
| 12 | w/o electricity | 99 | 99 |
| 13 | w/o electrolyte | 99 | 0 |

[a]Reaction conditions: undivided cell, graphite felt (GF) as anode and Pb as cathode, constant current (20 mA), **S1** (0.3 mmol), $D_2O$ (15.0 mmol), $^{n}Bu_4NI$ (1.0 equiv.), DMF (4.0 mL), room temperature, 10 h. [b]Isolated yield. [c]Deuterium incorporation percentages were determined by $^{1}H$ NMR spectroscopy. CF = carbon felt. DMF = *N*,*N*-dimethylformamide. DMA = *N*,*N*-dimethylacetamide.

**2.2 Supplementary Table 2:** The effect of the electrolyte[a]

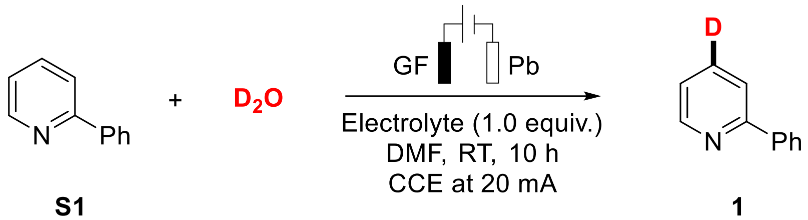


| Entry | Electrolyte | Yield of **1** or recovered **S1** (%)[b] | **1** (D%)[c] |
|---|---|---|---|
| **1** | **$^{n}Bu_4NI$** | **99** | **>99** |
| 2 | $^{n}Bu_4NBF_4$ | 83 | 99 |
| 3 | $Et_4NI$ | 99 | 90 |
| 4 | $LiClO_4$ | 99 | 0 |
| 5 | NaI | 99 | 0 |
| 6 | NaCl | 99 | 0 |
| 7 | $LiBF_4$ | 99 | 0 |
| 8 | $KPF_6$ | 99 | 0 |
| 9 | $^{n}Bu_4NBr$ | 93 | 99 |
| 10 | $^{n}Bu_4NCl$ | 88 | 96 |
| 11 | $^{n}Bu_4NOAc$ | 42 | >99 |
| 12 | $^{n}Bu_4NPF_6$ | 67 | >99 |
| 13 | $^{n}Bu_4NHSO_4$ | 76 | >99 |
| 14 | $^{n}Bu_4NHPO_4$ | 90 | 96 |

[a]Reaction conditions: undivided cell, graphite felt (GF) as anode and Pb as cathode, constant current (20 mA), **S1** (0.3 mmol), $D_2O$ (15.0 mmol), electrolyte (1.0 equiv.), DMF (4.0 mL), room temperature, 10 h. [b]Isolated yield. [c]Deuterium incorporation percentages were determined by $^{1}H$ NMR spectroscopy. DMF = *N*,*N*-dimethylformamide.

**2.3 Supplementary Table 3:** Optimization of the reaction time[a]

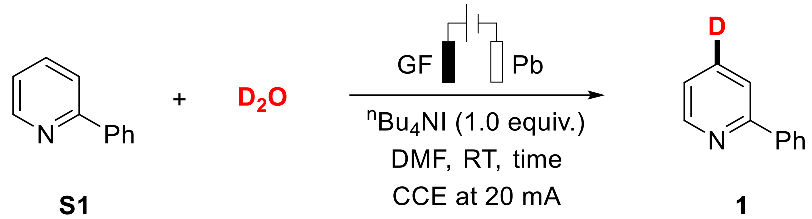


**S1** **1**

| Entry | Time | Yield of **1** or recovered **S1** (%)[b] | **1** (D%)[c] |
|---|---|---|---|
| 1 | 1 h | 99 | 21 |
| 2 | 2 h | 99 | 37 |
| 3 | 3 h | 99 | 49 |
| 4 | 4 h | 99 | 61 |
| 5 | 5 h | 99 | 70 |
| 6 | 6 h | 99 | 75 |
| 7 | 7 h | 99 | 80 |
| 8 | 8 h | 99 | 86 |
| 11 | 12 h | 99 | >99 |

[a]Reaction conditions: undivided cell, graphite felt (GF) as anode and Pb as cathode, constant current (20 mA), **S1** (0.3 mmol), $D_2O$ (15.0 mmol), $^nBu_4NI$ (1.0 equiv.), DMF (4.0 mL), room temperature. [b]Isolated yield. [c]Deuterium incorporation percentages were determined by $^1H$ NMR spectroscopy. DMF = *N*,*N*-dimethylformamide.

## 2.4 **Supplementary Table 4:** The effect of the electrode[a]

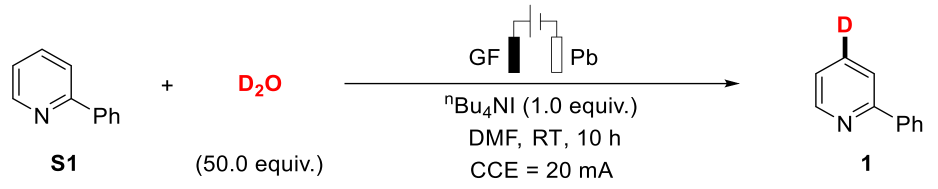

| Entry | Variation | Yield %[b] | D-inc % of **1**[c] |
| --- | --- | --- | --- |
| **1** | **None** | **99** | **>99** |
| 2 | CF (+) \| (−) Pb | 90 | 80 |
| 3 | GF (+) \| (−) GF | 99 | 35 |
| 4 | GF (+) \| (−) Pt | 99 | 53 |
| 5 | GF (+) \| (−) Ni | 99 | 22 |
| 6 | Fe (+) \| (−) Pb | trace | -- |
| 7 | Pt (+) \| (−) Pb | 99 | 90 |

[a]Reaction conditions: undivided cell, graphite felt (GF) as anode, lead plate (Pb) as cathode, constant current = 20 mA, 2-phenylpyridine **S1** (0.3 mmol), $D_2O$ (15.0 mmol, 50.0 equiv), $^nBu_4NI$ (1.0 equiv.), DMF (4.0 mL), room temperature, air, 10 h. [b]Isolated yield. [c]Deuterium incorporation percentages were determined by $^1H$ NMR spectroscopy. CF = carbon felt. DMF = *N*,*N*-dimethylformamide.

## 2.5 General procedure of electrochemical C−H deuteration of pyridine derivatives

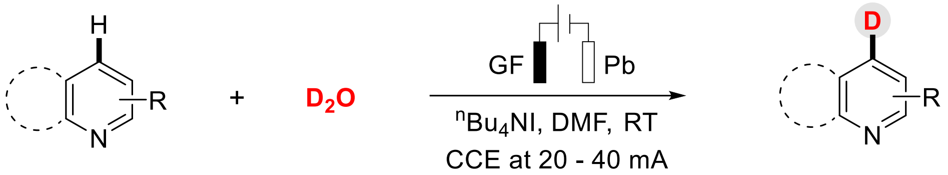


**S1-S62** **1-62**

The electrocatalysis was carried out in an undivided cell with graphite felt (GF, 10 mm × 15 mm × 5 mm) as anode and Pb (10 mm × 15 mm × 0.3 mm) as cathode. To an oven-dried undivided electrochemical cell (15 mL) equipped with a magnetic bar was added organic *N*-heteroarenes (0.3 mmol, 1.0 equiv.), $^{n}Bu_4NI$ (0.3 mmol, 110.8 mg, 1.0 equiv.) and $D_2O$ (15 mmol, 300 mg, 50.0 equiv.), then anhydrous DMF (4.0 mL) was added via a syringe. The electrocatalysis system was performed at 20~40 mA of constant current for 10 h at room temperature. After that, the reaction mixture was extracted with EtOAc (30 mL × 3) and the combined organic phase was dried by anhydrous $MgSO_4$, filtered, and concentrated in vacuo. The crude product was purified by column chromatography to furnish the deuterated products.

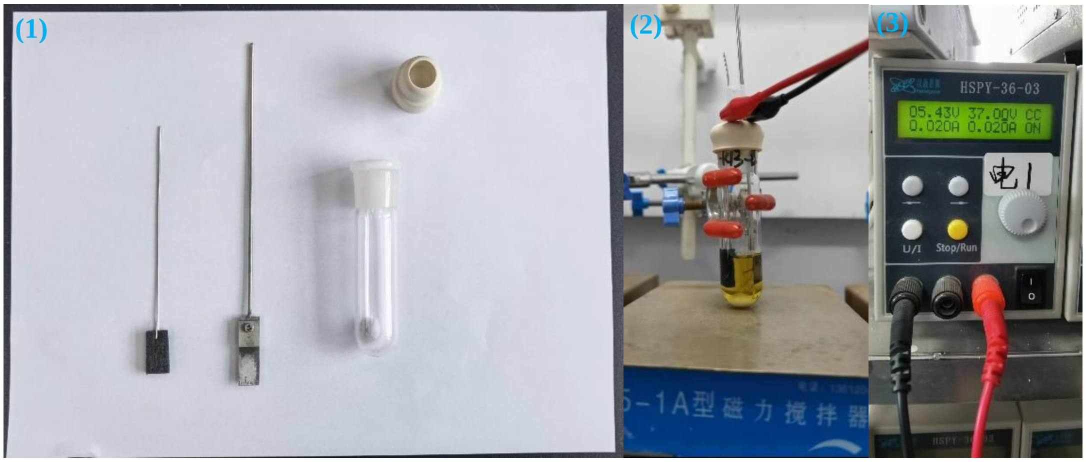


**Supplementary Figure 1.** (1) Reaction apparatus and tools. (2) General reaction apparatus. (3) Reaction potentiometer.

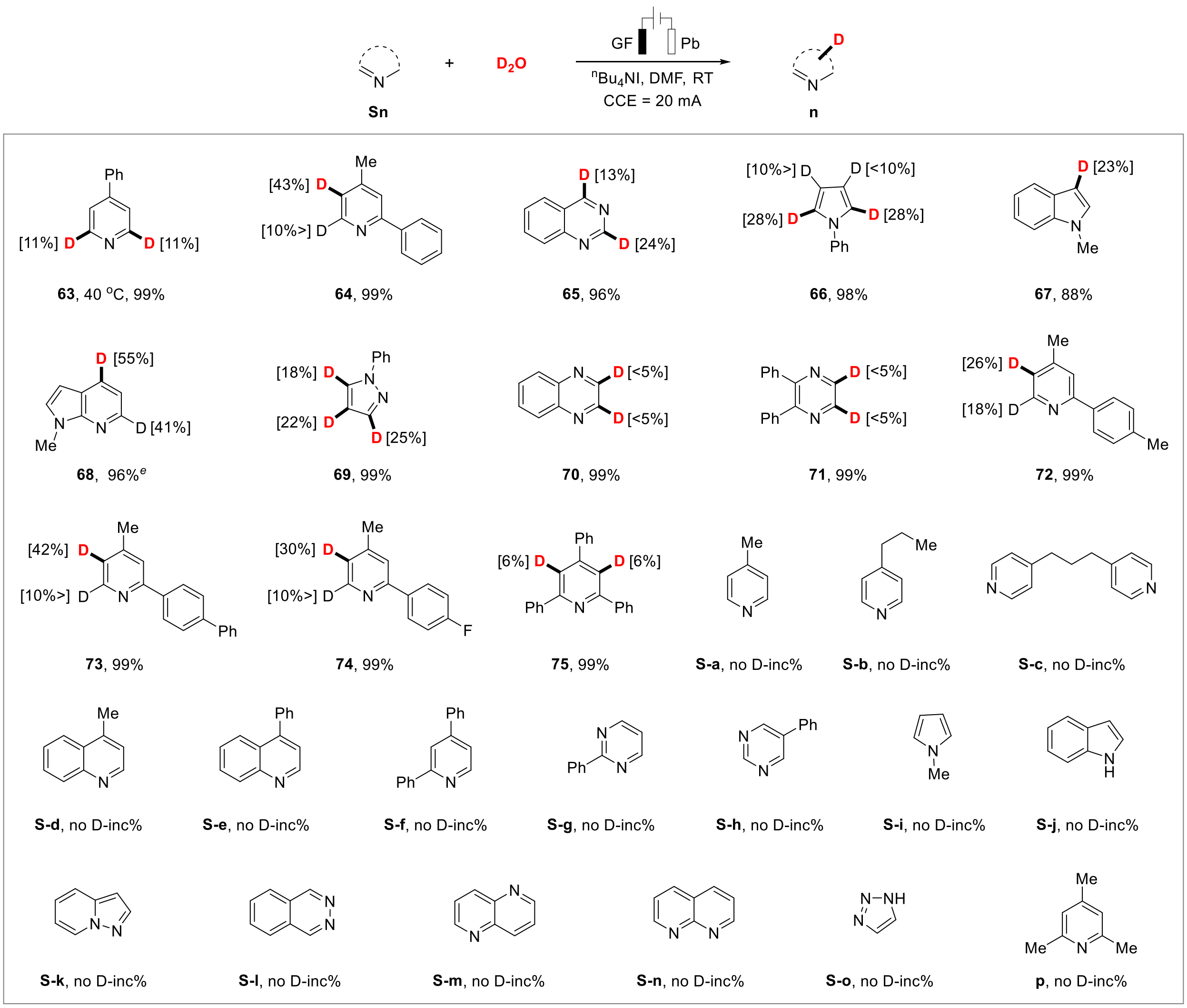


**Supplementary Figure 2.** Substrate scope of *N*-heteroarenes. Reaction conditions: [a]Electrochemical C−H deuteration of pyridines and quinolones in an undivided cell, GF as anode and Pb as cathode, constant current (20 mA), pyridine derivatives (0.3 mmol), $D_2O$ (15.0 mmol), $^{n}Bu_4NI$ (1.0 equiv.), DMF (4.0 mL), room temperature, 10 h, isolated yield. Deuterium incorporation percentages were determined by $^{1}H$ NMR spectroscopy. [c]30 mA. [d]40 mA. [e]16 h.

## 2.6 General procedure of observation of intermediate S1-a

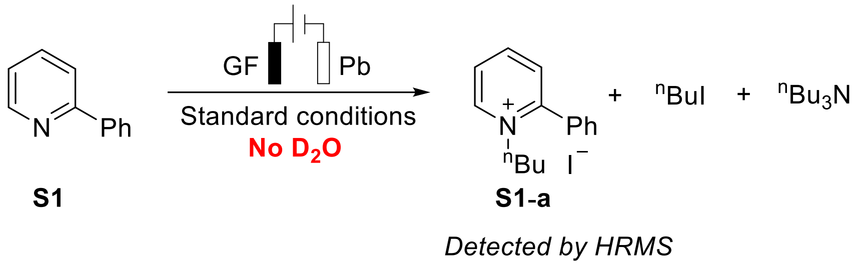


The electrocatalysis was carried out in an undivided cell with graphite felt (GF, 10 mm × 15 mm × 5 mm) as anode and Pb (10 mm × 15 mm × 0.3 mm) as cathode. To an oven-dried undivided electrochemical cell (15 mL) equipped with a magnetic bar was added 2-phenylpyridine (**S1**, 0.3 mmol, 46.5 mg, 1.0 equiv.), $^{n}Bu_4NI$ (0.3 mmol, 110.8 mg, 1.0 equiv.), then anhydrous DMF (4.0 mL) was added via a syringe. The electrocatalysis system was performed at 20 mA of constant current for 10 h at room temperature.

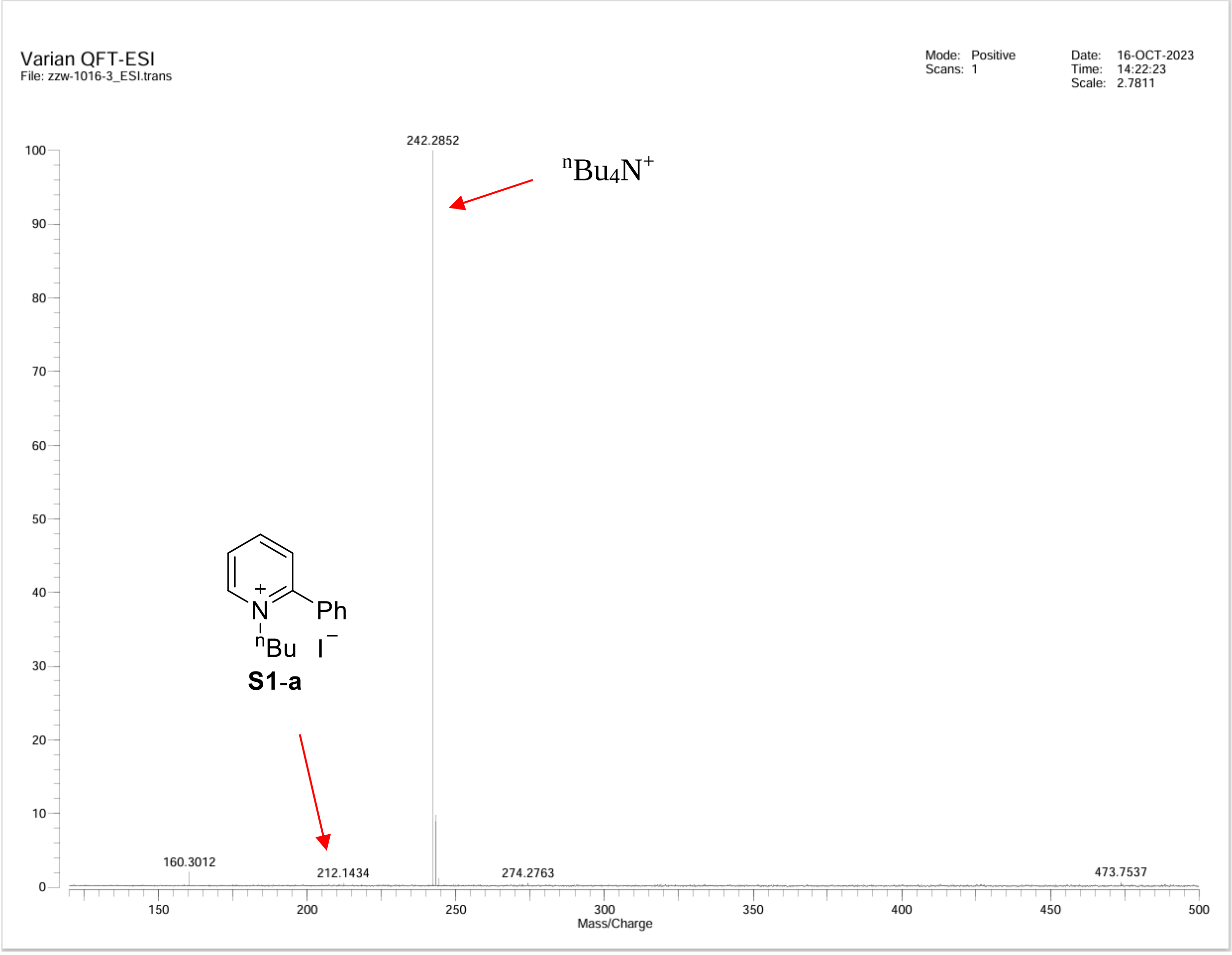

**Supplementary Figure 3.** HR-MS for **S1-a**.

## 2.7 General procedure of the preparation of *N*-alkyl-2-phenylpyridinium iodide salts[3]

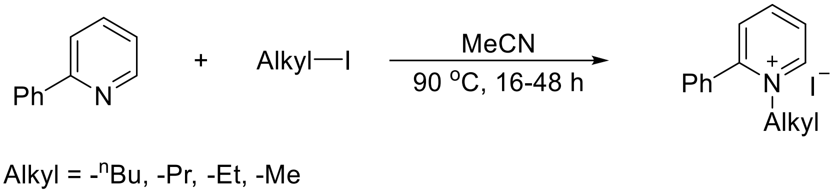


To a solution of appropriate 2-phenylpyridine (15.0 mmol, 2.328 g, 1.0 equiv.) in acetonitrile (5 mL, 3 M) was added alkyl iodide (60.0 mmol, 4.0 equiv.) under argon in a two-necked flask. The mixture was heated at 90 ℃ by heating mantle for 16–48 h, and then cooled to room temperature. After removal of the solvent under reduced pressure to afford the crude product (**S1-a**~**d**), which was purified by recrystallization in $CH_3CN$/EtOAc.

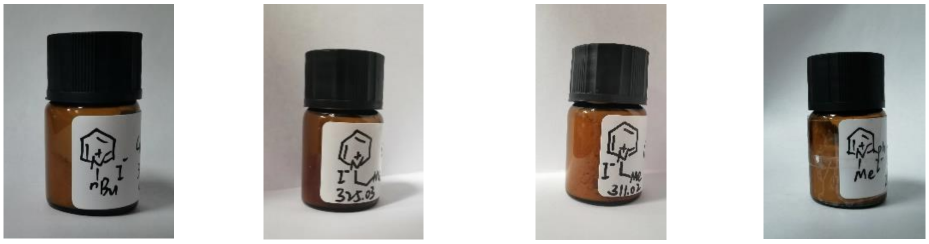

**Supplementary Figure 4.** *N*-alkyl-2-phenylpyridinium iodide salts.

## 3. Supplementary discussion

### 3.1 Mechanism research

### 3.1.1 General procedure of control experiments

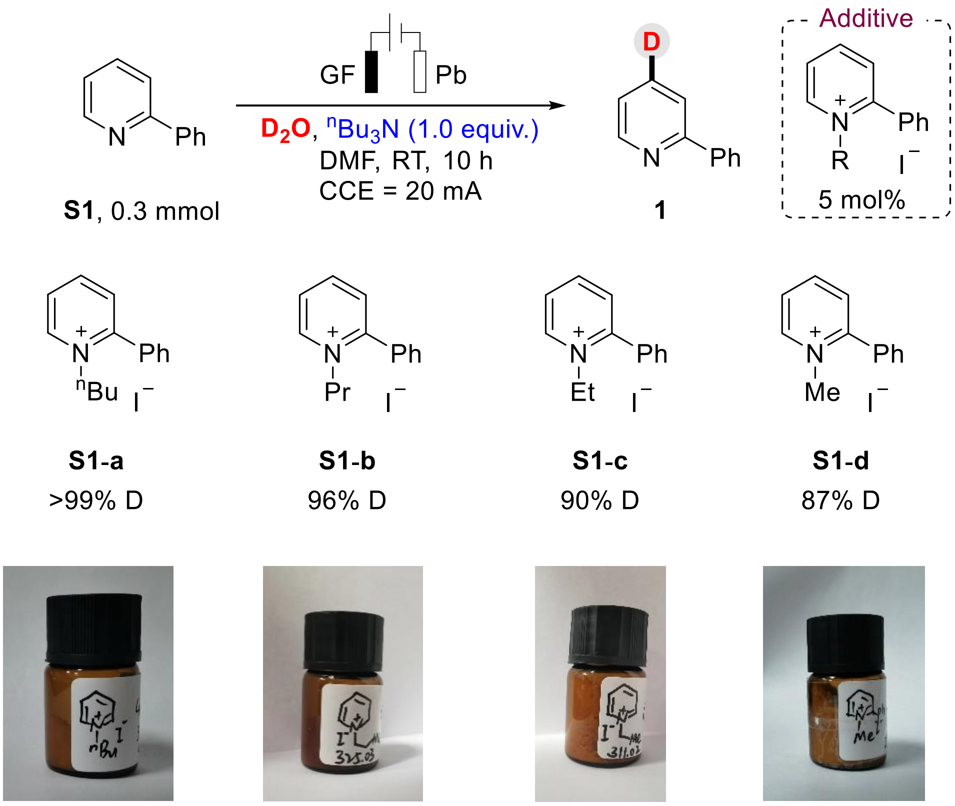


**Supplementary Figure 5.** Several *N*-alkyl-2-phenylpyridinium iodide salts and corresponding D-inc%.

The electrocatalysis was carried out in an undivided cell with graphite felt (GF, 10 mm × 15 mm × 5 mm) as anode and Pb (10 mm × 15 mm × 0.3 mm) as cathode. To an oven-dried undivided electrochemical cell (15 mL) equipped with a magnetic bar was added 2-phenylpyridine (0.3 mmol, 46.5 mg, 1.0 equiv.), additive (*N*-alkyl-2-phenylpyridinium iodide salts, **S1-a~d**, 5 mol%), $^{n}Bu_3N$ (0.3 mmol, 55.6 mg, 1.0 equiv.) and $D_2O$ (15 mmol, 300 mg, 50.0 equiv.), then anhydrous DMF (4.0 mL) was added via a syringe. The electrocatalysis system was performed at 20 mA of constant current for 10 h at room temperature. After that, the reaction mixture was extracted with EtOAc (30 mL × 3) and the combined organic phase was dried by anhydrous $MgSO_4$, filtered, and concentrated in vacuo. The crude product was purified by column chromatography to furnish the deuterated product **1**.

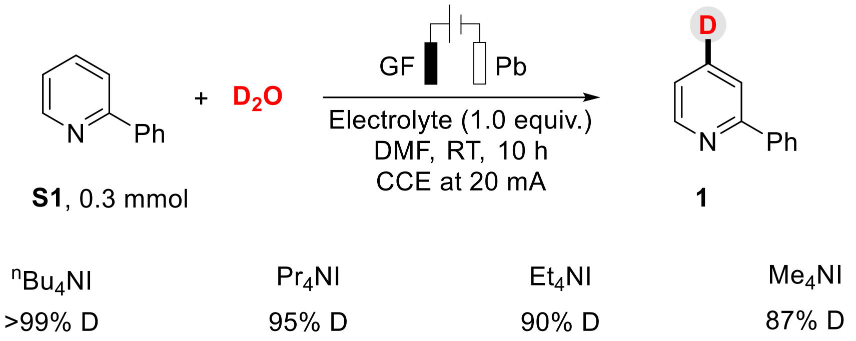


**Supplementary Figure 6.** Several electrolytes and corresponding D-inc%.

The electrocatalysis was carried out in an undivided cell with graphite felt (GF, 10 mm × 15 mm × 5 mm) as anode and Pb (10 mm × 15 mm × 0.3 mm) as cathode. To an oven-dried undivided electrochemical cell (15 mL) equipped with a magnetic bar was added 2-phenylpyridine (0.3 mmol, 46.5 mg, 1.0 equiv.), electrolyte (0.3 mmol, 1.0 equiv.), and $D_2O$ (15 mmol, 300 mg, 50.0 equiv.), then anhydrous DMF (4.0 mL) was added via a syringe. The electrocatalysis system was performed at 20 mA of constant current for 10 h at room temperature. After that, the reaction mixture was extracted with EtOAc (30 mL × 3) and the combined organic phase was dried by anhydrous $MgSO_4$, filtered, and concentrated in vacuo. The crude product was purified by column chromatography to furnish the deuterated product **1**.

### 3.1.2 General procedure of gram-scale reaction

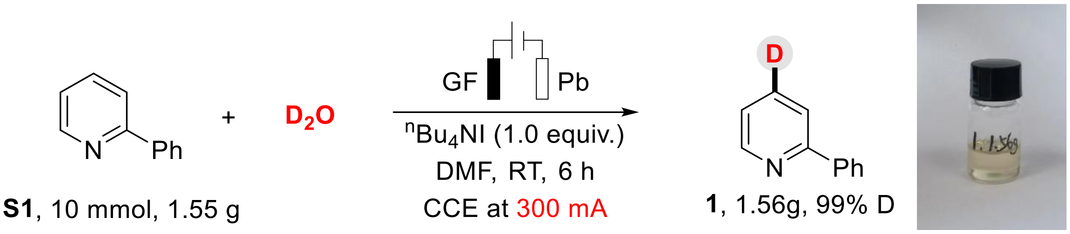


The electrocatalysis was carried out in an undivided cell with graphite felt (GF, 30 mm × 45 mm × 5 mm) as anode and Pb (30 mm × 45 mm × 0.3 mm) as cathode. To an oven-dried undivided electrochemical cell (200 mL) equipped with a magnetic bar was added 2-phenylpyridine (**S1**, 10 mmol, 1.55 g, 1.0 equiv.), $^{n}Bu_4NI$ (10 mmol, 3.70 g, 1.0 equiv.) and $D_2O$ (500 mmol, 10 g, 50.0 equiv.). Then anhydrous DMF (80 mL) was added via a syringe. The electrocatalysis system was performed at 300.0 mA of constant current for 6 h at room temperature. After that, the reaction mixture was extracted with EtOAc (60 ml × 3) and the combined organic phase were dried by

anhydrous $MgSO_4$, filtered, and concentrated in vacuo. The crude product was purified by column chromatography to furnish the desired deuterated product **1** (1.56 g, 99% D).

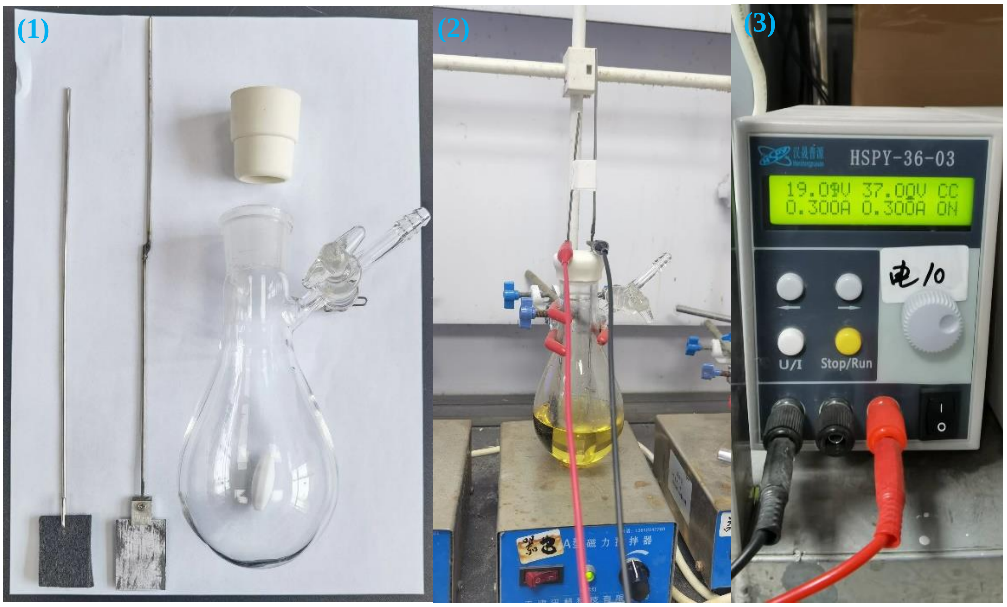


**Supplementary Figure 7.** (1) Reaction apparatus and tools. (2) General reaction apparatus. (3) Reaction potentiometer.

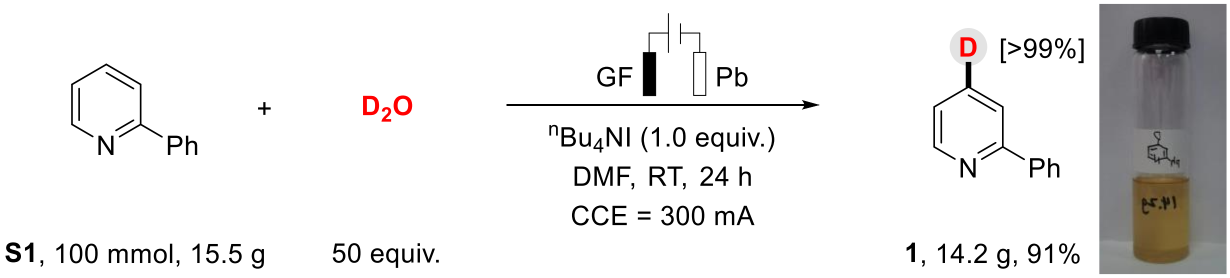


The electrocatalysis was carried out in an undivided cell with graphite felt (GF, 30 mm × 45 mm × 5 mm) as anode and Pb (30 mm × 45 mm × 0.3 mm) as cathode. To an oven-dried undivided electrochemical cell (1000 mL) equipped with a magnetic bar was added 2-phenylpyridine (**S1**, 100 mmol, 15.5 g, 1.0 equiv.), $^nBu_4NI$ (100 mmol, 36.94 g, 1.0 equiv.) and $D_2O$ (100 g, 50.0 equiv.). Then anhydrous DMF (400 mL) was added *via* a syringe. The electrocatalysis system was performed at 300.0 mA of constant current for 24 h at room temperature. After that, the reaction mixture was divided into many parts and extracted with EtOAc (60 ml × 40) and the combined organic phase were dried by anhydrous $MgSO_4$, filtered, and concentrated in vacuo.

The crude product was purified by column chromatography to furnish the desired deuterated product **1** (14.2 g, 91%, >99% D).

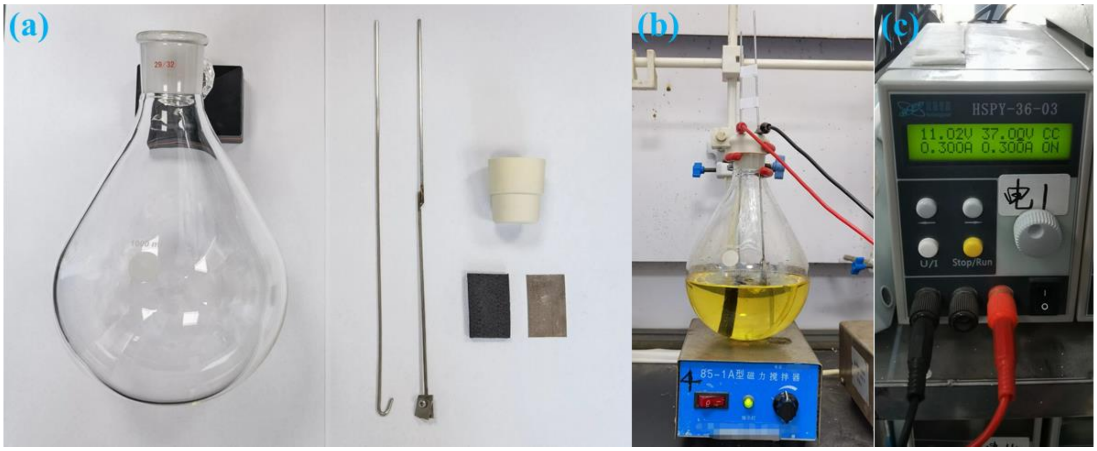


**Supplementary Figure 8.** (a) Reaction apparatus and tools. (b) General reaction apparatus. (c) Reaction potentiometer.

### 3.1.3 General procedure of flow-synthesis reaction

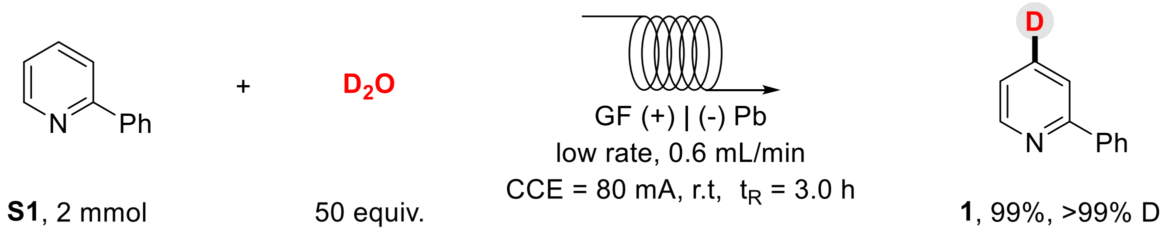


### Supplementary method for the flow-synthesis electrolysis

The substrate **S1** (2.0 mmol, 0.3104 g), $^{n}Bu_4NI$ (2.0 mmol, 0.7388 g) and $D_2O$ (50.0 equiv, 2 g) in dry DMF under air was pushed *via* a micro syringe pump to pass though the flow electrolytic cell with a flow rate of 0.6 mL $min^{-1}$ and a 80 mA current to react for 3 h. After that, the reaction mixture was extracted with EtOAc (100 ml × 3) and the combined organic phase were dried by anhydrous $MgSO_4$, filtered, and concentrated in vacuo. The crude product was purified by column chromatography to furnish the desired deuterated product **1**.

### Supplementary method: design of the flow-synthesis reaction microreactor

The flow electrolysis cell is assembled using two aluminum bodies (**a**, 75 mm x 75 mm x 15 mm) with a groove (50 mm x 50 mm x 3.0 mm). The cathode (**b**, middle and right) consists a piece of Pb foil (50 mm x 50 mm x 0.30 mm thickness) fixed on a stainless steel base (49 mm x 49 mm x

3.0 mm). The anode (**b**, left), which is made of carbon base (49 mm x 49 mm x 5.0 mm), is insulated from the aluminum body by silicone film. The anode and cathode are held apart by a fluorinated ethylene propylene (FEP) foil (**e**) of 0.1 mm thickness. The whole device is held together by steel screws and wing nuts. The reaction mixture flows in and out through inlet and outlet (**a**, left, red circle). The reaction setup is shown in Fig. R12

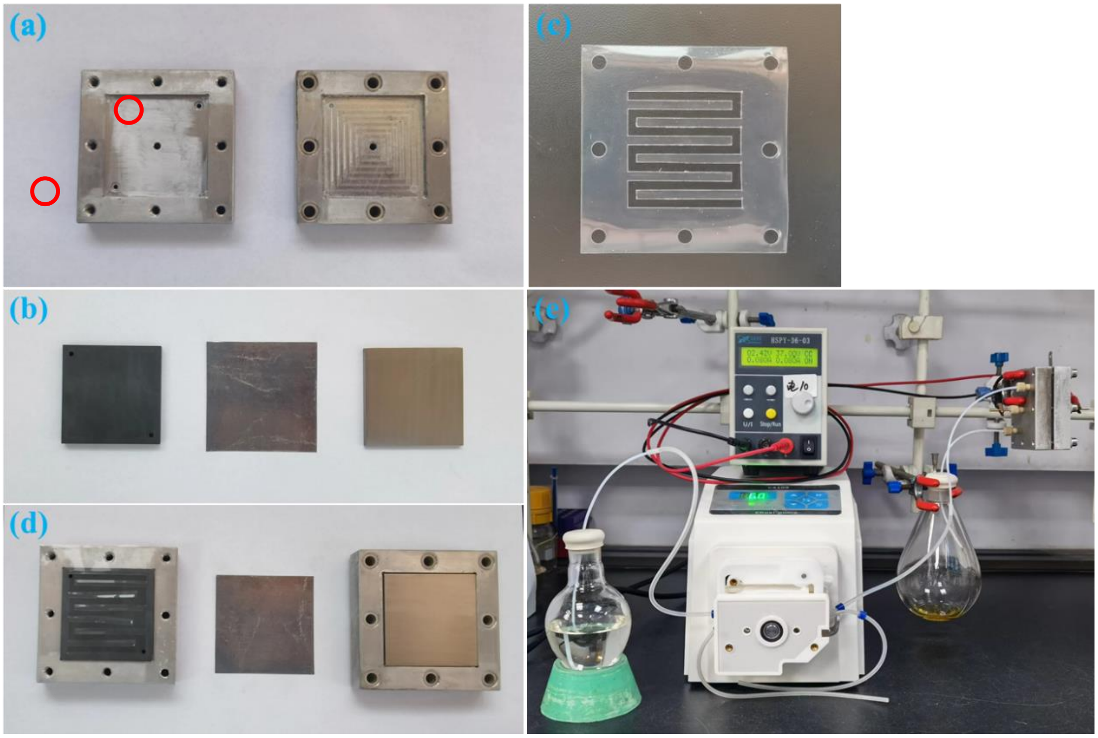


**Supplementary Figure 9.** (a). Aluminum bodies with a groove. (b) Electrode materials. (c) FEP spacer. (d) Anode and cathode. (e) Reaction setup

### 3.1.4 General procedure of D/H exchange experiments

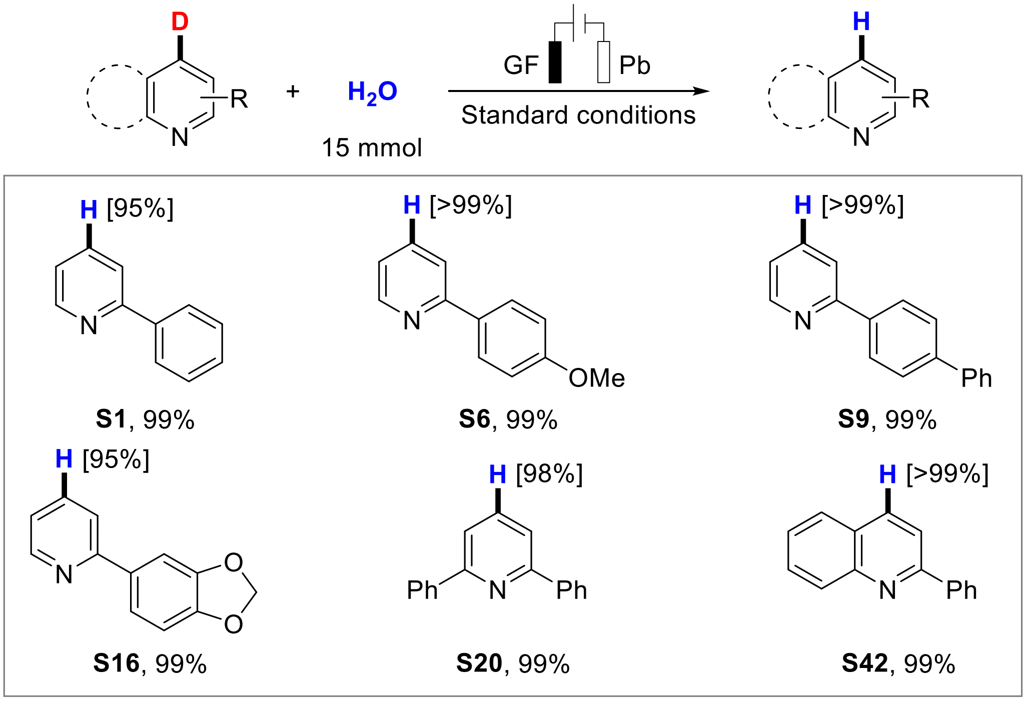


**Supplementary Figure 10.** D/H exchange substrates.

The electrocatalysis was carried out in an undivided cell with graphite felt (GF, 10 mm × 15 mm × 5 mm) as anode and Pb (10 mm × 15 mm × 0.3 mm) as cathode. To an oven-dried undivided electrochemical cell (15 mL) equipped with a magnetic bar was added organic deuterated pyridine derivatives (**1**, **6**, **9**, **16**, **20**, **42**, 0.3 mmol, 1.0 equiv.), $^{n}Bu_4NI$ (0.3 mmol, 110.8 mg, 1.0 equiv.) and $H_2O$ (15 mmol, 270 mg, 50.0 equiv.), then anhydrous DMF (4.0 mL) was added via a syringe. The electrocatalysis system was performed at 20 mA of constant current for 10 h at room temperature. After that, the reaction mixture was extracted with EtOAc (30 mL × 3) and the combined organic phase was dried by anhydrous $MgSO_4$, filtered, and concentrated in vacuo. The crude product was purified by column chromatography to furnish the initial materials (**S1**, **S6**, **S9**, **S16**, **S20**, **S42**).

**3.1.5 Supplementary Table 5:** H/D exchange experiments[a]

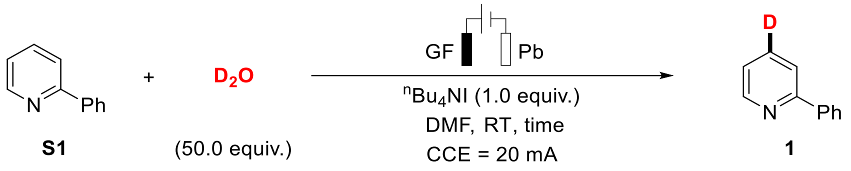


| Entry | Time (h) | Yield %[b] | D-inc % of **1**[c] |
|---|---|---|---|
| 1 | 0 | 0 | 0 |
| 2 | 0.5 | 99 | 12 |
| 3 | 1.0 | 99 | 21 |
| 4 | 1.5 | 99 | 30 |
| 5 | 2.0 | 99 | 37 |
| 6 | 2.5 | 99 | 44 |
| 7 | 3.0 | 99 | 50 |
| 8 | 3.5 | 99 | 56 |
| 9 | 4.0 | 99 | 61 |

[a]Reaction conditions: undivided cell, graphite felt (GF) as anode, lead plate (Pb) as cathode, constant current = 20 mA, 2-phenylpyridine **S1** (0.3 mmol), $D_2O$ (15.0 mmol, 50.0 equiv), $^nBu_4NI$ (1.0 equiv.), DMF (4.0 mL), room temperature, air. [b]Isolated yield. [c]Deuterium incorporation percentages were determined by $^1H$ NMR spectroscopy. CF = carbon felt. DMF = *N*,*N*-dimethylformamide.

**3.1.6 Supplementary Table 6**: D/H exchange experiments[a]

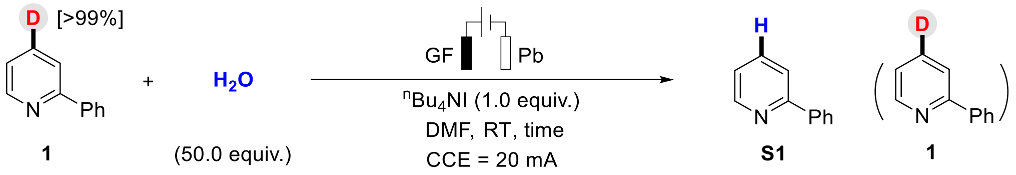


| Entry | Time (h) | Yield %[b] | H-inc % of S1 (D-inc% of 1)[c] |
|---|---|---|---|
| 1 | 0 | 0 | 0 (>99) |
| 2 | 0.5 | 99 | 17 (83) |
| 3 | 1.0 | 99 | 32 (68) |

| | | | |
|---|---|---|---|
| 4 | 1.5 | 99 | 44 (56) |
| 5 | 2.0 | 99 | 54 (46) |
| 6 | 2.5 | 99 | 61(39) |
| 7 | 3.0 | 99 | 65 (35) |
| 8 | 3.5 | 99 | 68 (32) |
| 9 | 4.0 | 99 | 71 (29) |

[a]Reaction conditions: undivided cell, graphite felt (GF) as anode, lead plate (Pb) as cathode, constant current = 20 mA, **1** (0.3 mmol), $D_2O$ (15.0 mmol, 50.0 equiv), $^nBu_4NI$ (1.0 equiv.), DMF (4.0 mL), room temperature, air. [b]Isolated yield. [c]Deuterium incorporation percentages were determined by $^1H$ NMR spectroscopy. CF = carbon felt. DMF = *N*,*N*-dimethylformamide.

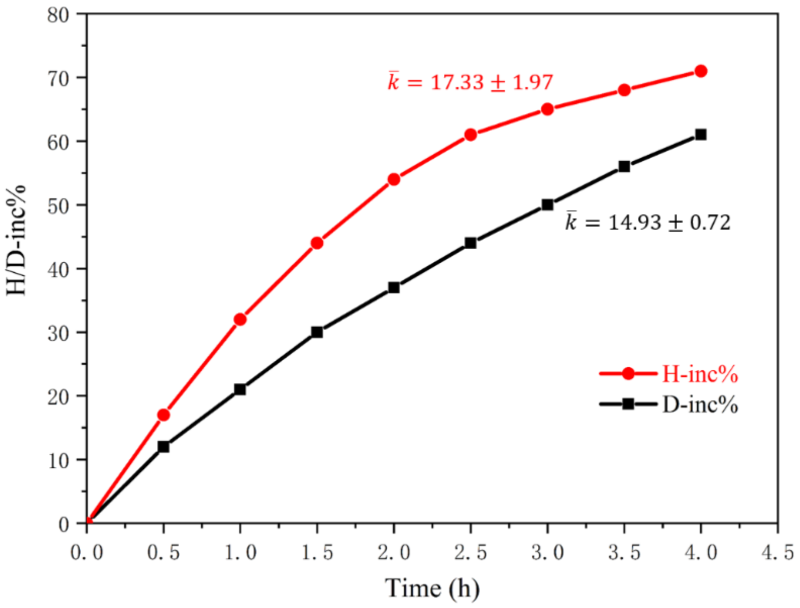


**Supplementary Figure 11.** The H/D exchange (black line) and D/H exchange (red line) rate studies.

### 3.1.7 General procedure of competition experiments

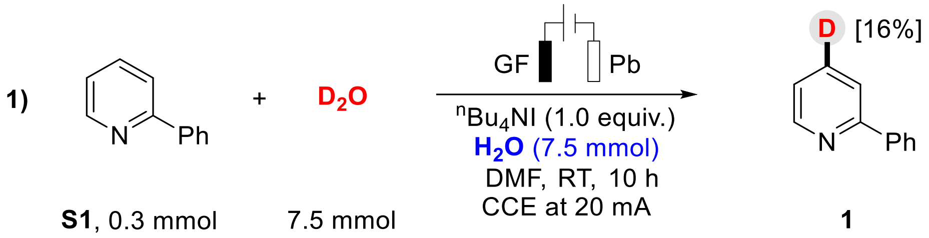

The electrocatalysis was carried out in an undivided cell with graphite felt (GF, 10 mm × 15 mm × 5 mm) as anode and Pb (10 mm × 15 mm × 0.3 mm) as cathode. To an oven-dried undivided electrochemical cell (15 mL) equipped with a magnetic bar was added 2-phenylpyridine (**S1**, 0.3 mmol, 1.0 equiv.), $^{n}Bu_4NI$ (0.3 mmol, 110.8 mg, 1.0 equiv.), $D_2O$ (7.5 mmol, 150.0 mg, 25.0 equiv.) and $H_2O$ (7.5 mmol, 135.0 mg, 25.0 equiv.), then anhydrous DMF (4.0 mL) was added via a syringe. The electrocatalysis system was performed at 20 mA of constant current for 10 h at room temperature. After that, the reaction mixture was extracted with EtOAc (30 mL × 3) and the combined organic phase was dried by anhydrous $MgSO_4$, filtered, and concentrated in vacuo. The crude product was purified by column chromatography to furnish the deuterated product **1**.

$^{1}$H NMR and $^{13}$C NMR of **1** (**h**, competition experiment **1**)

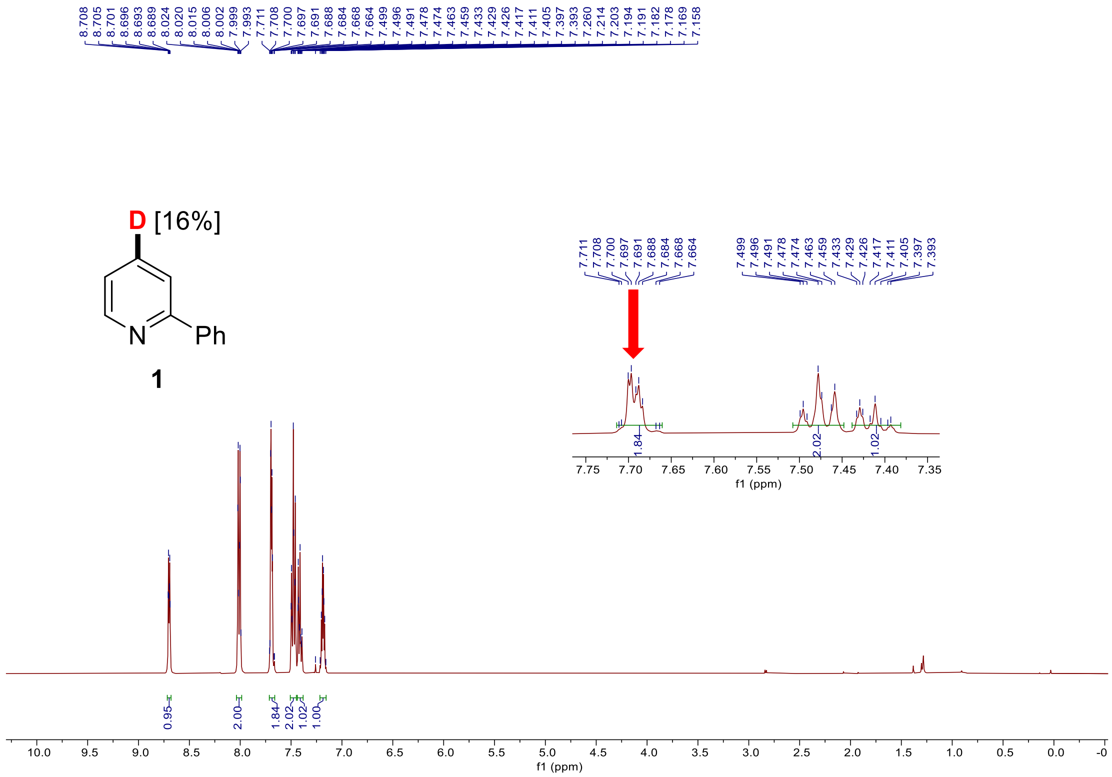


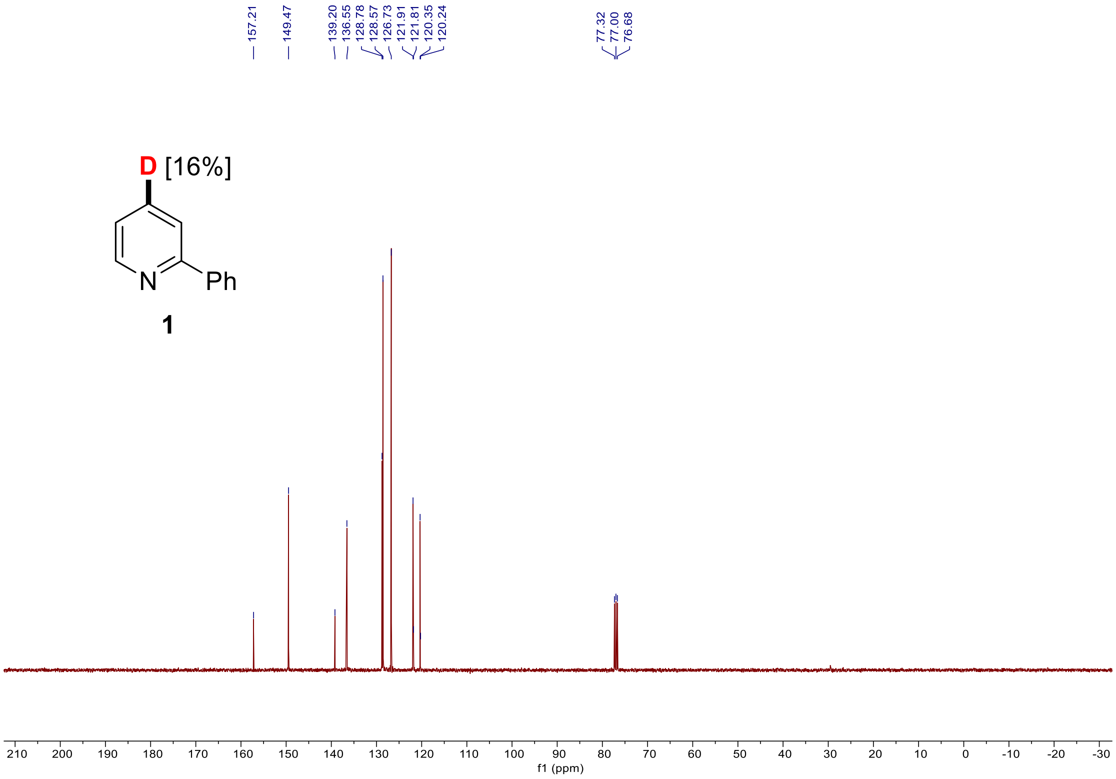

2)

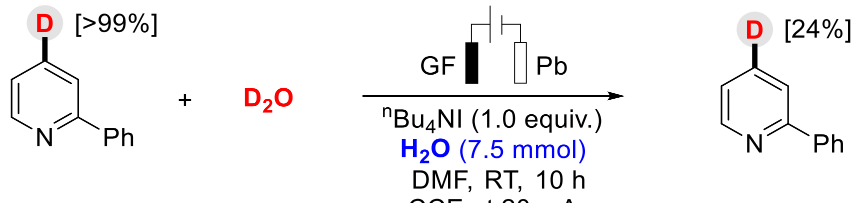


**1**, 0.3 mmol    7.5 mmol    **1**

The electrocatalysis was carried out in an undivided cell with graphite felt (GF, 10 mm × 15 mm × 5 mm) as anode and Pb (10 mm × 15 mm × 0.3 mm) as cathode. To an oven-dried undivided electrochemical cell (15 mL) equipped with a magnetic bar was added the D-labeled product (**1**, 0.3 mmol, 1.0 equiv.), $^{n}Bu_4NI$ (0.3 mmol, 110.8 mg, 1.0 equiv.), $D_2O$ (7.5 mmol, 150.0 mg, 25.0 equiv.) and $H_2O$ (7.5 mmol, 135.0 mg, 25.0 equiv.), then anhydrous DMF (4.0 mL) was added via a syringe. The electrocatalysis system was performed at 20 mA of constant current for 10 h at room temperature. After that, the reaction mixture was extracted with EtOAc (30 mL × 3) and the combined organic phase was dried by anhydrous $MgSO_4$, filtered, and concentrated in vacuo. The crude product was purified by column chromatography to furnish the deuterated product **1**.

**$^{1}$H NMR** and **$^{13}$C NMR** of **1** (**h**, competition experiment **2**)

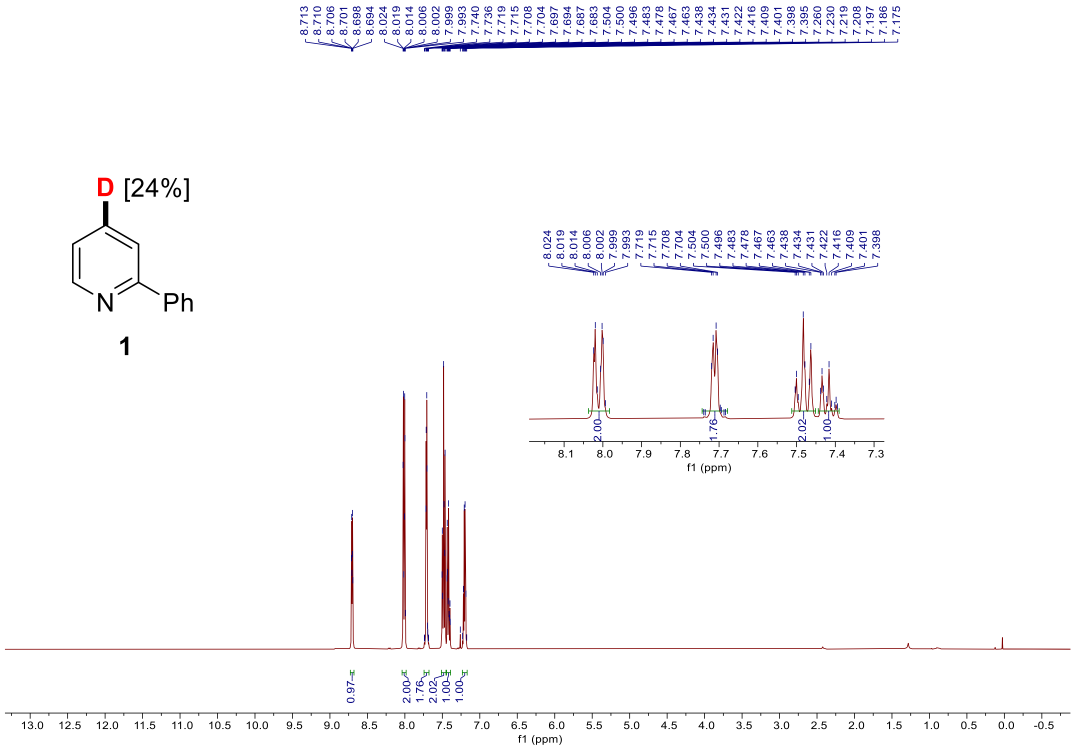


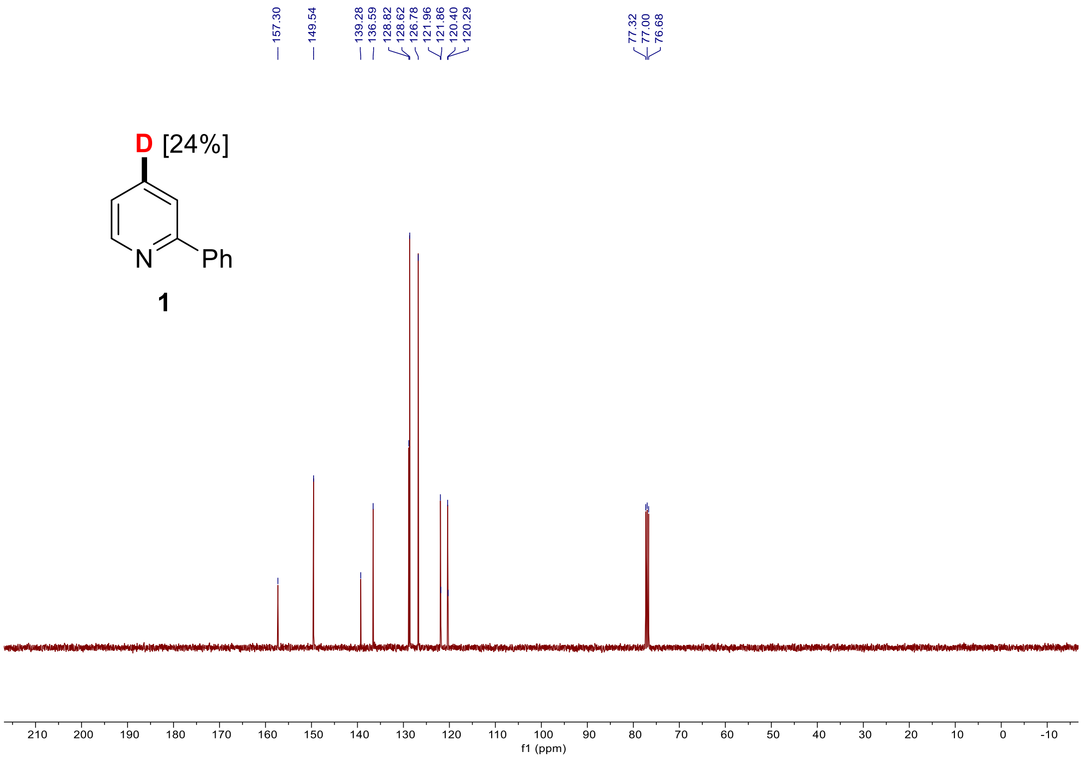

3)

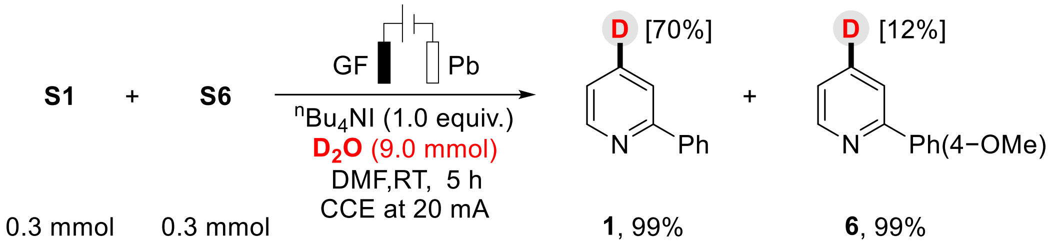


The electrocatalysis was carried out in an undivided cell with graphite felt (GF, 10 mm × 15 mm × 5 mm) as anode and Pb (10 mm × 15 mm × 0.3 mm) as cathode. To an oven-dried undivided electrochemical cell (15 mL) equipped with a magnetic bar was added initial substrates **S1** (0.3 mmol, 46.5 mg, 1.0 equiv.), **S6** (0.3 mmol, 55.5 mg, 1.0 equiv.), $^nBu_4NI$ (0.3 mmol, 110.8 mg, 1.0 equiv.) and $D_2O$ (9.0 mmol, 180.0 mg, 30.0 equiv.), then anhydrous DMF (4.0 mL) was added via a syringe. The electrocatalysis system was performed at 20 mA of constant current for 10 h at room temperature. After that, the reaction mixture was extracted with EtOAc (30 mL × 3) and the combined organic phase was dried by anhydrous $MgSO_4$, filtered, and concentrated in vacuo. The crude product was purified by column chromatography to furnish the deuterated products **1** and **6** respectively.

**$^{1}$H NMR** and **$^{13}$C NMR** of **1** (**h**, competition experiment **3**)

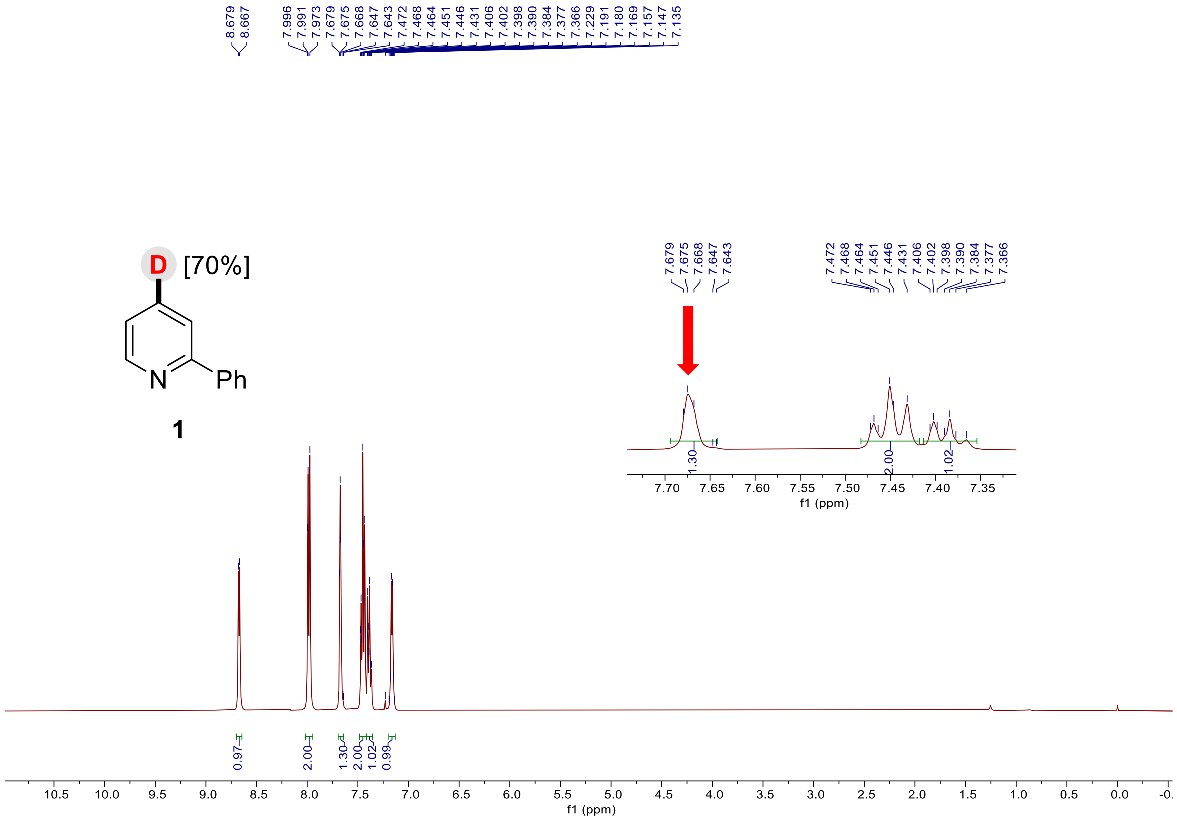


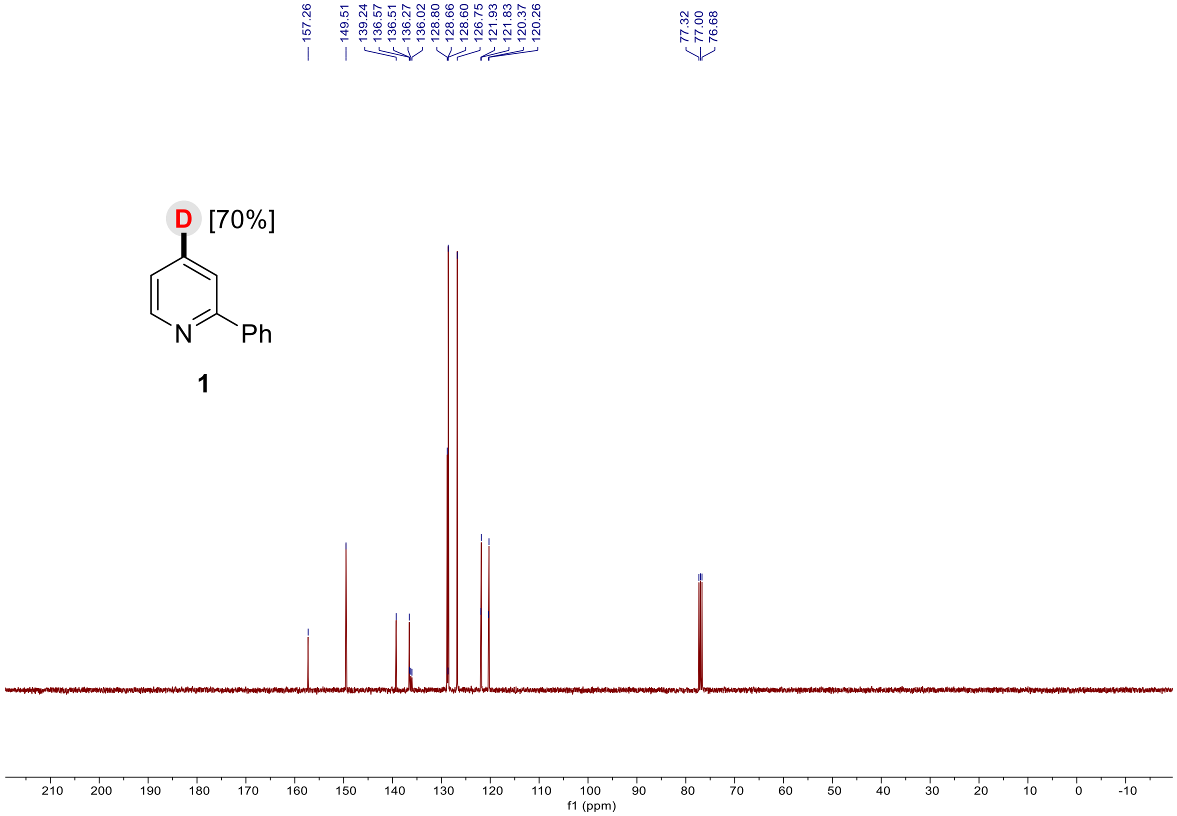

**$^{1}$H NMR** and **$^{13}$C NMR** of **6** (**h**, competition experiment **3**)

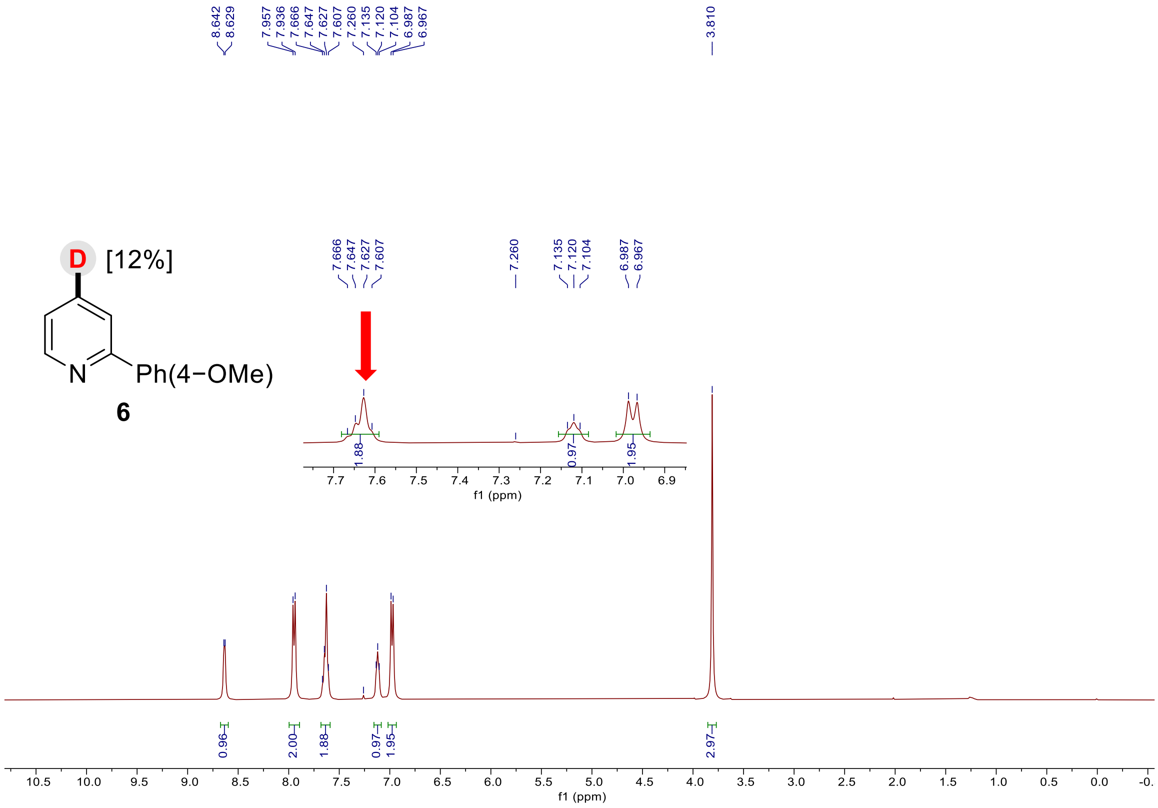


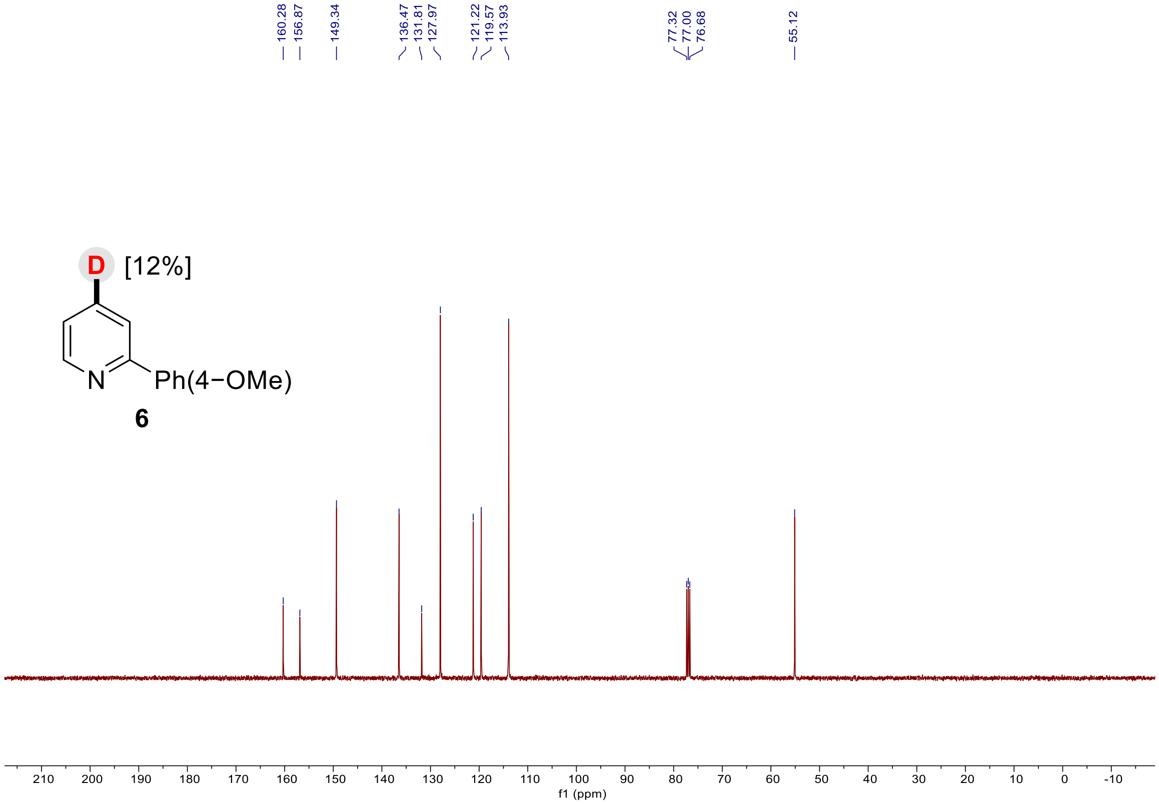

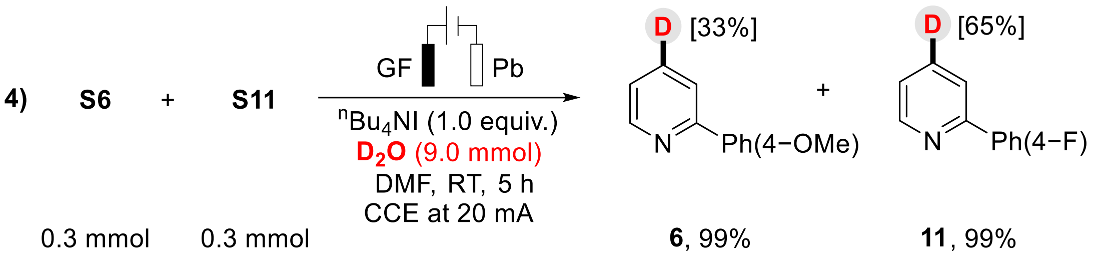


The electrocatalysis was carried out in an undivided cell with graphite felt (GF, 10 mm × 15 mm × 5 mm) as anode and Pb (10 mm × 15 mm × 0.3 mm) as cathode. To an oven-dried undivided electrochemical cell (15 mL) equipped with a magnetic bar was added initial substrates **S6** (0.3 mmol, 55.5 mg, 1.0 equiv.), **S11** (0.3 mmol, 51.9 mg, 1.0 equiv.), $^{n}Bu_4NI$ (0.3 mmol, 110.8 mg, 1.0 equiv.) and $D_2O$ (9.0 mmol, 180.0 mg, 30.0 equiv.), then anhydrous DMF (4.0 mL) was added via a syringe. The electrocatalysis system was performed at 20 mA of constant current for 10 h at room temperature. After that, the reaction mixture was extracted with EtOAc (30 mL × 3) and the combined organic phase was dried by anhydrous $MgSO_4$, filtered, and concentrated in vacuo. The crude product was purified by column chromatography to furnish the deuterated products **6** and **11** respectively.

**$^{1}$H NMR** and **$^{13}$C NMR** of **6** (**h**, competition experiment **4**)

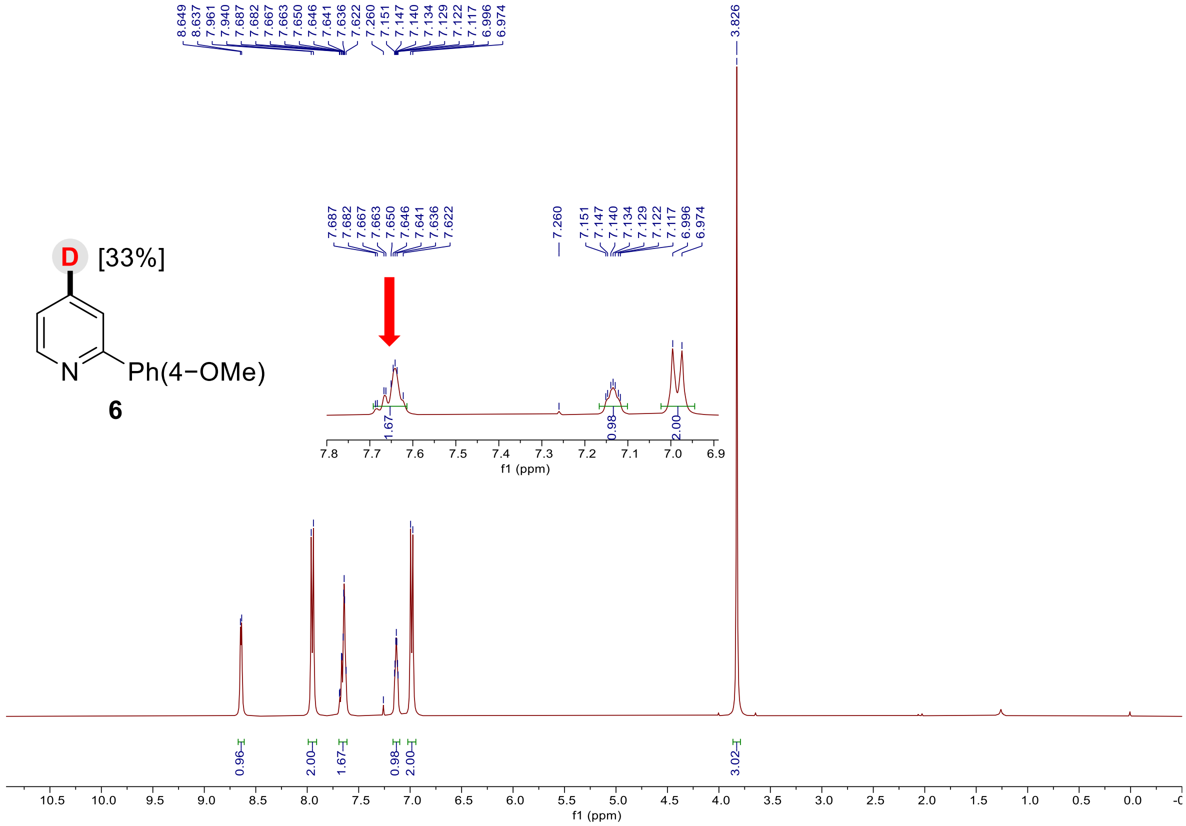


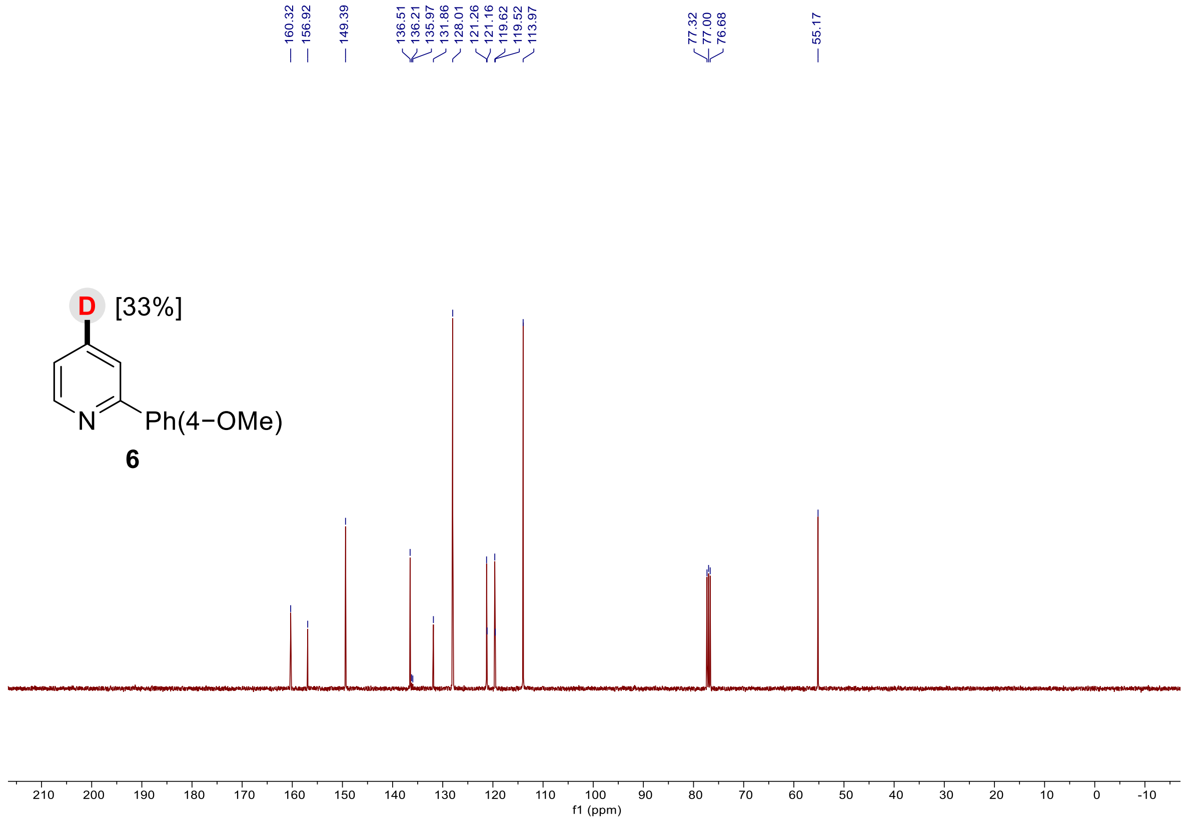

**$^{1}$H NMR** and **$^{13}$C NMR** of **11** (**h**, competition experiment **4**)

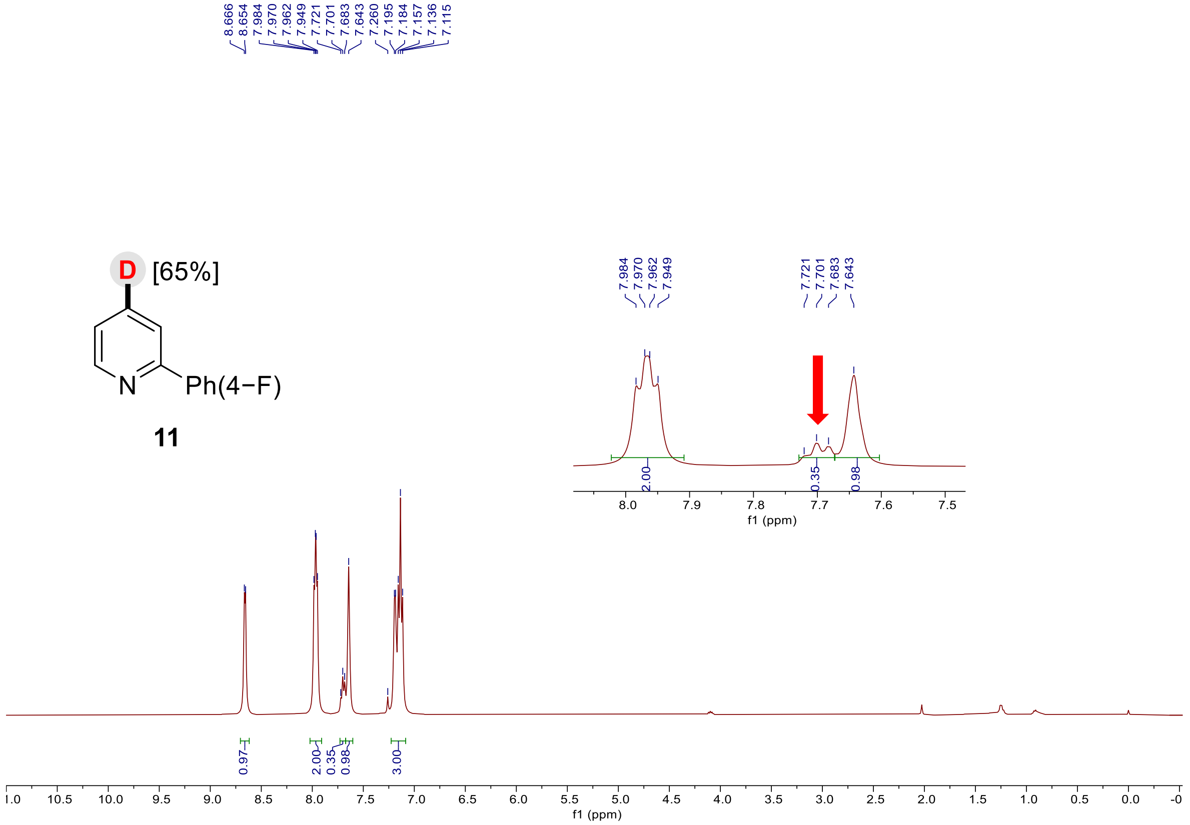


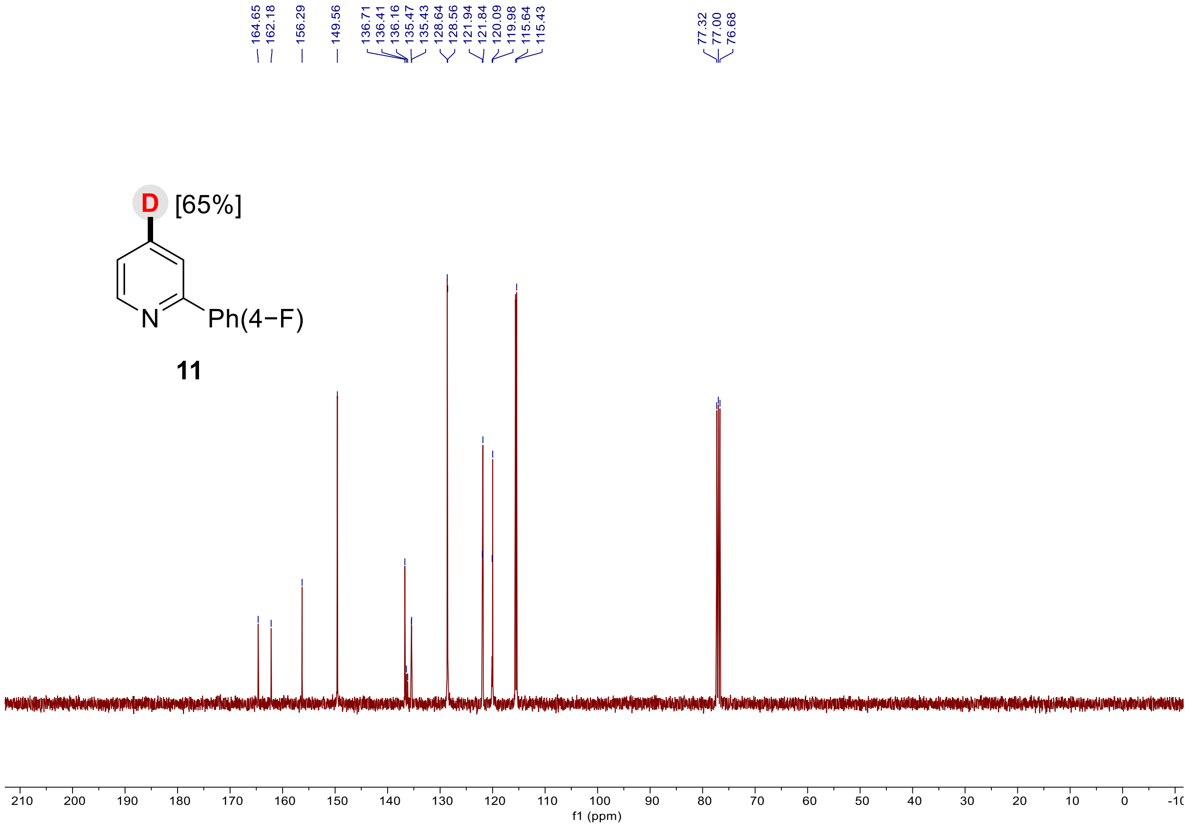

### 3.2 Cyclic voltammetry

The cyclic voltammetry was carried out with a Shanghai Chenhua CHI760E workstation. A glassy-carbon electrode (5 mm-diameter, disc-electrode) was used as the working electrode, a Pt plate was used as the auxiliary electrode and an $Ag/Ag^{+}$ electrode was used as a reference electrode. All samples should be bubbled with Ar for 5 min before testing. The measurements were carried out at a scan rate of 100 mV $s^{-1}$ in DMF/$^{n}Bu_4NBF_4$ (0.1 M) under reduction conditions or MeCN/$KPF_6$ (0.1 M) under oxidation conditions.

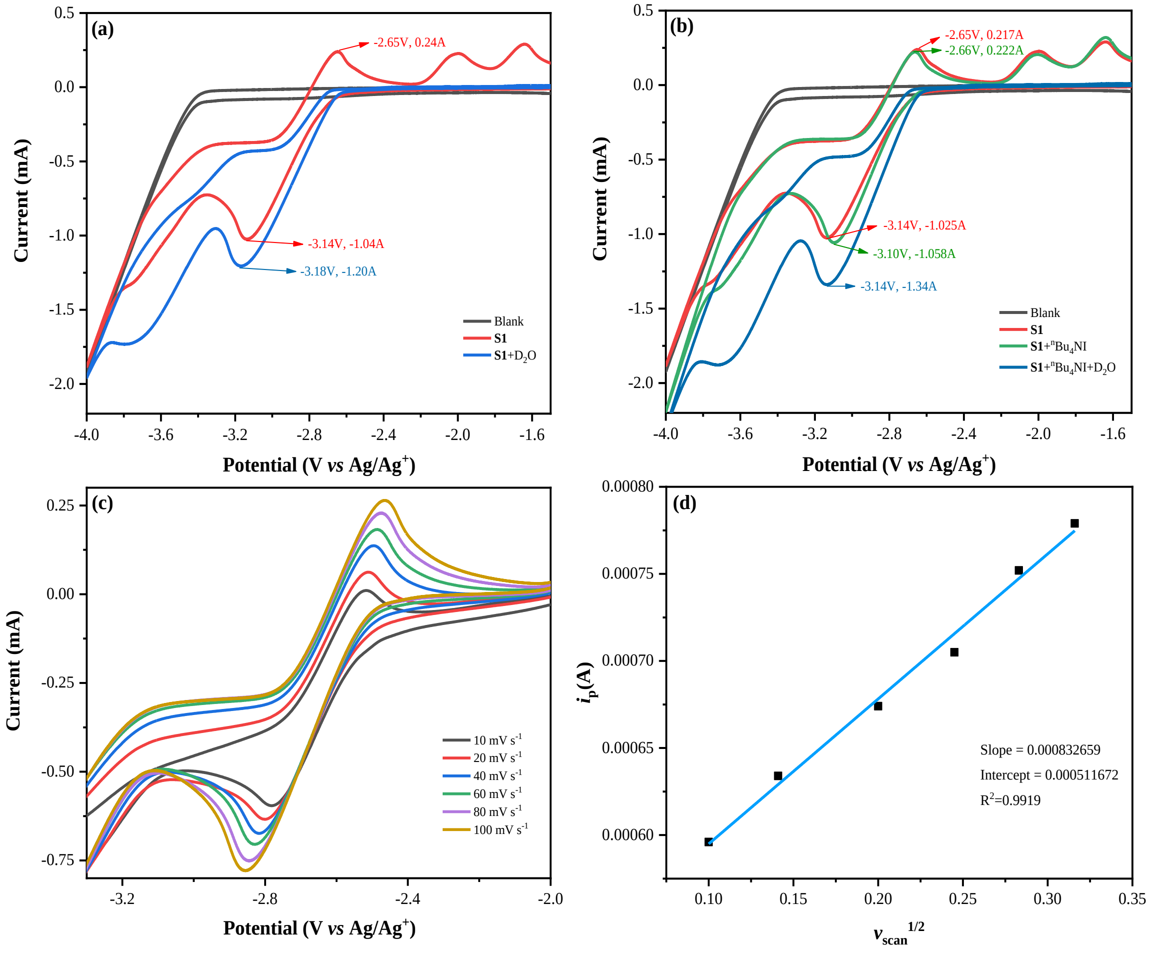


**Supplementary Figure 12.** CV experiments, using glass carbon as work electrode, Pt plate and $Ag/Ag^{+}$ as counter and reference electrodes. Scan rate: 100 mV $s^{-1}$. Solvent: DMF/$^{n}Bu_4NBF_4$ (0.1 M) or MeCN/$KPF_6$ (0.1 M). Experiments were conducted under Ar unless otherwise noted. (a) CVs of **S1** and **S1** with $D_2O$. (b) CVs of **S1**, **S1** with $^{n}Bu_4NI$ and the reaction system. (c) CVs of **S1** performed at variable scan rates ranging from 10 mV $s^{-1}$ to 100 mV $s^{-1}$. (d) Linear fit analysis of $v_{scan}^{1/2}$ and $i_p$.

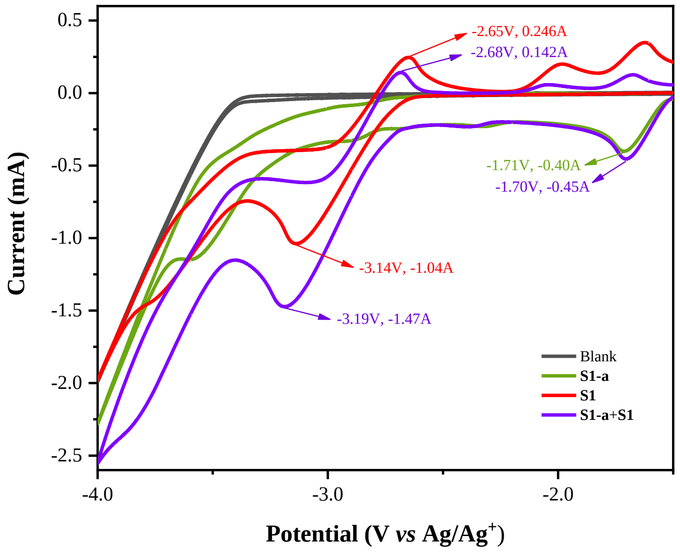


**Supplementary Figure 13.** Cyclic voltammograms of **S1-a** (*N*-butyl-2-phenylpyridinium iodide), **S1** (2-phenylpyridine) and the mixture of them in DMF with $^{n}Bu_4NBF_4$ (0.1 M) in Ar atmosphere.

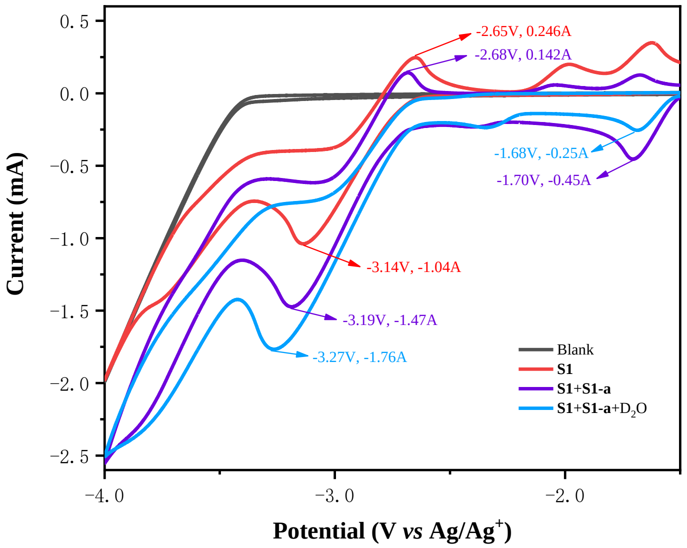


**Supplementary Figure 14.** Cyclic voltammograms of **S1** (2-phenylpyridine), **S1** with **S1-a** (*N*-butyl-2-phenylpyridinium iodide) and both of them with $D_2O$ in DMF with $^{n}Bu_4NBF_4$ (0.1 M) in Ar atmosphere.

### 3.3 Characterization of products

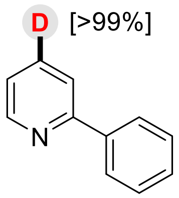


***2-Phenylpyridine-4-d (1)***

The compounds were prepared following a general procedure and purified on silica gel (petroleum ethers ethyl acetate = 30:1) by column chromatography to obtain (46.4 mg, 99%) yellow liquid.

**$^{1}$H NMR (400 MHz, $CDCl_3$)** $\delta$ = 8.71 (d, *J* = 4.8 Hz, 1H), 8.01 (d, *J* = 7.6 Hz, 2H), 7.72 (s, labeled, 1H, >99% D), 7.50 – 7.46 (m, 2H), 7.44 – 7.40 (m, 1H), 7.21 (d, *J* = 4.8 Hz, 1H).

**$^{13}$C NMR (100 MHz, $CDCl_3$)** $\delta$ = 157.3, 149.6, 139.3, 136.6 – 136.1 (m, labeled, 1C), 128.8, 128.6, 126.8, (122.0) 121.9 (1C), (120.4) 120.3 (1C).

note: The small peaks in parentheses are due to the splitting effect of the deuterium atom.

The analytical data corresponds with those reported in the literature.[4]

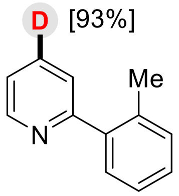


***2-(o-Tolyl)pyridine-4-d (2)***

The compounds were prepared following a general procedure and purified on silica gel (petroleum ethers ethyl acetate = 30:1) by column chromatography to obtain (50.5 mg, 99%) yellow liquid.

**$^{1}$H NMR (400 MHz, $CDCl_3$)** $\delta$ = 8.73 (d, *J* = 4.8 Hz, 1H), 7.77 – 7.73 (m, labeled, 0.07H, 93% D), 7.45 – 7.42 (m, 2H), 7.36 – 7.28 (m, 3H), 7.25 (d, *J* = 4.8 Hz, 1H), 2.40 (s, 3H).

**$^{13}$C NMR (100 MHz, $CDCl_3$)** $\delta$ = 159.9, 149.1, 140.3, 136.0 – 135.4 (m, labeled 1C), 135.6, 130.6, 129.5, 128.1, 125.7, (123.9) 123.8 (1C), (121.5) 121.4 (1C), 20.2.

**HRMS (ESI, m/z)**: calculated for $C_{12}H_{11}ND$ $[M+H]^+$: 171.1027, found: 171.1032.

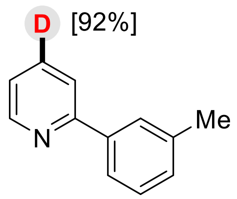


***2-(m-Tolyl)pyridine-4-d (3)***

The compounds were prepared following a general procedure and purified on silica gel (petroleum ethers ethyl acetate = 30:1) by column chromatography to obtain (50.4 mg, 99%) yellow liquid.

**$^1$H NMR (400 MHz, CDCl$_3$)** δ = 8.70 – 8.69 (m, 1H), 7.87 – 7.86 (m, 1H), 7.78 – 7.76 (m, 1H), 7.69 – 7.67 (m, labeled, 1.08H, 92% D), 7.38 – 7.34 (m, 1H), 7.23 (d, *J* = 7.6 Hz, 1H), 7.19 – 7.16 (m, 1H), 2.44 (s, 3H).

**$^{13}$C NMR (100 MHz, CDCl$_3$)** δ = 157.4, 149.4, 139.2, 138.2, 136.5 – 135.9 (m, labeled, 1C), 129.5, 128.4, 127.4, 123.8, (121.8) 121.7 (1C), (120.4) 120.3 (1C), 21.3.

**HRMS (ESI, m/z)**: calculated for $C_{12}H_{11}ND$ [M+H]$^+$: 171.1027, found: 171.1035.

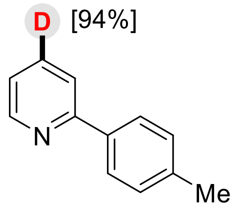


***2-(p-Tolyl)pyridine-4-d (4)***

The compounds were prepared following a general procedure and purified on silica gel (petroleum ethers ethyl acetate = 30:1) by column chromatography to obtain (50.5 mg, 99%) light yellow liquid.

**$^1$H NMR (400 MHz, CDCl$_3$)** δ = 8.53 (d, *J* = 4.8 Hz, 1H), 7.77 (d, *J* = 8.0 Hz, 2H), 7.52 (s, labeled, 1.06H, 94% D), 7.13 (d, *J* = 7.6 Hz, 2H), 7.01 (d, *J* = 4.8 Hz, 1H), 2.25 (s, 3H).

**$^{13}$C NMR (100 MHz, CDCl$_3$)** δ = 157.2, 149.4, 138.7, 136.5 – 135.9 (m, labeled, 1C), 129.3, 126.6, (121.6) 121.5 (C), (120.0) 119.9 (1C), 21.1.

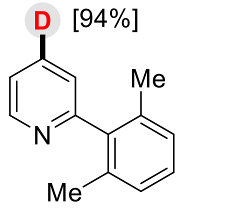


***2-(2,6-Dimethylphenyl)pyridine-4-d (5)***

The compounds were prepared following a general procedure and purified on silica gel (petroleum ethers ethyl acetate = 30:1) by column chromatography to obtain (54.7 mg, 99%) white solid.

**$^{1}$H NMR (400 MHz, CDCl$_3$)** δ = 8.76 – 8.74 (m, 1H), 8.00 – 7.75 (m, labeled, 0.06H, 94% D), 7.29 – 7.25 (m, 2H), 7.24 – 7.20 (m, 1H), 7.13 (d, *J* = 7.6 Hz, 2H), 2.07 (s, 6H).

**$^{13}$C NMR (100 MHz, CDCl$_3$)** δ = 159.8, 149.6, 140.4, 136.2 – 135.9 (m, labeled, 1C), 135.7, 127.8, 127.4, (124.3) 124.2 (1C), (121.5) 121.4 (1C), 20.1.

**HRMS (ESI, m/z)**: calculated for $C_{13}H_{13}ND$ $[M+H]^+$: 185.1184, found: 185.1190.

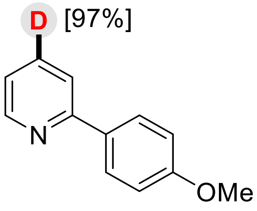


***2-(4-Methoxyphenyl)pyridine-4-d (6)***

The compounds were prepared following a general procedure and purified on silica gel (petroleum ethers ethyl acetate = 30:1 to 10:1) by column chromatography to obtain (55.3 mg, 99%) white solid.

**$^{1}$H NMR (400 MHz, CDCl$_3$)** δ = 8.64 (d, *J* = 4.8 Hz, 1H), 7.95 (d, *J* = 8.4 Hz, 2H), 7.67 – 7.63 (m, labeled, 1.03H, 97% D), 7.13 (d, *J* = 4.8 Hz, 1H), 6.98 (d, *J* = 8.0 Hz, 2H), 3.82 (s, 3H).

**$^{13}$C NMR (100 MHz, CDCl$_3$)** δ = 160.3, 156.9, 149.4, 136.5 – 136.0 (m, labeled, 1C), 131.8, 128.0, (121.2) 121.1 (1C), (119.6) 119.5 (1C), 114.0, 55.2.

**HRMS (ESI, m/z)**: calculated for $C_{12}H_{11}NOD$ $[M+H]^+$: 187.0976, found: 187.0980.

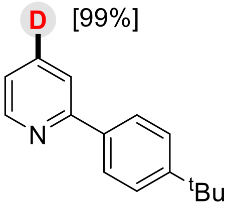


***2-(4-(Tert-Butyl)phenyl)pyridine-4-d (7)***

The compounds were prepared following a general procedure and purified on silica gel (petroleum ethers ethyl acetate = 30:1) by column chromatography to obtain (62.9 mg, 99%) white solid.

**$^{1}$H NMR (400 MHz, CDCl$_3$)** δ = 8.70 (d, *J* = 4.8 Hz, 1H), 7.96 (d, *J* = 8.0 Hz, 2H), 7.71 (s, labeled, 1.01H, 99% D), 7.52 (d, *J* = 8.0 Hz, 2H), 7.19 (d, *J* = 4.8 Hz, 1H), 1.38 (s, 9H).

**$^{13}$C NMR (100 MHz, $CDCl_3$)** δ = 157.3, 152.0, 149.5, 136.5 – 136.0 (m, labeled, 1C), 136.4, 126.5, 125.6, (121.7) 121.6 (1C), (120.2) 120.1 (1C), 34.6, 31.2.

**HRMS (ESI, m/z)**: calculated for $C_{15}H_{17}ND$ $[M+H]^+$: 213.1497, found: 213.1490.

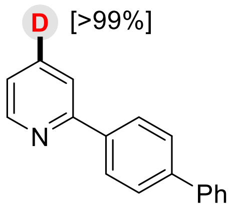


***2-([1,1'-Biphenyl]-4-yl)pyridine-4-d (8)***

The compounds were prepared following a general procedure and purified on silica gel (petroleum ethers ethyl acetate = 30:1) by column chromatography to obtain (68.9 mg, 99%) white solid.

**$^1$H NMR (400 MHz, $CDCl_3$)** δ = 8.73 (d, *J* = 4.8 Hz, 1H), 8.11 – 8.09 (m, 2H), 7.78 (s, labeled, 1H, >99% D), 7.74 – 7.72 (m, 2H), 7.69 – 7.67 (m, 2H), 7.50 – 7.46 (m, 2H), 7.40 – 7.36 (m, 1H), 7.26 – 7.23 (m, 1H).

**$^{13}$C NMR (100 MHz, $CDCl_3$)** δ = 156.9, 149.7, 141.6, 140.5, 138.2, 136.7 – 136.2 (m, labeled, 1C), 128.8, 127.5, 127.4, 127.2, 127.0, 121.9, 120.3.

**HRMS (ESI, m/z)**: calculated for $C_{17}H_{13}ND$ $[M+H]^+$: 233.1184, found: 233.1189.

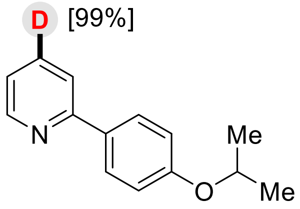


***2-(4-Isopropoxyphenyl)pyridine-4-d (9)***

The compounds were prepared following a general procedure and purified on silica gel (petroleum ethers ethyl acetate = 30:1 to 15:1) by column chromatography to obtain (63.5 mg, 99%) light yellow solid.

**$^1$H NMR (400 MHz, DMSO)** δ = 8.65 – 8.63 (m, 1H), 7.95 – 7.91 (m, 2H), 7.66 – 7.64 (m, 1.01H, 99%D), 7.14 – 7.13 (m, 1H), 6.99 – 6.95 (m, 2H), 4.64 – 4.57 (m, 1H), 1.35 (d, *J* = 6.0 Hz, 6H).

**$^{13}$C NMR (100 MHz, DMSO)** δ = 158.7, 157.0, 149.4, 136.5 – 136.0 (m, labeled, 1C), 131.6, 128.0, (121.2) 121.1 (1C), (119.6) 119.5 (1C), 115.8, 69.7, 21.9

**HRMS (ESI, m/z)**: calculated for $C_{11}H_8NOD_2$ $[M+H]^+$: 215.1289, found: 215.1280.

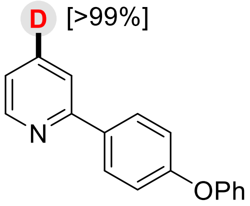


***2-(4-Phenoxyphenyl)pyridine-4-d (10)***

The compounds were prepared following a general procedure and purified on silica gel (petroleum ethers ethyl acetate = 30:1 to 15:1) by column chromatography to obtain (73.7 mg, 99%) white solid.

**$^{1}$H NMR (400 MHz, CDCl$_3$)** δ = 8.68 (d, *J* = 4.8 Hz, 1H), 7.99 (d, *J* = 8.4 Hz, 2H), 7.72 – 7.68 (d, *J* = 14.4 Hz, labeled, 1H, >99% D), 7.39 – 7.35 (m, 2H), 7.19 (d, *J* = 4.8 Hz, 1H), 7.16 – 7.07 (m, 5H).

**$^{13}$C NMR (100 MHz, CDCl$_3$)** δ = 158.1, 156.8, 156.7, 149.5, 136.6 – 136.1 (m, labeled, 1C), 134.3, 129.7, 128.3, 123.4, 121.6, 119.9, 119.1, 118.7.

**HRMS (ESI, m/z)**: calculated for $C_{17}H_{13}NOD$ $[M+H]^+$: 249.1133, found: 249.1133.

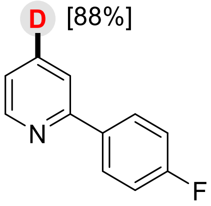


***2-(4-Fluorophenyl)pyridine-4-d (11)***

The compounds were prepared following a general procedure and purified on silica gel (petroleum ethers ethyl acetate = 30:1) by column chromatography to obtain (50.7 mg, 97%) light yellow solid.

**$^{1}$H NMR (400 MHz, CDCl$_3$)** δ = 8.66 (d, *J* = 4.8 Hz, 1H), 7.98 – 7.95 (m, 2H), 7.72 – 7.64 (m, labeled, 1.12H, 88% D), 7.19 (d, *J* = 4.8 Hz, 1H), 7.16 – 7.10 (m, 2H).

**$^{13}$C NMR (100 MHz, CDCl$_3$)** δ = 163.4 (d, $J_{C\text{-}F}$ = 247.0 Hz), 156.3, 149.5, 136.7 – 136.1 (m, labeled, 1C), 135.4 (d, $J_{C\text{-}F}$ = 3.0 Hz), 128.6 (d, $J_{C\text{-}F}$ = 8.0 Hz), (121.9) 121.8 (1C), (120.1) 120.0 (1C), 115.5 (d, $J_{C\text{-}F}$ = 21.0 Hz).

**$^{19}$F NMR (376 MHz, CDCl$_3$)** δ = -113.10.

**HRMS (ESI, m/z)**: calculated for $C_{11}H_8NFD$ $[M+H]^+$: 175.0777, found: 175.0783.

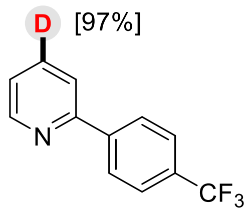

***2-(4-(Trifluoromethyl)phenyl)pyridine-4-d (12)***

The compounds were prepared following a general procedure and purified on silica gel (petroleum ethers ethyl acetate = 30:1 to 20:1) by column chromatography to obtain (66.5 mg, 99%) yellow solid.

**$^1$H NMR (400 MHz, $CDCl_3$)** δ = 8.69 (d, *J* = 4.8 Hz, 1H), 7.92 – 7.90 (m, 2H), 7.72 – 7.70 (m, labeled, 1.03H, 97% D), 7.30 – 7.28 (m, 2H), 7.20 – 7.17 (m, 1H).

**$^{13}$C NMR (100 MHz, $CDCl_3$)** δ = 157.3, 149.5, 138.7, 136.6 – 136.0 (m, labeled, 1C), 136.5, 129.4, 126.7, (121.7) 121.6 (1C), (120.1) 120.0 (1C), 20.7 – 20.0 (m, 1C, $-CF_3$).

**$^{19}$F NMR (376 MHz, $CDCl_3$)** δ = -62.5.

**HRMS (ESI, m/z)**: calculated for $C_{12}H_8NF_3D$ $[M+H]^+$: 225.0745, found: 225.0749.

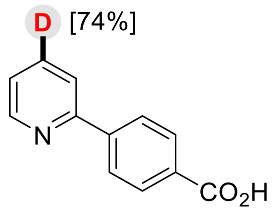

***4-(Pyridin-2-yl-4-d)benzoic acid (13)***

The compounds were prepared following a general procedure and purified on silica gel (petroleum ethers ethyl acetate = 10:1 to 3:1) by column chromatography to obtain (54.0 mg, 90%) light yellow solid.

**$^1$H NMR (400 MHz, DMSO-$d_6$)** δ = 13.04 (s, 1H), 8.72 (d, *J* = 4.8 Hz, 1H), 8.21 (d, *J* = 8.4 Hz, 2H), 8.05 (d, *J* = 8.0 Hz, 3H), 7.96 – 7.92 (m, labeled, 0.26H, 74% D), 7.45-7.41 (m, 1H).

**$^{13}$C NMR (100 MHz, DMSO-$d_6$)** δ = 167.5, 155.3, 150.2, 143.0, 138.0 (m, labeled, 1C), 131.5, 130.2, 127.1, (123.9) 123.8 (1C), (121.4) 121.3 (1C).

**HRMS (ESI, m/z)**: calculated for $C_{12}H_7NO_2^-D$ $[M-H]^+$: 199.0623, found: 199.0631.

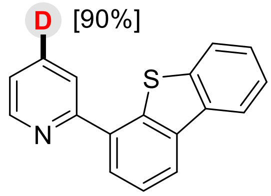

***2-(Dibenzo[b,d]thiophen-4-yl)pyridine-4-d (14)***

The compounds were prepared following a general procedure and purified on silica gel (petroleum ethers ethyl acetate = 30:1) by column chromatography to obtain (69.2 mg, 88%) light yellow solid.

**$^{1}$H NMR (400 MHz, CDCl$_3$)** δ = 8.92 (d, *J* = 4.8 Hz, 1H), 8.28 – 8.22 (m, 2H), 8.02 – 7.96 (m, 3H), 7.82 – 7.78 (m, labeled, 0.1H, 90% D), 7.61 – 7.57 (m, 1H), 7.55 – 7.48 (m, 2H), 7.30 (d, *J* = 4.8 Hz, 1H).

**$^{13}$C NMR (100 MHz, CDCl$_3$)** δ = 156.0, 148.3, 141.9, 137.5, 137.1, 136.5 – 136.0 (m, labeled, 1C), 134.6, 133.2, 126.6, 124.9, 124.4, 124.0, 122.3, 122.0, 121.9, 121.2, (120.7) 120.6 (1C).

**HRMS (ESI, m/z)**: calculated for $C_{17}H_{11}NSD$ $[M+H]^+$: 263.0748, found: 263.0740.

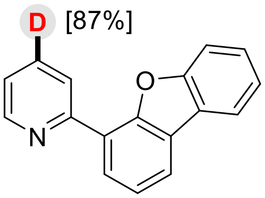


***2-(Dibenzo[b,d]furan-4-yl)pyridine-4-d (15)***

The compounds were prepared following a general procedure and purified on silica gel (petroleum ethers ethyl acetate = 30:1) by column chromatography to obtain (67.8 mg, 92%) white solid.

**$^{1}$H NMR (400 MHz, CDCl$_3$)** δ = 8.81 (d, *J* = 4.4 Hz, 1H), 8.41 (s, 1H), 8.31 (d, *J* = 7.6 Hz, 1H), 8.00 – 7.96 (m, 2H), 7.87 – 7.83 (m, labeled, 0.13H, 87% D), 7.64 (d, *J* = 8.2 Hz, 1H), 7.51 – 7.46 (m, 2H), 7.38 – 7.35 (m, 1H), 7.28 (d, *J* = 4.8 Hz, 1H).

**$^{13}$C NMR (100 MHz, CDCl$_3$)** δ = 156.1, 153.7, 153.7, 149.8, 136.6 – 136.1 (m, labeled, 1C), 127.29, 127.26, 125.2, (125.1) (1C), 124.3 124.2 (1C), 124.0, 123.3 (123.2) (1C), 123.0, (122.5) 122.4 (1C), 121.3, 120.7, 111.8.

**HRMS (ESI, m/z)**: calculated for $C_{17}H_{11}NOD$ $[M+H]^+$: 247.0976, found: 247.0982.

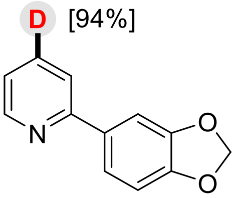


***2-(Benzo[d][1,3]dioxol-5-yl)pyridine-4-d (16)***

The compounds were prepared following a general procedure and purified on silica gel (petroleum ethers ethyl acetate = 30:1 to 10:1) by column chromatography to obtain (59.4 mg, 99%) white solid.

**$^1$H NMR (400 MHz, CDCl$_3$)** δ = 8.61 – 8.59 (m, 1H), 7.62 – 7.60 (m, labeled, 0.06H, 94%D), 7.56 (s, 1H), 7.51 (d, *J* = 1.6 Hz, 1H), 7.48 – 7.45 (m, 1H), 7.11 – 7.10 (m, 1H), 6.86 (d, *J* = 8.4 Hz, 1H), 5.94 (s, 2H).

**$^{13}$C NMR (100 MHz, CDCl$_3$)** δ = 156.6, 149.3, 148.2, 148.1, 136.4 – 135.9 (m, labeled, 1C), 133.6, (121.4) 121.3 (1C), 120.6, (119.7) 119.6 (1C), 108.2, 107.1, 101.1.

**HRMS (ESI, m/z)**: calculated for $C_{12}H_9NO_2D$ [M+H]$^+$: 201.0769, found: 201.0775.

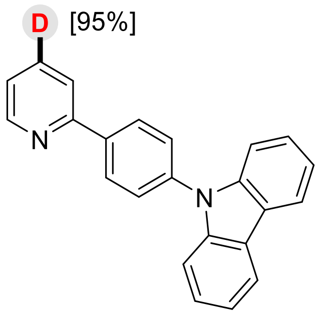


***9-(4-(Pyridin-2-yl-4-d)phenyl)-9H-carbazole (17)***

The compounds were prepared following a general procedure and purified on silica gel (petroleum ethers ethyl acetate = 30:1 to 20:1) by column chromatography to obtain (95.3 mg, 99%) yellow solid.

**$^1$H NMR (400 MHz, CDCl$_3$)** δ = 8.78 (d, *J* = 4.8 Hz, 1H), 8.26 – 8.23 (m, 2H), 8.19 (d, *J* = 7.6 Hz, 2H), 7.82 – 7.80 (m, labeled, 1.05H, 95% D), 7.72 – 7.69 (m, 2H), 7.52 (d, *J* = 8.0 Hz, 2H), 7.47 – 7.43 (m, 2H), 7.35 – 7.28 (m, 3H).

**$^{13}$C NMR (100 MHz, CDCl$_3$)** δ = 156.4, 149.8, 140.6, 138.3, 136.9 – 136.3 (m, labeled, 1C), 128.3, 127.1, 126.0, 123.4, (122.3) 122.2 (1C), (120.5) 120.4 (1C), 120.3, 120.0, 109.8.

**HRMS (ESI, m/z)**: calculated for $C_{23}H_{16}N_2D$ [M+H]$^+$: 322.1449, found: 322.1454.

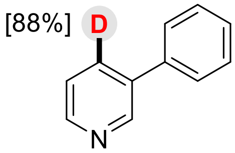


***3-Phenylpyridine-4-d (18)***

The compounds were prepared following a general procedure and purified on silica gel (petroleum ethers ethyl acetate = 30:1) by column chromatography to obtain (46.3 mg, 99%) yellow liquid.

**$^{1}$H NMR (400 MHz, CDCl$_3$)** δ = 8.83 (m, 1H), 8.57 (d, *J* = 4.8 Hz, 1H), 7.84 – 7.81 (m, labeled, 0.12H, 88% D), 7.56 – 7.53 (m, 2H), 7.46 – 7.42 (m, 2H), 7.39 – 7.35 (m, 1H), 7.33 – 7.29 (m, 1H).

**$^{13}$C NMR (100 MHz, CDCl$_3$)** δ = 148.2, 148.1, 137.5, (136.4) 136.3 (1C), 134.1 – 133.6 (m, labeled, 1C), 128.9, 127.9, 126.9, (123.4) 123.2 (1C).

The analytical data corresponds with those reported in the literature.[4]

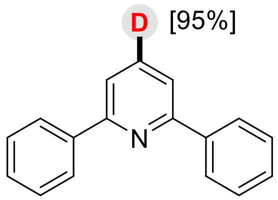


***2,6-Diphenylpyridine-4-d (19)***

The compounds were prepared following a general procedure and purified on silica gel (petroleum ethers ethyl acetate = 30:1) by column chromatography to obtain (68.9 mg, 99%) light yellow solid.

**$^{1}$H NMR (400 MHz, CDCl$_3$)** δ = 8.26 – 8.24 (m, 4H), 7.85 – 7.83 (m, labeled, 0.05H, 95% D), 7.75 (d, *J* = 3.6 Hz, 2H), 7.61 – 7.57 (m, 4H), 7.53 – 7.49 (m, 2H).

**$^{13}$C NMR (100 MHz, CDCl$_3$)** δ = 156.8, 139.5, 137.5 – 137.0 (m, labeled, 1C), 129.1, 128.8, 127.1, (118.7) 118.6 (1C).

**HRMS (ESI, m/z)**: calculated for $C_{17}H_{13}ND$ $[M+H]^+$: 233.1184, found: 233.1190.

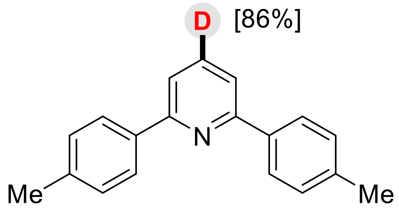


***2,6-Di-p-tolylpyridine-4-d (20)***

The compounds were prepared following a general procedure and purified on silica gel (petroleum ethers ethyl acetate = 30:1) by column chromatography to obtain (77.3 mg, 99%) light yellow solid.

**$^{1}$H NMR (400 MHz, $CDCl_3$)** δ = 8.07 – 8.05 (m, 4H), 7.79 – 7.76 (m, labeled, 0.14H, 86% D), 7.65 – 7.63 (m, 1.94H, 6% D), 7.31 (d, *J* = 8.0 Hz, 4H), 2.43 (s, 6H).

**$^{13}$C NMR (100 MHz, $CDCl_3$)** δ = 156.7, 138.9, 136.7, 129.4, 126.8, (118.0) 117.9 (1C), 21.3.

**HRMS (ESI, m/z)**: calculated for $C_{19}H_{17}ND$ $[M+H]^+$: 261.1497, found: 261.1490.

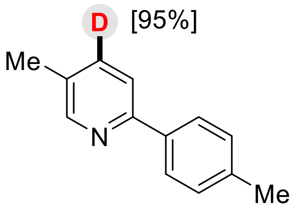


***5-Methyl-2-(p-tolyl)pyridine-4-d (21)***

The compounds were prepared following a general procedure and purified on silica gel (petroleum ethers ethyl acetate = 30:1) by column chromatography to obtain (54.7 mg, 99%) white solid.

**$^{1}$H NMR (400 MHz, $CDCl_3$)** δ = 8.53 (s, 1H), 7.91 – 7.89 (m, 2H), 7.61 (s, 1H), 7.55 – 7.52 (m, labeled, 0.05H, 95% D), 7.29 (d, *J* = 8.0 Hz, 2H), 2.42 (s, 3H), 2.37 (s, 3H).

**$^{13}$C NMR (100 MHz, $CDCl_3$)** δ = 154.7, 149.8 (d, $J_{C–D}$ = 5.0 Hz, 1C), 138.4, 137.1 – 136.6 (m, labeled, 1C), 136.5, 131.0, 129.3, 126.4 (d, $J_{C–D}$ = 8.0 Hz, 1C), 119.5 (d, $J_{C–D}$ = 8.0 Hz, 1C), 21.14 – 21.1 (d, $J_{C–D}$ = 2.0 Hz, 1C), 18.0.

**HRMS (ESI, m/z)**: calculated for $C_{13}H_{13}ND$ $[M+H]^+$: 185.1184, found: 185.1189.

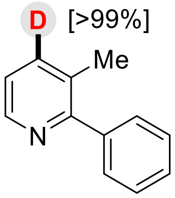


***3-Methyl-2-phenylpyridine-4-d (22)***

The compounds were prepared following a general procedure and purified on silica gel (petroleum ethers ethyl acetate = 30:1) by column chromatography to obtain (50.9 mg, 99%) white solid.

**$^{1}$H NMR (400 MHz, $CDCl_3$)** δ = 8.52 (d, *J* = 4.8 Hz, 1H), 7.53 – 7.50 (m, 2H), 7.46 – 7.42 (m, 2H), 7.40 – 7.35 (m, 1H), 7.17 – 7.14 (m, 1H), 2.34 (s, 3H).

**$^{13}$C NMR (100 MHz, $CDCl_3$)** δ = 158.5, 146.82 (146.76) (1C), 140.5, 138.4 – 137.8 (m, labeled, 1C), 130.6, 128.8, 128.0, 127.8, (121.9) 121.8 (1C), 19.9.

**HRMS (ESI, m/z)**: calculated for $C_{12}H_{11}ND$ $[M+H]^+$: 171.1027, found: 171.1025.

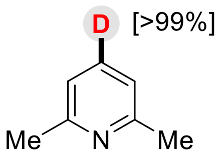


***2,6-Dimethylpyridine-4-d (23)***

The compounds were prepared following a general procedure and purified on silica gel (petroleum ethers ethyl acetate = 30:1) by column chromatography to obtain (30.8 mg, 95%) yellow liquid.

**$^1$H NMR (400 MHz, $CDCl_3$)** δ = 7.17 (s, 2H), 2.60 (s, 6H).

**$^{13}$C NMR (100 MHz, $CDCl_3$)** δ = 158.5, 146.8 (labeled, 1C) 118.2, 24.5.

**HRMS (ESI, m/z)**: calculated for $C_7H_9ND$ $[M+H]^+$: 109.0871, found: 109.0877.

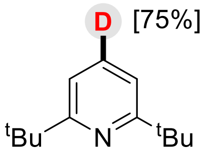


***2,6-Di-tert-butylpyridine-4-d (24)***

The compounds were prepared following a general procedure and purified on silica gel (petroleum ethers ethyl acetate = 30:1) by column chromatography to obtain (54.7 mg, 95%) yellow liquid.

**$^1$H NMR (400 MHz, $CDCl_3$)** δ = 7.53 – 7.49 (m, labeled, 0.25H, 75% D), 7.10 – 7.08 (m, labeled, 2H), 1.36 (s, 18H).

**$^{13}$C NMR (100 MHz, $CDCl_3$)** δ = 167.5, 135.9 – 135.4 (m, labeled, 1C), (115.2) 115.1 (1C), 37.6, 30.1.

**HRMS (ESI, m/z)**: calculated for $C_{13}H_{21}ND$ $[M+H]^+$: 193.1810, found: 193.1821.

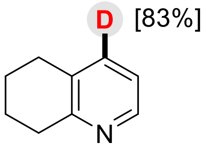


***5,6,7,8-Tetrahydroquinoline-4-d (25)***

The compounds were prepared following a general procedure and purified on silica gel (petroleum ethers ethyl acetate = 30:1) by column chromatography to obtain (39.8 mg, 99%) yellow liquid.

**$^{1}$H NMR (400 MHz, CDCl$_3$)** δ = 8.31 (d, *J* = 4.8 Hz, 1H), 7.31 – 7.29 (m, labeled, 0.17H, 83%), 6.99 – 6.96 (m, 1H), 2.89 (t, *J* = 6.4 Hz, 2H), 2.73 (t, *J* = 6.4 Hz, 2H), 1.89 – 1.83 (m, 2H), 1.81 – 1.74 (m, 2H).

**$^{13}$C NMR (100 MHz, CDCl$_3$)** δ = 157.2, 146.6, 136.6 – 136.1 (m, labeled, 1C), 132.1, (120.8) 120.6 (1C), 32.4, 28.6, 23.0, 22.6.

The analytical data corresponds with those reported in the literature.[4]

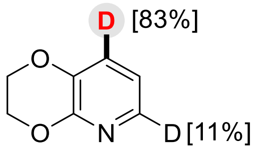


***2,3-Dihydro-[1,4]dioxino[2,3-b]pyridine-6,8-d$_2$ (26)***

The compounds were prepared following a general procedure and purified on silica gel (petroleum ethers ethyl acetate = 30:1 to 10:1) by column chromatography to obtain (40.4 mg, 97%) yellow liquid.

**$^{1}$H NMR (400 MHz, CDCl$_3$)** δ = 7.74 (d, *J* = 5.2 Hz, labeled, 0.89H, 11% D), 7.09 (d, *J* = 8.0 Hz, labeled, 0.17H, 83% D), 6.78 (d, *J* = 4.4 Hz, 1H), 4.37 – 4.35 (m, 2H), 4.18 – 4.16 (m, 2H).

**$^{13}$C NMR (100 MHz, CDCl$_3$)** δ = 150.8, 139.6, (139.2) 139.1 (1C), 124.5 – 124.0 (m, labeled, 1C), 118.2 – 118.0 (m, 1C), 64.7, 63.7.

**HRMS (ESI, m/z)**: calculated for $C_7H_6NO_2D_2$ $[M+H]^+$: 140.0675, found: 140.0681.

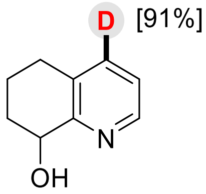


***5,6,7,8-Tetrahydroquinolin-4-d-8-ol (27)***

The compounds were prepared following a general procedure and purified on silica gel (petroleum ethers ethyl acetate = 30:1 to 5:1) by column chromatography to obtain (42.7 mg, 95%) white solid.

**$^{1}$H NMR (400 MHz, CDCl$_3$)** δ = 8.39 (d, *J* = 4.8 Hz, 1H), 7.42 – 7.39 (m, labeled, 0.09H, 91% D), 7.11 (d, *J* = 4.4 Hz, 1H), 4.74 – 4.70 (m, 1H), 4.25 (s, 1H), 2.87 – 2.72 (m, 2H), 2.30 – 2.21 (m, 1H), 2.04 – 1.95 (m, 1H), 1.87 – 1.74 (m, 2H).

**$^{13}$C NMR (100 MHz CDCl$_3$,)** δ = 158.0, 146.5, 137.0 – 136.4 (m, labeled, 1C), (131.7) 131.6 (1C), (122.3) 122.2 (1C), 68.5, 30.7, (28.32) 28.26 (1C), 19.2.

**HRMS (ESI, m/z)**: calculated for $C_9H_{11}NOD$ $[M+H]^+$: 151.0976, found: 151.0980.

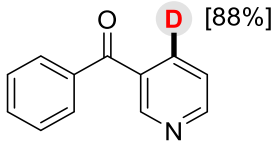


***Phenyl(pyridin-3-yl-4-d)methanone (28)***

The compounds were prepared following a general procedure and purified on silica gel (petroleum ethers ethyl acetate = 30:1 to 15:1) by column chromatography to obtain (50.8 mg, 92%) light yellow solid.

**$^1$H NMR (400 MHz, CDCl$_3$)** δ = 8.51 (s, 1H), 8.46 (d, *J* = 4.8 Hz, 1H), 7.48 – 7.45 (m, labeled, 0.12H, 88% D), 7.33 – 7.28 (m, 2H), 7.25 – 7.22 (m, 1H), 7.21 – 7.16 (m, 3H).

**$^{13}$C NMR (100 MHz, CDCl$_3$)** δ = 150.1, 147.6, 139.7, 136.3 (m, labeled, 1C), 128.8, 128.7, 126.5, 123.3.

**HRMS (ESI, m/z)**: calculated for $C_{12}H_9NOD$ $[M+H]^+$: 185.0820, found: 185.0827.

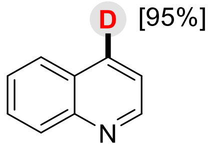


***Quinoline-4-d (29)***

The compounds were prepared following a general procedure and purified on silica gel (petroleum ethers ethyl acetate = 30:1) by column chromatography to obtain (38.7 mg, 99%) yellow liquid.

**$^1$H NMR (400 MHz, CDCl$_3$)** δ = 8.93 (d, *J* = 4.4 Hz, 1H), 8.17 – 8.12 (m, labeled, 1.07H, 93% D), 7.82 (d, *J* = 8.0 Hz, 1H), 7.74 – 7.71 (m, 1H), 7.57 – 7.53 (m, 1H), 7.40 (d, *J* = 4.2 Hz, 1H).

**$^{13}$C NMR (100 MHz, CDCl$_3$)** δ = 150.3, 148.1, 136.1 – 135.5 (m, labeled, 1C), 129.4, 129.3, 128.1, (127.73) 127.68 (1C), 126.5, 120.9.

The analytical data corresponds with those reported in the literature.[5]

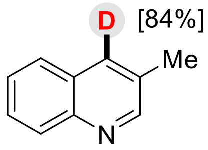

***3-Methylquinoline-4-d (30)***

The compounds were prepared following a general procedure and purified on silica gel (petroleum ethers ethyl acetate = 30:1) by column chromatography to obtain (42.8 mg, 99%) light yellow liquid.

**$^1$H NMR (400 MHz, CDCl$_3$)** δ = 8.73 (s, 1H), 8.06 – 8.04 (m, 1H), 7.84 – 7.83 (m, labeled, 0.16H, 84% D), 7.70 – 7.67 (m, 1H), 7.62 – 7.58 (m, 1H), 7.48 – 7.44 (m, 1H), 2.45 (s, 3H).

**$^{13}$C NMR (100 MHz, CDCl$_3$)** δ = 152.2, 146.4, 134.5 – 134.0 (m, labeled, 1C), (130.3) 130.2 (1C), 129.0, 128.3, (128.0) 127.9 (1C), (127.0) 126.9 (1C), 126.4, (18.6) 18.5 (1C).

**HRMS (ESI, m/z)**: calculated for $C_{10}H_9ND$ $[M+H]^+$: 145.0871, found: 145.0878.

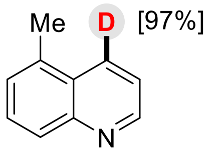


***5-Methylquinoline-4-d (31)***

The compounds were prepared following a general procedure and purified on silica gel (petroleum ethers ethyl acetate = 30:1) by column chromatography to obtain (42.7 mg, 99%) yellow liquid.

**$^1$H NMR (400 MHz, CDCl$_3$)** δ = 8.92 (d, $J$ = 4.0 Hz, 1H), 8.34 – 8.31 (m, labeled, 0.03H, 97% D), 7.97 (d, $J$ = 8.4 Hz, 1H), 7.62 – 7.58 (m, 1H), 7.43 – 7.36 (m, 2H), 2.69 (s, 3H).

**$^{13}$C NMR (100 MHz, CDCl$_3$)** δ = 149.9, 148.5, 134.5, 132.4 – 131.9 (m, labeled, 1C), 129.1, 127.7, 127.5, 127.0, 120.5, 18.6.

**HRMS (ESI, m/z)**: calculated for $C_{10}H_9ND$ $[M+H]^+$: 145.0871, found: 145.0875.

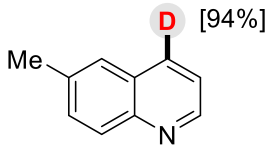


***6-Methylquinoline-4-d (32)***

The compounds were prepared following a general procedure and purified on silica gel (petroleum ethers ethyl acetate = 30:1) by column chromatography to obtain (42.8 mg, 99%) yellow liquid.

**$^1$H NMR (400 MHz, CDCl$_3$)** δ = 8.81 (d, $J$ = 4.2 Hz, 1H), 8.00 – 7.97 (m, labeled, 1.06H, 94% D), 7.51 – 7.49 (m, 2H), 7.29 (d, $J$ = 4.2 Hz, 1H), 2.49 (s, 3H).

**$^{13}$C NMR (100 MHz, $CDCl_3$)** δ = 149.4, 146.7, 136.2, 135.1 – 134.6 (m, labeled, 1C), 131.6, 129.0, 128.1, 126.4, 120.8, 21.4.

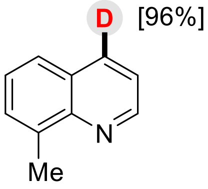


***8-Methylquinoline-4-d (33)***

The compounds were prepared following a general procedure and purified on silica gel (petroleum ethers ethyl acetate = 30:1) by column chromatography to obtain (42.7 mg, 99%) colorless liquid.

**$^{1}$H NMR (400 MHz, $CDCl_3$)** δ = 8.95 (d, *J* = 4.2 Hz, 1H), 8.12 – 8.10 (m, labeled, 0.04H, 96% D), 7.65 (d, *J* = 8.0 Hz, 1H), 7.56 (d, *J* = 6.8 Hz, 1H), 7.44 – 7.41 (m, 1H), 7.38 (d, *J* = 4.0 Hz, 1H), 2.83 (s, 3H).

**$^{13}$C NMR (100 MHz, $CDCl_3$)** δ = 149.2, 147.3, 137.0, 136.2 – 135.7 (m, labeled, 1C), 129.6, 128.1, 126.2, (125.81) 125.76 (1C), (120.8) 120.6 (1C), 18.1.

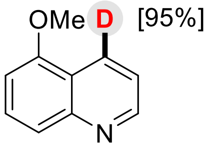


***5-Methoxyquinoline-4-d (34)***

The compounds were prepared following a general procedure and purified on silica gel (petroleum ethers ethyl acetate = 30:1 to 15:1) by column chromatography to obtain (47.6 mg, 99%) yellow liquid.

**$^{1}$H NMR (400 MHz, $CDCl_3$)** δ = 8.90 (d, *J* = 4.2 Hz, 1H), 8.58 (d, *J* = 8.4 Hz, labeled, 0.05H, 95% D), 7.70 (d, *J* = 8.4 Hz, 1H), 7.61- 7.59 (m, 1H), 7.37 (d, *J* = 4.2 Hz, 1H), 6.86 (d, *J* = 7.6 Hz, 1H), 4.00 (s, 3H).

**$^{13}$C NMR (100 MHz, $CDCl_3$)** δ = 155.1, 150.6, 148.9, 129.4, 121.4, 120.7, 120.0, 104.2, 55.7.

**HRMS (ESI, m/z)**: calculated for $C_{10}H_9NOD$ $[M+H]^+$: 161.0820, found: 161.0825.

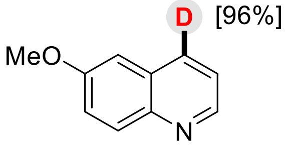


***6-Methoxyquinoline-4-d (35)***

The compounds were prepared following a general procedure and purified on silica gel (petroleum ethers ethyl acetate = 30:1 to 15:1) by column chromatography to obtain (47.5 mg, 99%) yellow liquid.

**$^1$H NMR (400 MHz, CDCl$_3$)** δ = 8.68 (d, *J* = 4.4 Hz, 1H), 7.95 – 7.91 (m, labeled, 1.04H, 96% D), 7.30 – 7.27 (m, 1H), 7.23 (d, *J* = 4.4 Hz, 1H), 6.94 (d, *J* = 2.8 Hz, 1H), 3.81 (s, 3H).

**$^{13}$C NMR (100 MHz, CDCl$_3$)** δ = 157.4, 147.6, 144.1, 134.6 – 134.0 (m, labeled, 1C), 130.5, 129.0, 122.1, 121.0, 104.8, 55.2.

**HRMS (ESI, m/z)**: calculated for $C_{10}H_9NOD$ $[M+H]^+$: 161.0820, found: 161.0831.

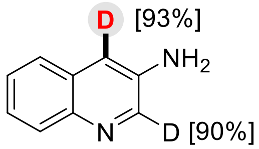


***Quinolin-2,4-d$_2$-3-amine (36)***

The compounds were prepared following a general procedure and purified on silica gel (petroleum ethers ethyl acetate = 15:1 to 2:1) by column chromatography to obtain (43.3 mg, 99%) yellow solid.

**$^1$H NMR (400 MHz, CDCl$_3$)** δ = 8.51 (s, labeled, 0.1H, 90% D), 7.99 – 7.94 (m, 1H), 7.60 – 7.56 (m, 1H), 7.46 – 7.40 (m, 2H), 7.23 (d, *J* = 0.8 Hz, labeled, 0.07H, 93% D), 3.92 (s, 2H).

**$^{13}$C NMR (100 MHz, CDCl$_3$)** δ = 143.0 (142.9) (1C), 142.7 – 142.4 (m, labeled, 1C), (139.65) 139.57 (1C), 129.0, 128.9, 126.9, (125.82) 125.77 (1C), 125.6, 115.0 – 114.5 (m, labeled, 1C).

**HRMS (ESI, m/z)**: calculated for $C_9H_7N_2D_2$ $[M+H]^+$: 147.0886, found: 147.0895.

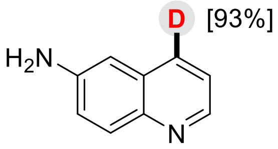


***Quinolin-4-d-6-amine (37)***

The compounds were prepared following a general procedure and purified on silica gel (petroleum ethers ethyl acetate = 15:1 to 2:1) by column chromatography to obtain (43.0 mg, 99%) light yellow solid.

**$^1$H NMR (400 MHz, CDCl$_3$)** δ = 8.66 (d, *J* = 4.4 Hz, 1H), 7.93 – 7.88 (m, labeled, 1.07H, 93% D), 7.28 – 7.25 (m, 1H), 7.17 – 7.14 (m, 1H), 6.89 (d, *J* = 2.8 Hz, 1H), 3.98 (s, 2H).

**$^{13}$C NMR (100 MHz, CDCl$_3$),** δ = 146.7, 144.6, 143.3, 133.7 – 133.1 (m, labeled, 1C), 130.4, 129.6, 121.5, (121.3) 121.2 (1C), (107.3) 107.2 (1C).

**HRMS (ESI, m/z)**: calculated for $C_9H_8N_2D$ [M+H]$^+$: 146.0823, found: 146.0829.

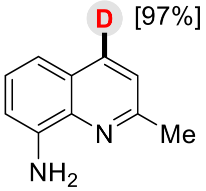


***2-Methylquinolin-4-d-8-amine (38)***

The compounds were prepared following a general procedure and purified on silica gel (petroleum ethers ethyl acetate = 30:1 to 10:1) by column chromatography to obtain (47.2 mg, 99%) light yellow solid.

**$^1$H NMR (400 MHz, CDCl$_3$)** δ = 7.98 –7.96 (m, labeled, 0.03H, 97% D), 7.32 – 7.26 (m, 2H), 7.16 – 7.14 (m, 1H), 6.93 (d, $J$ = 7.6 Hz, 1H), 5.02 (s, 2H), 2.75 (s, 3H).

**$^{13}$C NMR (100 MHz, CDCl$_3$)** δ = 156.0, 143.3, 137.7, 135.9 – 135.4 (m, labeled, 1C), 126.7, 126.2 (126.1) (1C), 121.9, 115.7, 110.0, 25.13 (25.07) (1C).

**HRMS (ESI, m/z)**: calculated for $C_{10}H_{10}N_2D$ [M+H]$^+$: 160.0980, found: 160.0989.

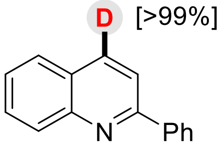


***2-Phenylquinoline-4-d (39)***

The compounds were prepared following a general procedure and purified on silica gel (petroleum ethers ethyl acetate = 30:1 to 20:1) by column chromatography to obtain (61.2 mg, 99%) white solid.

**$^1$H NMR (400 MHz, CDCl$_3$)** δ = 8.23 – 8.18 (m, labeled, 3H, >99% D), 7.88 – 7.86 (m, 1H), 7.83 – 7.81 (m, 1H), 7.77 – 7.72 (m, 1H), 7.57 – 7.47 (m, 4H).

**$^{13}$C NMR (100 MHz, CDCl$_3$)** δ = 157.2, 148.2, 139.6, 136.7 – 136.1 (m, labeled, 1C), 129.64, 129.56, 129.2, 128.8, 127.5, (127.4) 127.3 (1C), 127.0, 126.2, 118.8.

**HRMS (ESI, m/z)**: calculated for $C_{15}H_{11}ND$ [M+H]$^+$: 207.1027, found: 207.1029.

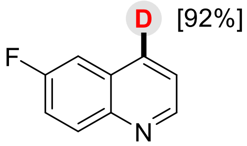


***6-Fluoroquinoline-4-d (40)***

The compounds were prepared following a general procedure and purified on silica gel (petroleum ethers ethyl acetate = 30:1) by column chromatography to obtain (41.7 mg, 94%) yellow liquid.

**$^{1}$H NMR (400 MHz, CDCl$_3$)** δ = 8.89 (d, *J* = 4.4 Hz, 1H), 8.15 – 8.12 (m, labeled, 1.08H, 92% D), 7.53 – 7.48 (m, 1H), 7.46 – 7.43 (m, 2H).

**$^{13}$C NMR (100 MHz, CDCl$_3$)** δ = 160.3 (d, $J_{C–F}$ = 247 Hz, 1C), 149.6, 145.3, 135.4 – 134.8 (m, labeled, 1C), 132.0 – 131.8 (m, 1C), 128.8 (d, $J_{C–F}$ = 10.0 Hz, 1C), 121.6 (d, $J_{C–D}$ = 6.0 Hz, 1C), 119.9 – 119.6 (m, 1C), 110.7 – 110.5 (m, 1C).

**$^{19}$F NMR (376 MHz, CDCl$_3$)** δ = -113.1.

**HRMS (ESI, m/z)**: calculated for $C_9H_6NFD$ $[M+H]^+$: 149.0620, found: 149.0625.

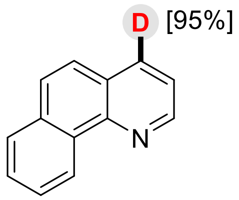


***Benzo[h]quinoline-4-d (41)***

The compounds were prepared following a general procedure and purified on silica gel (petroleum ethers ethyl acetate = 30:1) by column chromatography to obtain (53.5 mg, 99%) white solid.

**$^{1}$H NMR (400 MHz, CDCl$_3$)** δ = 9.34 (d, *J* = 8.2 Hz, 1H), 9.01 (d, *J* = 4.4 Hz, 1H), 8.14 – 8.12 (m, labeled, 0.05H, 95% D), 7.91 – 7.89 (m, 1H), 7.80 – 7.74 (m, 2H), 7.72 – 7.68 (m, 1H), 7.65 (d, *J* = 8.8 Hz, 1H), 7.49 (d, *J* = 4.4 Hz, 1H).

**$^{13}$C NMR (100 MHz, CDCl$_3$)** δ = 148.7, 146.4, 135.7 – 135.2 (m, labeled, 1C), 133.5, 131.4, 128.1, 127.74, 127.66, 127.0, 126.2, (125.24) 125.19 (1C), 124.3, (121.7) 121.6 (1C).

The analytical data corresponds with those reported in the literature.[5]

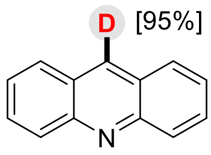


***Acridine-9-d (42)***

The compounds were prepared following a general procedure and purified on silica gel (petroleum ethers ethyl acetate = 20:1 to 15:1) by column chromatography to obtain (53.4 mg, 99%) white solid.

**$^{1}$H NMR (400 MHz, CDCl$_3$)** δ = 8.53 (s, labeled, 0.05H, 95% D), 8.19 (d, *J* = 8.8 Hz, 2H), 7.82 – 7.80 (m, 2H), 7.70 – 7.66 (m, 2H), 7.41 – 7.37 (m, 2H).

**$^{13}$C NMR (100 MHz, CDCl$_3$)** δ = 148.7, 135.8 – 135.1 (m, labeled, 1C), 130.0, 129.1, (127.95) 127.9 (1C), (126.2) 126.2 (1C), 125.4.

**HRMS (ESI, m/z)**: calculated for $C_{13}H_9ND$ $[M+H]^+$: 181.0871, found: 181.0880.

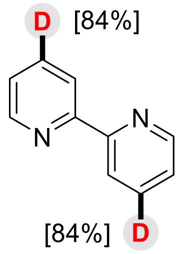


***2,2'-Bipyridine-4,4'-d$_2$ (43)***

The compounds were prepared following a general procedure and purified on silica gel (petroleum ethers ethyl acetate = 10:1 to 3:1) by column chromatography to obtain (46.9 mg, 99%) white solid.

**$^{1}$H NMR (400 MHz, CDCl$_3$)** δ = 8.67 (d, *J* = 4.8 Hz, 2H), 8.39 (d, *J* = 4.8 Hz, 2H), 7.82 – 7.78 (m, labeled, 0.32H, 84% D), 7.29 (d, *J* = 4.8 Hz, 2H).

**$^{13}$C NMR (100 MHz, CDCl$_3$)** δ = 156.0, 149.1 (149.0) (2C), 136.8 – 136.2 (m, labeled, 2C), (123.6) 123.5 (2C), (120.9) 120.8 (2C).

The analytical data corresponds with those reported in the literature.[5]

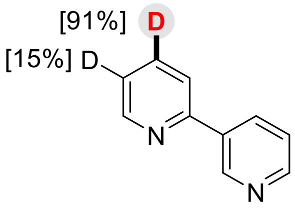


***2,3'-Bipyridine-4,5-d$_2$ (44)***

The compounds were prepared following a general procedure and purified on silica gel (petroleum ethers ethyl acetate = 10:1 to 3:1) by column chromatography to obtain (46.4 mg, 98%) white solid.

**$^{1}$H NMR (400 MHz, CDCl$_3$)** δ = 9.15 (d, *J* = 2.0 Hz, 1H), 8.66 (d, *J* = 4.4 Hz, 1H), 8.60 – 8.59 (m, 1H), 8.28 – 8.25 (m, 1H), 7.73 – 7.69 (m, labeled, 1.09H, 91% D), 7.36 – 7.33 (m, 1H), 7.23 (d, *J* = 4.8 Hz, labeled, 0.85H, 15% D).

**$^{13}$C NMR (100 MHz, CDCl$_3$)** δ = 154.6, <u>149.9 (149.8) (1C)</u>, 149.7, 148.1, 136.8 – 136.3 (m, labeled, 1C), 134.7, 134.2, <u>123.4 (123.3) (1C)</u>, <u>(122.7) 122.6 (C)</u>, <u>(120.4) 120.3 (1C)</u>.

**HRMS (ESI, m/z)**: calculated for $C_{10}H_7N_2D_2$ $[M+H]^+$: 159.0886, found: 159.0895.

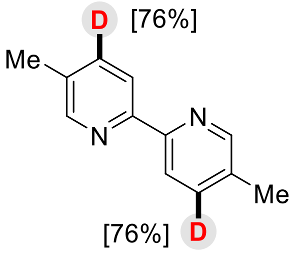


***5,5'-Dimethyl-2,2'-bipyridine-4,4'-d$_2$* (45)**

The compounds were prepared following a general procedure and purified on silica gel (petroleum ethers ethyl acetate = 10:1 to 3:1) by column chromatography to obtain (55.3 mg, 99%) white solid.

**$^1$H NMR (400 MHz, CDCl$_3$)** δ = 8.44 (s, 2H), 8.21 – 8.19 (m, 2H), 7.56 – 7.53 (m, labeled, 0.48H, 76% D), 2.31 (s, 6H).

**$^{13}$C NMR (100 MHz, CDCl$_3$)** δ = 153.5, 149.3, 137.3 – 136.7 (m, labeled, 2C), <u>(132.9) 132.8 (2C)</u>, <u>(120.2) 120.0 (2C)</u>, <u>(18.2) 18.1 (2C)</u>.

**HRMS (ESI, m/z)**: calculated for $C_{12}H_{11}N_2D_2$ $[M+H]^+$: 187.1199, found: 187.1195.

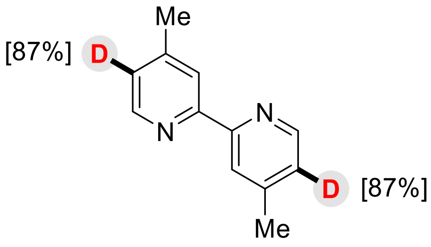


***4,4'-Dimethyl-2,2'-bipyridine-5,5'-d$_2$* (46)**

The compounds were prepared following a general procedure and purified on silica gel (petroleum ethers ethyl acetate = 10:1 to 3:1) by column chromatography to obtain (54.7 mg, 98%) yellow solid.

**$^1$H NMR (400 MHz, CDCl$_3$)** δ = 8.54 (s, 2H), 8.23 (s, 2H), 7.15 – 7.13 (m, labeled, 0.26H, 87% D), 2.44 (s, 6H).

**$^{13}$C NMR (100 MHz, CDCl$_3$)** δ = 155.7, <u>(148.8) 148.7 (2C)</u>, 148.3, 124.7 – 124.2 (m, labeled, 2C), 122.1, 21.1.

**HRMS (ESI, m/z)**: calculated for $C_{12}H_{11}N_2D_2$ $[M+H]^+$: 187.1199, found: 187.1205.

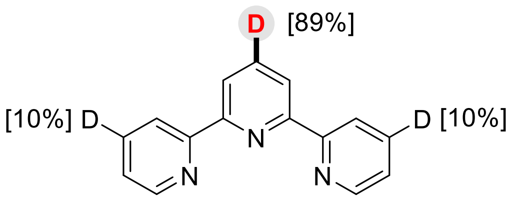


***2,2':6',2''-Terpyridine-4,4',4''-$d_3$ (47)***

The compounds were prepared following a general procedure and purified on silica gel (petroleum ethers ethyl acetate = 10:1 to 2:1) by column chromatography to obtain (70.1 mg, 99%) white solid.

**$^1$H NMR (400 MHz, $CDCl_3$)** δ = 8.69 – 8.67 (m, 2H), 8.60 (d, *J* = 8.0 Hz, 2H), 8.45 – 8.43 (m, labeled, 1.80H, 10% D), 7.95 – 7.91 (m, labeled, 0.11H, 89% D), 7.84 – 7.80 (m, 2H), 7.31 – 7.28 (m, 2H).

**$^{13}$C NMR (100 MHz, $CDCl_3$)** δ = 156.1, 155.2, 149.0, 137.8 – 137.2 (m, labeled, 1C), 136.7, 123.6, 121.1, (120.9) 120.8 (2C).

**HRMS (ESI, m/z)**: calculated for $C_{15}H_9N_3D_3$ $[M+H]^+$: 237.1214, found: 237.1225.

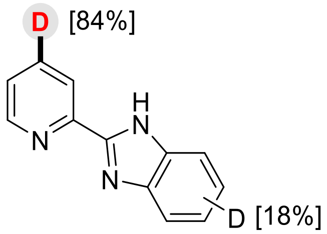


***2-(Pyridin-2-yl-4-d)-1H-benzo[d]imidazole (48)***

The compounds were prepared following a general procedure and purified on silica gel (petroleum ethers ethyl acetate = 20:1 to 3:1) by column chromatography to obtain (55.3 mg, 94%) yellow solid.

**$^1$H NMR (400 MHz, $CDCl_3$)** δ = 11.65 (s, 1H), 8.63 (d, *J* = 4.8 Hz, 1H), 8.52 (s, 1H), 7.90 – 7.85 (m, labeled, 0.16H, 84% D), 7.62 (s, 1.82H, 18% D), 7.39 – 7.36 (m, 1H), 7.29 – 7.25 (m, 2H).

**$^{13}$C NMR (100 MHz, $CDCl_3$)** δ = 150.7, 148.9, 148.4, 137.5 – 136.9 (m, labeled, 1C), (124.6) 124.5 (2C), 123.3, (121.9) 121.8 (2C).

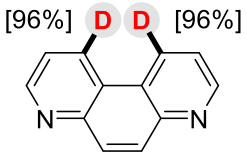

***4,7-Phenanthroline-1,10-d$_2$ (49)***

The compounds were prepared following a general procedure and purified on silica gel (petroleum ethers ethyl acetate = 10:1 to 3:1) by column chromatography to obtain (54.0 mg, 99%) yellow solid.

**$^1$H NMR (400 MHz, CDCl$_3$)** δ = 8.95 (d, *J* = 4.0 Hz, 2H), 8.77 (d, *J* = 8.4 Hz, labeled, 0.09H, 96% D), 8.17 (s, 2H), 7.52 (d, *J* = 4.4 Hz, 2H).

**$^{13}$C NMR (100 MHz, CDCl$_3$)** δ = 150.3, 147.5, 132.0, 130.4 – 129.9 (m, labeled, 2C), 124.5, (121.6) 121.5 (2C).

**HRMS (ESI, m/z)**: calculated for $C_{12}H_7N_2D_2$ [M+H]$^+$: 183.0886, found: 183.0897.

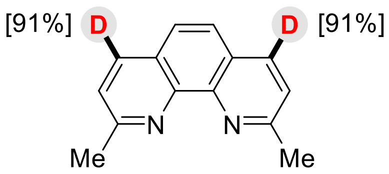


***2,9-Dimethyl-1,10-phenanthroline-4,7-d$_2$ (50)***

The compounds were prepared following a general procedure and purified on silica gel (petroleum ethers ethyl acetate = 10:1 to 3:1) by column chromatography to obtain (61.1 mg, 97%) yellow solid.

**$^1$H NMR (400 MHz, CDCl$_3$)** δ = 8.07 (d, *J* = 8.0 Hz, labeled, 0.18H, 91% D), 7.65 (s, 2H), 7.45 – 7.43 (m, 2H), 2.91 (s, 6H).

**$^{13}$C NMR (100 MHz, CDCl$_3$)** δ = 159.2, 145.1, 136.0 – 135.6 (m, labeled, 2C), (126.7) 126.6 (2C), 125.3 – 125.2 (m, 2C), 123.2 (d, $J_{C\text{-}D}$ = 11.0 Hz, 2C), 25 (d, $J_{C\text{-}D}$ = 12.0 Hz, 2C).

**HRMS (ESI, m/z)**: calculated for $C_{14}H_{11}N_2D_2$ [M+H]$^+$: 211.1199, found: 211.1205.

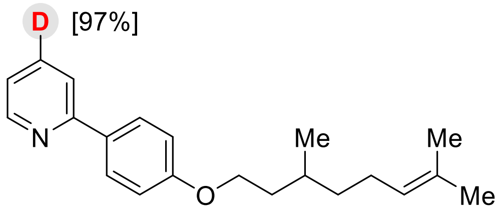


***2-(4-((3,7-Dimethyloct-6-en-1-yl)oxy)phenyl)pyridine-4-d (51)***

The compounds were prepared following a general procedure and purified on silica gel (petroleum ethers ethyl acetate = 15:1) by column chromatography to obtain (92.1 mg, 99%) white solid.

**$^{1}$H NMR (400 MHz, CDCl$_3$)** δ = 8.65 – 8.64 (m, 1H), 7.96 – 7.92 (m, 2H), 7.70 – 7.66 ( m, labeled, 1.03 H, 97% D), 7.16 – 7.15 (m, 1H), 7.01 – 6.97 (m, 2H), 5.14 – 5.09 (m, 1H), 4.09 – 4.00 (m, 2H), 2.06 – 1.98 (m, 2H), 1.90 – 1.82 (m, 1H), 1.69 (d, *J* = 1.6 Hz, 3H), 1.62 (d, *J* = 1.2 Hz, 3H), 1.46 – 1.36 (m, 1H), 1.30 – 1.19 (m, 3H), 0.97 (d, J = 6.4 Hz, 3H).

**$^{13}$C NMR (100 MHz, CDCl$_3$)** δ = 160.0, 157.1, 149.5, 131.7, 131.3, 128.1, 124.6, 121.2, 119.6, 114.6, 66.3, 37.1, 36.1, 29.5, 25.7, 25.4, 19.5, 17.6.

**HRMS (ESI, m/z)**: calculated for $C_{21}H_{27}NOD$ $[M+H]^+$: 311.2228, found: 311.2220.

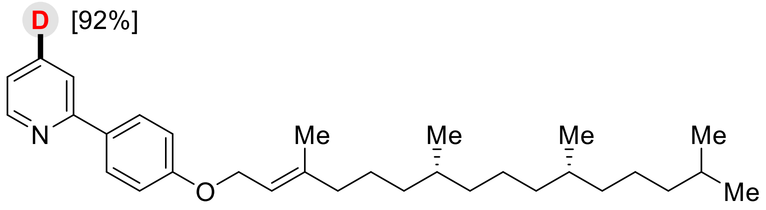


***2-(4-(((7R,11R,E)-3,7,11,15-tetramethylhexadec-2-en-1-yl)oxy)phenyl)pyridine-4-d (52)***

The compounds were prepared following a general procedure and purified on silica gel (petroleum ethers ethyl acetate = 15:1) by column chromatography to obtain (133.7 mg, 99%) yellow oil.

**$^{1}$H NMR (400 MHz, CDCl$_3$)** δ = 8.65 (d, *J* = 4.8 Hz, 1H), 7.96 – 7.92 (m, 2H), 7.71 – 7.67 (m, labeled, 1.08H, 92% D), 7.18 – 7.15 (m, 1H), 7.03 – 6.99 (m, 2H), 5.52 – 5.49 (m, 1H), 4.60 (d, *J* = 6.8 Hz, 2H), 2.07 – 2.03 (m, 2H), 1.74 (s, 3H), 1.55 – 1.46 (m, 2H), 1.44 – 1.34 (m, 4H), 1.32 – 1.21 (m, 8H), 1.16 – 1.12 (m, 2H), 1.09 – 1.03 (m, 3H), 0.87 – 0.83 (m, 12H).

**$^{13}$C NMR (100 MHz, CDCl$_3$)** δ = 159.8, 157.1, 149.5, 141.8, 136.7 – 136.1 (m, labeled, 1C), 131.8, 128.1, (121.3) 121.2 (1C), (119.8) 119.7 (1C), 119.1, 114.9, 64.9, 39.9, 39.3, 37.4, 37.35, 37.27, 36.6, 32.8, 32.7, 28.0, 25.0, 24.8, 24.4, 22.7, 22.6, 19.74, 19.71, 16.61.

**HRMS (ESI, m/z)**: calculated for $C_{31}H_{47}NOD$ $[M+H]^+$: 451.3793, found: 451.3805.

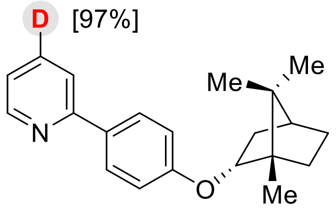


***2-(4-(((1S,2R,4S)-1,7,7-trimethylbicyclo[2.2.1]heptan-2-yl)oxy)phenyl)pyridine-4-d (53)***

The compounds were prepared following a general procedure and purified on silica gel (petroleum ethers ethyl acetate = 15:1) by column chromatography to obtain (90.6 mg, 98%) white solid.

**$^{1}$H NMR (400 MHz, $CDCl_3$)** δ = 8.65 (d, *J* = 4.8 Hz, 1H), 7.96 (d, *J* = 8.0 Hz, 2H), 7.64 (s, labeled, 1.03H, 97% D), 7.12 (d, *J* = 4.8 Hz, 1H), 7.01 (d, *J* = 8.4 Hz, 2H), 4.71 (s, 1H), 2.15 (d, *J* = 14.0 Hz, 1H), 1.82 – 1.68 (m, 4H), 1.64 – 1.57 (m, 1H), 1.10 – 1.05 (m, 1H), 0.95 (d, *J* = 6.8 Hz, 3H), 0.89 (d, *J* = 6.8 Hz, 3H), 0.83 (d, *J* = 6.8 Hz, 3H).

**$^{13}$C NMR (100 MHz, $CDCl_3$)** δ = 159.1, 157.1, 149.3, 136.4 – 135.9 (m, labeled, 1C), 131.3, 128.1, (121.1) 121.0 (1C), (119.5) 119.4 (1C), 115.6, 73.3, 47.6, 37.6, 34.9, 29.2, 26.0, 24.7, 22.2, 20.9, 20.7,

**HRMS (ESI, m/z)**: calculated for $C_{21}H_{25}NOD$ $[M+H]^+$: 309.2072, found: 309.2085.

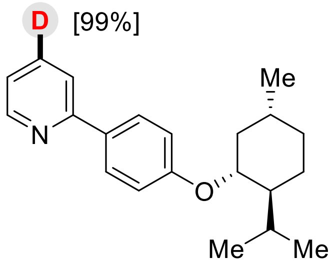


***2-(4-(((1R,2S,5R)-2-isopropyl-5-methylcyclohexyl)oxy)phenyl)pyridine-4-d (54)***

The compounds were prepared following a general procedure and purified on silica gel (petroleum ethers ethyl acetate = 15:1) by column chromatography to obtain (92.1 mg, 99%) yellow oil.

**$^{1}$H NMR (400 MHz, $CDCl_3$)** δ = 8.67 – 8.63 (m, 1H), 7.97 – 7.93 (m, 2H), 7.67 – 7.65 (m, labeled, 1.01H, 99% D), 7.14 – 7.11 (m, 1H), 7.03 – 6.99 (m, 2H), 4.71 (q, *J* = 2.8 Hz, 1H), 2.17 – 2.12 (m, 1H), 1.82 – 1.69 (m, 4H), 1.67 – 1.56 (m, 1H), 1.10 – 1.01 (m, 2H), 0.95 (d, *J* = 6.8 Hz, 3H), 0.89 (d, *J* = 6.4 Hz, 3H), 0.83 (d, *J* = 6.4 Hz, 3H).

**$^{13}$C NMR (100 MHz, $CDCl_3$)** δ = 159.1, 157.1, 149.4, 136.4 – 135.9 (m, labeled, 1C), 131.3, 128.1, (121.1) 121.0 (1C), (119.5) 119.4 (1C), 115.6, 73.2, 47.6, 37.5, 34.8, 29.2, 26.0, 24.8, 22.2, 21.0, 20.7.

**HRMS (ESI, m/z)**: calculated for $C_{21}H_{27}NOD$ $[M+H]^+$: 311.2228, found: 311.2235.

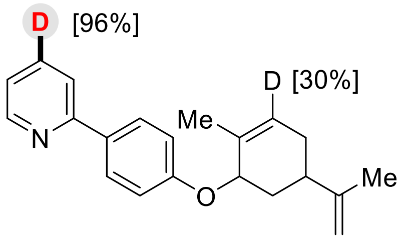


***2-(4-((2-methyl-5-(prop-1-en-2-yl)cyclohex-2-en-1-yl-3-d)oxy)phenyl)pyridine-4-d (55)***

The compounds were prepared following a general procedure and purified on silica gel (petroleum ethers ethyl acetate = 10:1) by column chromatography to obtain (91.2 mg, 99%) yellow oil.

**$^1$H NMR (400 MHz, CDCl$_3$)** δ = 8.65 (d, *J* = 4.8 Hz, 1H)， 7.97 – 7.94 (m, 2H), 7.68 – 7.65 (m, labeled, 1.04H, 96% D), 7.14 (d, *J* = 4.8 Hz, 1H), 7.07 – 7.05 (m, 2H), 5.79 – 5.62 (m, 1H), 4.96 – 4.91 (m, labeled, 0.7H, 30% D), 4.74 – 4.67 (m, 2H), 2.51 – 2.22 (m, 2H), 2.20 – 1.88 (m, 2H), 1.84 (d, *J* = 2.4 Hz, 1H), 1.79 (s, 2H), 1.72 (d, *J* = 5.6 Hz, 3H), 1.68 – 1.56 (m, 1H).

**$^{13}$C NMR (100 MHz, CDCl$_3$)** δ = 159.4, 159.3, 156.9, 149.4, 148.8, 148.3, 136.5 – 135.9 (m, labeled, 1C), 134.1, 132.0 – 131.7 (m, labeled, 1C), (128.11) 128.07 (1C), 127.1, 125.3, (121.2) 121.1 (1C), (119.6) 119.5 (1C), 116.2, 115.9, 109.3, 109.0, 74.7, 40.4, 35.5, 33.9, 32.3, 30.9, 20.8 (d, $J_{C–D}$ = 8.0 Hz, 1C), 20.2, 19.1.

**HRMS (ESI, m/z)**: calculated for $C_{21}H_{22}NOD_2$ $[M+H]^+$: 308.1978, found: 308.1989.

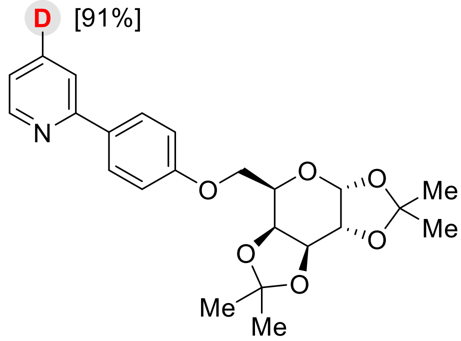


***2-(4-(((3aR,5R,5aS,8aS,8bR)-2,2,7,7-tetramethyltetrahydro-5H-bis([1,3]dioxolo)[4,5-b:4',5'-d]pyran-5-yl)methoxy)phenyl)pyridine-4-d (56)***

The compounds were prepared following a general procedure and purified on silica gel (petroleum ethers ethyl acetate = 10:1 to 5:1) by column chromatography to obtain (91.2 mg, 99%) yellow oil.

**$^1$H NMR (400 MHz, CDCl$_3$)** δ = 8.62 (d, *J* = 4.8 Hz, 1H), 7.93 – 7.91 (m, 2H), 7.66 – 7.62 (m, labeled, 1.09H, 91% D), 7.12 (d, *J* = 4.8 Hz, 1H), 7.03 – 7.01 (m, 2H), 5.57 (d, *J* = 5.2 Hz, 1H), 4.65 – 4.62 (m, 1H), 4.37 – 4.32 (m, 2H), 4.23 – 4.15 (m, 3H), 1.51 (s, 3H), 1.46 (s, 3H), 1.33 (d, *J* = 8.4 Hz, 6H).

**$^{13}$C NMR (100 MHz, CDCl$_3$)** δ = 159.4, 156.9, 149.4, 136.5 – 136.0 (m, labeled, 1C), 132.1, 127.9, (121.3) 121.2 (1C), (119.6) 119.5 (1C), 114.8, 109.3, 108.6, 96.2, 70.9, 70.52, 70.49, 66.6, 66.1, 26.0, 25.9, 24.8, 24.3.

**HRMS (ESI, m/z)**: calculated for $C_{23}H_{27}NO_6D$ $[M+H]^+$: 415.1974, found: 415.1985

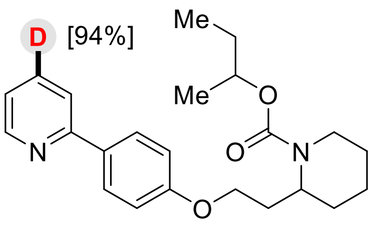


***Sec-butyl 2-(2-(4-(pyridin-2-yl-4-d)phenoxy)ethyl)piperidine-1-carboxylate (57)***

The compounds were prepared following a general procedure and purified on silica gel (petroleum ethers ethyl acetate = 30:1 to 20:1) by column chromatography to obtain (109.2 mg, 95%) light yellow solid.

**$^1$H NMR (400 MHz, CDCl$_3$)** δ = 8.62 (d, *J* = 4.8 Hz, 1H), 7.92 – 7.90 (m, 2H), 7.67 – 7.63 (m, labeled, 1.06H, 94%D), 7.13 (d, *J* = 4.8 Hz, 1H), 6.94 (d, *J* = 8.4 Hz, 2H), 4.75 – 4.66 (m, 1H), 4.57 – 4.51 (m, 1H), 4.12 – 3.93 (m, 3H), 2.85 (t, *J* = 13.2 Hz, 1H), 2.31 – 2.20 (m, 1H), 1.94 – 1.82 (m, 1H), 1.69 – 1.57 (m, 5H), 1.56 – 1.40 (m, 3H), 1.25 – 1.15 (m, 2H), 1.08 (s, 1H), 0.88 – 0.82 (m, 3H).

**$^{13}$C NMR (100 MHz, CDCl$_3$)** δ = 159.6, 157.0, (155.53) 155.51 (1C), 149.4, 136.5 – 136.0 (m, labeled, 1C), 131.8, 128.0, 121.1, 119.5, (114.49) 114.47 (1C), (72.84) 72.77 (1C), 65.4, 47.9 (47.8) (1C), 38.9, (29.49) 29.46 (1C), 29.0, 28.8, (25.5) 25.4 (1C), 19.7 (19.6) (1C), 19.0, 9.63 (9.59) (1C).

**HRMS (ESI, m/z)**: calculated for $C_{23}H_{30}N_2O_3D$ $[M+H]^+$: 384.2392, found: 384.2405.

note: The small peaks in parentheses are due to the splitting effect of the deuterium atom.

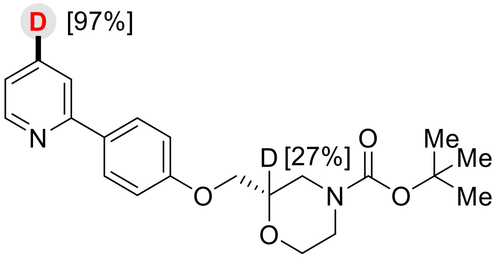


***Tert-butyl (S)-2-((4-(pyridin-2-yl-4-d)phenoxy)methyl)morpholine-4-carboxylate-2-d (58)***

The compounds were prepared following a general procedure and purified on silica gel (petroleum ethers ethyl acetate = 30:1 to 20:1) by column chromatography to obtain (110.3 mg, 99%) light yellow solid.

**$^1$H NMR (400 MHz, CDCl$_3$)** δ = 8.61 (d, *J* = 4.8 Hz, 1H), 7.92 – 7.90 (m, 2H), 7.65 – 7.62 (m, labeled, 1.03H, 97%D), 7.13 (d, *J* = 4.8 Hz, 1H), 6.98 – 6.96 (m, 2H), 4.97 – 4.88 (m, labeled,

0.73H, 27%D), 4.08 – 4.04 (m, 1H), 4.00 – 3.96 (m, 1H), 3.81 – 3.75 (m, 1H), 3.60 – 3.54 (m, 1H), 9.09 (s, 9H), 1.21 (d, *J* = 6.4 Hz, 4H).

**$^{13}$C NMR (100 MHz, $CDCl_3$)** δ = 159.2, 156.8, 154.6, 149.4, 136.2 – 136.0 (m, labeled, 1C), 132.3, 128.0, 121.2, 119.6, 114.6, 80.0, 73.6, 69.6, 68.5, 66.5, 28.2, 21.8.

**HRMS (ESI, m/z)**: calculated for $C_{21}H_{25}N_2O_4D_2$ $[M+H]^+$: 373.2091, found: 373.2081.

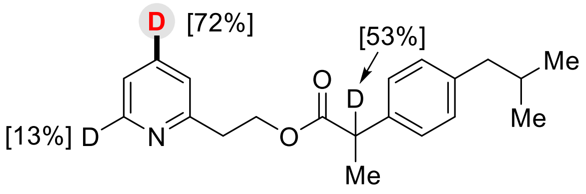


***2-(Pyridin-2-yl-4,6-d2)ethyl 2-(4-isobutylphenyl)propanoate-2-d (59)***

The compounds were prepared following a general procedure and purified on silica gel (petroleum ethers ethyl acetate = 15:1 to 6:1) by column chromatography to obtain (91.7 mg, 98%) light yellow solid.

**$^{1}$H NMR (400 MHz, $CDCl_3$)** δ = 8.50 – 8.48 (m, labeled, 0.87H, 13%D), 7.49 – 7.45 (m, labeled, 0.28H, 72%D), 7.12 (d, *J* = 8.0 Hz, 2H), 7.09 – 7.03 (m, 3H), 6.94 – 6.92 (m, 1H), 4.50 – 4.38 (m, 2H), 3.63 (q, *J* = 7.2 Hz, labeled, 0.47H, 53%D), 3.05 – 3.01 (m, 2H), 2.43 (d, *J* = 7.2 Hz, 2H), 1.88 – 1.78 (m, 1H), 1.43 (d, *J* = 6.8 Hz, 3H), 0.89 (d, *J* = 6.8Hz, 6H).

**$^{13}$C NMR (100 MHz, $CDCl_3$)** δ = 174.4, 157.9, 149.2, 140.3, <u>137.6 (137.5) (1C)</u>, 136.1 – 135.6 (m, labeled, 1C), 129.1, <u>127.05 (127.04) (1C)</u>, <u>(123.3), 123.2 (1C)</u>, <u>(121.4) 121.3 (1C)</u>, 63.6, 44.9 – 44.6 (m, labeled, 1C), 37.2, 30.1, 22.3, <u>18.2 (18.1) (1C)</u>.

**HRMS (ESI, m/z)**: calculated for $C_{20}H_{23}NO_2D_3$ $[M+H]^+$: 315.2147, found: 315.2155.

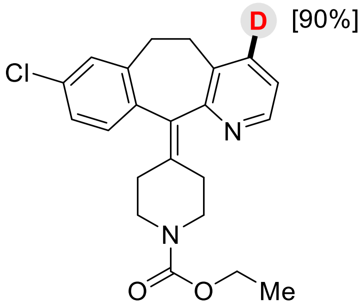


***Ethyl-4-(8-chloro-5,6-dihydro-11H-benzo[5,6]cyclohepta[1,2-b]pyridin-11-ylidene-4-d)piperidine-1-carboxylate (60)***

The compounds were prepared following a general procedure and purified on silica gel (petroleum ethers ethyl acetate = 15:1 to 10:1) by column chromatography to obtain (103.4 mg, 90%) light yellow solid.

**$^1$H NMR (400 MHz, CDCl$_3$)** δ = 8.30 (d, *J* = 4.8 Hz, 1H), 7.36 (d, *J* = 8.0 Hz, labeled, 0.10H, 90% D), 7.14 – 7.08 (m, 3H), 7.03 (d, *J* = 4.4 Hz, 1H), 4.10 (q, *J* = 7.2 Hz, 4H), 3.49 – 3.36 (m, 2H), 2.91 – 2.85 (m, 2H), 2.59 – 2.49 (m, 2H), 1.44 – 1.27 (m, 3H), 1.20 (t, *J* = 7.2 Hz, 4H).

**$^{13}$C NMR (100 MHz, CDCl$_3$)** δ = 158.8, 155.3, 146.0, 138.4 (m, labeled, 1C), 133.9, 131.8, 130.1, 130.0, 126.9, 125.9, 125.8, 121.6 (121.5) (1C), 61.0, 43.92 (43.86) (2C), 32.0 (d, $J_{C-D}$ = 26.0 Hz, 1C), 31.1 (d, $J_{C-D}$ = 24.0 Hz, 1C), 14.6 (2C).

The analytical data corresponds with those reported in the literature.[4]

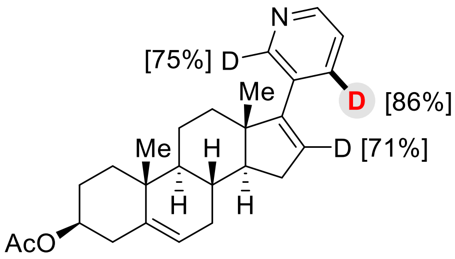


***(3S,8R,9S,10R,13S,14S)-10,13-Dimethyl-17-(pyridin-3-yl-2,4-d$_2$)-2,3,4,7,8,9,10,11,12,13,14,15-dodecahydro-1H-cyclopenta[a]phenanthren-3-yl-16-d acetate (61)***

The compounds were prepared following a general procedure and purified on silica gel (petroleum ethers ethyl acetate = 15:1 to 8:1) by column chromatography to obtain (106.4 mg, 90%) light yellow solid.

**$^1$H NMR (400 MHz, CDCl$_3$)** δ = 8.59 (s, labeled, 0.25H, 75% D), 8.43 – 8.40 (m, 1H), 7.50 (d, *J* = 8.0 Hz, labeled, 0.14H, 86% D), 7.18 – 7.16 (m, 1H), 5.97 – 5.96 (m, labeled, 0.29H, 71% D), 5.39 – 5.36 (m, 1H), 4.63 – 4.54 (m, 1H), 2.35 – 2.20 (m, 2H), 2.06 – 1.97 (m, 4H), 1.86 – 1.74 (m, 3H), 1.64 – 1.47 (m, 5H), 1.39 – 1.21 (m, 3H), 1.16 – 1.09 (m, 1H), 1.05 – 0.98 (m, 5H), 0.46 (s, 2H).

The analytical data corresponds with those reported in the literature.[4]

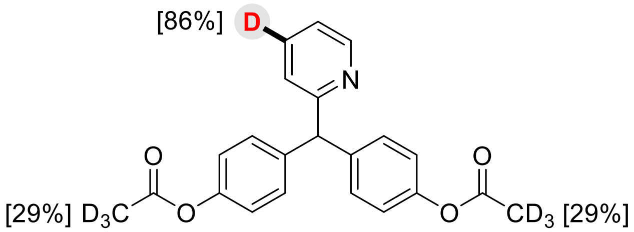

***((Pyridin-2-yl-4-d)Methylene)bis(4,1-phenylene) bis(acetate-d$_6$) (62)***

The compounds were prepared following a general procedure and purified on silica gel (petroleum ethers ethyl acetate = 15:1 to 8:1) by column chromatography to obtain (101.5 mg, 92%) yellow solid.

**$^1$H NMR (400 MHz, CDCl$_3$)** δ = 8.59 (d, *J* = 4.8 Hz, 1H), 7.64 – 7.60 (m, labeled, 0.14H, 86% D), 7.18 – 7.14 (m, 5H), 7.10 (s, 1H), 7.02 (d, *J* = 8.0 Hz, 4H), 5.68 (s, 1H), 2.28 – 2.26 (m, labeled, 5.42H, 29% D).

**$^{13}$C NMR (100 MHz, CDCl$_3$)** δ = 169.3, 162.5, 149.4, 149.3, 139.9, 136.7 (m, labeled, 1C), 130.2, (123.8) 123.7 (1C), (121.6) 121.5 (1C), 121.4, 57.9, 21.1 – 20.6 (m, labeled, 2C).

The analytical data corresponds with those reported in the literature.[4]

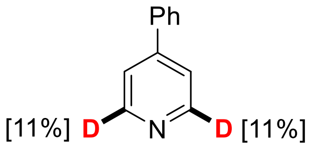


***4-Phenylpyridine-2,6-d2 (63)***

The compounds were prepared following a general procedure and purified on silica gel (petroleum ethers ethyl acetate = 10:1) by column chromatography to obtain (46.4 mg, 99%) yellow solid.

**$^1$H NMR (400 MHz, CDCl$_3$)** δ = 8.67 – 8.65 (m, labeled, 1.78H, 11%D), 7.64 – 7.62 (m, 2H), 7.50 – 7.41 (m, 5H).

**$^{13}$C NMR (100 MHz, CDCl$_3$)** δ = 150.2, 148.2, 138.0, 129.1, 129.0, 126.9, 121.6.

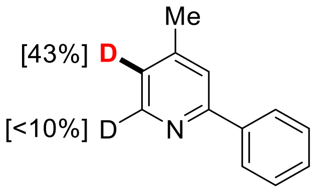


***4-Methyl-2-phenylpyridine-5-d (64)***

The compounds were prepared following a general procedure and purified on silica gel (petroleum ethers ethyl acetate = 30:1) by column chromatography to obtain (49.9 mg, 99%) yellow liquid.

**$^1$H NMR (400 MHz, CDCl$_3$)** δ = 8.56 – 8.55 (m, 1H), 8.00 – 7.97 (m, 2H), 7.54 (s, 1H), 7.49 – 7.45 (m, 2H), 7.42 – 7.38 (m, 1H), 7.06 – 7.04 (m, labeled, 0.57H, 43%D), 2.40 (s, 3H).

**$^{13}$C NMR (100 MHz, $CDCl_3$)** δ = 157.3, 149.34 (149.27) (1C), 147.65 (147.57) (1C), 139.45, 128.7, 128.6, 126.8, 123.0, 121.4, 21.15 (21.09) (1C).

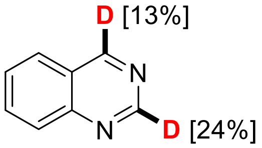


***Quinazoline-2,4-d2 (65)***

The compounds were prepared following a general procedure and purified on silica gel (petroleum ethers ethyl acetate = 20:1) by column chromatography to obtain (38.0 mg, 96%) yellow liquid.

**$^{1}$H NMR (400 MHz, $CDCl_3$)** δ = 9.41 (s, labeled, 0.76H, 24%D), 9.33 (s, labeled, 0.87H, 13%D), 8.07 – 8.06 (m, 1H), 7.95 – 7.91 (m, 2H), 7.69 – 7.65 (m, 1H).

**$^{13}$C NMR (100 MHz, $CDCl_3$)** δ = 160.2, 155.2, 150.0, 134.2, 128.4, 127.9, 127.2, 125.1.

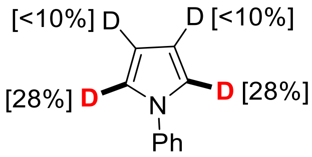


***1-Phenyl-1H-pyrrole-2,3,4,5-d4 (66)***

The compounds were prepared following a general procedure and purified on silica gel (petroleum ethers ethyl acetate = 30:1) by column chromatography to obtain (43.0 mg, 98%) yellow liquid.

**$^{1}$H NMR (400 MHz, $CDCl_3$)** δ = 7.51 – 7.45 (m, 4H), 7.33 – 7.29 (m, 1H), 7.19 – 7.17 (m, labeled, 1.44H, 28%D), 6.45 – 6.44 (m, labeled, 1.87H, <10%D).

**$^{13}$C NMR (100 MHz, $CDCl_3$)** δ = 140.6, 129.5, 125.5, 120.41 – 120.36 (labeled, 2C), 119.2 (119.1) (2C), 110.3 (110.1) (1C).

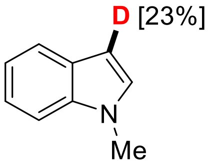


***1-Methyl-1H-indole-3-d (67)***

The compounds were prepared following a general procedure and purified on silica gel (petroleum ethers ethyl acetate = 30:1) by column chromatography to obtain (34.9 mg, 88%) brown solid.

**$^{1}$H NMR (400 MHz, CDCl$_3$)** δ = 7.89 (d, *J* = 8.0 Hz, 1H), 7.54 (d, *J* = 8.4 Hz, 2H), 7.49 – 7.45 (m, 1H), 7.38 – 7.35 (m, 1H), 7.24 (d, *J* = 2.4 Hz, 1H), 6.73 (d, *J* = 3.2 Hz, labeled, 0.77H, 23%D), 3.94 (s, 3H).

**$^{13}$C NMR (100 MHz, CDCl$_3$)** δ = 136.6, <u>128.7 (128.6) (1C)</u>, 128.4, 121.4, <u>120.8 (120.7) (1C)</u>, 119.2, 109.1, 100.8, 32.6.

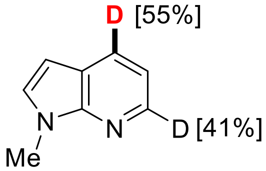


***1-Methyl-1H-pyrrolo[2,3-b]pyridine-4,6-d2 (68)***

The compounds were prepared following a general procedure and purified on silica gel (petroleum ethers ethyl acetate = 30:1 to 15:1) by column chromatography to obtain (38.6 mg, 96%) yellow solid.

**$^{1}$H NMR (400 MHz, CDCl$_3$)** δ = 8.34 – 8.33 (m, labeled, 0.59H, 41%D), 7.91 – 7.88 (m, labeled, 0.45H, 55% D), 7.17 (d, *J* = 3.6 Hz, 1H), 7.06 – 7.03 (m, 1H), 6.45 – 6.44 (d, *J* = 3.2 Hz, 1H), 3.88 (s, 3H).

**$^{13}$C NMR (100 MHz, CDCl$_3$)** δ = 147.7, 142.8, 128.9, 128.6 – 128.1 (m, labeled, 1C), <u>120.4 (120.3) (1C)</u>, 115.4 – 115.2 (m, labeled, 1C), <u>99.2 (99.1) (1C)</u>, 31.2.

**HRMS** (**ESI, m**/**z**): calculated for $C_8H_6N_2D_2$ $[M+H]^+$: 135.0886, found: 135.0890.

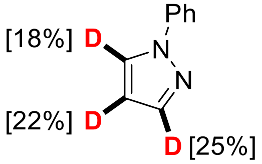


***1-Phenyl-1H-pyrazole-3,4,5-d3 (69)***

The compounds were prepared following a general procedure and purified on silica gel (petroleum ethers ethyl acetate = 30:1) by column chromatography to obtain (43.5 mg, 99%) brown oil.

**$^{1}$H NMR (400 MHz, CDCl$_3$)** δ = 7.90 (d, *J* = 2.4 Hz, labeled, 0.82H, 18%D), 7.72 – 7.67 (m, labeled, 2.75H, 25%D), 7.45 – 7.41 (m, 2H), 7.28 – 7.24 (m, 1H), 6.45 – 6.44 (m, labeled, 0.78H, 22%D).

**$^{13}$C NMR (100 MHz, CDCl$_3$)** δ = 141.0, 140.1, 129.3, 126.6, 126.3, 119.1, 107.5.

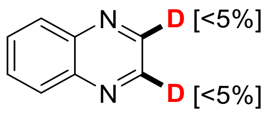


***Quinoxaline-2,3-d2 (70)***

The compounds were prepared following a general procedure and purified on silica gel (petroleum ethers ethyl acetate = 20:1) by column chromatography to obtain (39.1 mg, 99%) light yellow solid.

**$^1$H NMR (400 MHz, DMSO)** δ = 8.86 (d, *J* = 3.6 Hz, labeled, 1.96H, <5%D), 8.15 – 8.11 (m, 2H), 7.81 – 7.77 (m, 2H).

**$^{13}$C NMR (100 MHz, DMSO)** δ = 144.9, 143.0, 130.0, 129.4.

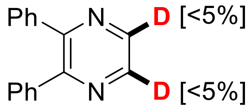


***2,3-Diphenylpyrazine-5,6-d2 (71)***

The compounds were prepared following a general procedure and purified on silica gel (petroleum ethers ethyl acetate = 20:1) by column chromatography to obtain (69.2 mg, 99%) white solid.

**$^1$H NMR (400 MHz, $CDCl_3$)** δ = 8.51 (s, labeled, 1.96H, <5%D), 7.40 – 7.37 (m, 4H), 7.24 – 7.18 (m, 6H).

**$^{13}$C NMR (100 MHz, $CDCl_3$)** δ = 152.6, 141.9, 138.4, 129.5, 128.5, 128.1.

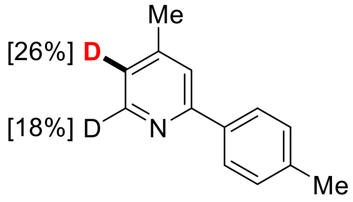


***4-Methyl-2-(p-tolyl)pyridine-5,6-d2 (72)***

The compounds were prepared following a general procedure and purified on silica gel (petroleum ethers ethyl acetate = 30:1) by column chromatography to obtain (54.9 mg, 99%) light yellow solid.

**$^1$H NMR (400 MHz, $CDCl_3$)** δ = 8.52 (d, *J* = 4.8 Hz, labeled, 0.82H, 18%D), 7.87 (d, *J* = 8.0 Hz, labeled, 2H), 7.51 (s, 1H), 7.26 (d, *J* = 8.0 Hz, labeled, 2H), 7.01 (d, *J* = 4.8 Hz, labeled, 0.74H, 26%D), 2.39 (d, *J* = 3.0 Hz, 6H).

**$^{13}$C NMR (100 MHz, $CDCl_3$)** δ = 157.3, 149.25 (149.18) (1C), 147.6 (147.5) (1C), 138.7, 136.6, 129.4, 126.7, 122.8 (122.7) (1C), 121.2, 21.2 – 21.1 (1C).

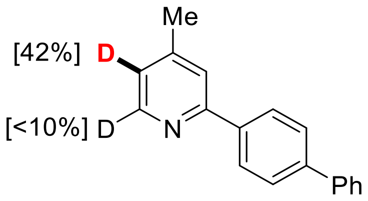


***2-([1,1'-Biphenyl]-4-yl)-4-methylpyridine-5,6-d2 (73)***

The compounds were prepared following a general procedure and purified on silica gel (petroleum ethers ethyl acetate = 30:1) by column chromatography to obtain (73 mg, 99%) light yellow solid.

**$^{1}$H NMR (400 MHz, DMSO-$d_6$)** δ = 8.59 – 8.47 (m, labeled, 0.94H, <10%D), 8.10 – 8.08 (m, 2H), 7.74 – 7.66 (m, 4H), 7.60 (s, 1H), 7.49 – 7.45 (m, 2H), 7.39 – 7.36 (m, 1H), 7.07 (d, $J$ = 5.2 Hz, labeled, 0.58H, 42%D), 2.42 (s, 3H).

**$^{13}$C NMR (100 MHz, DMSO-$d_6$)** δ = 156.9, 149.43 (149.37) (1C), 147.7 (147.6) (1C), 141.5, 140.6, 138.4, 128.7, 127.4, 127.3, 127.2, 127.0, 123.1, 121.3, 21.2 (21.1) (1C).

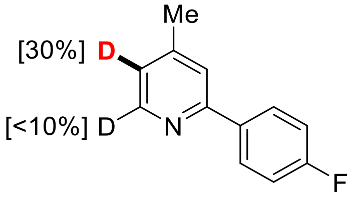


***2-(4-fluorophenyl)-4-methylpyridine-5,6-d2 (74)***

The compounds were prepared following a general procedure and purified on silica gel (petroleum ethers ethyl acetate = 25:1) by column chromatography to obtain (55.8 mg, 99%) yellow oil.

**$^{1}$H NMR (400 MHz, $CDCl_3$)** δ = 8.51 – 8.50 (m, labeled, 0.95H, <10%D), 7.97 – 7.92 (m, 2H), 7.46 (s, 1H), 7.15 – 7.09 (m, 2H), 7.02 – 7.01 (m, labeled, 0.7H, 30%D), 2.37 (s, 3H).

**$^{13}$C NMR (100 MHz, $CDCl_3$)** δ = 163.3 (d, $J_{C-F}$ = 246.0 Hz, 1C), 156.2, 149.3 (149.2) (1C), 147.7 (147.6) (1C), 135.6 (d, $J_{C-F}$ = 3.0 Hz, 1C), (128.6) 128.5 (1C), 123.0, 121.0, 115.4 (d, $J_{C-F}$ = 21.0 Hz, 1C), 21.1 (21.0) (1C).

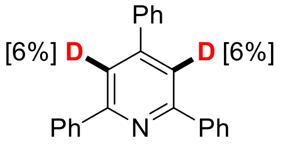

***2,4,6-Triphenylpyridine-3,5-d2 (75)***

The compounds were prepared following a general procedure and purified on silica gel (petroleum ethers ethyl acetate = 30:1) by column chromatography to obtain (91.7 mg, 99%) white solid.

**$^{1}$H NMR (400 MHz, $CDCl_3$)** δ = 8.25 (d, *J* = 7.6 Hz, 4H), 7.92 (s, labeled, 1.88H, 6%D), 7.78 (d, *J* = 7.4 Hz, 2H), 7.58 – 7.54 (m, 6H), 7.52 – 7.47 (m, 3H).

**$^{13}$C NMR (100 MHz, $CDCl_3$)** δ = 157.4, 150.1, 139.5, 139.0, 129.1 – 128.9 (m, labeled, 1C), 128.7, (127.14) 127.09 (1C), 117.1.

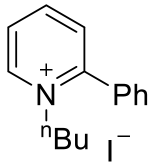

***N-Butyl-2-phenylpyridinium iodide (S1-a)***

Yellow solid.

**$^{1}$H NMR (400 MHz, $D_2O$)** δ = 8.93 – 8.92 (m, 1H), 8.56 – 8.51 (m, 1H), 8.08 – 8.04 (m, 1H), 7.96 – 7.94 (m, 1H), 7.71 – 7.60 (m, 5H), 4.52 – 4.48 (m, 2H), 1.82 – 1.74 (m, 2H), 1.22 – 1.12 (m, 2H), 0.72 (t, *J* = 7.4 Hz, 3H).

**$^{13}$C NMR (100 MHz, $D_2O$)** δ = 155.6, 145.3, 145.0, 131.5, 131.2, 130.6, 129.2, 128.9, 127.1, 58.5, 32.4, 18.8, 12.5.

**HRMS** (**ESI, m/z**): calculated for $C_{15}H_{18}N$ $[M]^+$: 212.1434, found: 212.1436.

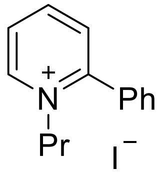

***N-Propyl-2-phenylpyridinium iodide (S1-b)***

Yellow solid.

**$^{1}$H NMR (400 MHz, $D_2O$)** δ = 8.91 – 8.89 (m, 1H), 8.54 – 8.49 (m, 1H), 8.06 – 8.02 (m, 1H), 7.96 – 7.93 (m, 1H), 7.70 – 7.61 (m, 3H), 7.60 – 7.57 (m, 2H), 4.47 – 4.43 (m, 2H), 1.85 – 1.75 (m, 2H), 0.75 (t, *J* = 7.4 Hz, 3H).

**$^{13}$C NMR (100 MHz, $D_2O$)** δ = 155.6, 145.2, 144.9, 131.5, 131.2, 130.6, 129.2, 128.8, 127.0, 60.1, 23.9, 9.8.

**HRMS** (**ESI, m/z**): calculated for $C_{14}H_{16}N$ $[M]^+$: 198.1277, found: 198.1273.

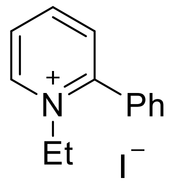

***N-Ethyl-2-phenylpyridinium-iodide (S1-c)***

Yellow solid.

**$^1$H NMR (400 MHz, D$_2$O)** δ = 8.95 (d, *J* = 6.4 Hz, 1H), 8.56 – 8.52 (m, 1H), 8.11 – 8.07 (m, 1H), 7.94 (d, *J* = 8.0 Hz, 1H), 7.71 – 7.61 (m, 5H), 4.52 (q, *J* = 7.6 Hz, 2H), 1.44 (t, *J* = 7.2 Hz, 3H).

**$^{13}$C NMR (100 MHz, D$_2$O)** δ = 155.5, 145.3, 144.6, 131.4, 131.3, 130.6, 129.3, 128.8, 127.3, 54.4, 15.7.

**HRMS** (**ESI, m**/**z**): calculated for $C_{13}H_{14}N$ $[M]^+$: 184.1121, found: 184.1127.

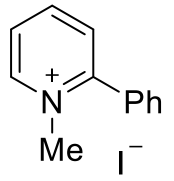

***N-Methyl-2-phenylpyridinium iodide (S1-d)***

Yellow solid.

**$^1$H NMR (400 MHz, D$_2$O)** δ = 8.88 – 8.86 (m, 1H), 8.58 – 8.53 (m, 1H), 8.07 – 8.03 (m, 1H), 7.98 – 7.95 (m, 1H), 7.69 – 7.61 (m, 5H), 4.17 (s, 3H).

**$^{13}$C NMR (100 MHz, D$_2$O)** δ = 155.7, 146.2, 145.5, 131.5, 131.4, 130.2, 129.3, 129.11, 129.07, 126.8, 47.3.

The analytical data corresponds with those reported in the literature.[6]

## 3.4 General Information of DFT Computational Studies

### 3.4.1 Details of Theoretical Calculation

For single-point energy (SPE) and natural population analysis (NPA) charge calculation of optimized geometries:

- Calculation software: Gaussian 16, Rev. A 03[7]
- DFT functional: M06-2X-D3 (Hybrid functional M06-2X[8] with dispersion-correction DFT-D3[9])
- Basis sets: def2-QZVP[10]
- Solvation model: SMD[11], an implicit solvation model. The solvent was chosen as DMF, with the keyword SCRF = (SMD, solvent = *N*,*N*-dimethylformamide).
- Other information: The keyword "Pop = NPA" was implemented to activate the calculation of NPA charges. The keyword "SCF = conver = 7" was implemented to loosen the convergence criteria of SCF iterations, with negligible loss of precision. Other settings were kept default.

For geometry optimization and frequency analysis:

- Calculation software: Gaussian 16, Rev. A 03[7]
- Basis sets: def2-TZVP[10]
- Solvation model: IEFPCM[12], an implicit solvation model. The solvent was chosen as DMF, with the keyword SCRF = (IEFPCM, solvent = *N*,*N* -dimethylformamide)
- Other information: Each intermediate does not contain any imaginary frequency. Most other settings, like the accuracy of integration grids, and criteria of convergence, were kept default.

The level of DFT computation can be noted as SMD(DMF) / M06-2X-D3 / def2-QZVP // IEFPCM(DMF) / M06-2X-D3 / def2-TZVP.

### 3.4.2 Computed NPA Charges of Stationary Points

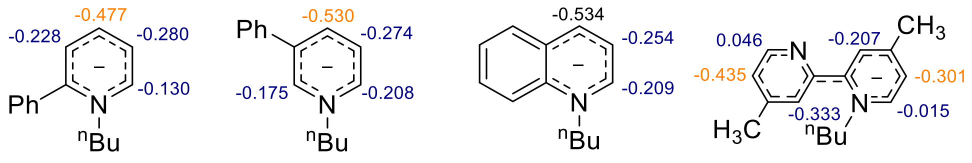


**Supplementary Figure 15.** The NPA charges of aromatic C atoms in the N-alkylated closed-shell anion intermediates (**1-VII, 18-VII, 29-VII, 46-VII**).

### 3.4.3 Cartesian Coordinates of Stationary Points (Unit: Å)

**1-VII**

Charge = -1 Spin Multiplicity = 1

| | | | |
|---|---|---|---|
| C | 2.97779620 | -0.18141501 | 1.30535521 |
| C | 1.63607419 | -0.46891161 | 1.14154368 |
| C | 0.87656921 | 0.07972188 | 0.07594586 |
| C | 1.57822627 | 0.94683402 | -0.79701093 |
| C | 2.92012261 | 1.24079344 | -0.61358914 |
| C | 3.64555754 | 0.67997857 | 0.43334636 |
| H | 3.51351878 | -0.62601373 | 2.13670889 |
| H | 1.15427301 | -1.11725588 | 1.86233031 |
| H | 1.03640094 | 1.38594025 | -1.62449113 |
| H | 3.41119338 | 1.91499244 | -1.30683093 |
| H | 4.69447496 | 0.90696846 | 0.57063465 |
| C | -0.51024047 | -0.23361747 | -0.14045514 |
| C | -1.23675464 | -1.18925365 | 0.56785372 |
| C | -2.62215488 | -1.26049345 | 0.47110793 |
| H | -0.72278793 | -1.84750005 | 1.26269060 |
| C | -2.53711241 | 0.71820322 | -0.91153356 |
| C | -3.26295564 | -0.15436819 | -0.17410188 |
| H | -3.19061809 | -1.98242487 | 1.04087801 |
| H | -2.95217878 | 1.64292309 | -1.29832028 |
| H | -4.31216927 | 0.05315473 | 0.01657175 |
| N | -1.17508826 | 0.40517327 | -1.26268345 |
| C | -1.08917132 | -0.40691002 | -2.48331578 |

| | | | |
|---|---|---|---|
| H | -0.04795263 | -0.73082068 | -2.58213209 |
| H | -1.70824348 | -1.31423244 | -2.39300322 |
| C | -1.49117068 | 0.36595477 | -3.72944422 |
| H | -2.51655969 | 0.73203858 | -3.62101065 |
| H | -0.84901682 | 1.24890411 | -3.81888105 |
| C | -1.39013273 | -0.47283473 | -4.99765834 |
| H | -0.36840118 | -0.85104826 | -5.10015291 |
| H | -2.03498804 | -1.35127326 | -4.90093665 |
| C | -1.77419443 | 0.30832877 | -6.24829039 |
| H | -1.69844138 | -0.30583923 | -7.14642667 |
| H | -2.80090575 | 0.67313673 | -6.17630064 |
| H | -1.12318419 | 1.17530981 | -6.37831482 |

**18-VII**

Charge = -1 Spin Multiplicity = 1

| | | | |
|---|---|---|---|
| C | -1.46902752 | 1.68191926 | 0.19784243 |
| C | -0.46526354 | 2.37456314 | -0.41417152 |
| C | 0.88455885 | 1.96083740 | -0.28916579 |
| H | -0.71982008 | 3.18300283 | -1.09612499 |
| C | 0.01330263 | -0.02952101 | 0.76518946 |
| C | 1.06148996 | 0.62166534 | 0.16654772 |
| H | 1.67045435 | 2.46121615 | -0.83889117 |
| H | 0.03686562 | -1.08709105 | 1.00417119 |
| N | -1.13115419 | 0.71978925 | 1.22611609 |
| H | -2.51917405 | 1.84515921 | -0.01622994 |
| C | 2.32979533 | -0.09446648 | -0.11346466 |
| C | 2.35708738 | -1.45655893 | -0.43420427 |
| C | 3.55382314 | 0.58113325 | -0.05995555 |
| C | 3.55381925 | -2.11442161 | -0.68219376 |
| H | 1.42383696 | -2.00074462 | -0.50570472 |
| C | 4.75246759 | -0.07485706 | -0.30633389 |

| | | | |
|---|---|---|---|
| H | 3.56043238 | 1.63412991 | 0.19350394 |
| C | 4.76111881 | -1.42863745 | -0.61857355 |
| H | 3.54246859 | -3.16776862 | -0.93458262 |
| H | 5.68471076 | 0.47391380 | -0.25052693 |
| H | 5.69434945 | -1.94151211 | -0.81265206 |
| C | -0.87581475 | 1.36214570 | 2.52400694 |
| H | -0.45729127 | 0.59574296 | 3.18501225 |
| H | -0.12029254 | 2.15773143 | 2.42571524 |
| C | -2.13866430 | 1.92793452 | 3.15371081 |
| H | -2.86785953 | 1.11894157 | 3.27071467 |
| H | -2.58789791 | 2.66113311 | 2.47746046 |
| C | -1.88033487 | 2.58337880 | 4.50505879 |
| H | -1.41713143 | 1.85504313 | 5.17780793 |
| H | -1.15399243 | 3.39208540 | 4.38099945 |
| C | -3.15136821 | 3.13126213 | 5.14259909 |
| H | -3.88119954 | 2.33386253 | 5.29754483 |
| H | -2.95078700 | 3.59491444 | 6.10931139 |
| H | -3.61397853 | 3.88255739 | 4.49915061 |

**29-VII**

Charge = -1    Spin Multiplicity = 1

| | | | |
|---|---|---|---|
| C | 1.95143808 | -2.51664399 | -0.28201513 |
| C | 0.89777580 | -1.70846370 | 0.15229463 |
| C | 1.02970261 | -0.33507820 | 0.24824563 |
| C | 2.22785154 | 0.31970477 | -0.18124829 |
| C | 3.29224945 | -0.52604261 | -0.58716609 |
| C | 3.15545458 | -1.90219728 | -0.63066616 |
| H | 1.84258792 | -3.59243777 | -0.32193269 |
| H | -0.04022292 | -2.15188433 | 0.47119226 |
| C | 2.23960503 | 1.73323477 | -0.25159980 |
| H | 4.23136587 | -0.06952424 | -0.88402965 |

| | | | |
|---|---|---|---|
| H | 3.99626529 | -2.50883522 | -0.94935200 |
| C | 0.97444384 | 2.37574753 | -0.18396158 |
| C | -0.13623608 | 1.73441835 | 0.26426230 |
| H | 3.10112761 | 2.24517918 | -0.66165720 |
| H | 0.86633493 | 3.38361502 | -0.57788772 |
| H | -1.13105945 | 2.15794604 | 0.21042670 |
| N | -0.00381985 | 0.43693438 | 0.89131053 |
| C | 0.24167743 | 0.55504505 | 2.33915314 |
| H | 0.52252370 | -0.43607056 | 2.70760878 |
| H | 1.08720213 | 1.23369244 | 2.53406282 |
| C | -0.98476915 | 1.03795360 | 3.09600974 |
| H | -1.80678669 | 0.33611783 | 2.92035705 |
| H | -1.30342350 | 2.00544206 | 2.69760754 |
| C | -0.73191020 | 1.17002780 | 4.59306676 |
| H | -0.39844091 | 0.20661458 | 4.99056991 |
| H | 0.09018292 | 1.87245126 | 4.75897411 |
| C | -1.96594905 | 1.63814294 | 5.35446135 |
| H | -2.79149440 | 0.93555106 | 5.22349380 |
| H | -1.76919084 | 1.72936302 | 6.42332076 |
| H | -2.29892358 | 2.61204749 | 4.98977628 |

**46-VII**

Charge = -1 Spin Multiplicity = 1

| | | | |
|---|---|---|---|
| C | -0.27593866 | 0.54032740 | 0.24489917 |
| C | -1.50630899 | 0.18310738 | -0.37842979 |
| C | -2.71252443 | 0.46877387 | 0.18483522 |
| C | -2.68461994 | 1.09200255 | 1.49886388 |
| C | -1.54263004 | 1.59208163 | 1.99697933 |
| H | -1.46338305 | -0.38370053 | -1.30390585 |
| H | -3.58743474 | 1.12289436 | 2.10044561 |
| H | -1.50265839 | 2.07516667 | 2.96996766 |

| | | | |
|---|---|---|---|
| C | 0.96113292 | -0.02864151 | -0.07479080 |
| C | 2.16277312 | 0.31571981 | 0.64115629 |
| C | 3.37141987 | -0.23732131 | 0.31058456 |
| H | 2.09321166 | 1.01083724 | 1.46798281 |
| C | 2.23657445 | -1.43220307 | -1.41665400 |
| C | 3.42911609 | -1.14444385 | -0.76969832 |
| H | 2.24752742 | -2.13460680 | -2.24939158 |
| H | 4.35980794 | -1.60024217 | -1.08080239 |
| N | -0.32552692 | 1.56401727 | 1.27743831 |
| N | 1.04757027 | -0.93241209 | -1.11467052 |
| C | -4.02913427 | 0.10751099 | -0.43788993 |
| H | -4.62616134 | 0.99075183 | -0.68882738 |
| H | -3.88038260 | -0.46139099 | -1.35698193 |
| H | -4.64123994 | -0.49810261 | 0.23871425 |
| C | 4.62235019 | 0.10508160 | 1.07120012 |
| H | 5.37380816 | 0.53548722 | 0.40550790 |
| H | 4.41994500 | 0.81852035 | 1.86941419 |
| H | 5.06375325 | -0.79131482 | 1.51242259 |
| C | 0.11581828 | 2.89772623 | 0.85872515 |
| H | 0.10811277 | 3.54067859 | 1.74704206 |
| H | 1.15432075 | 2.82304156 | 0.52286483 |
| C | -0.72466402 | 3.54237707 | -0.23957257 |
| H | -1.76721703 | 3.59364868 | 0.09133300 |
| H | -0.70208063 | 2.89779384 | -1.12312184 |
| C | -0.23130337 | 4.93649835 | -0.60613284 |
| H | 0.81506017 | 4.87754809 | -0.92155859 |
| H | -0.24834040 | 5.57284779 | 0.28412701 |
| C | -1.06182863 | 5.58009508 | -1.70982161 |
| H | -0.69657787 | 6.57661964 | -1.96144433 |
| H | -1.03515680 | 4.97300742 | -2.61700102 |
| H | -2.10599160 | 5.67202899 | -1.40373281 |

## 3.5 NMR spectra

### $^{1}$H NMR and $^{13}$C NMR of 1

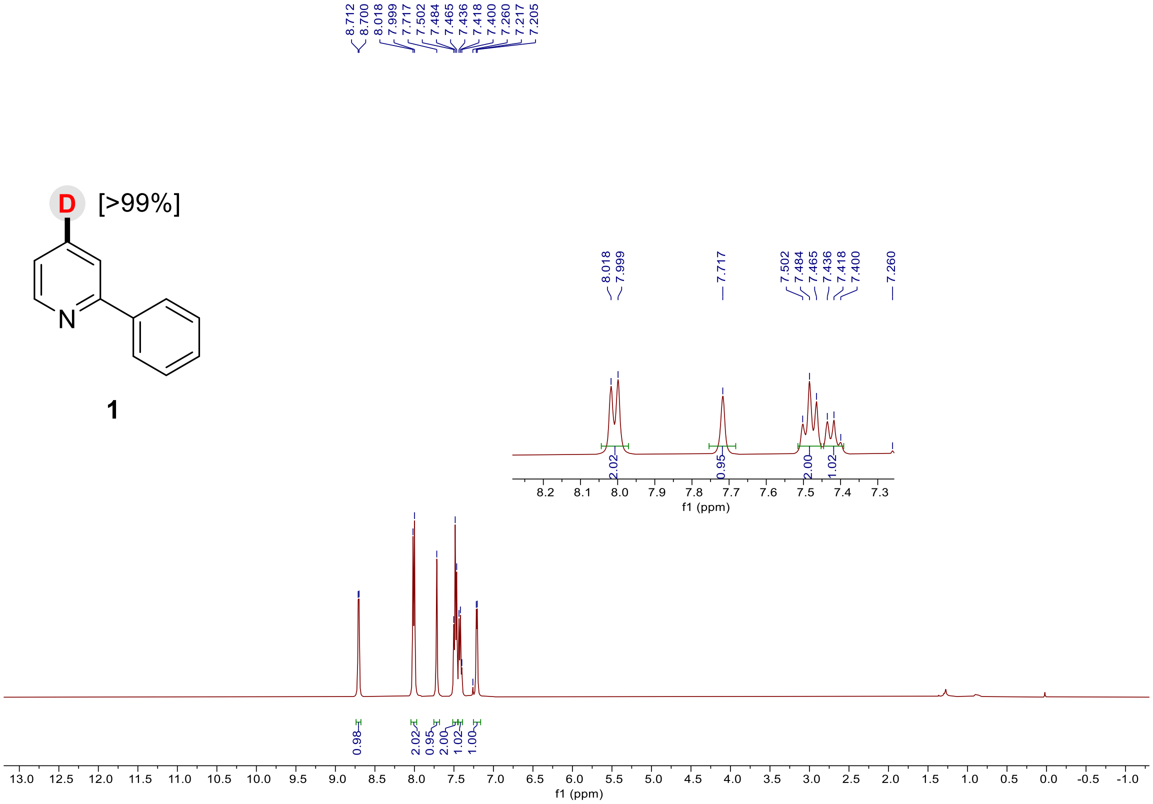

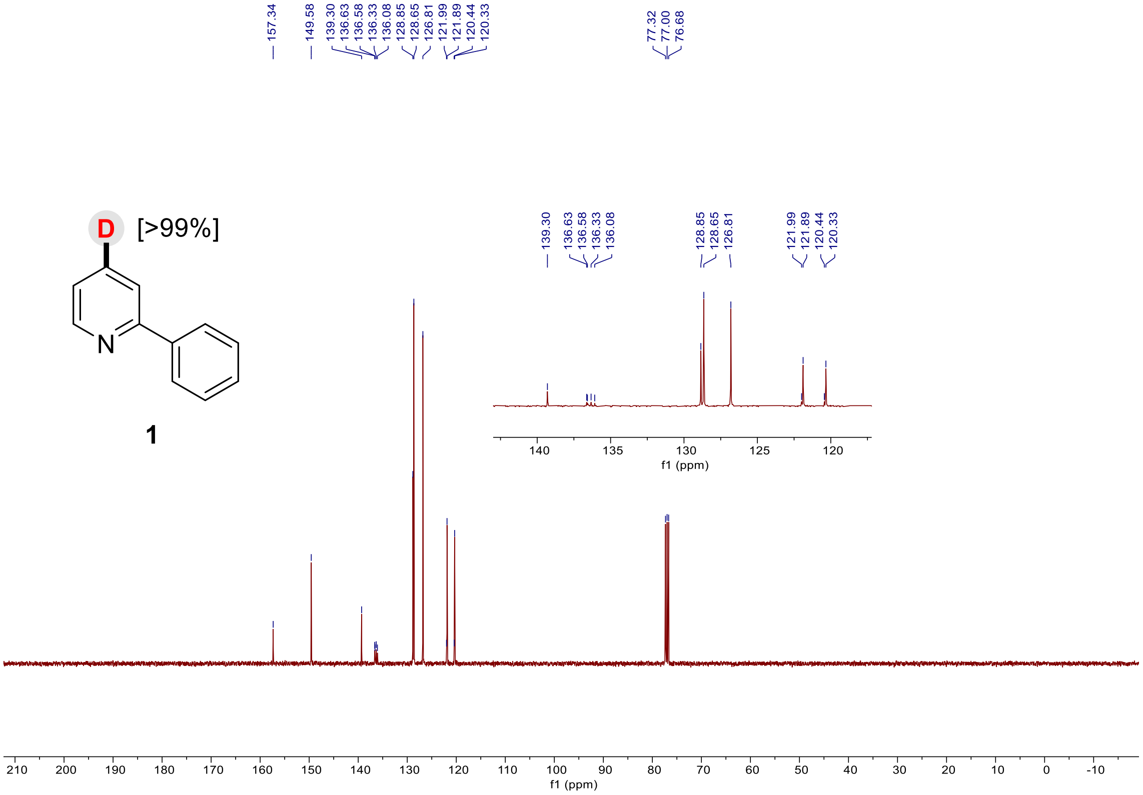

$^{1}H$ NMR and $^{13}C$ NMR of **2**

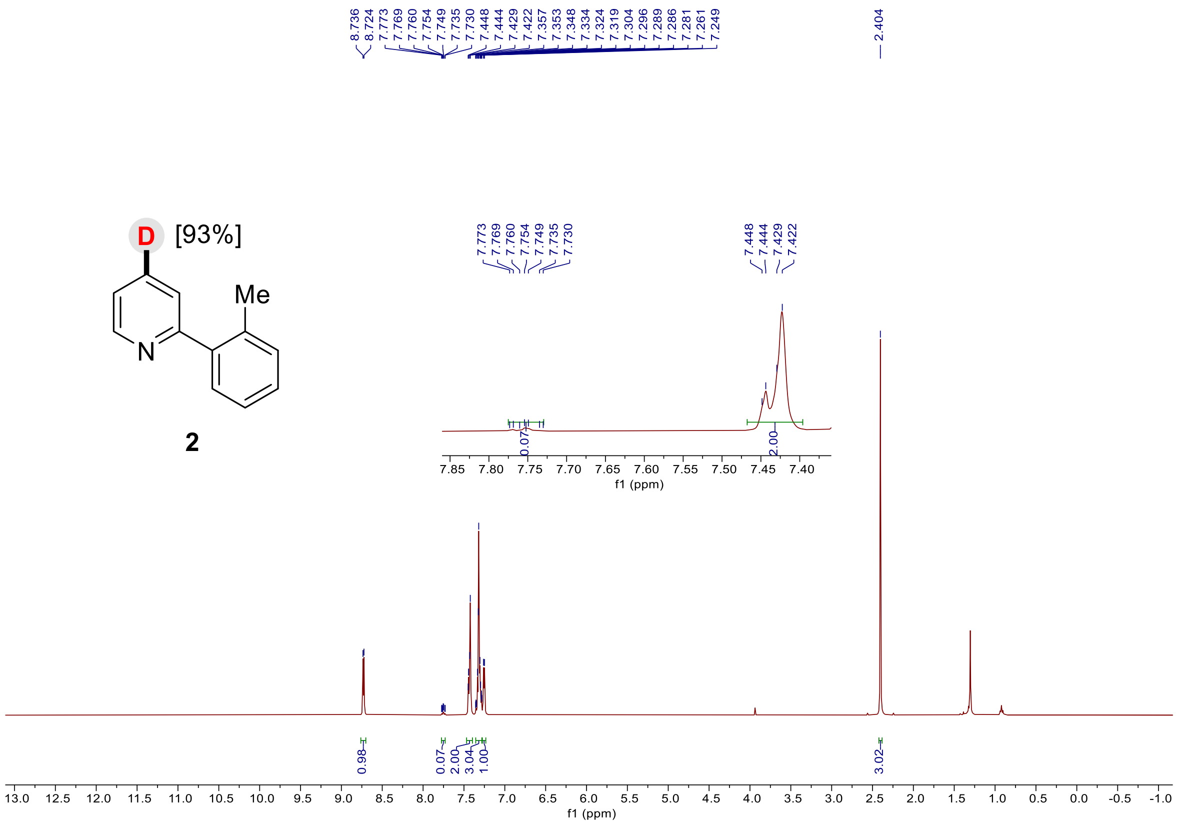

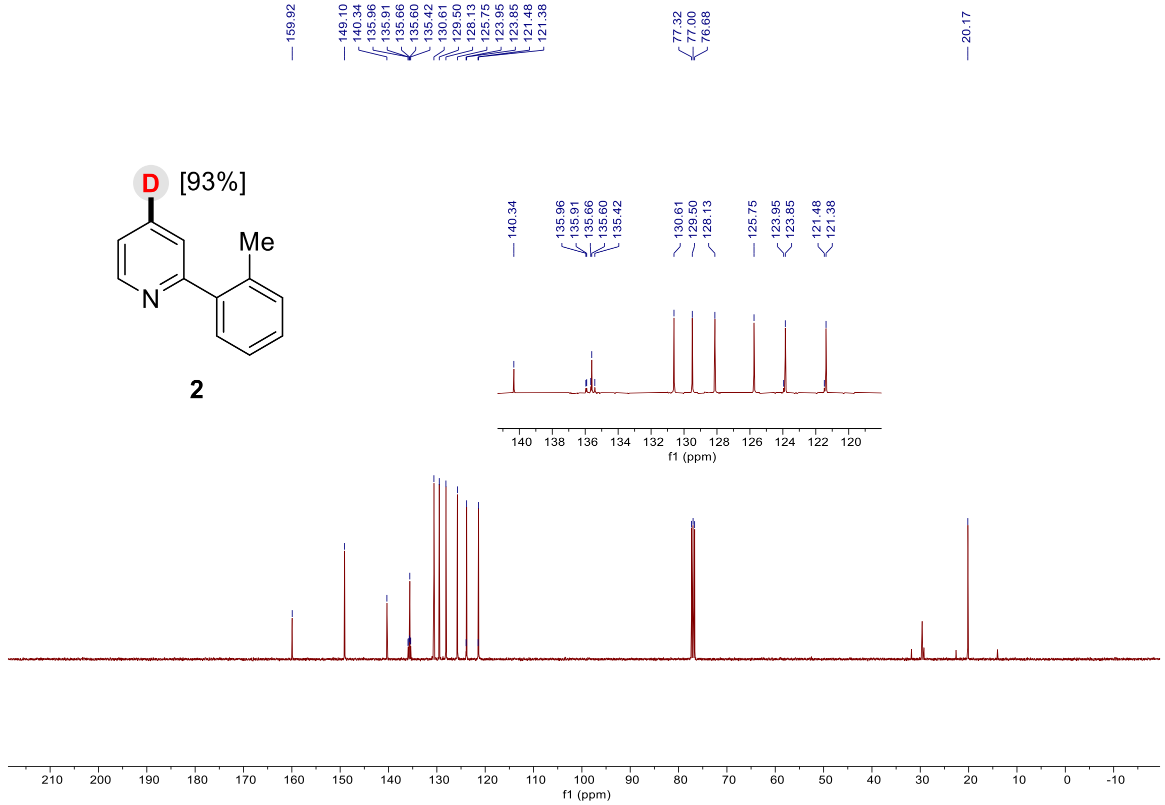

**$^{1}$H NMR** and **$^{13}$C NMR** of **3**

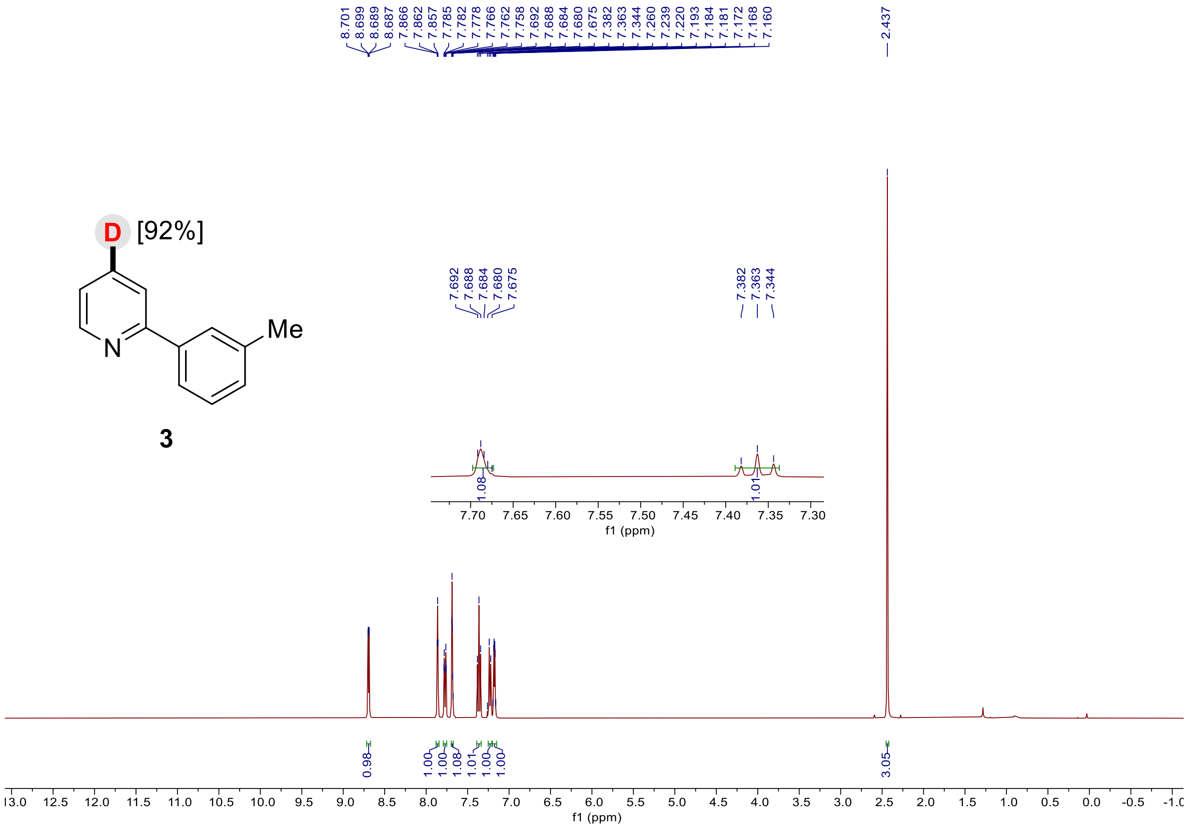


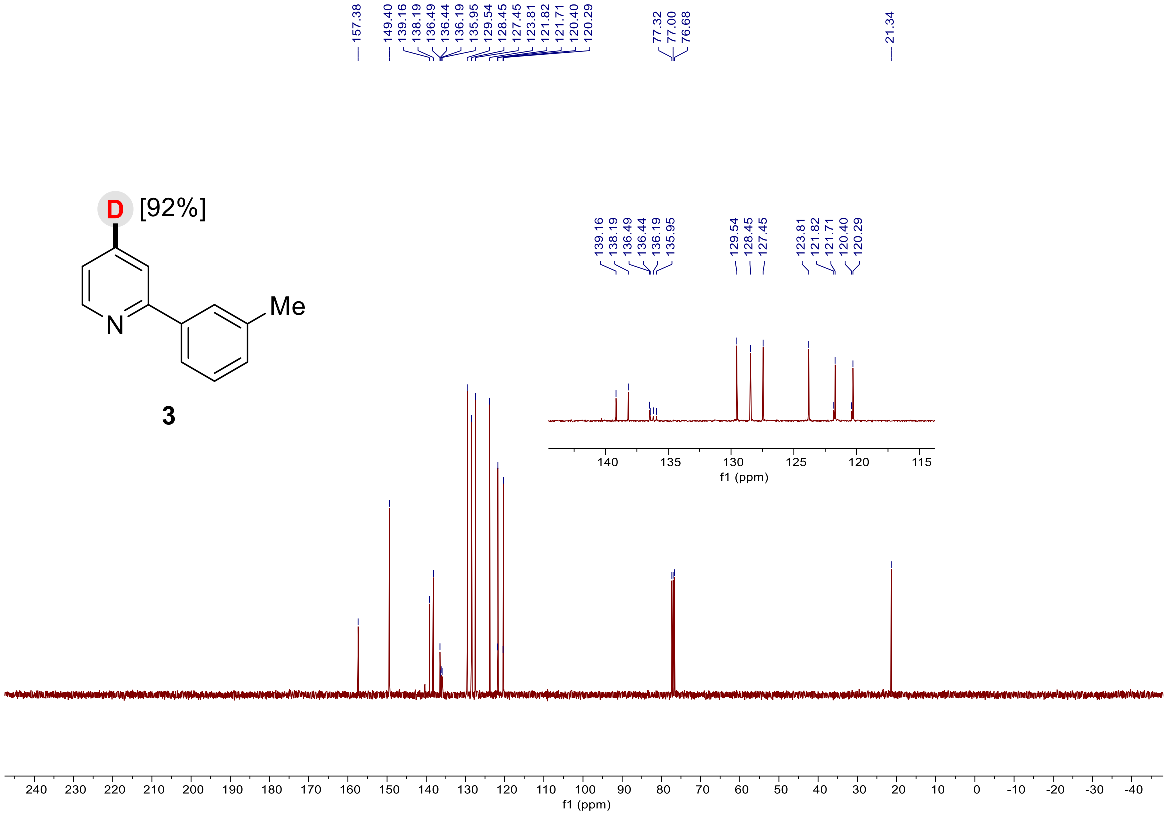

$^{1}H$ NMR and $^{13}C$ NMR of 4

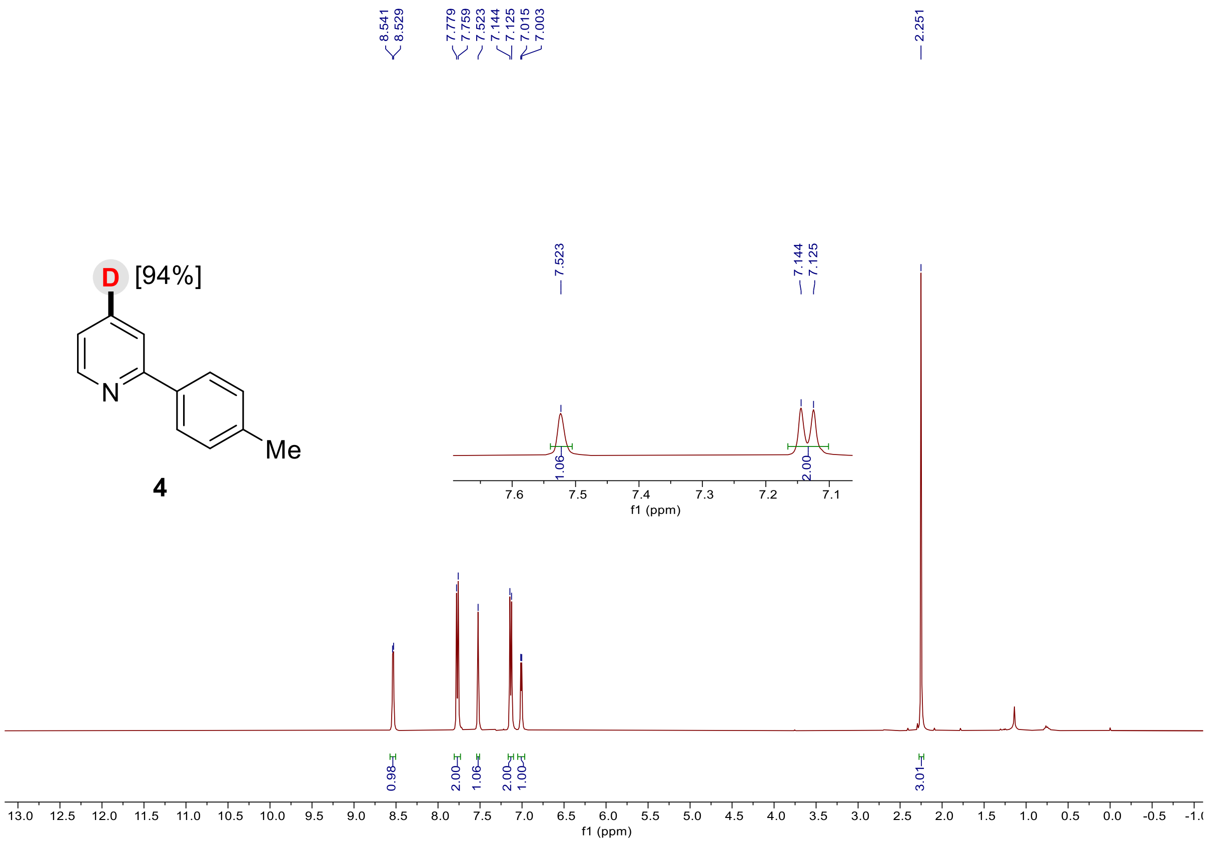


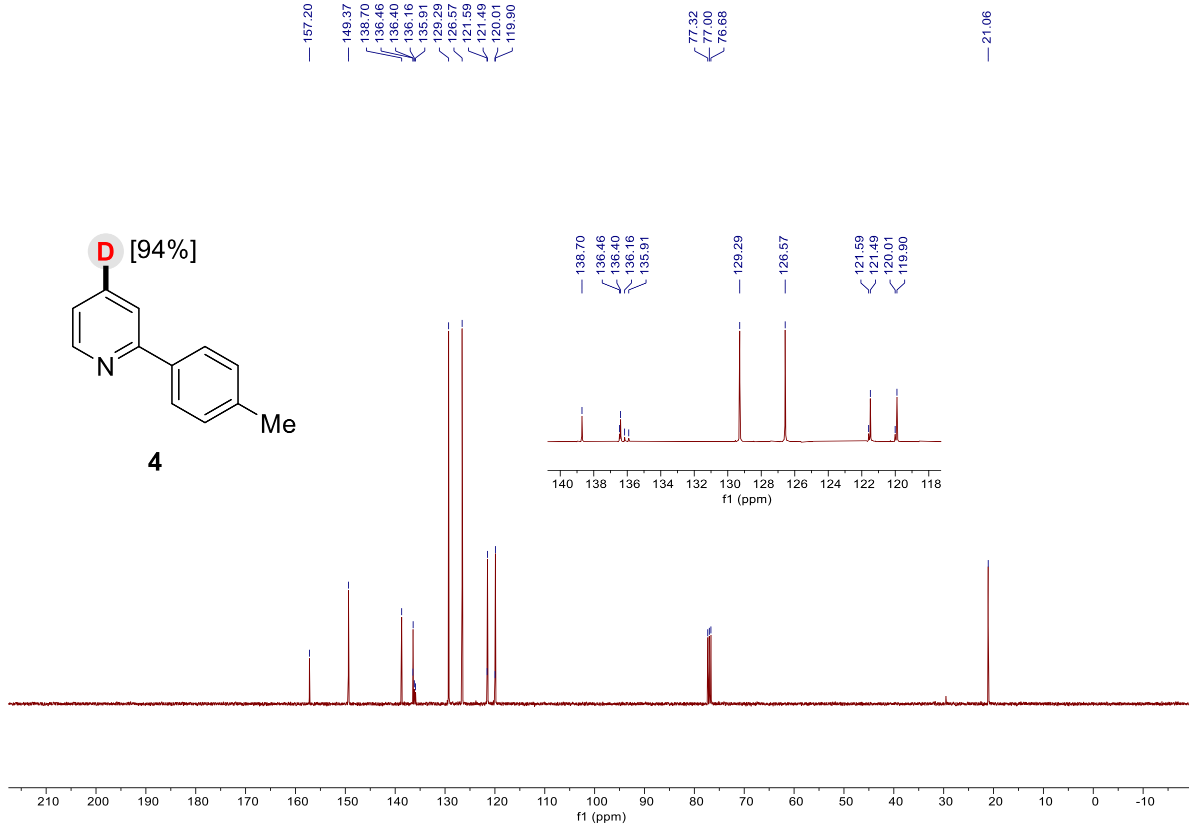

$^{1}$H NMR and $^{13}$C NMR of 5

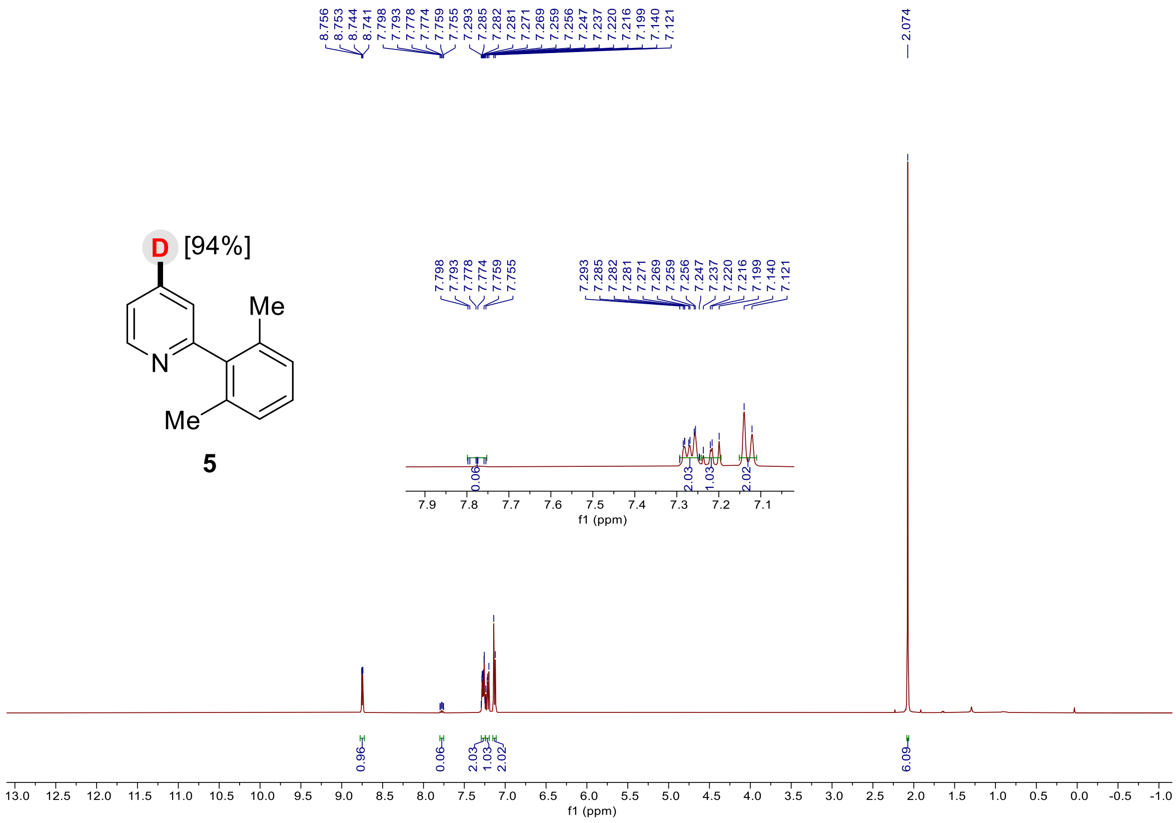

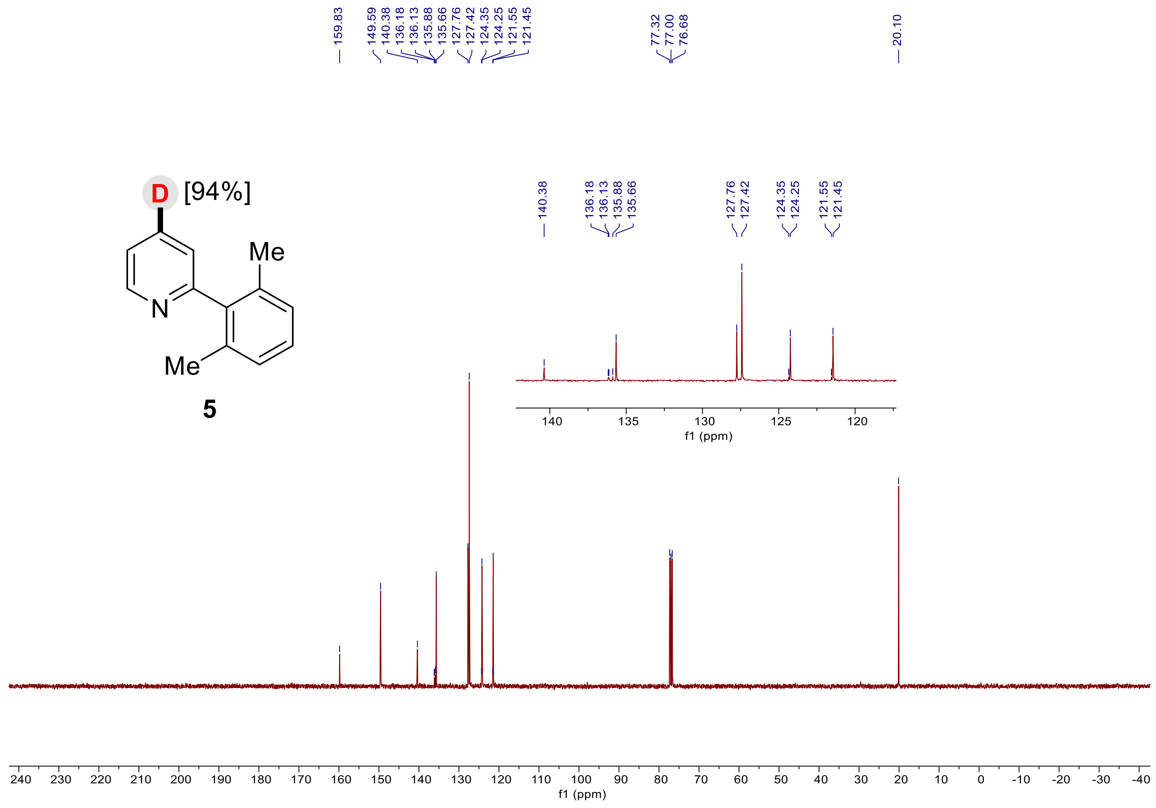

**$^{1}$H NMR** and **$^{13}$C NMR** of **6**

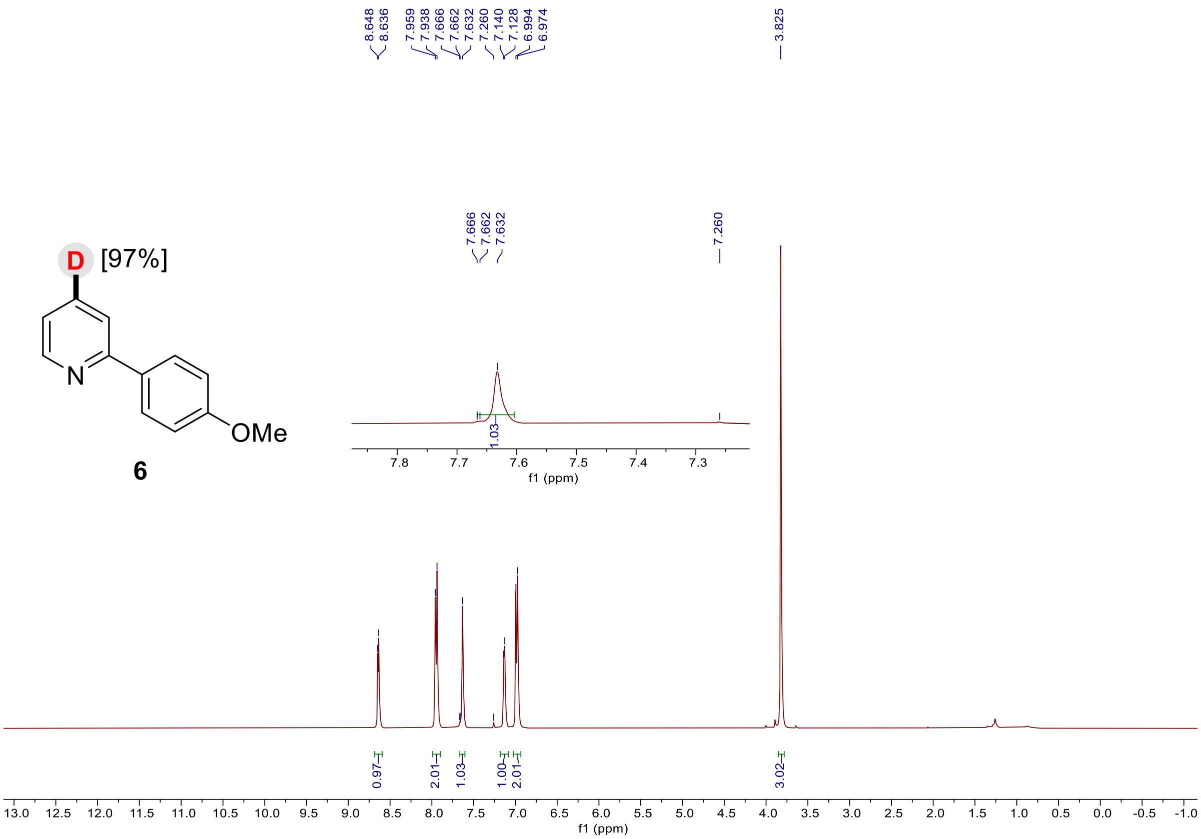


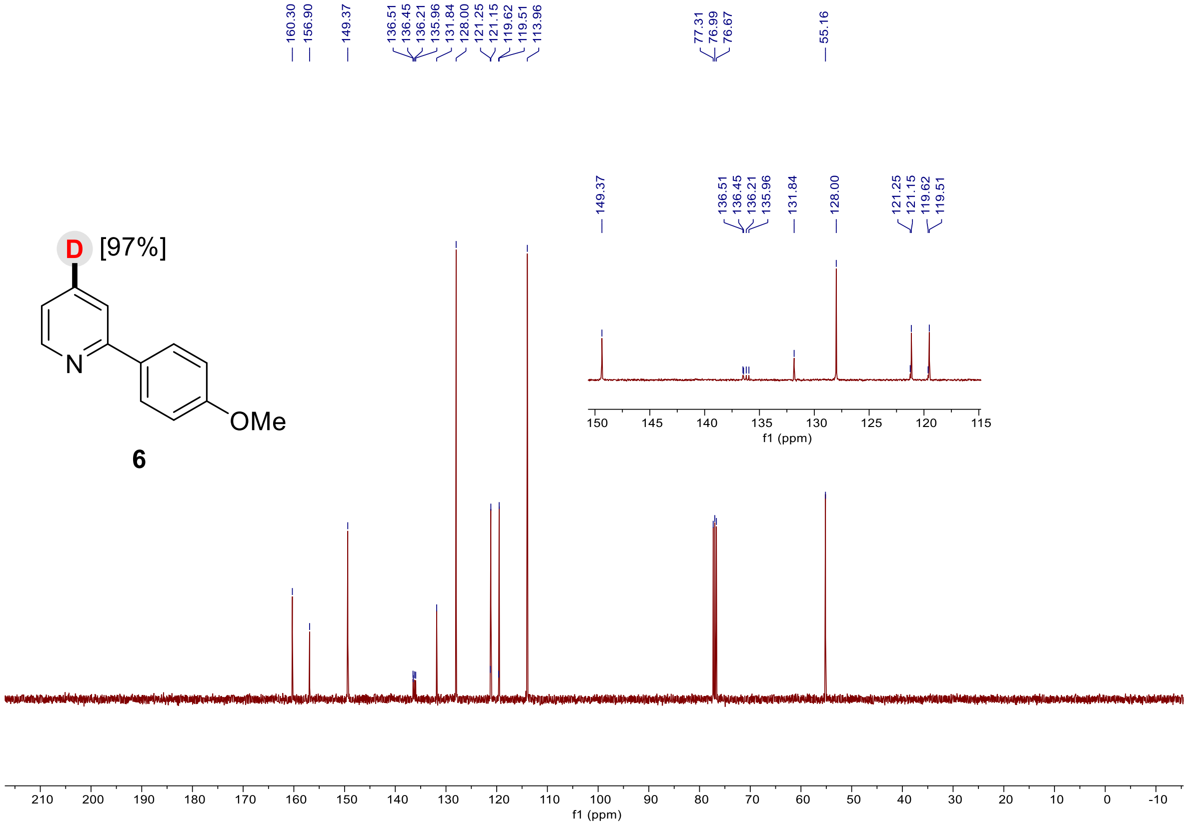

**$^{1}$H NMR** and **$^{13}$C NMR** of **7**

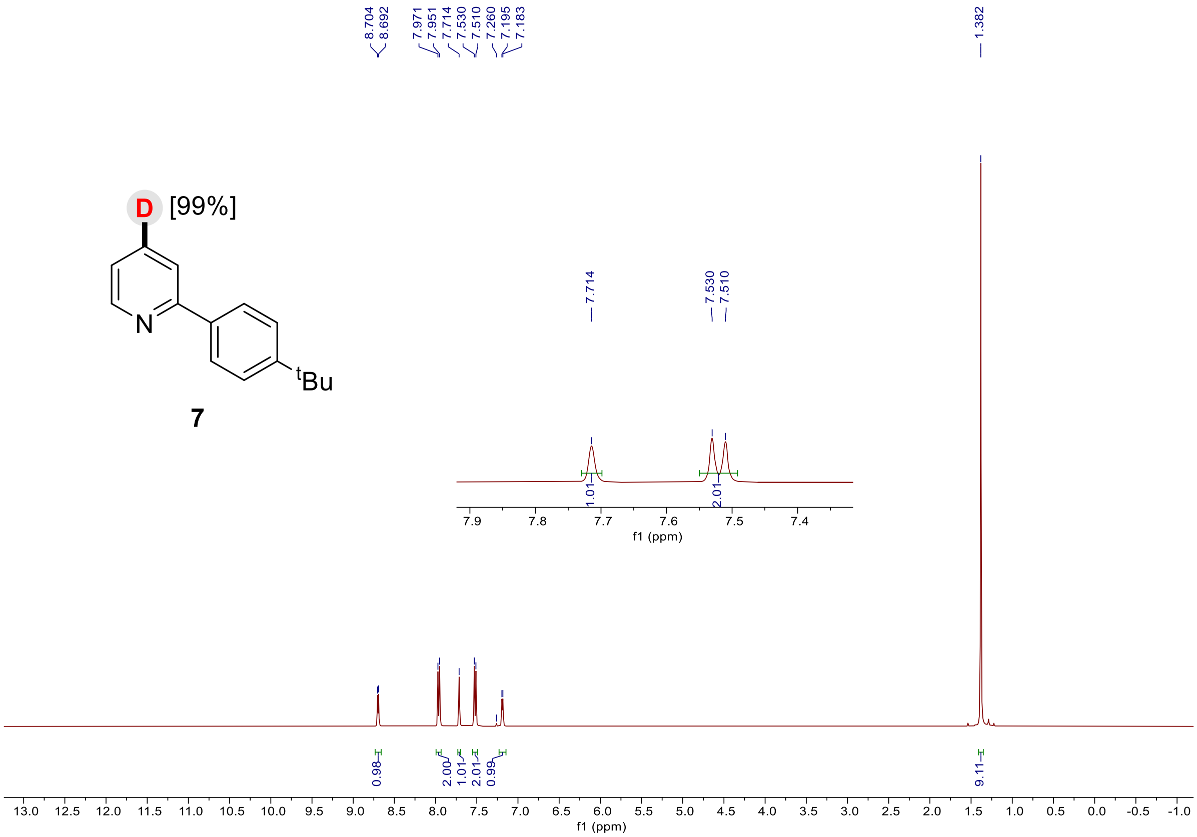


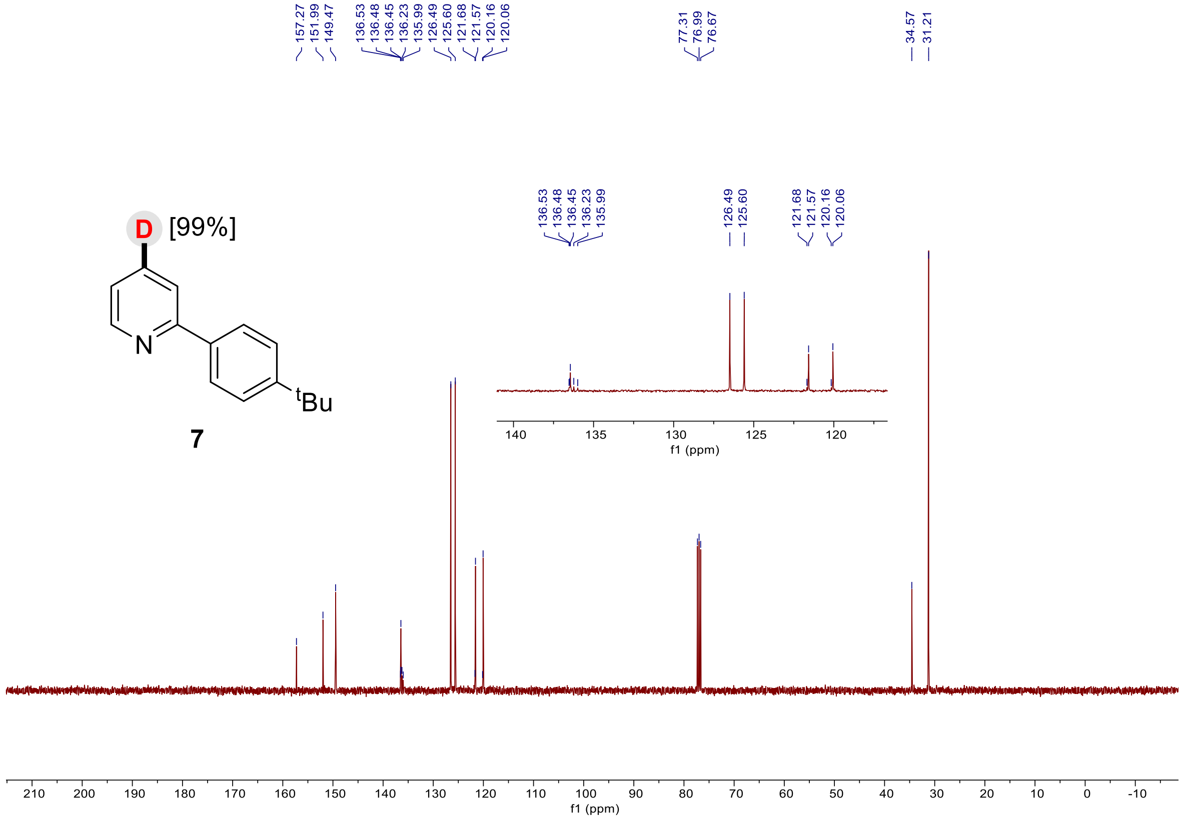

**$^{1}$H NMR and $^{13}$C NMR of 8**

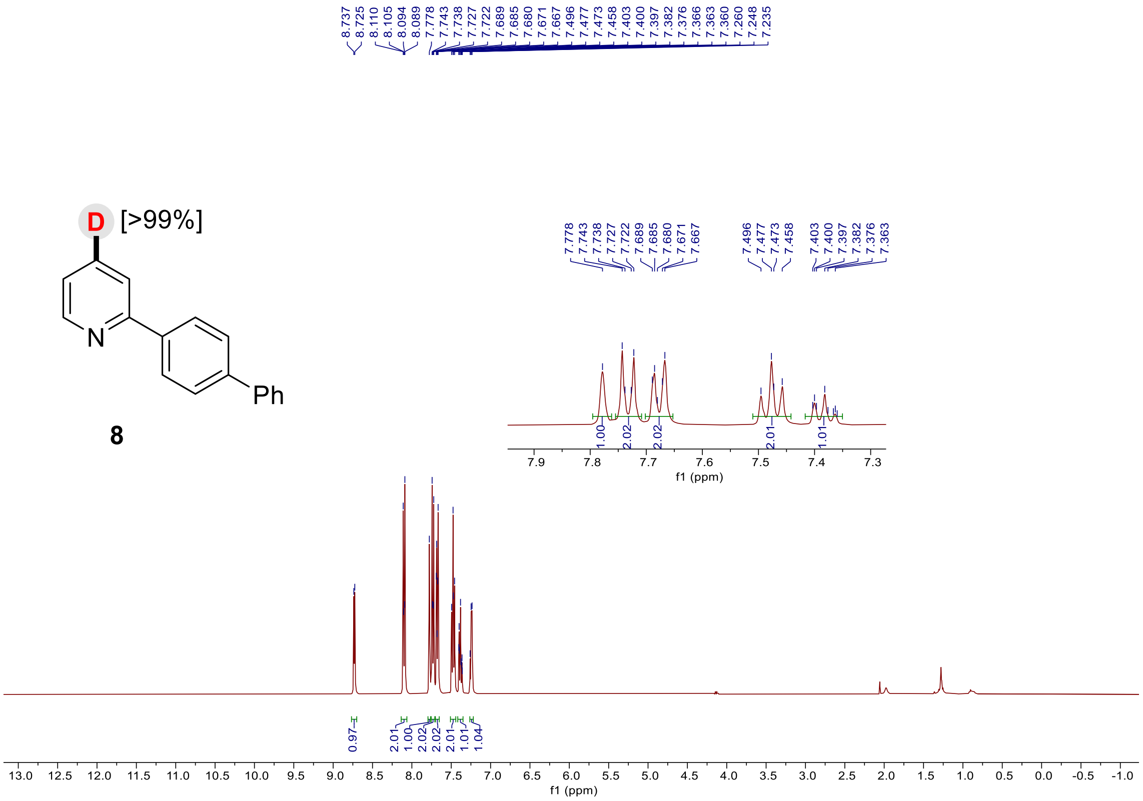


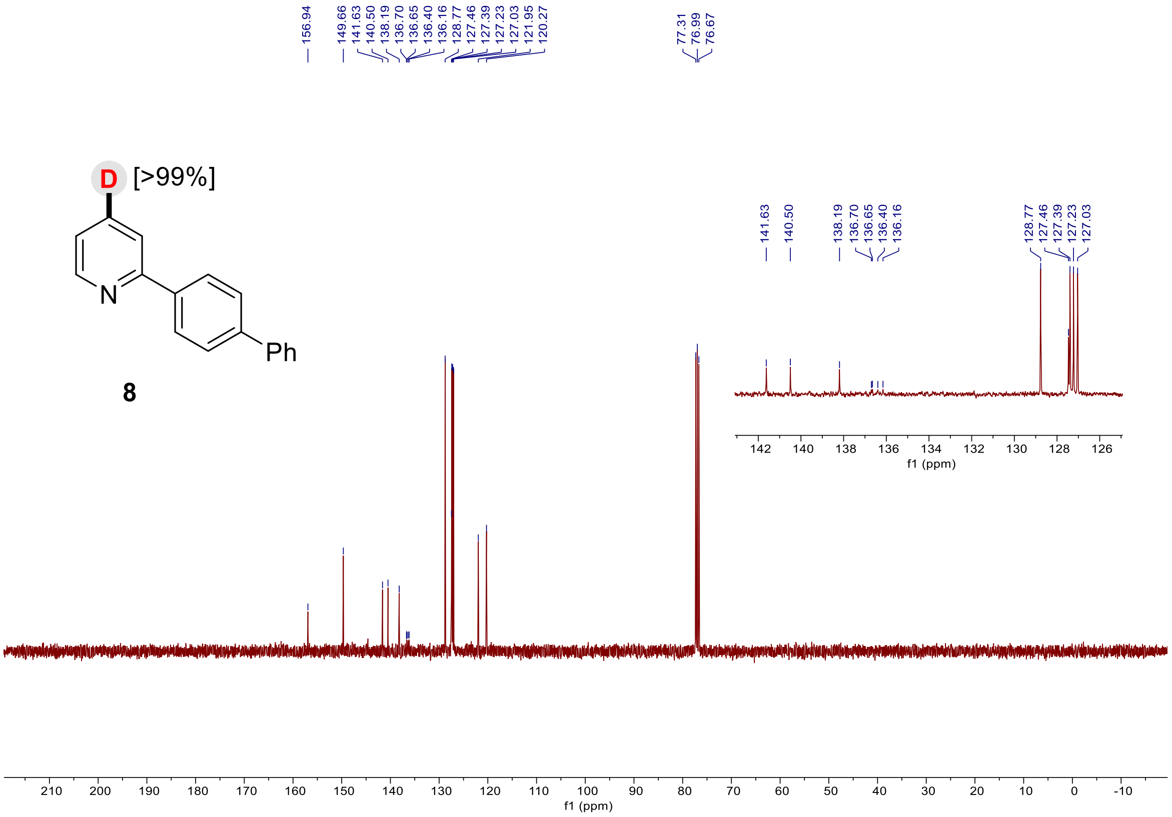

**$^{1}$H NMR** and **$^{13}$C NMR** of **9**

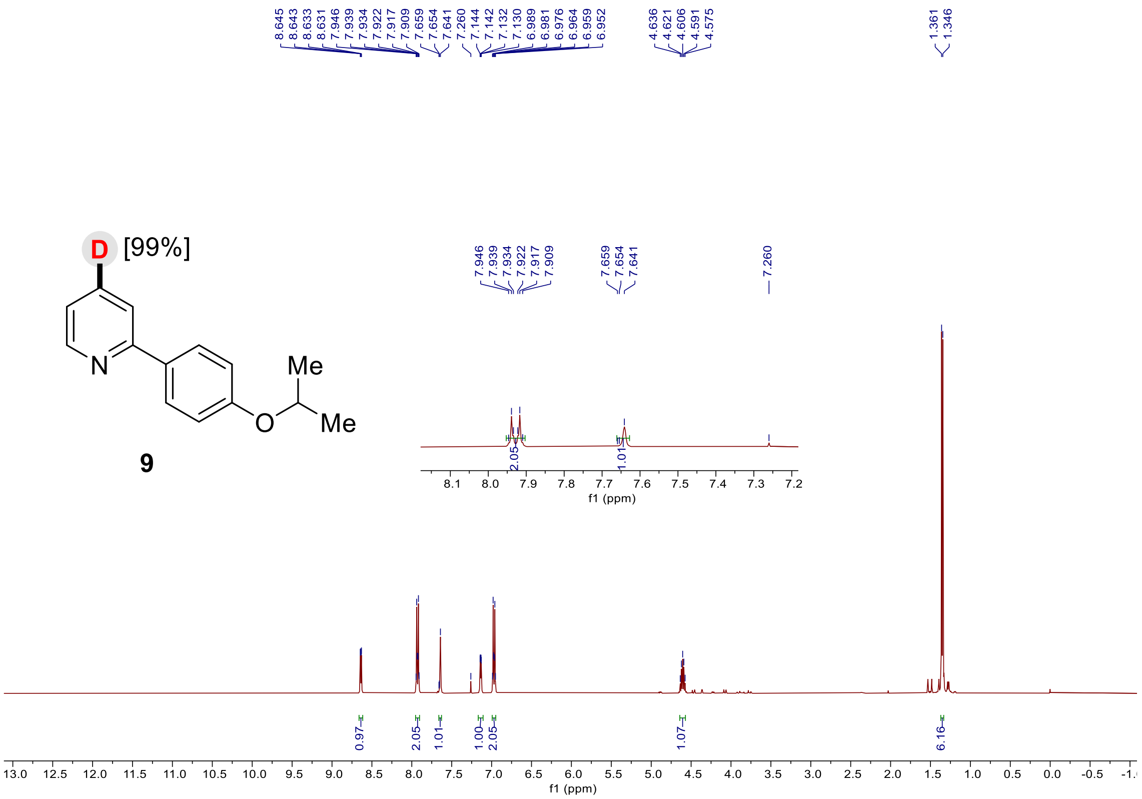

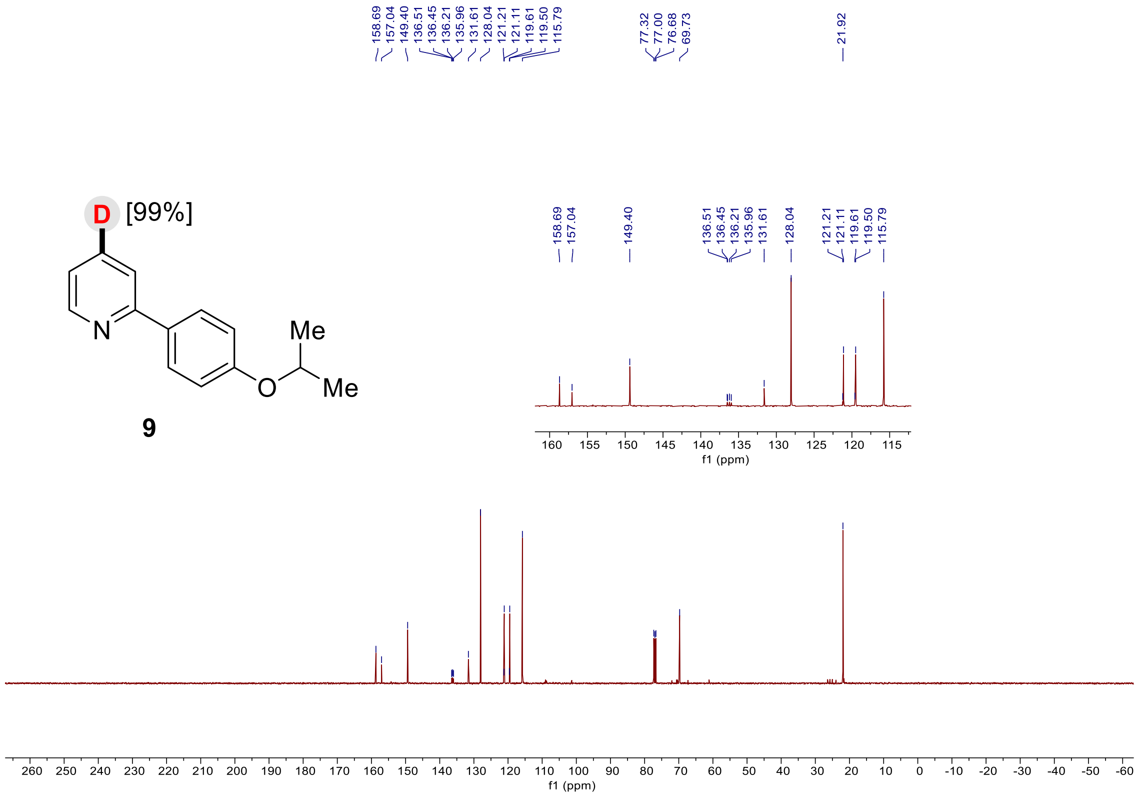

$^{1}$H NMR and $^{13}$C NMR of 10

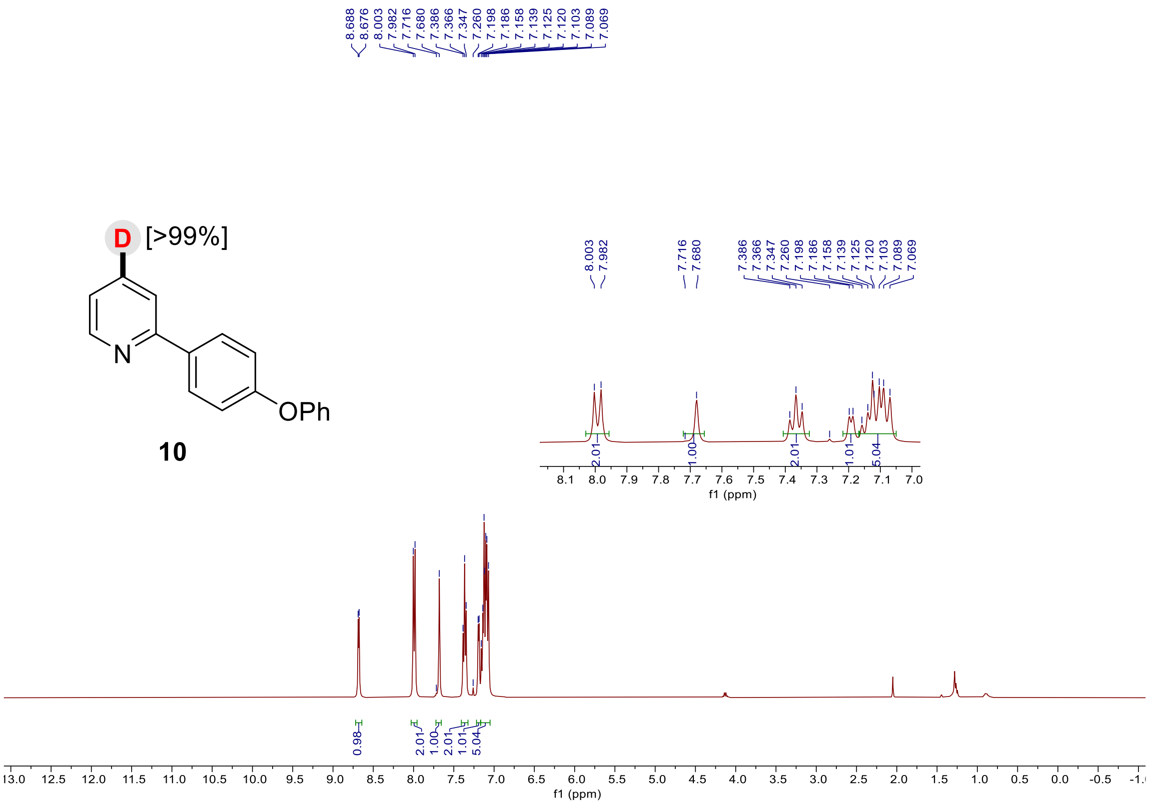


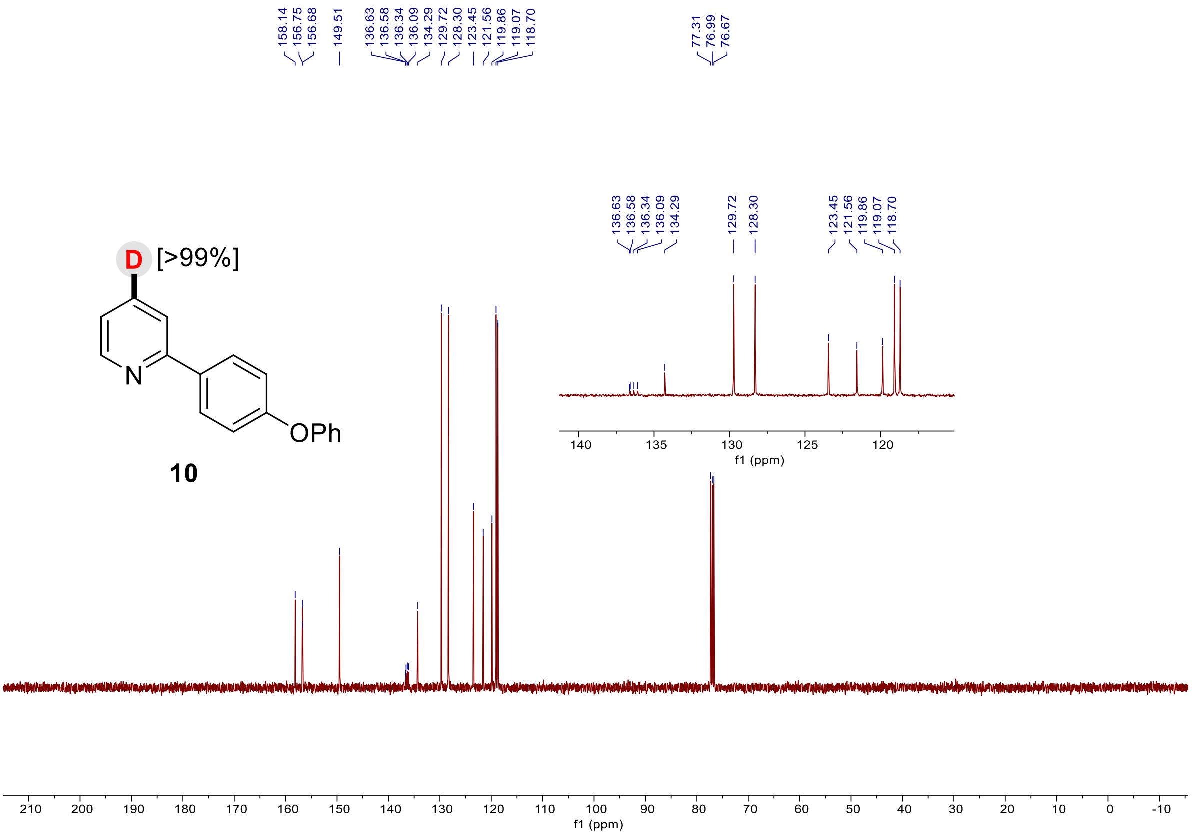

$^{1}$H NMR and $^{13}$C NMR of **11**

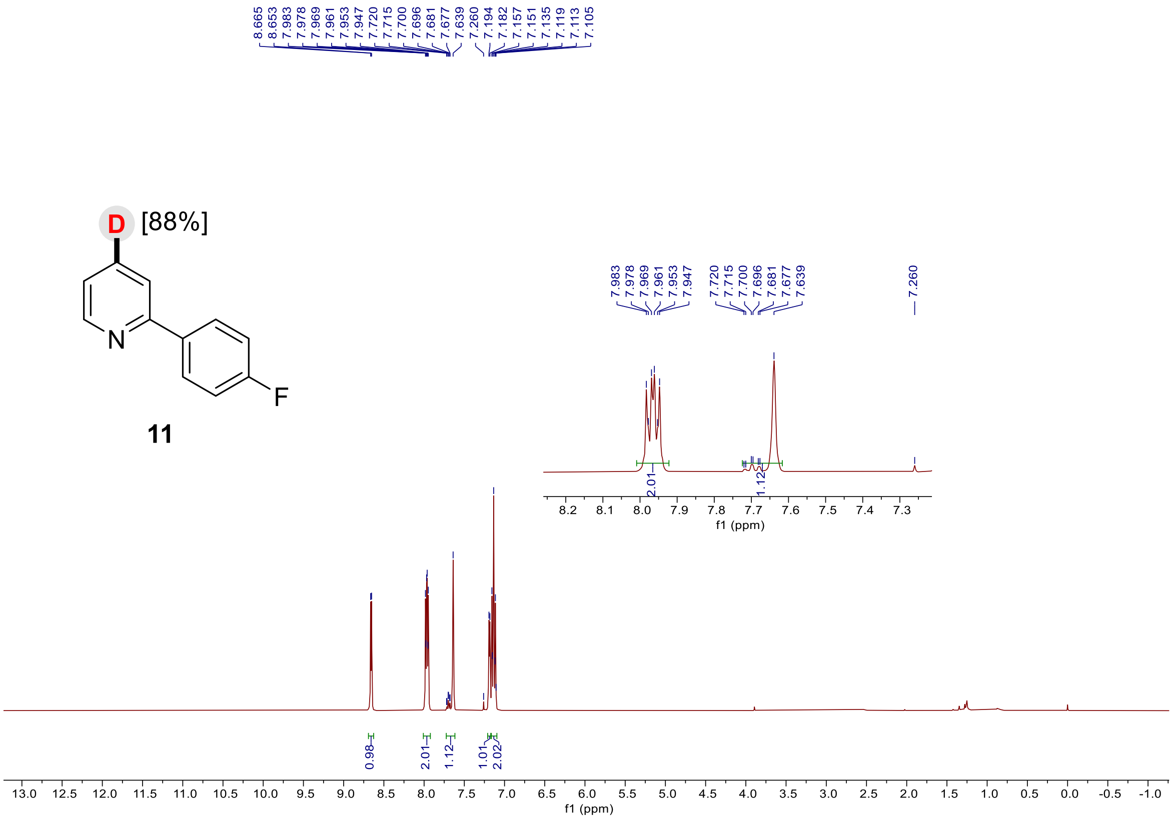


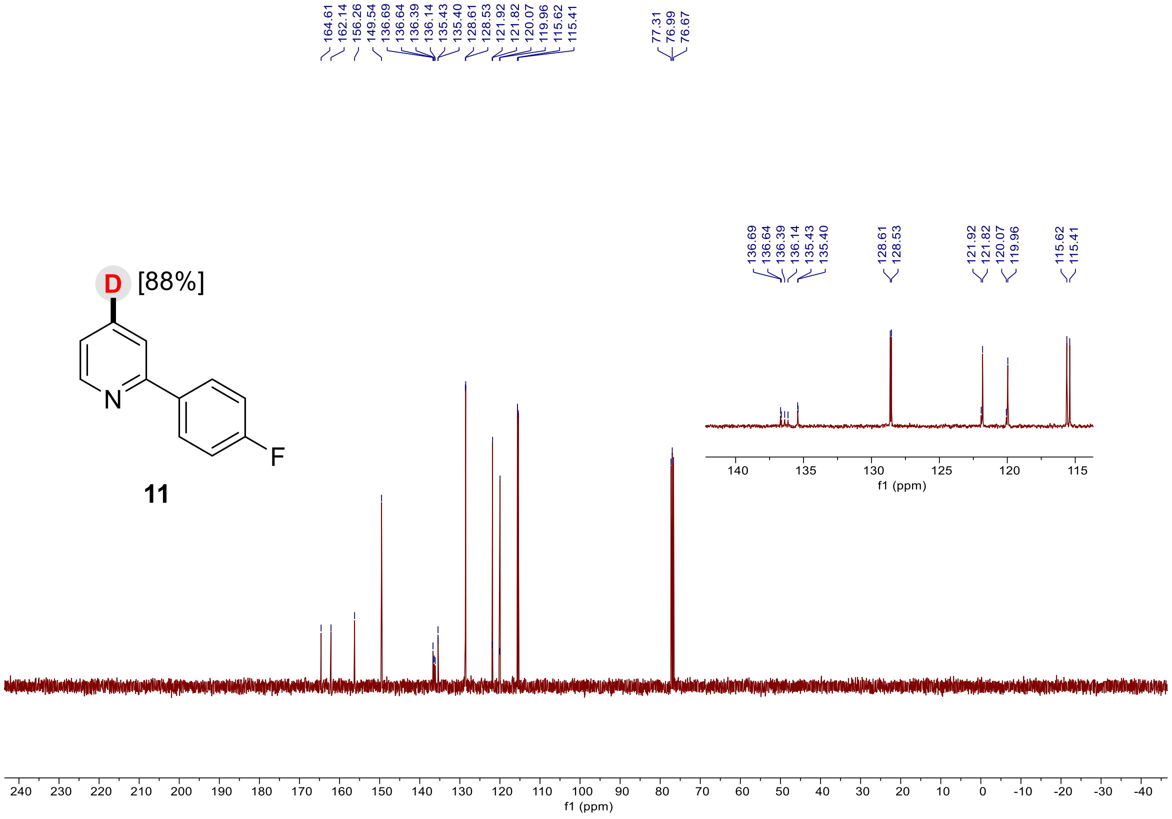

$^{19}$**F NMR** of **11**

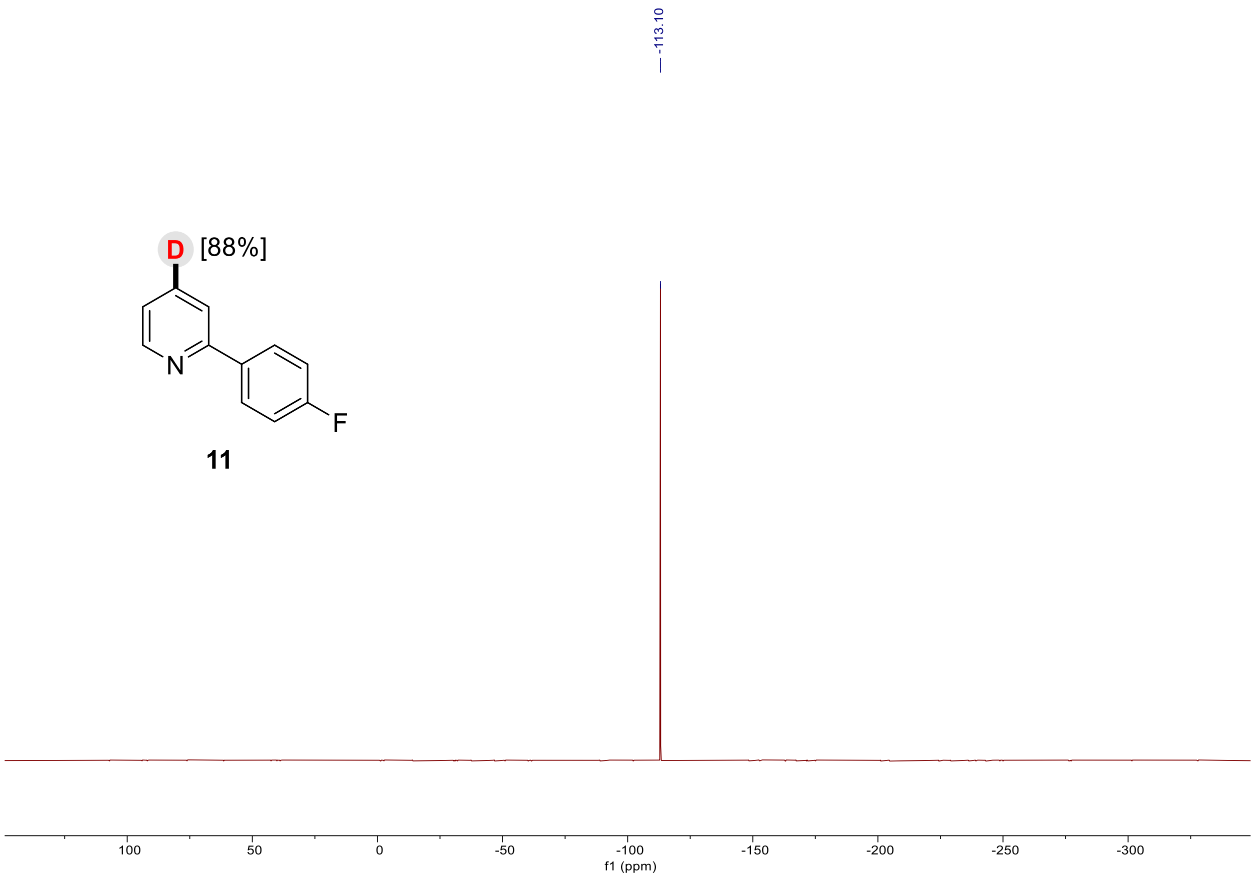

$^1$H NMR and $^{13}$C NMR of 12

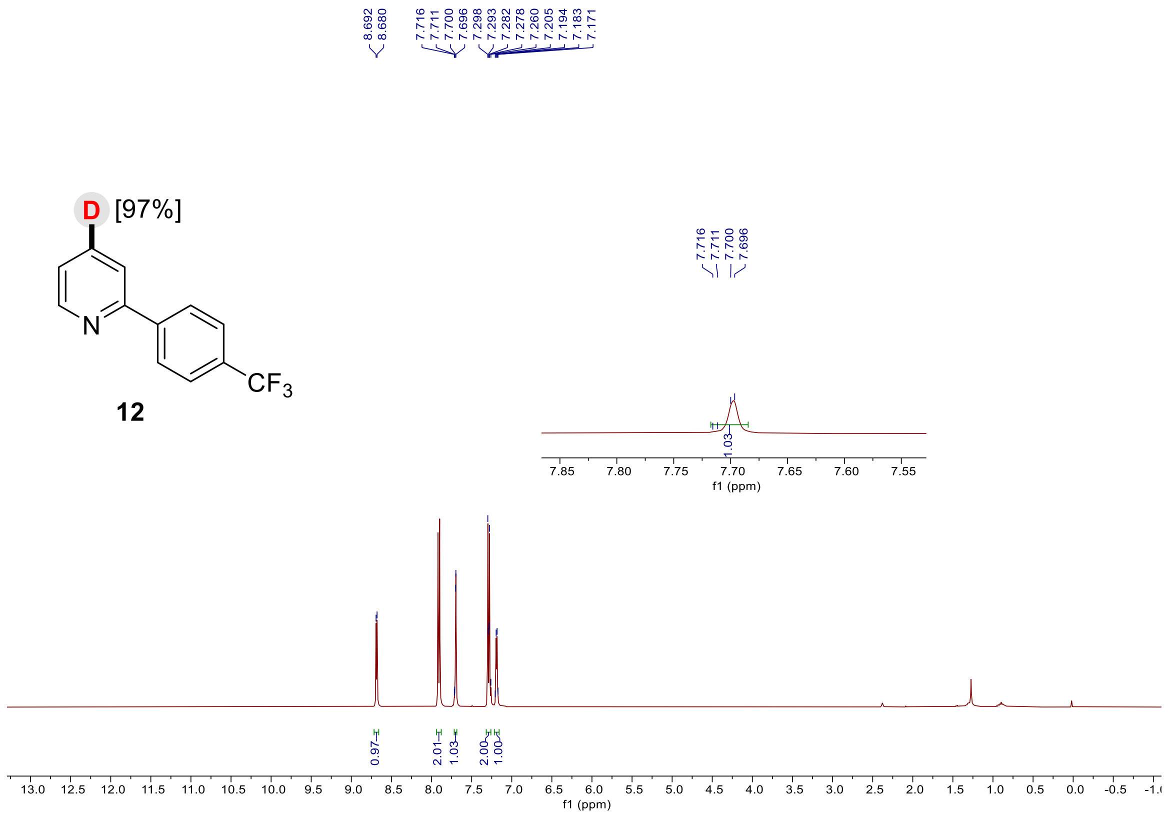


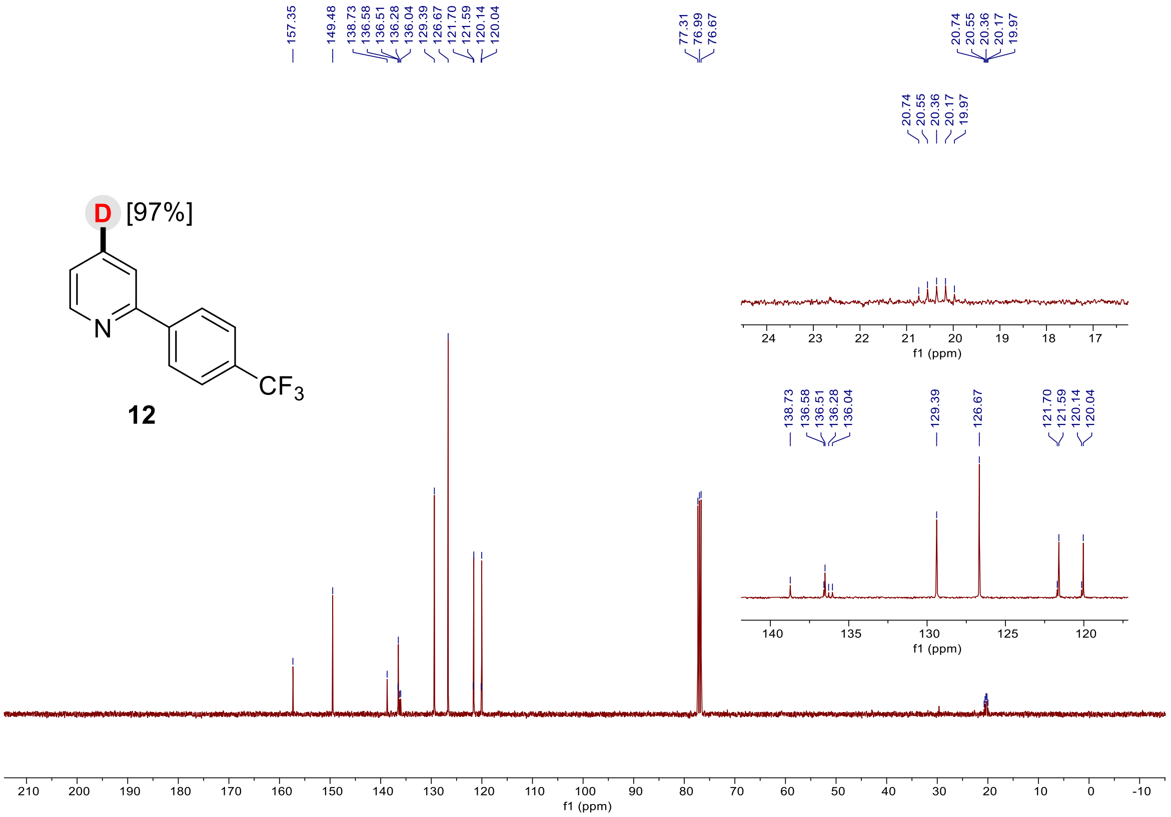

**$^{19}$F NMR** of **12**

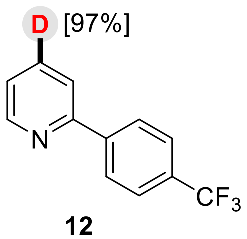


**12**

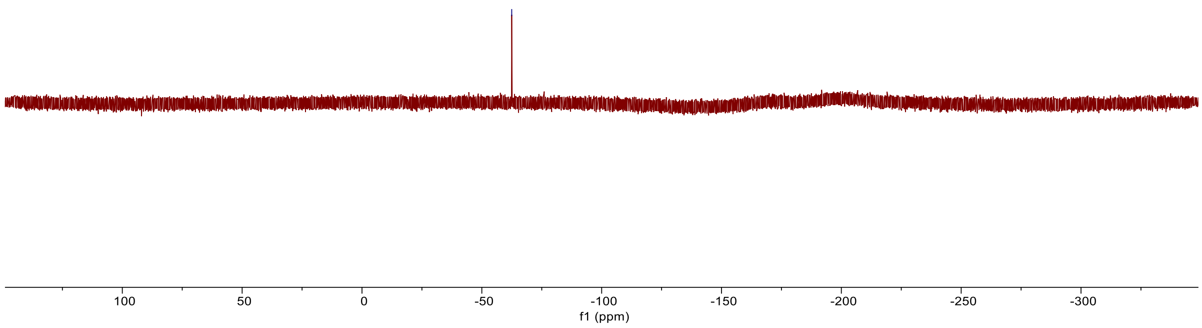

$^{1}$H NMR and $^{13}$C NMR of **13**

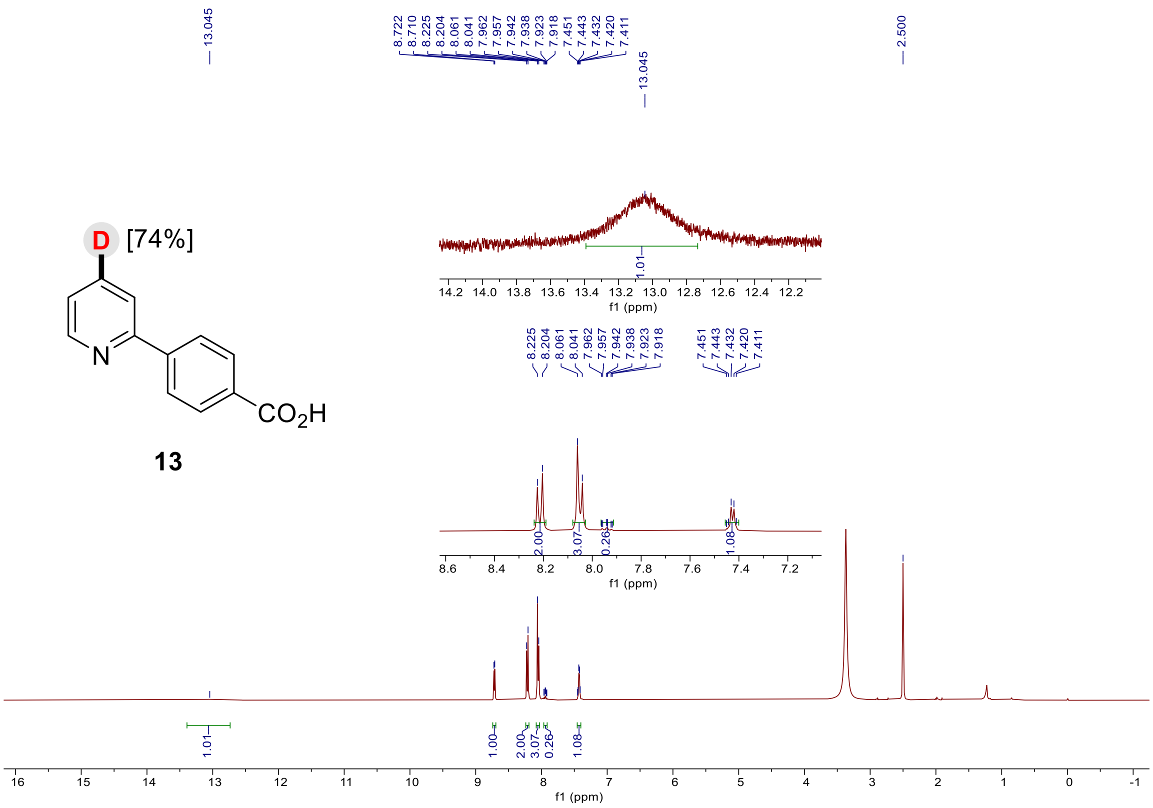


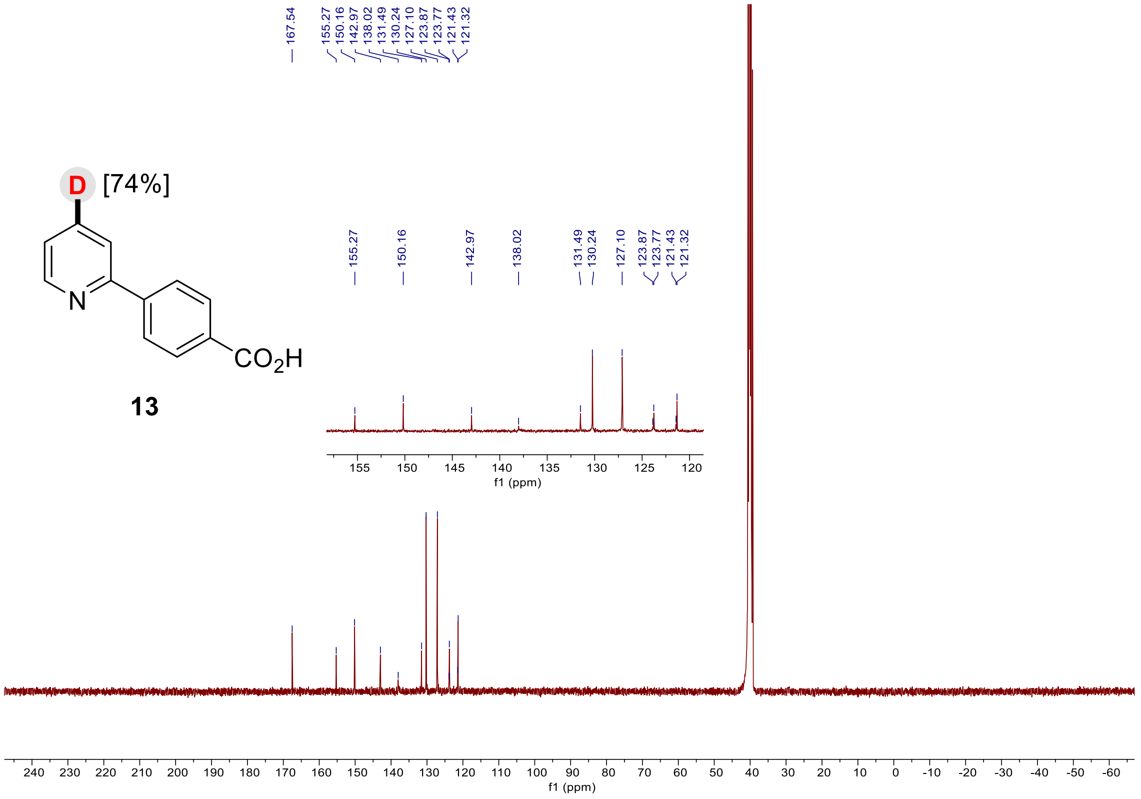

**$^1$H NMR and $^{13}$C NMR of 14**

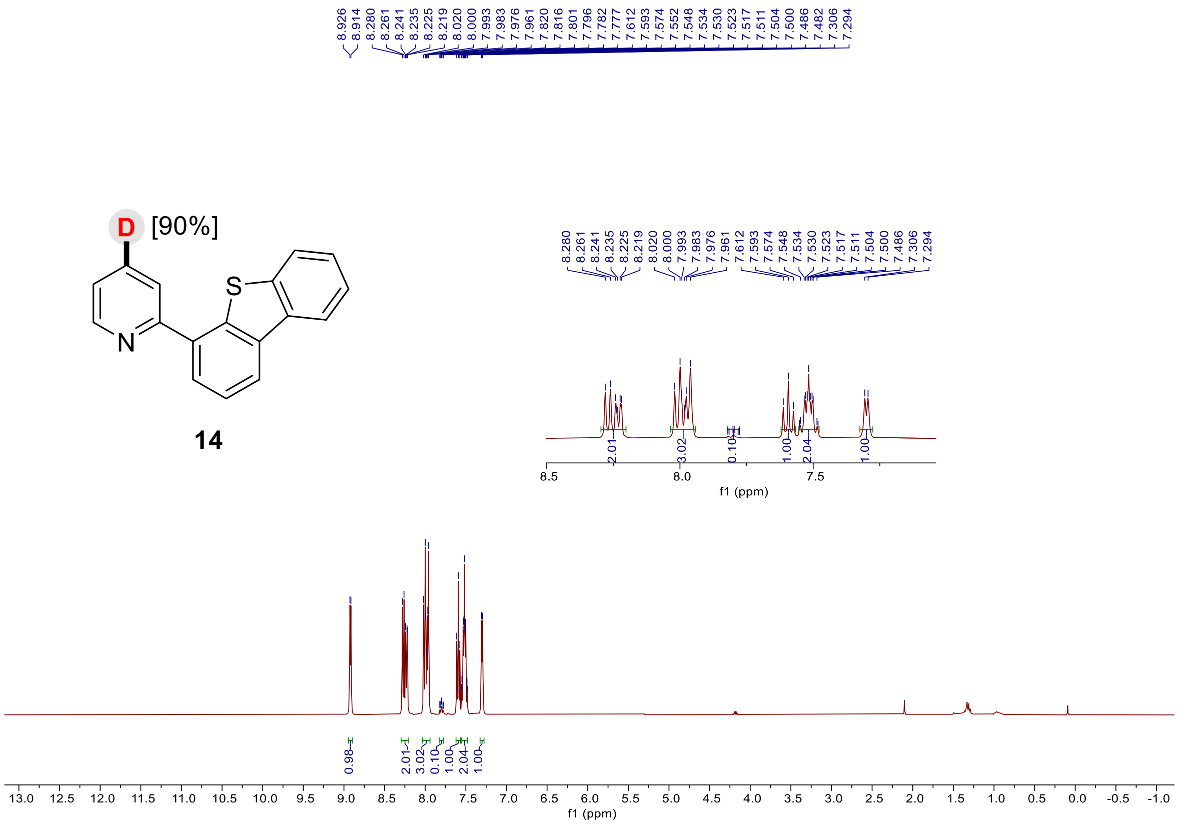


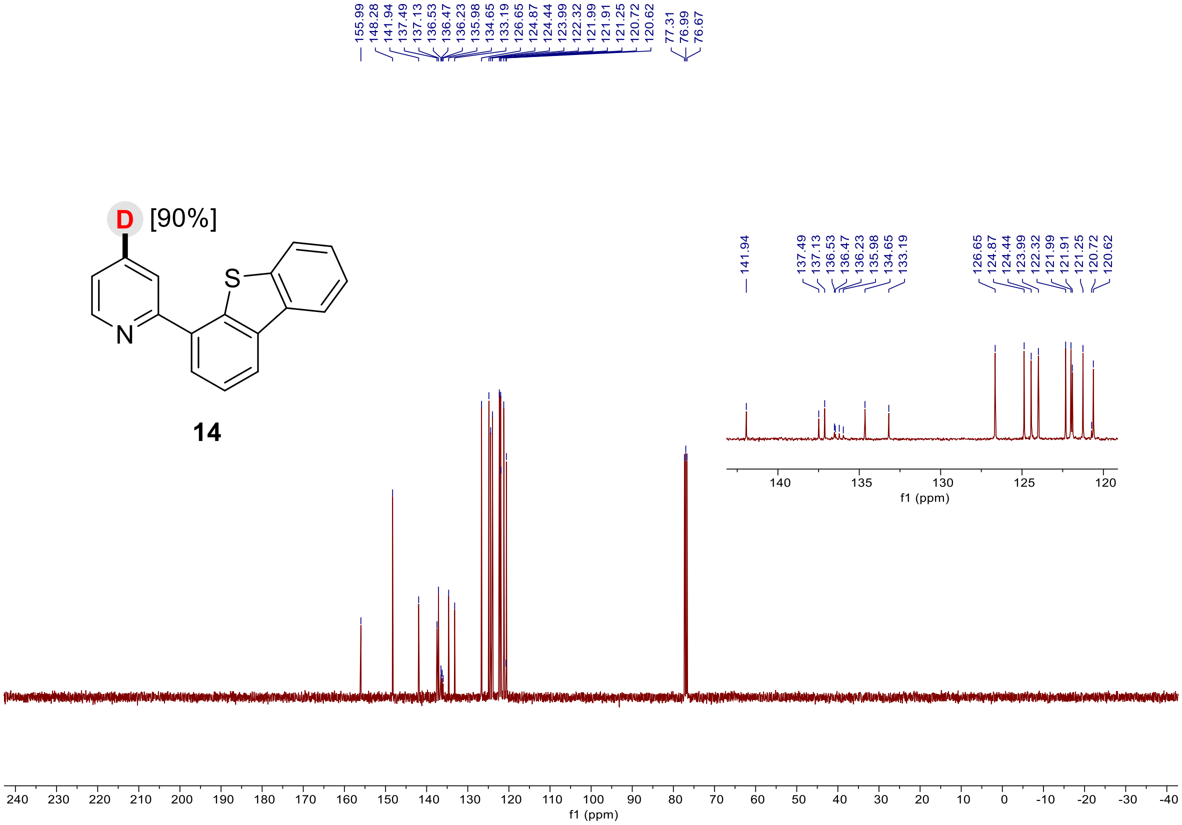

**$^{1}$H NMR** and **$^{13}$C NMR** of **15**

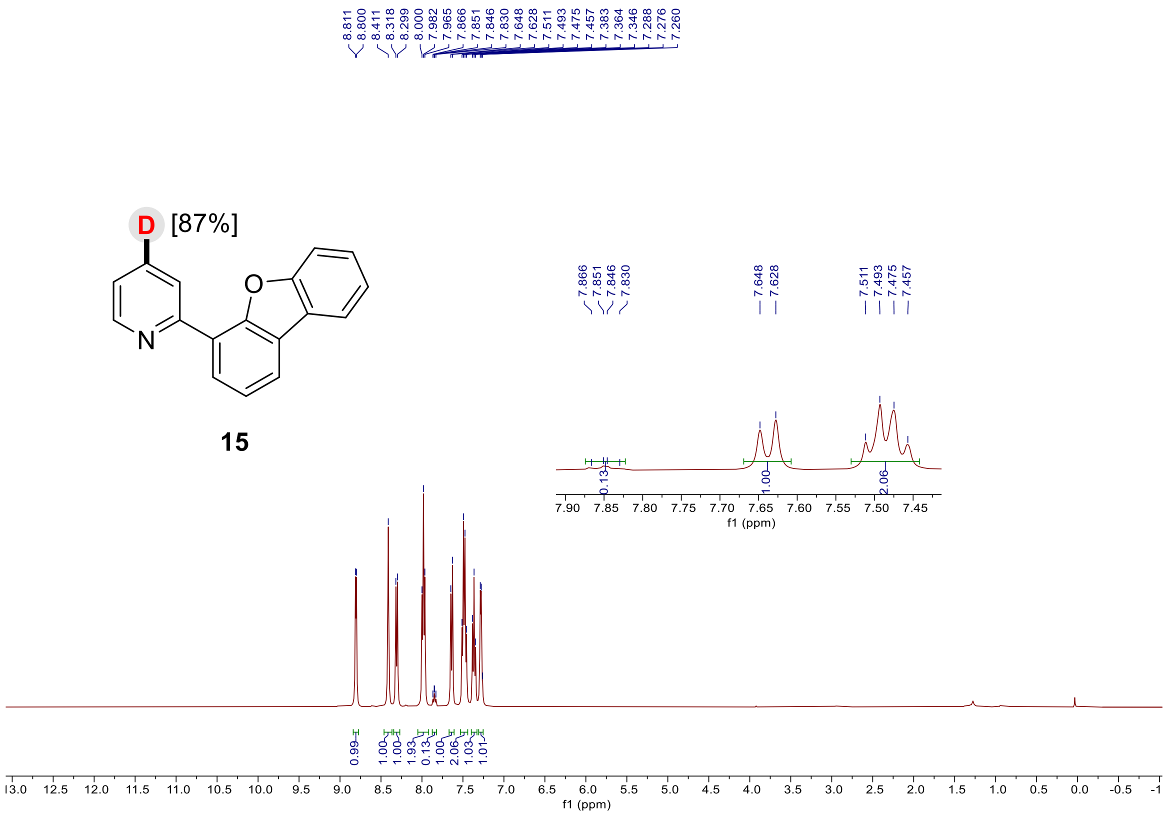


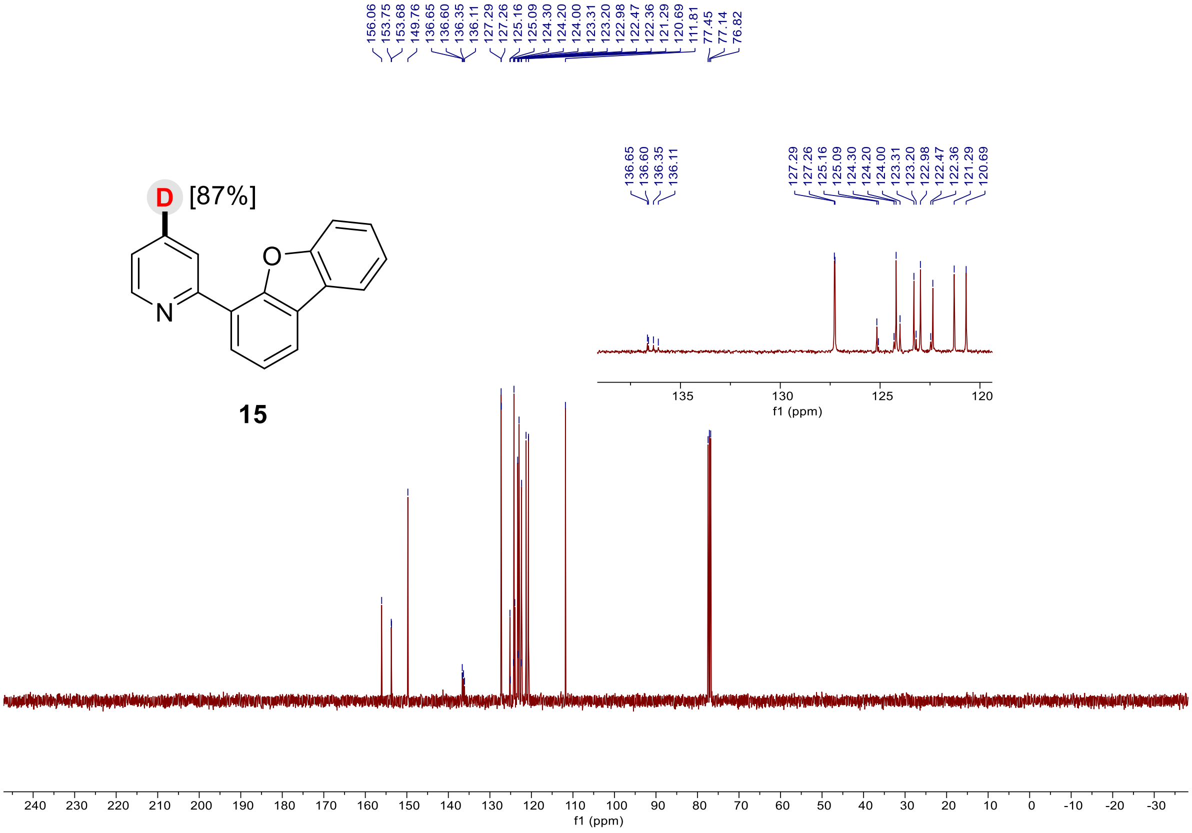

$^1$H NMR and $^{13}$C NMR of **16**

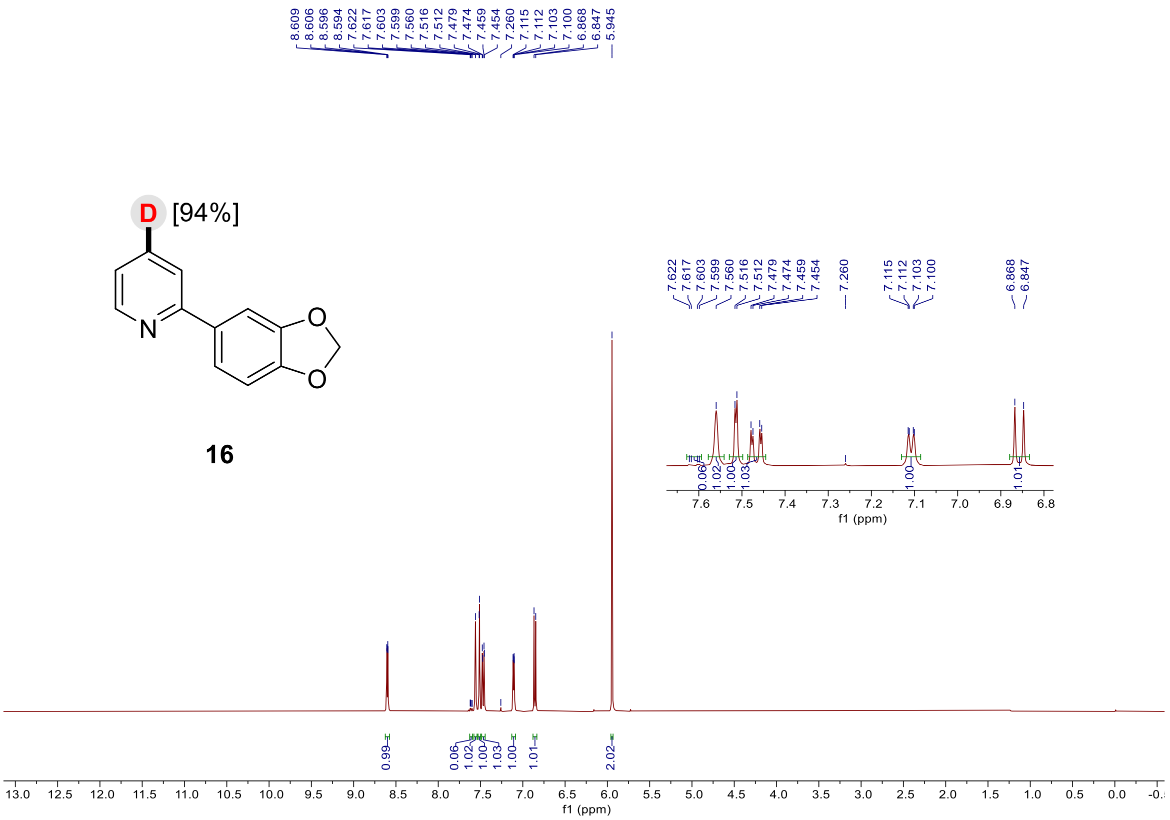


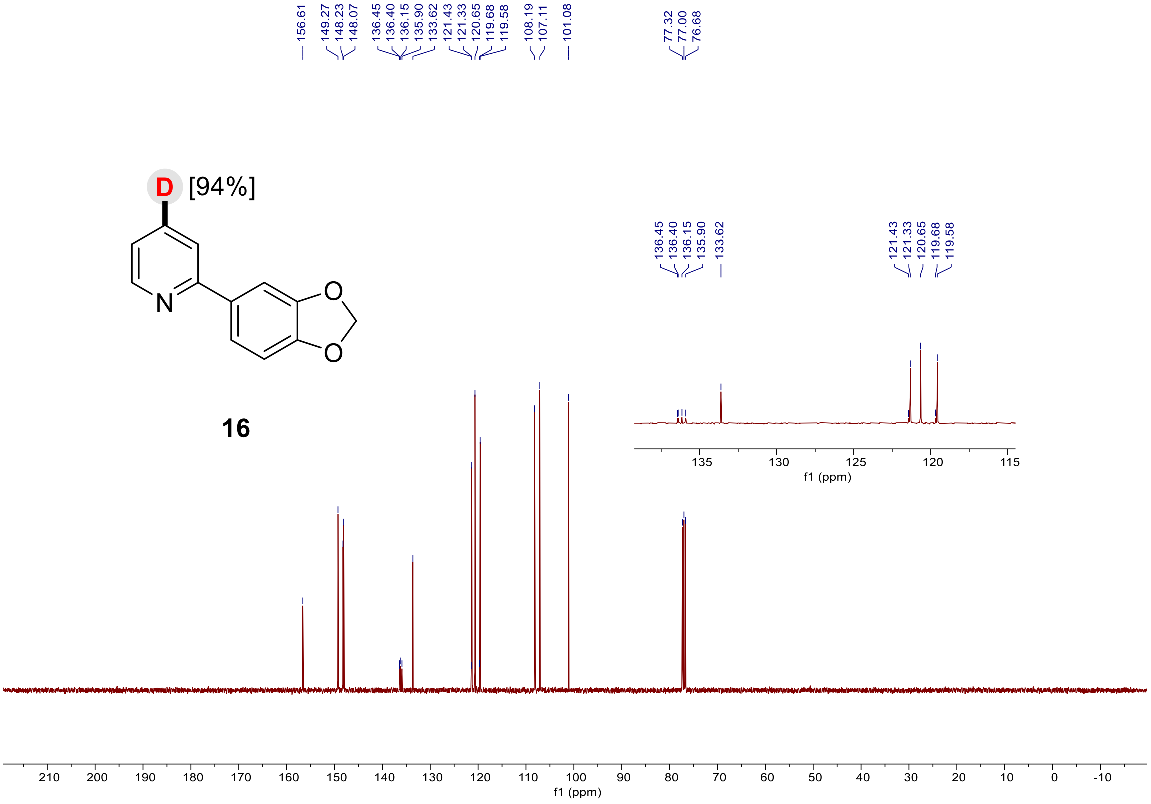

**$^{1}$H NMR** and **$^{13}$C NMR** of **17**

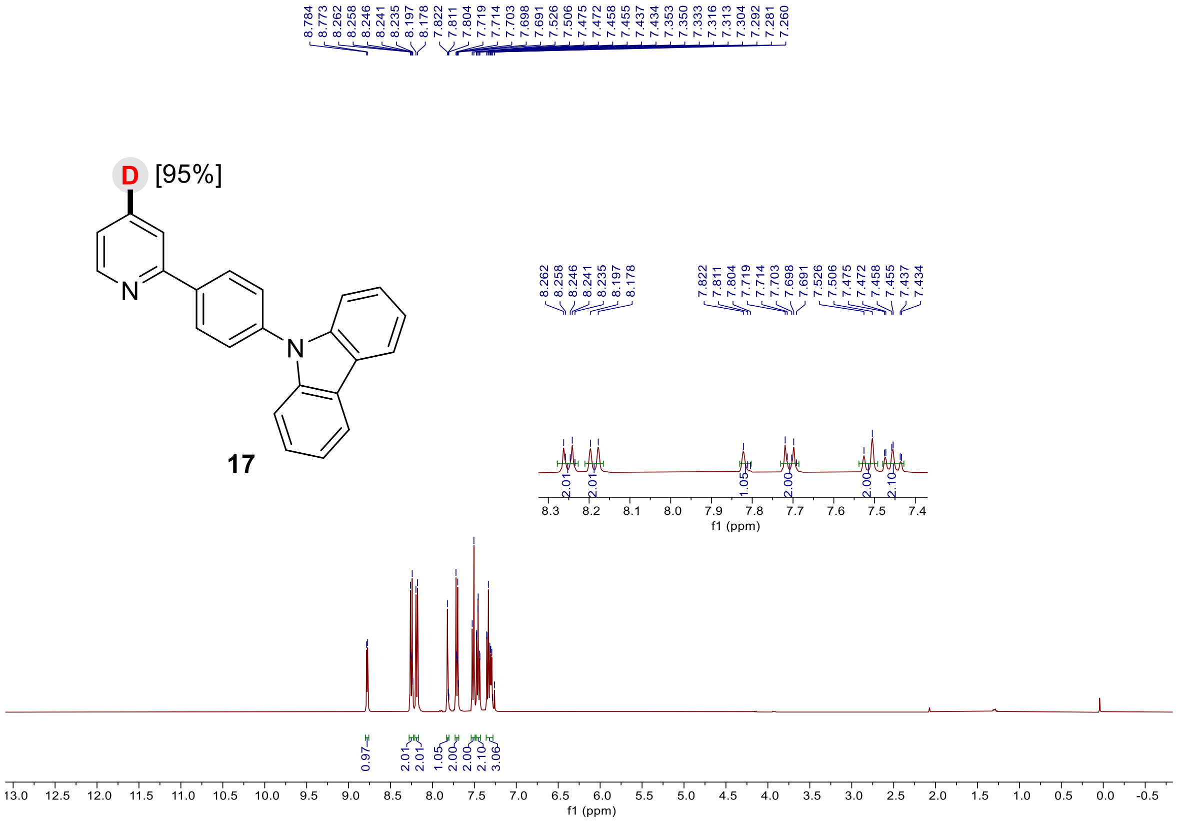


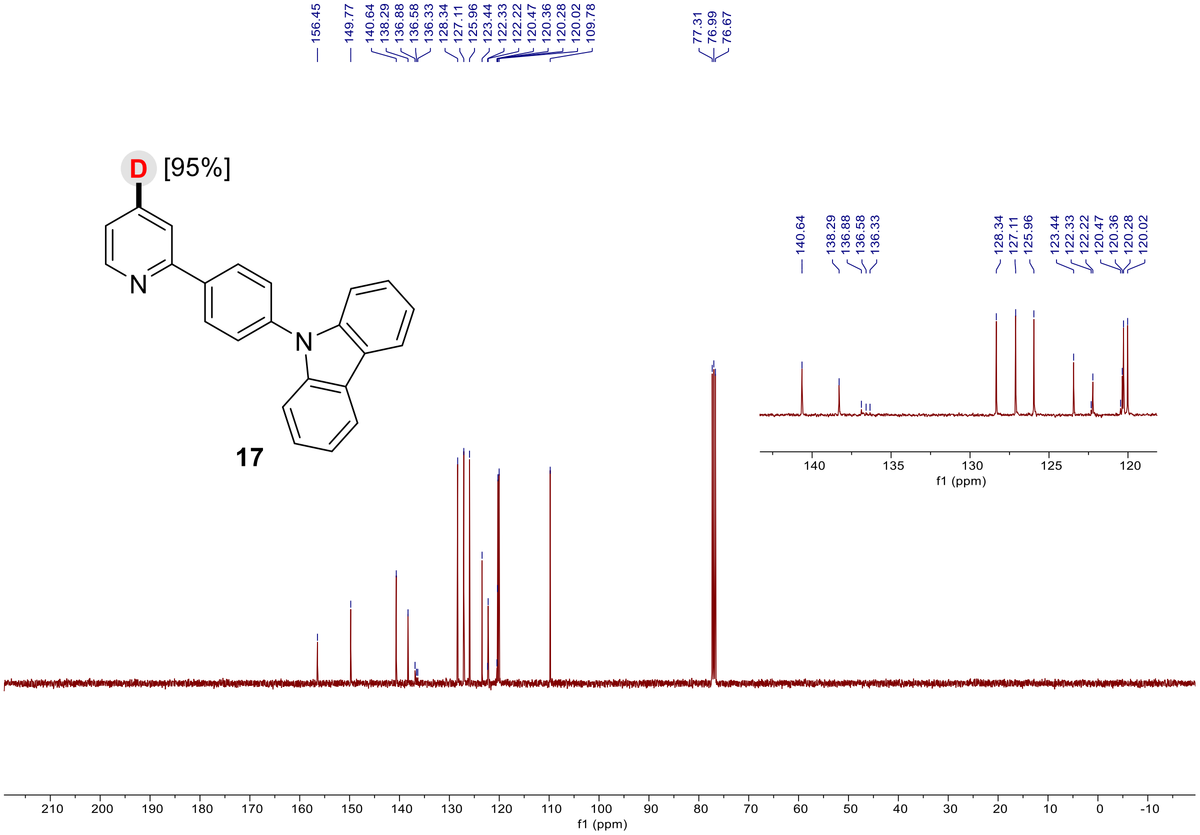

**$^{1}$H NMR** and **$^{13}$C NMR** of **18**

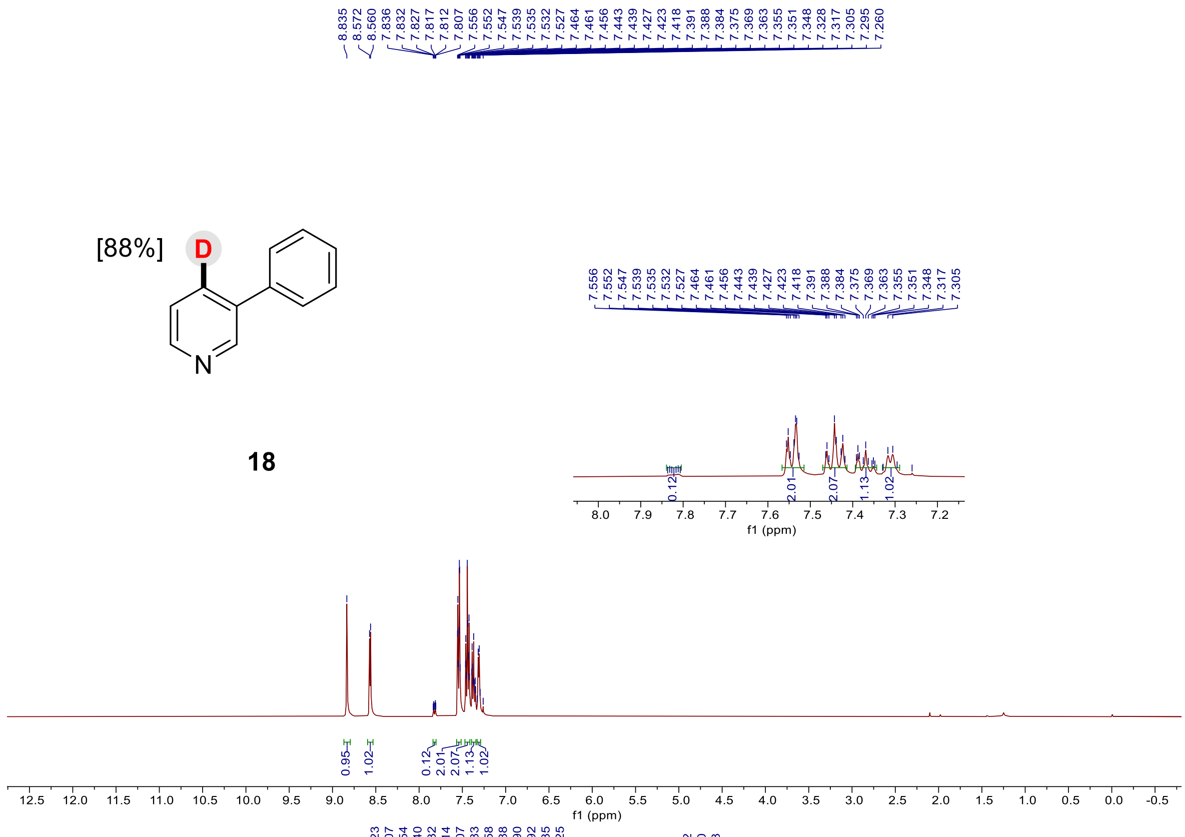


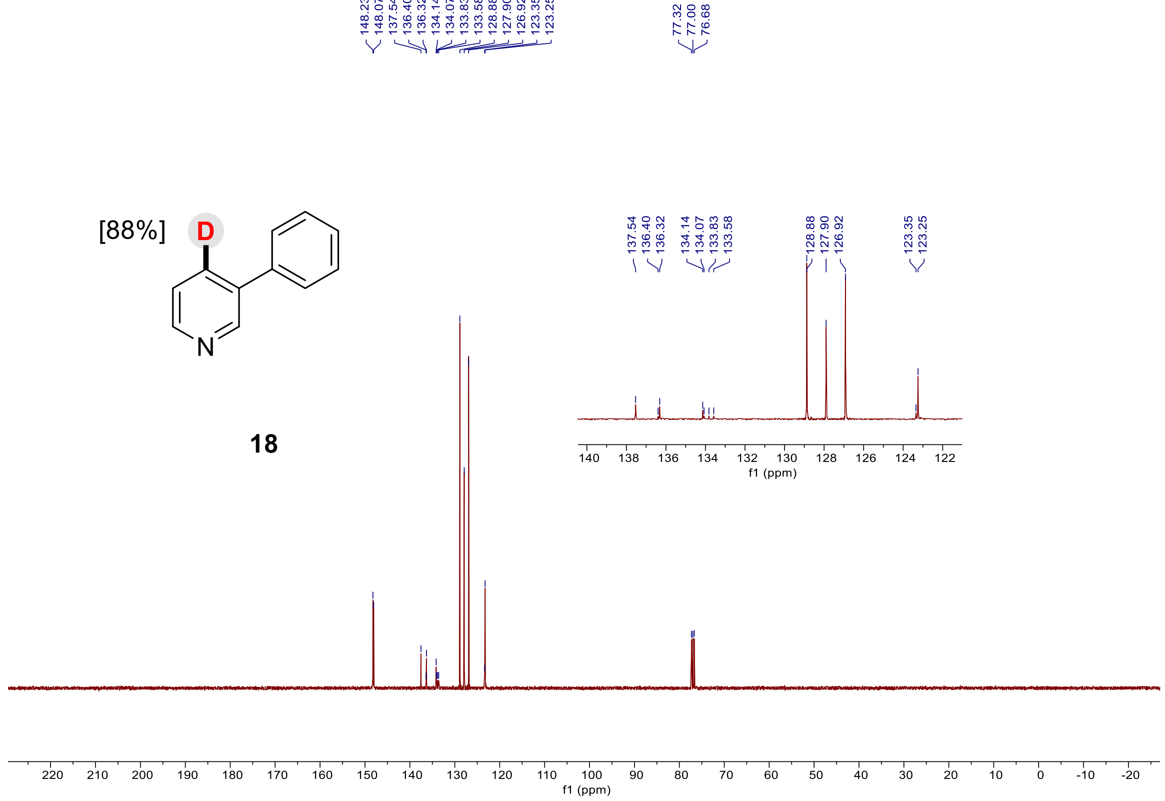

$^{1}$H NMR and $^{13}$C NMR of **19**

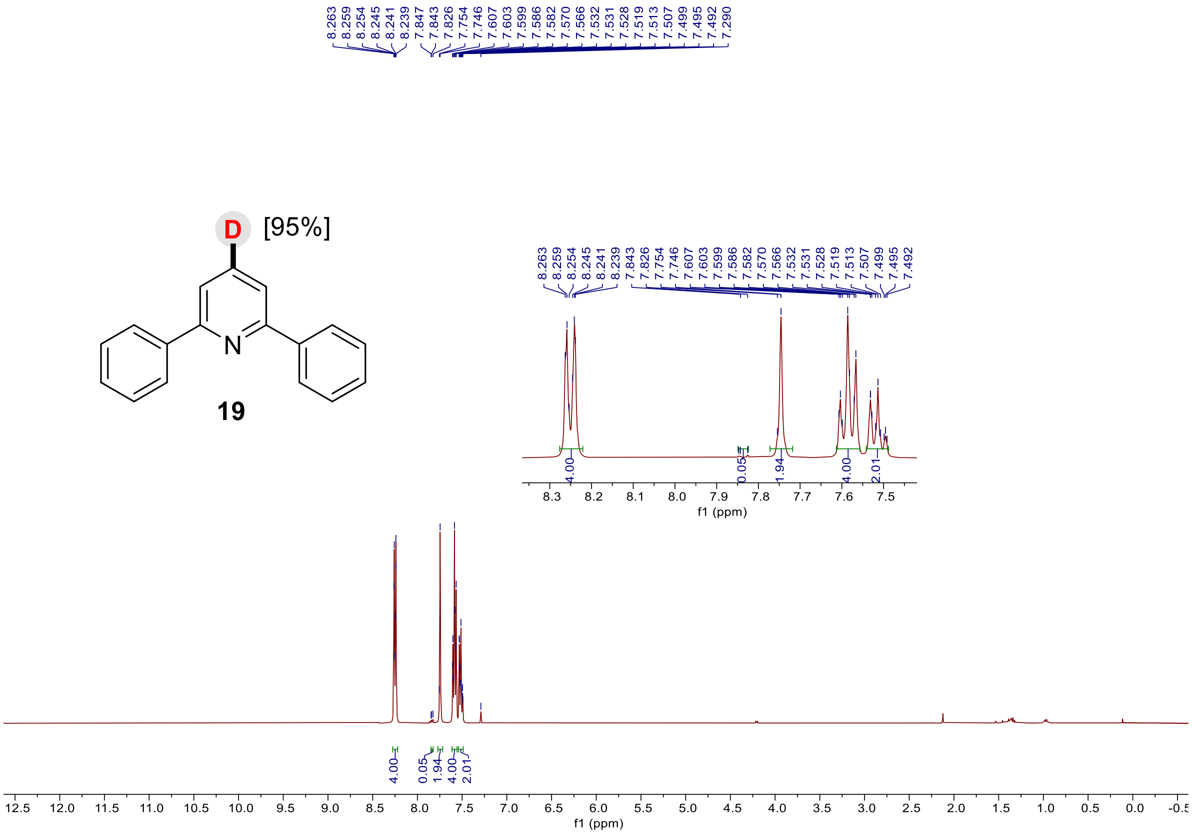


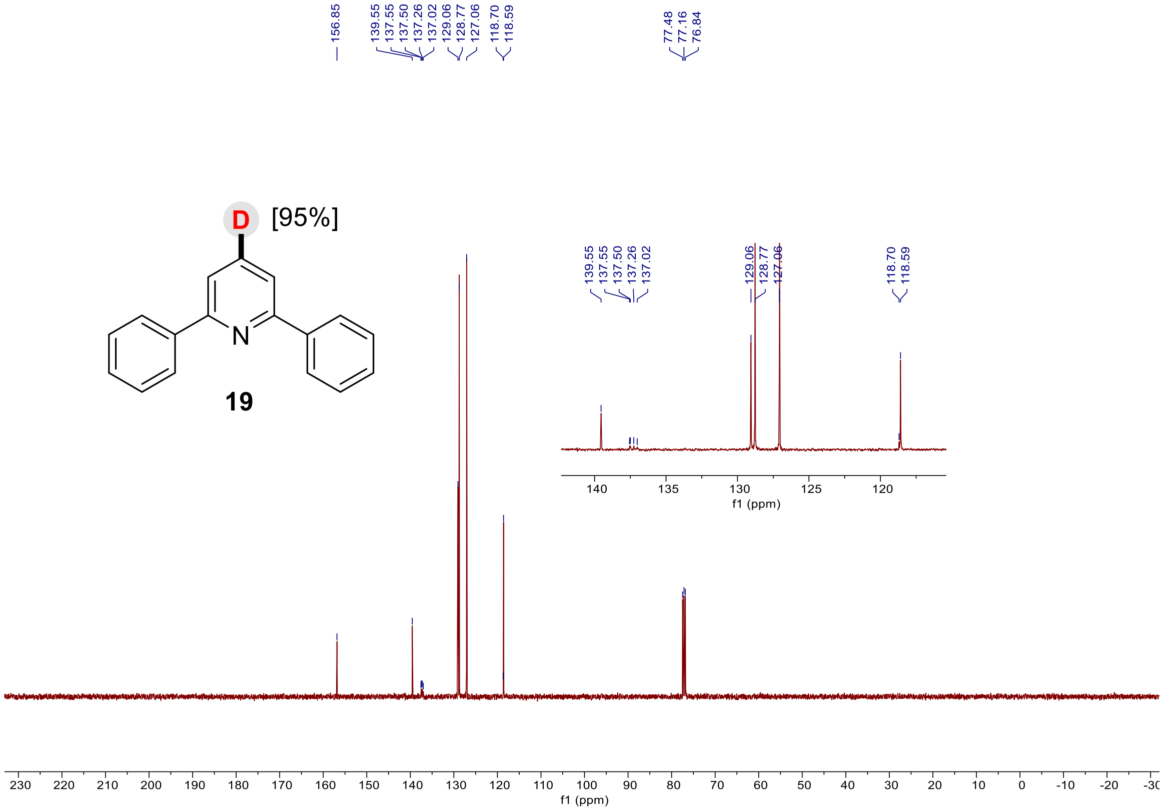

$^1$H NMR and $^{13}$C NMR of **20**

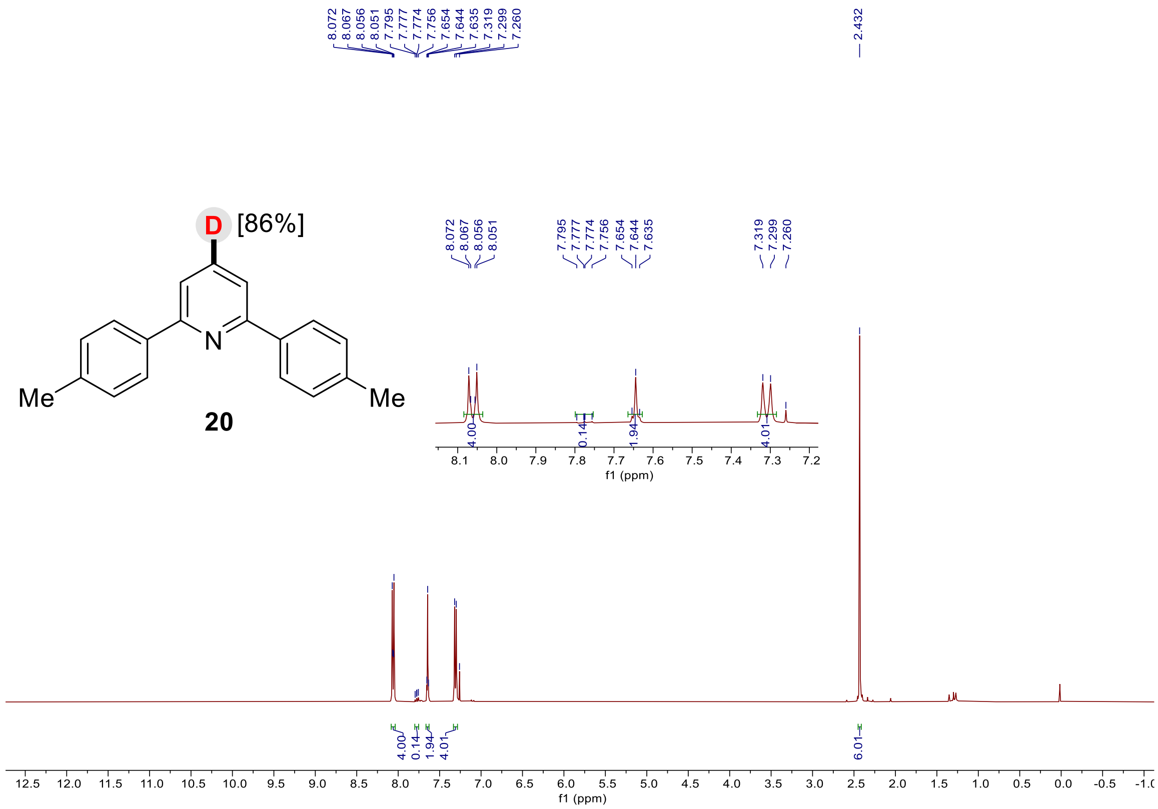

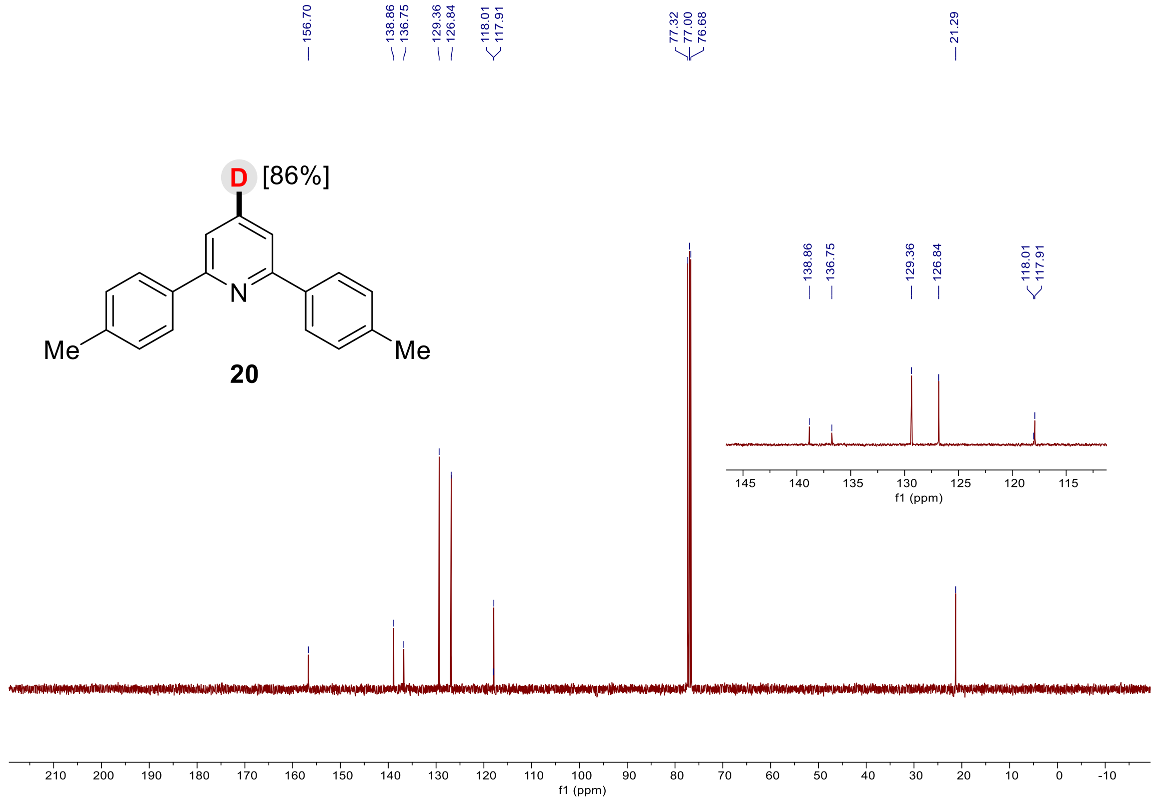

**$^{1}$H NMR** and **$^{13}$C NMR** of **21**

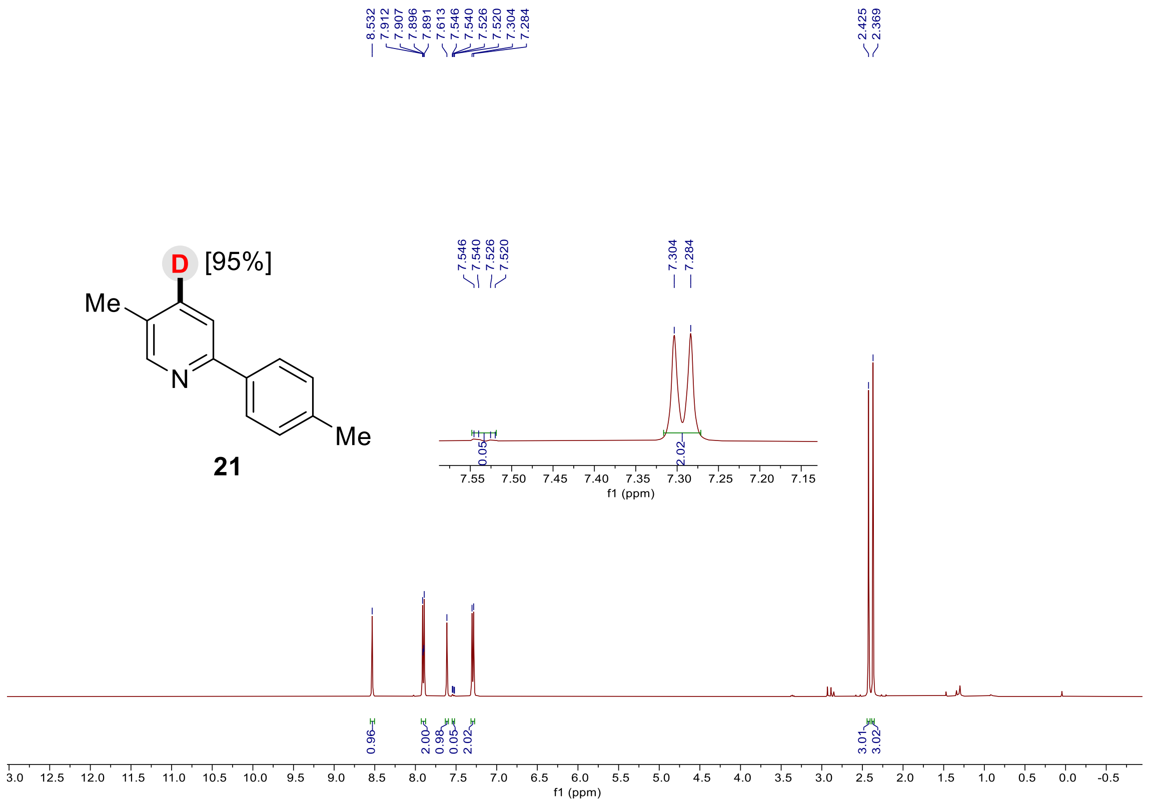


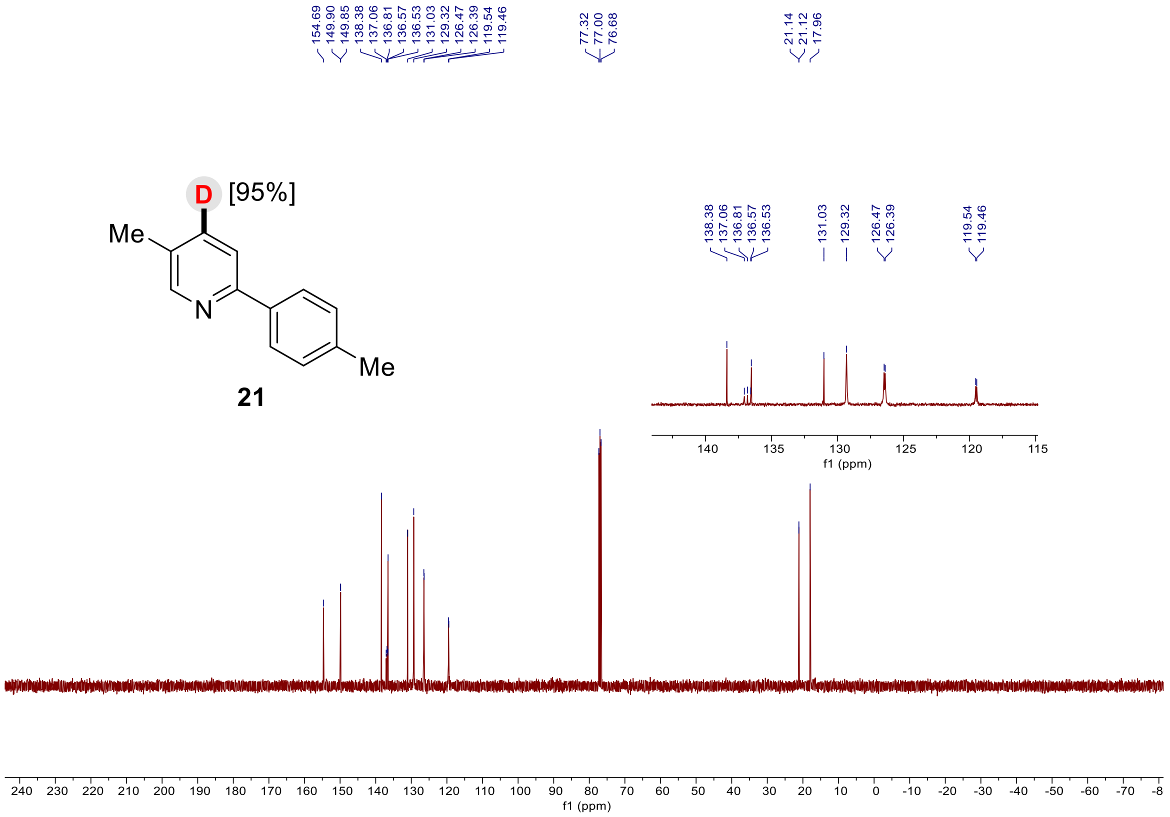

## $^{1}H$ NMR and $^{13}C$ NMR of 22

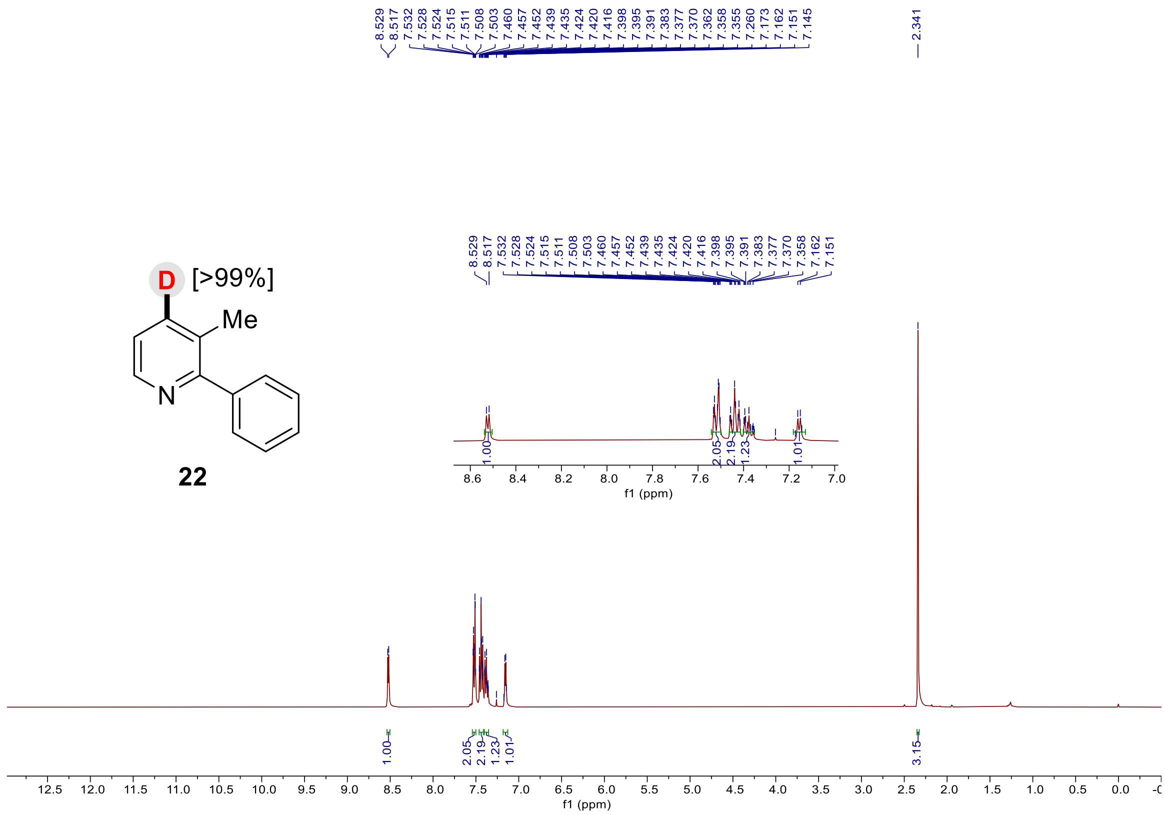

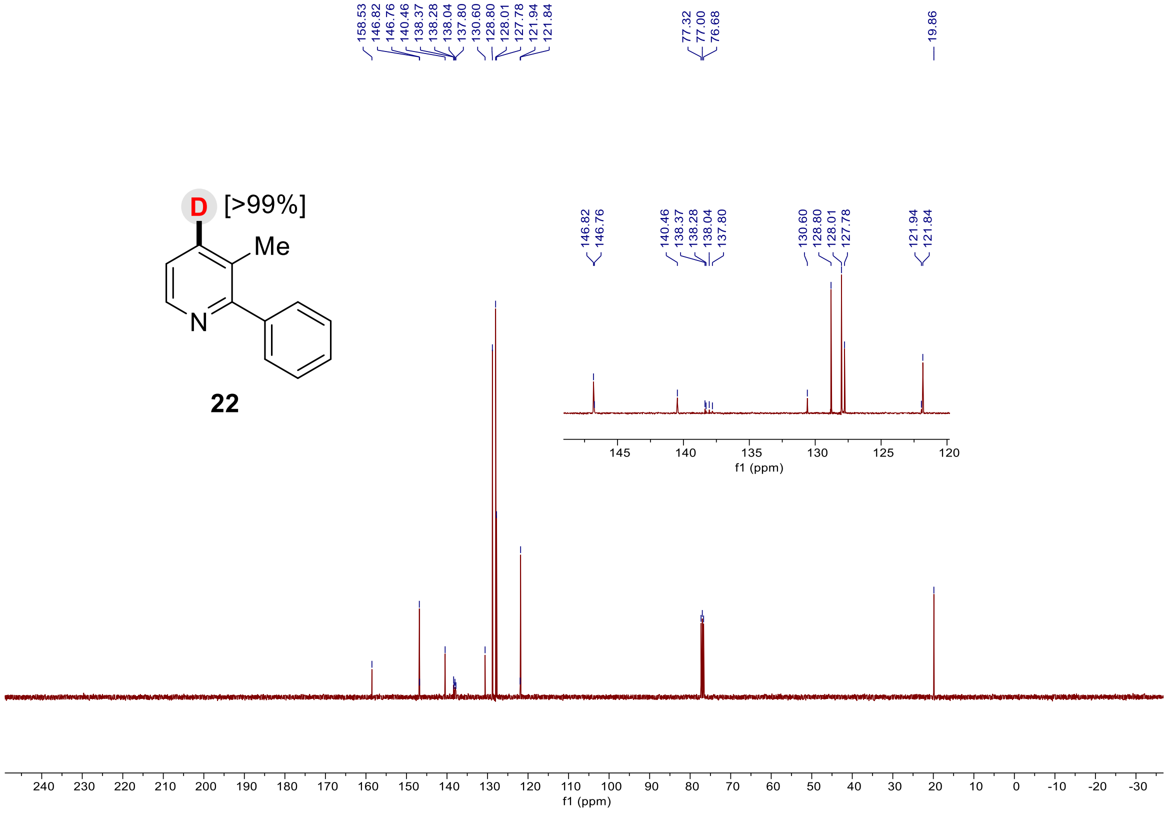

## $^{1}H$ NMR and $^{13}C$ NMR of **23**

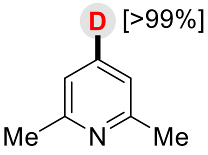


**23**

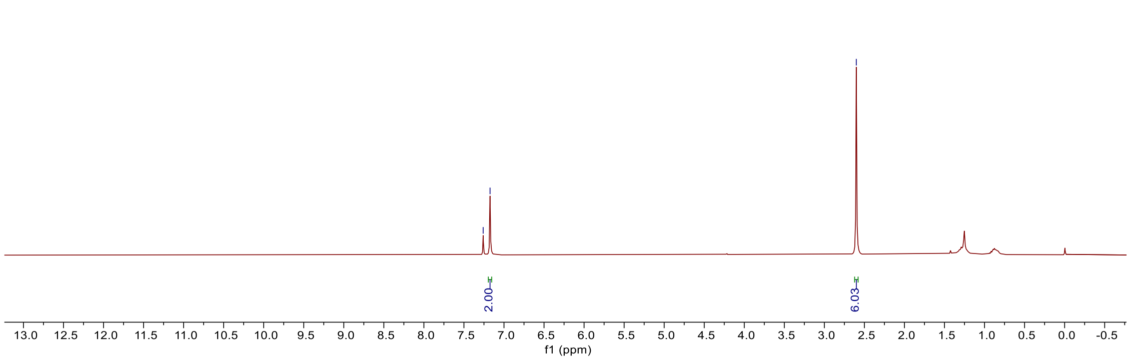


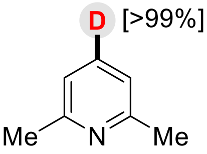


**23**

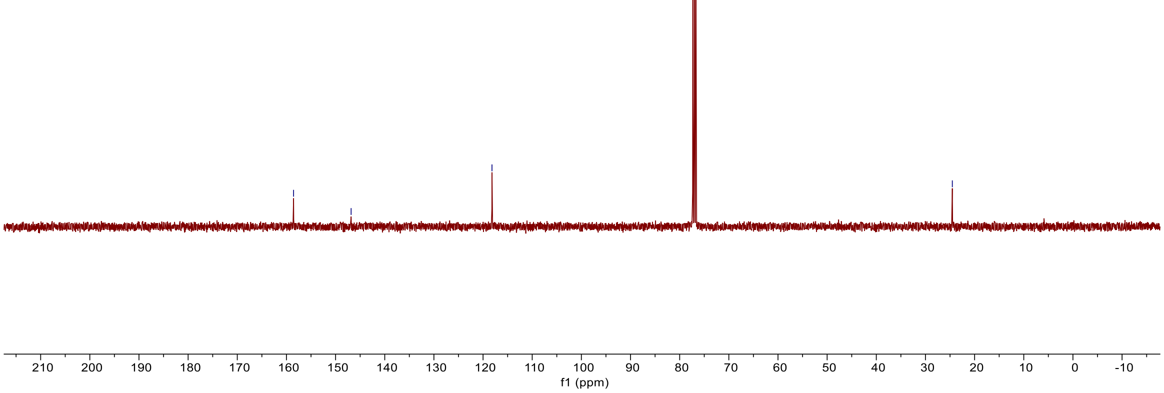

$^{1}$H NMR and $^{13}$C NMR of **24**

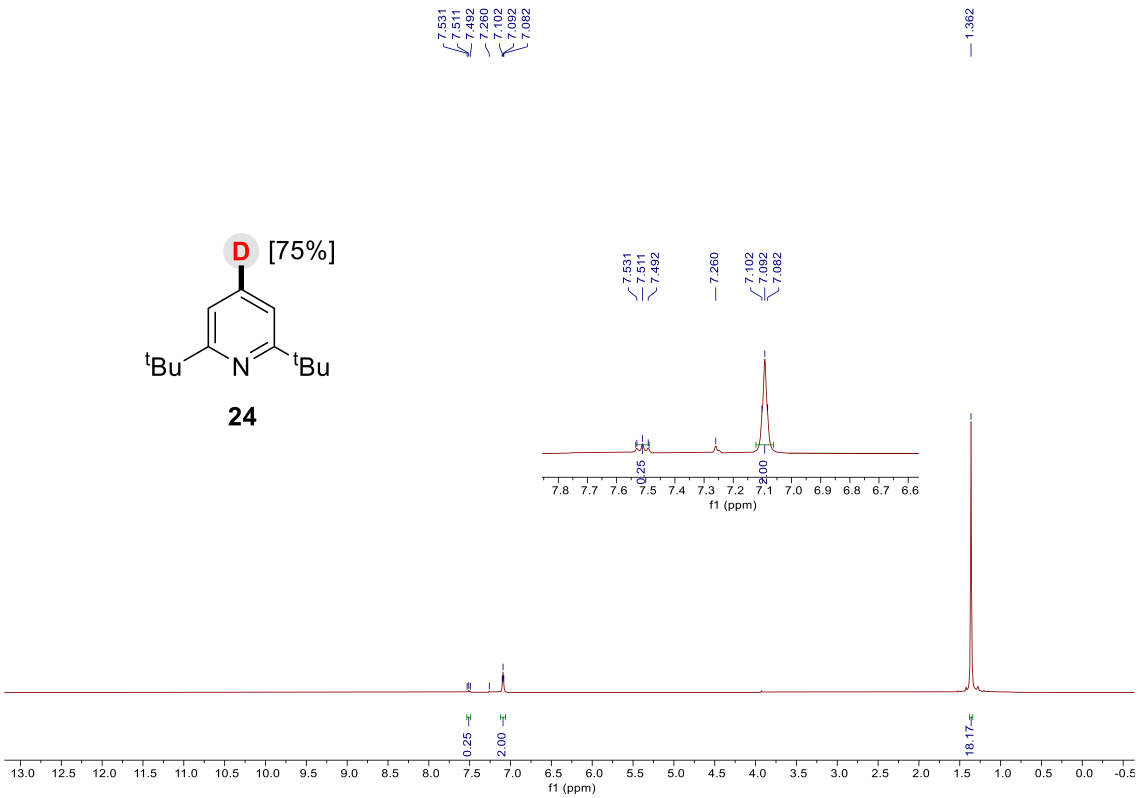

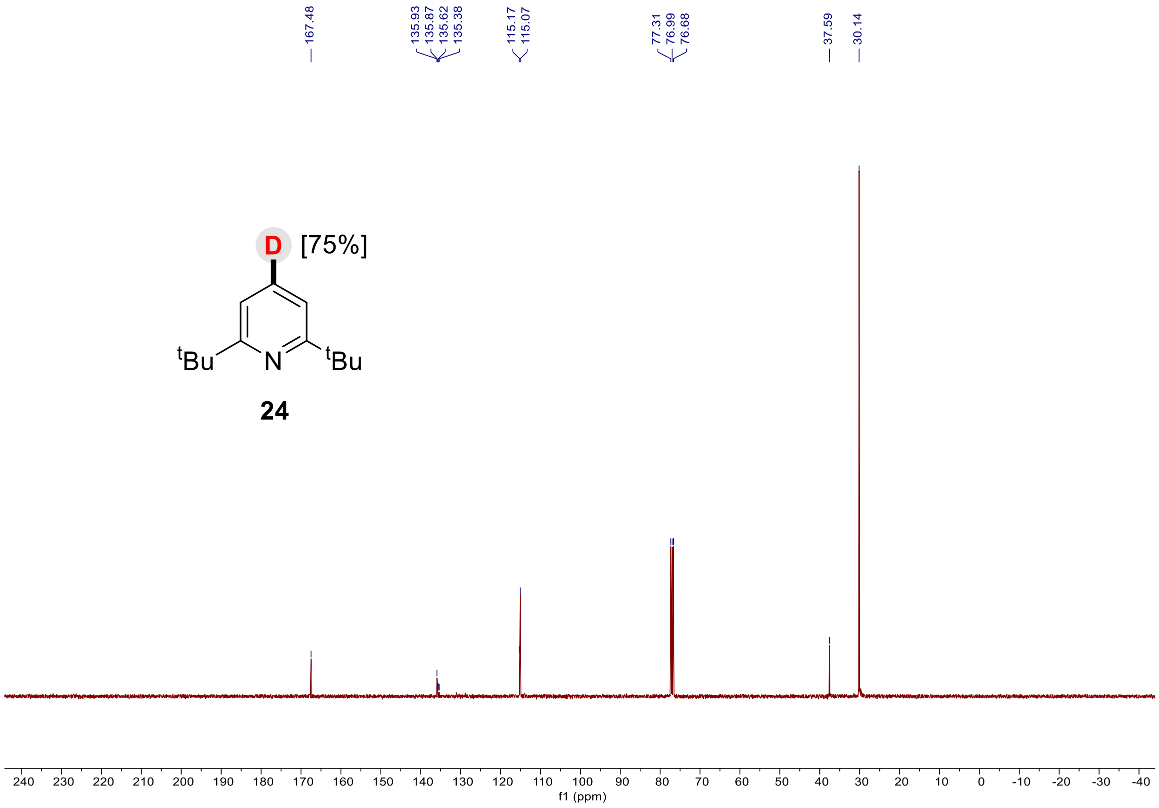

**$^{1}$H NMR** and **$^{13}$C NMR** of **25**

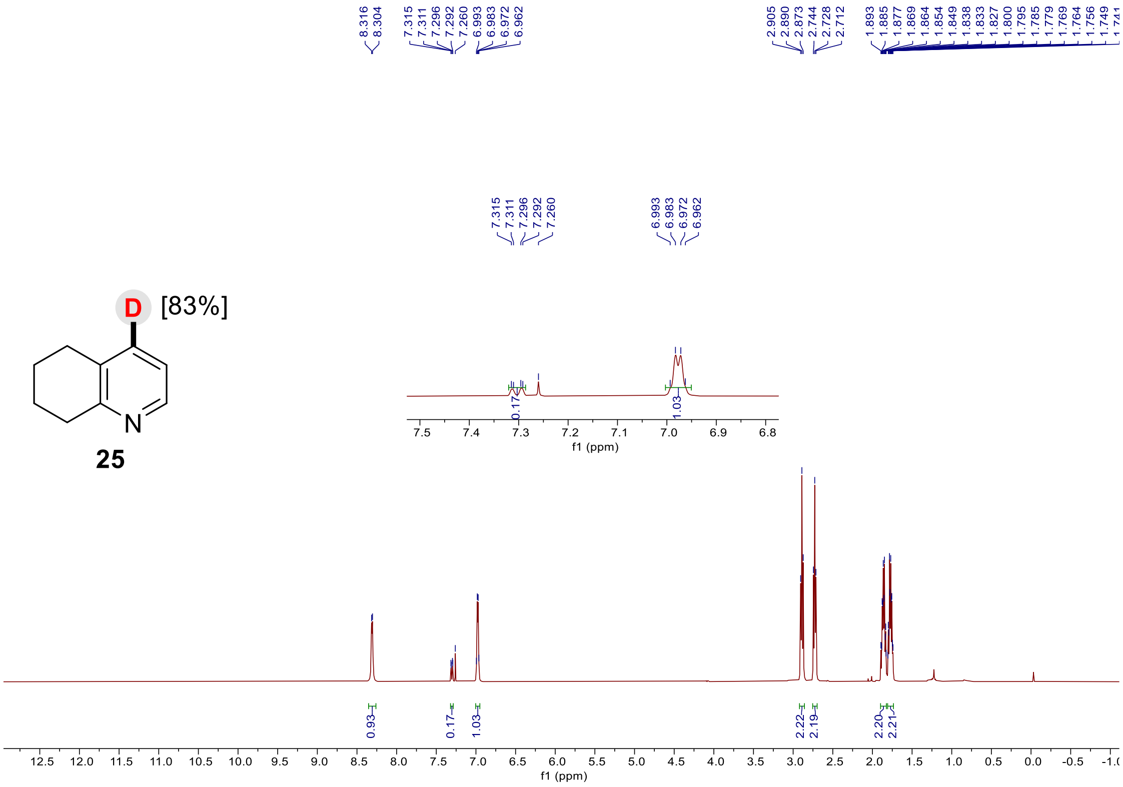


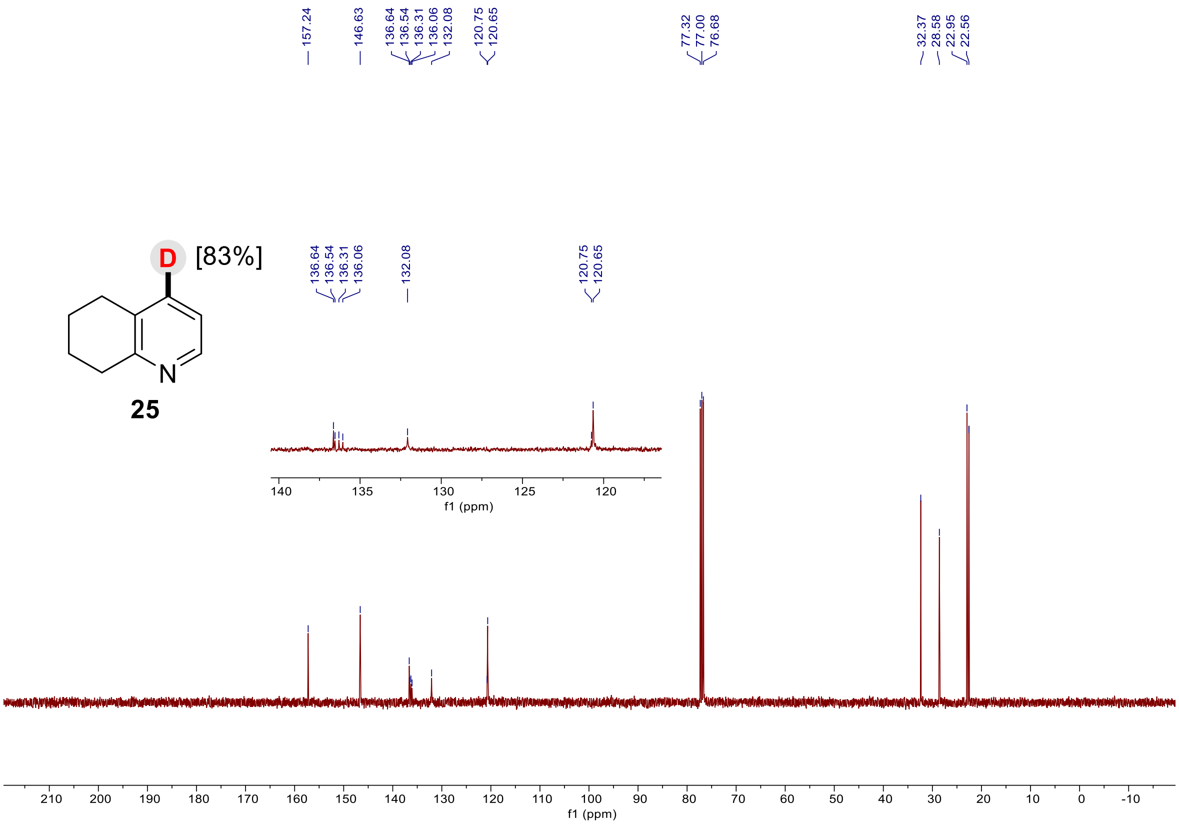

**$^{1}$H NMR** and **$^{13}$C NMR** of **26**

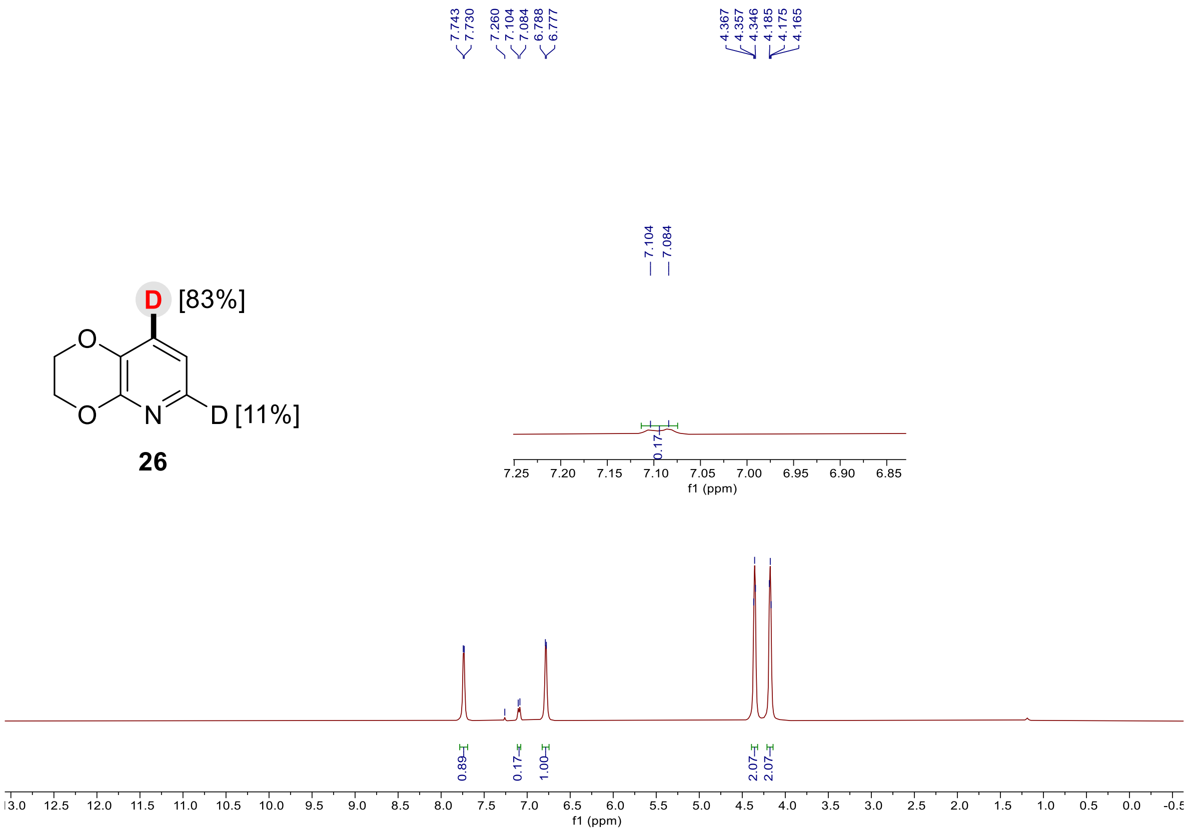


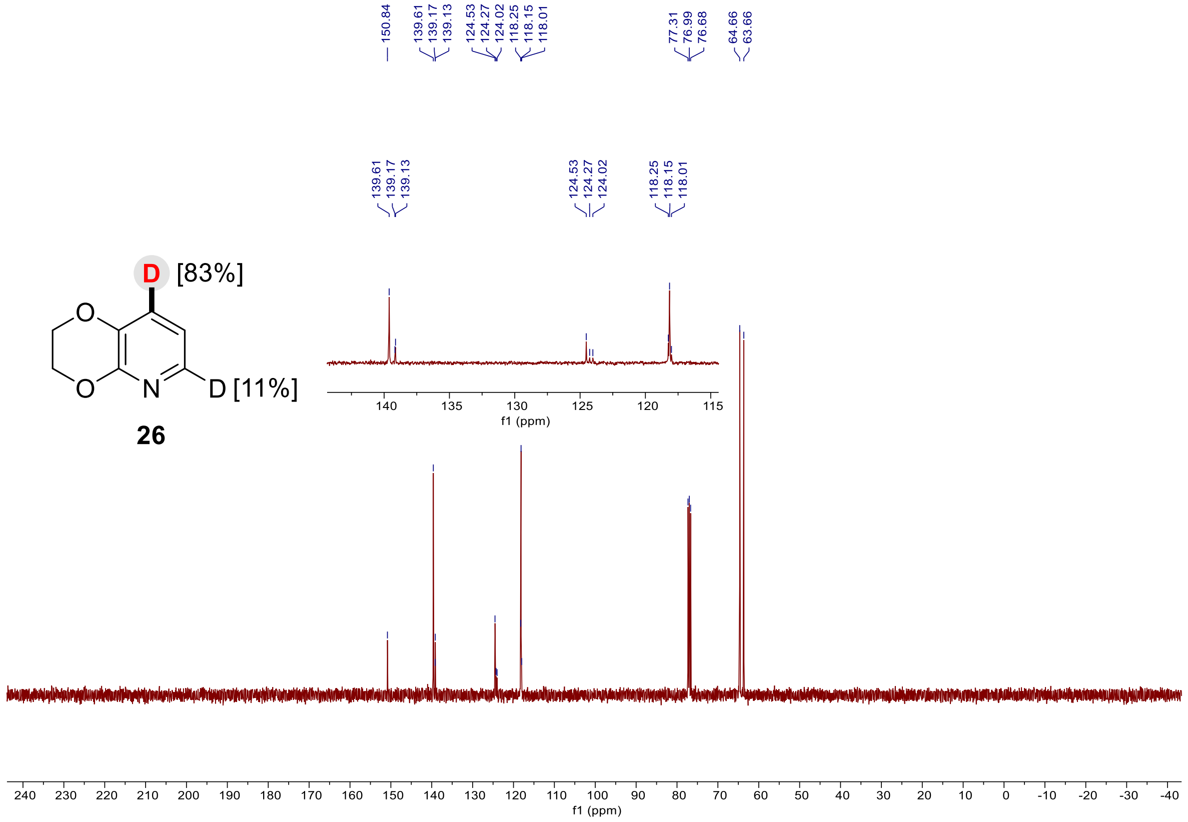

**$^1$H NMR** and **$^{13}$C NMR** of **27**

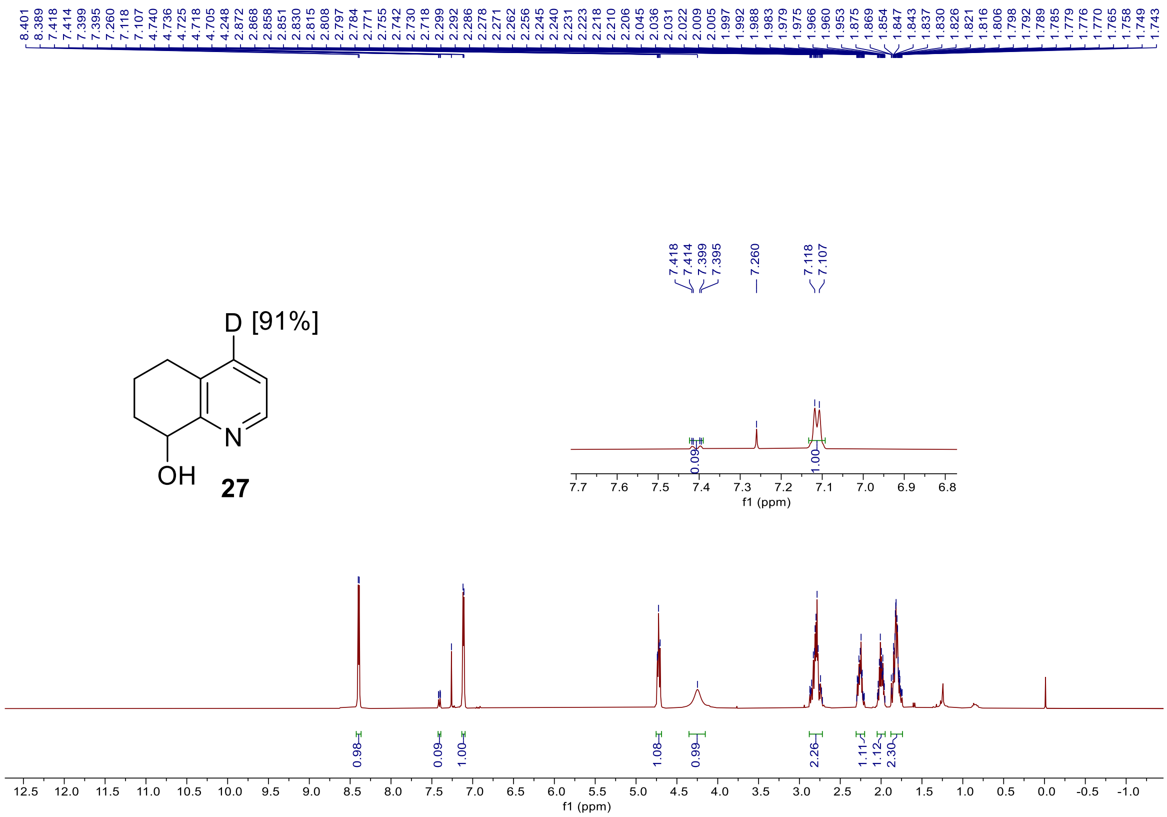


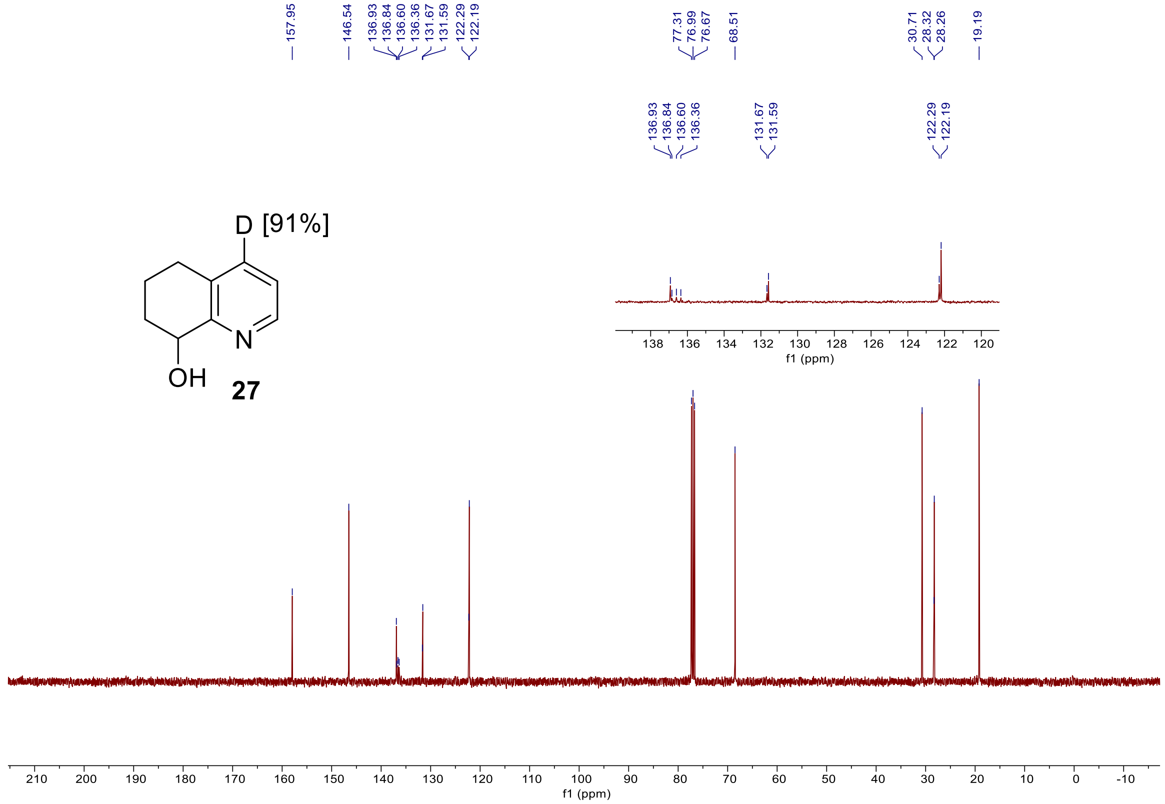

## $^{1}$H NMR and $^{13}$C NMR of 28

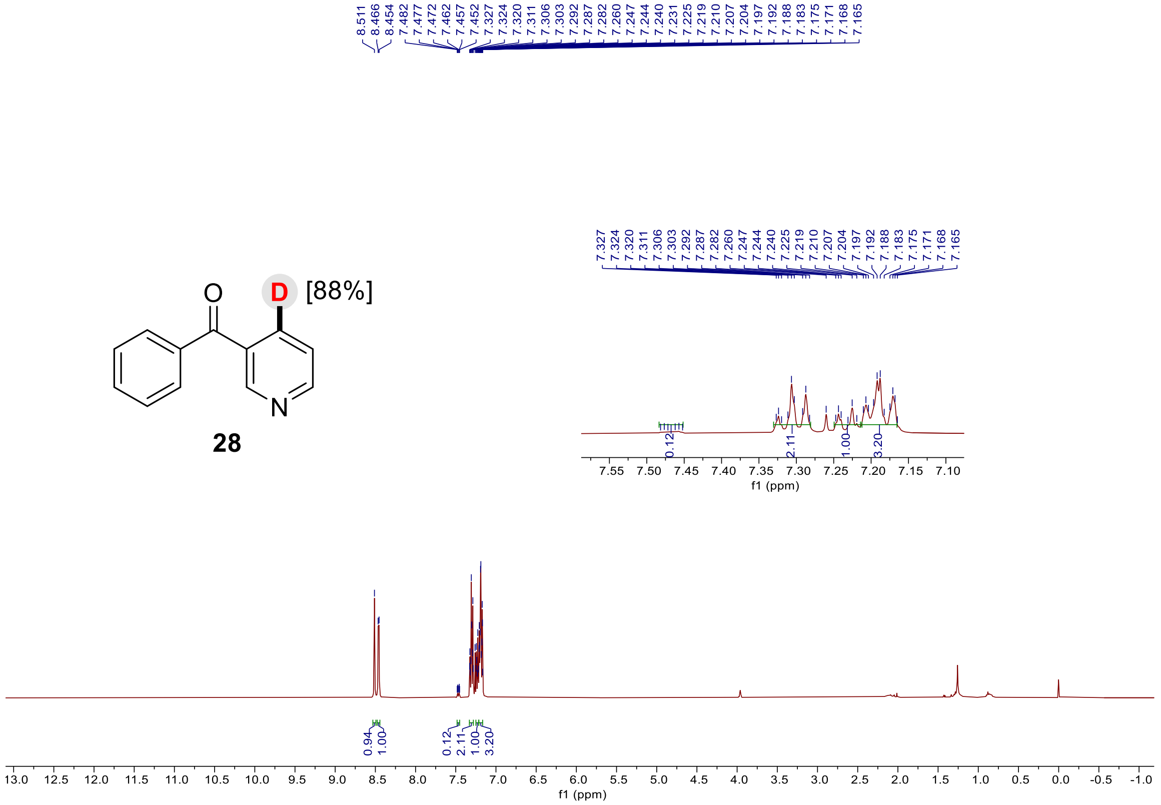


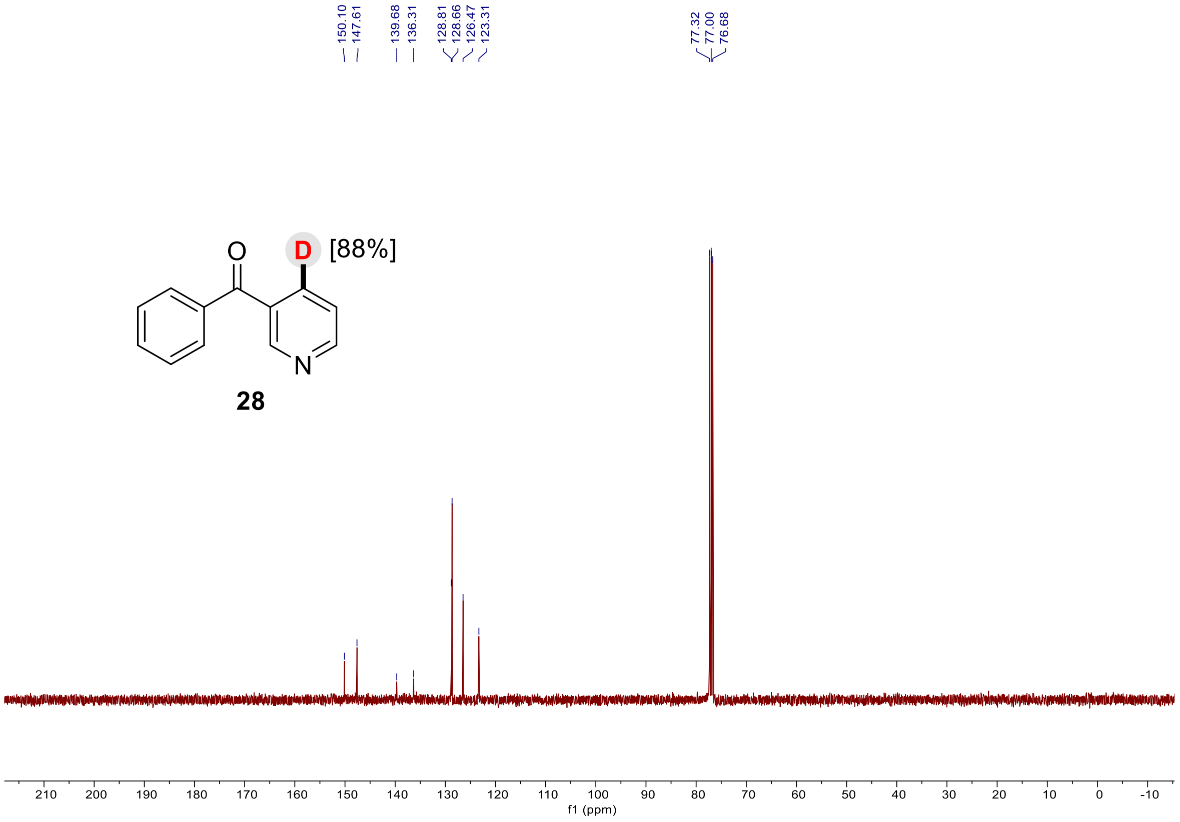

**$^{1}$H NMR** and **$^{13}$C NMR** of **29**

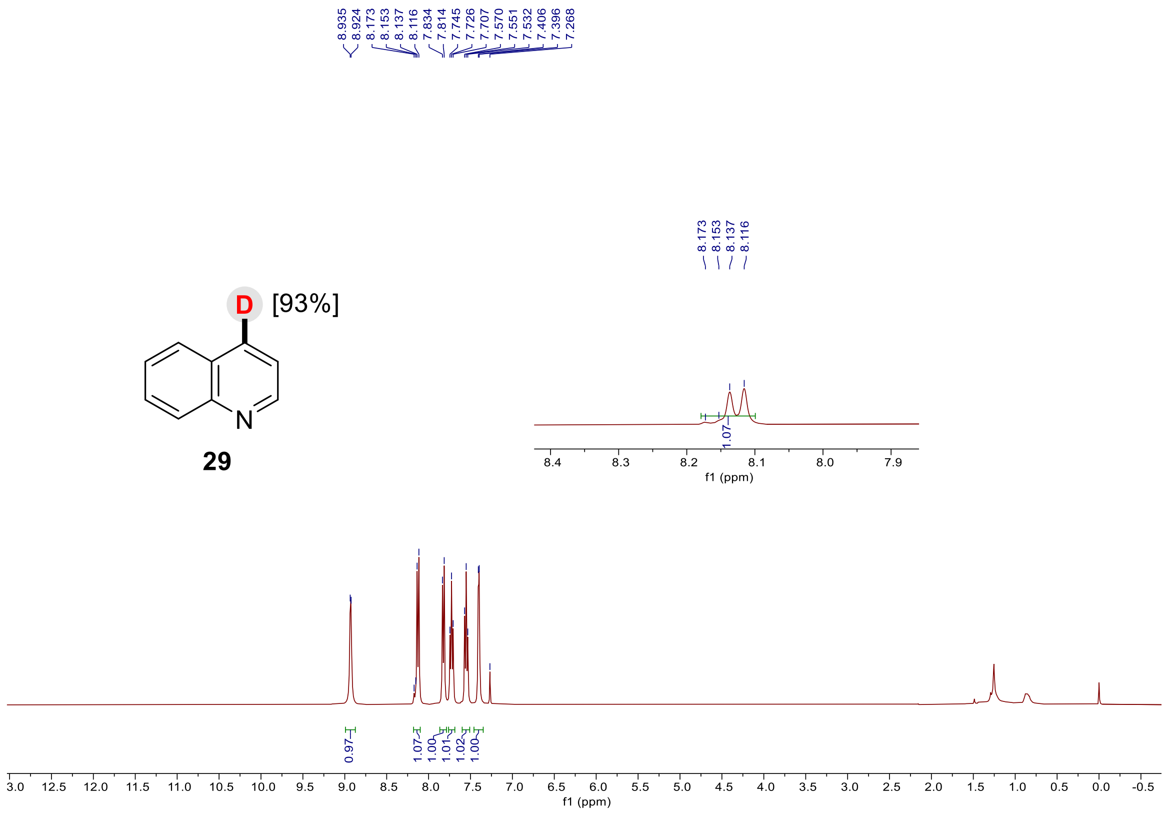


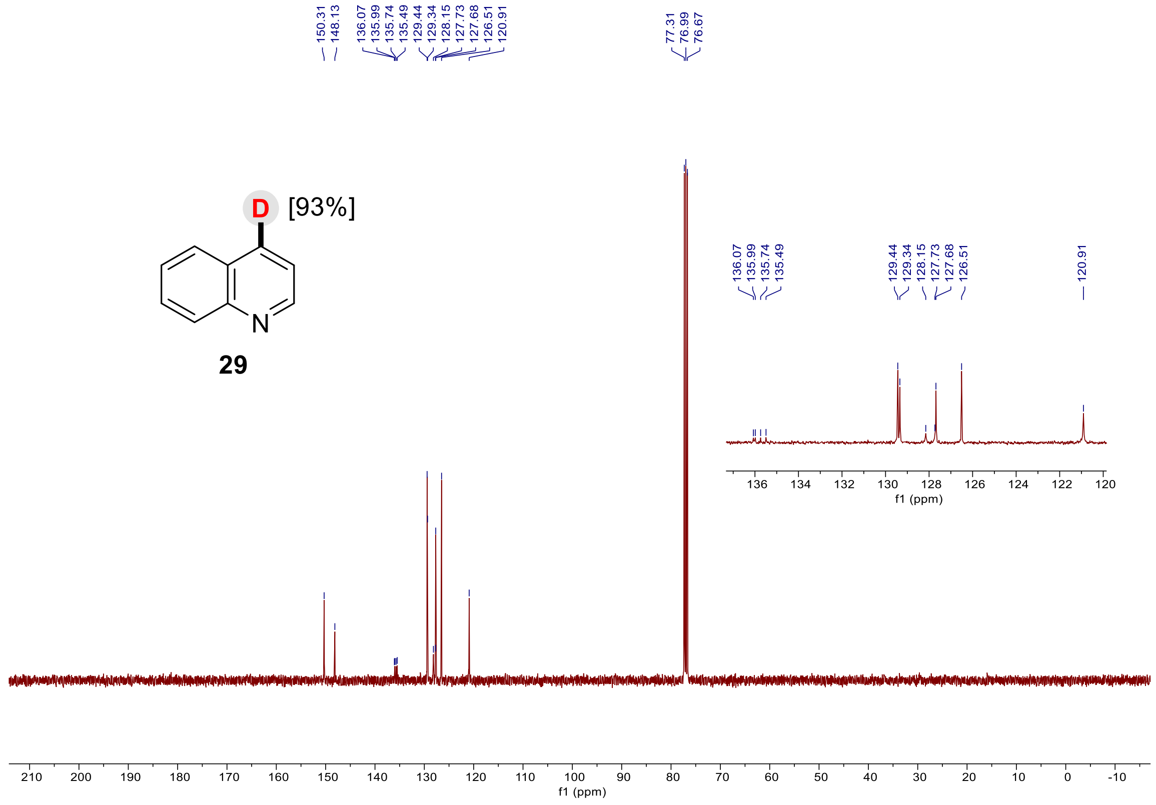

## $^{1}$H NMR and $^{13}$C NMR of **30**

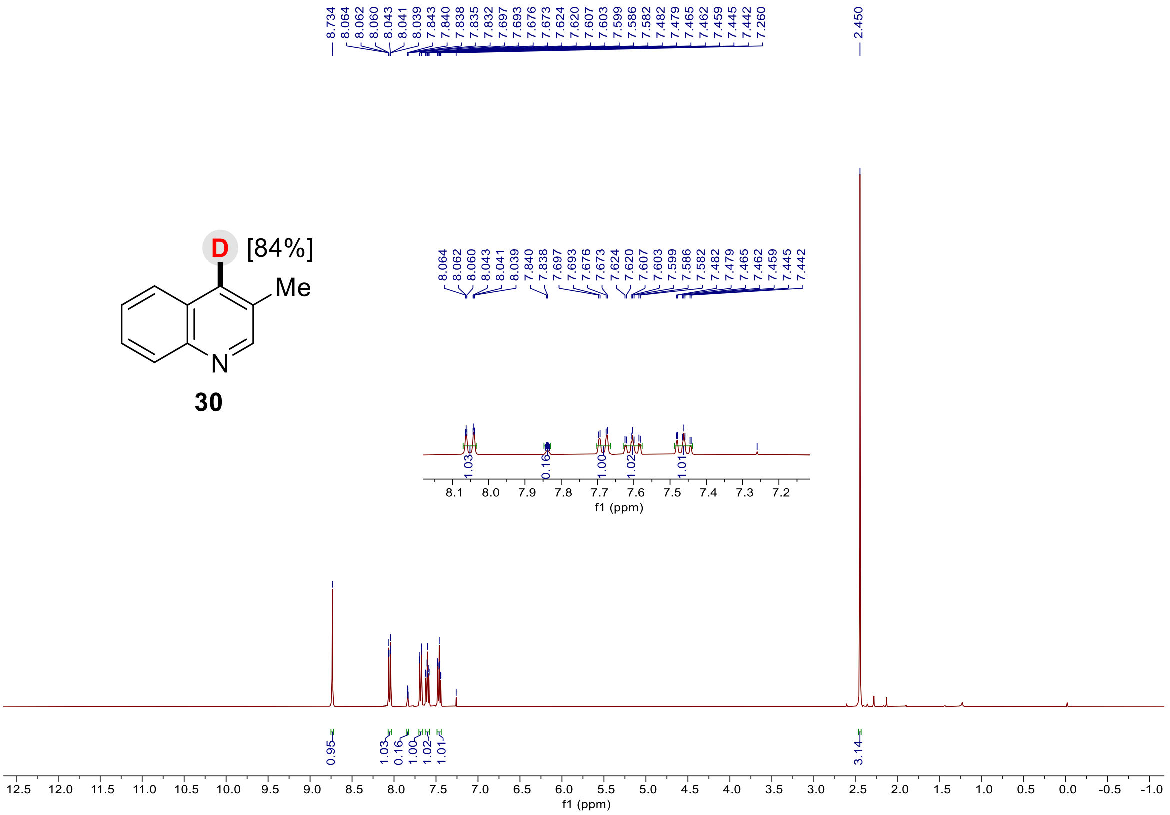


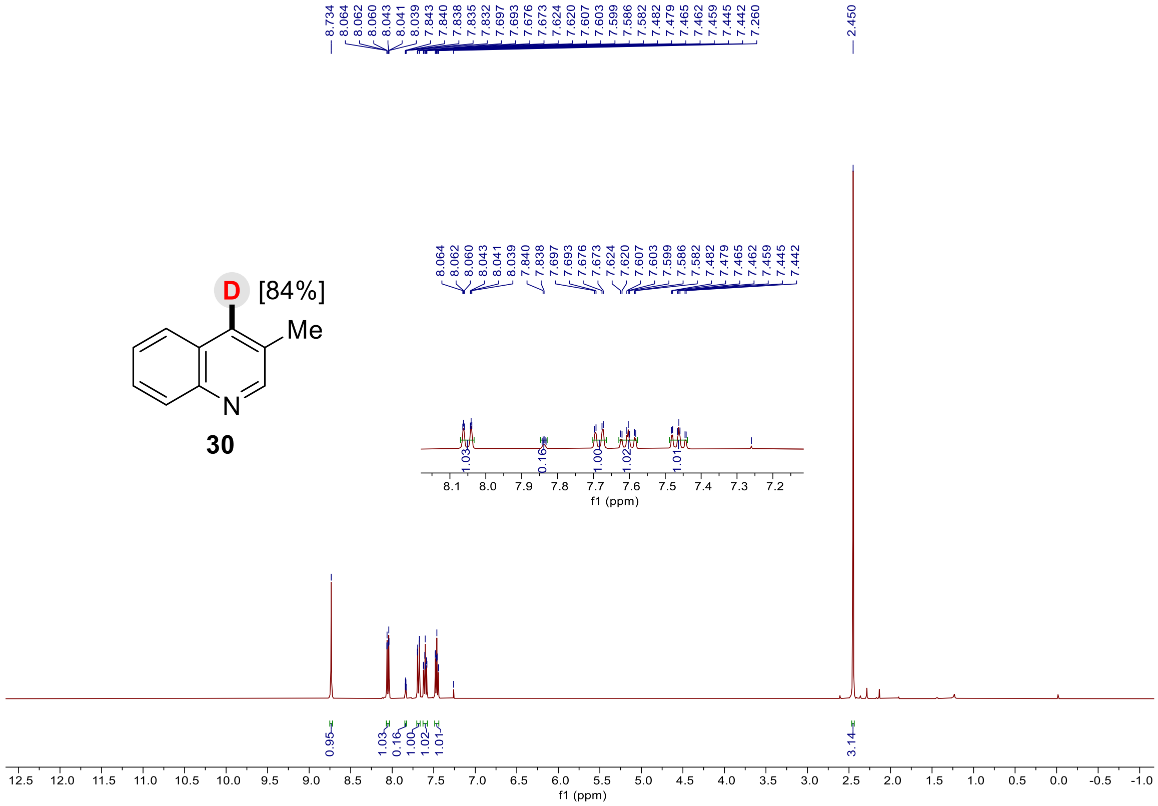

# $^{1}H$ NMR and $^{13}C$ NMR of **31**

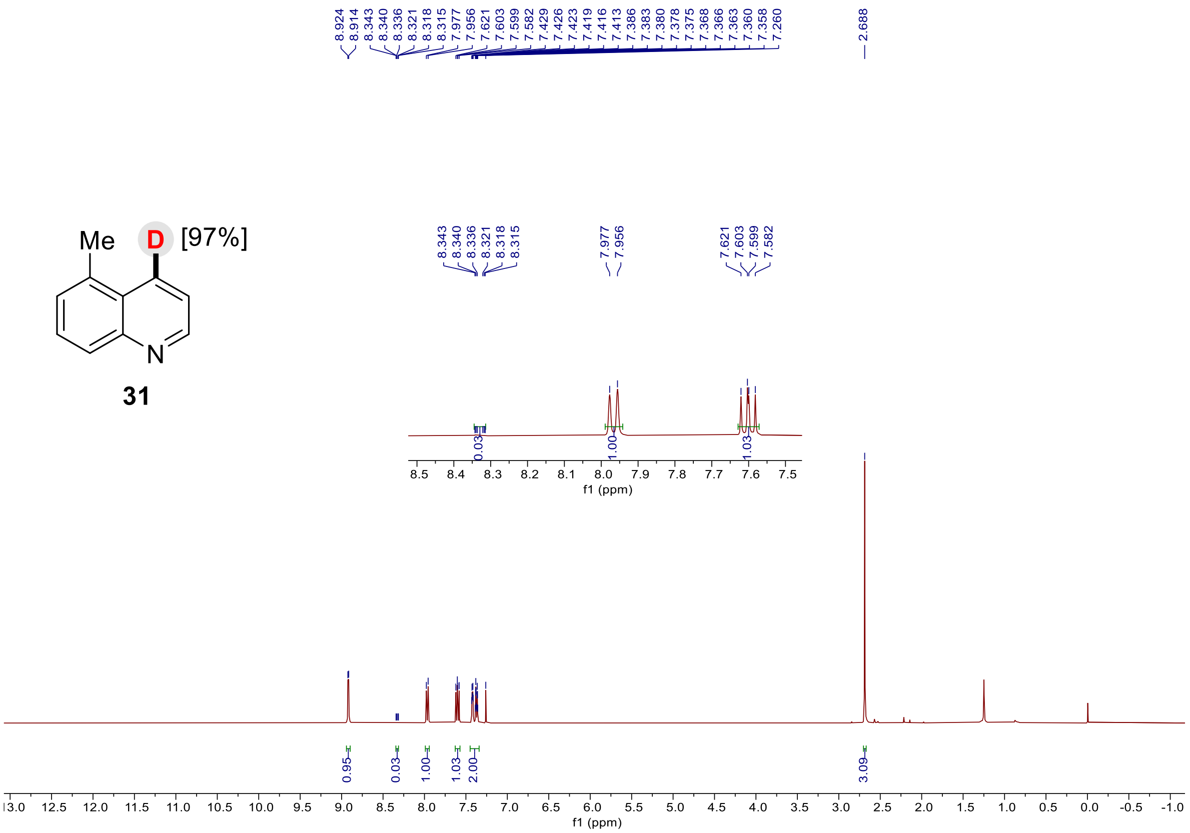


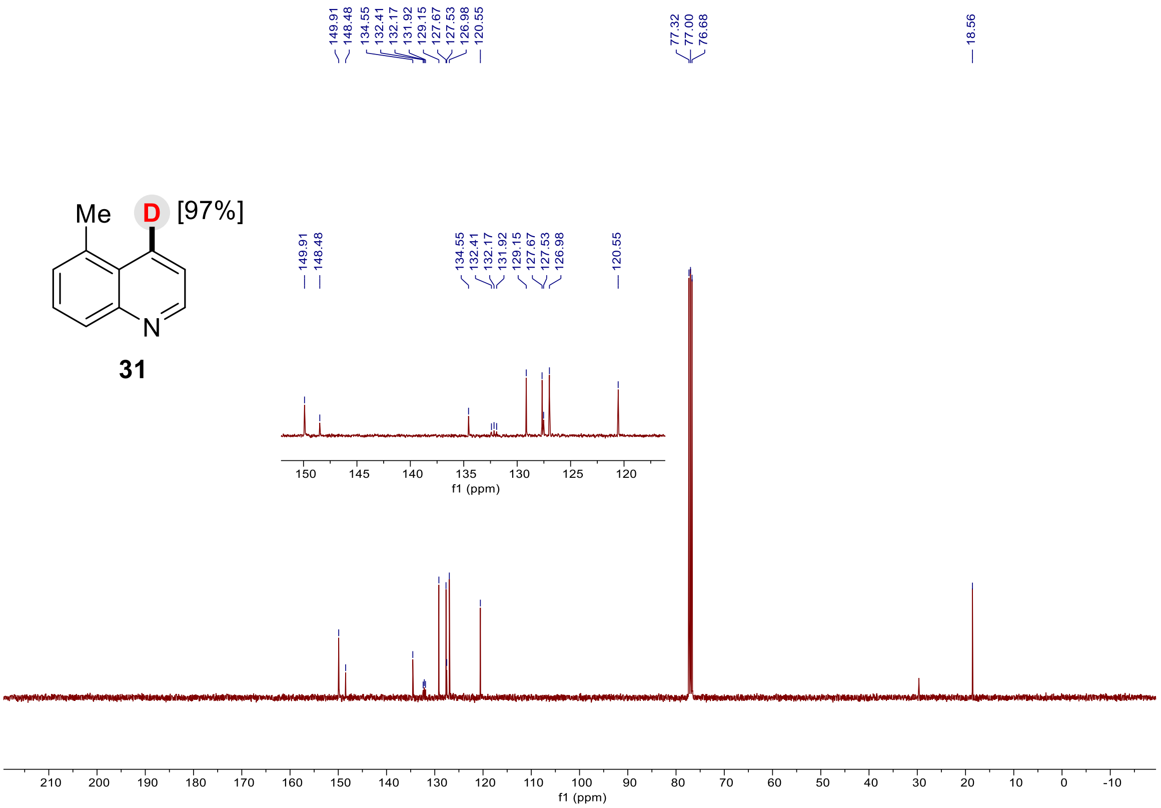

**$^{1}$H NMR** and **$^{13}$C NMR** of **32**

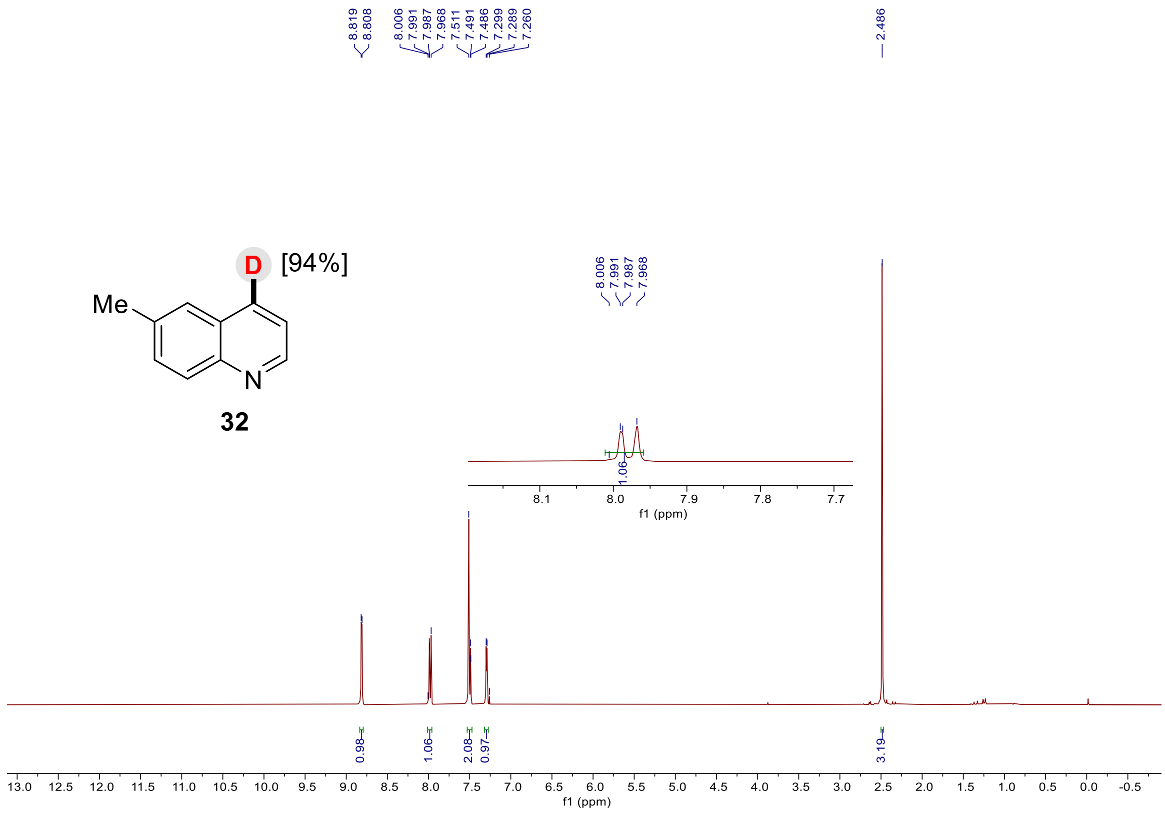


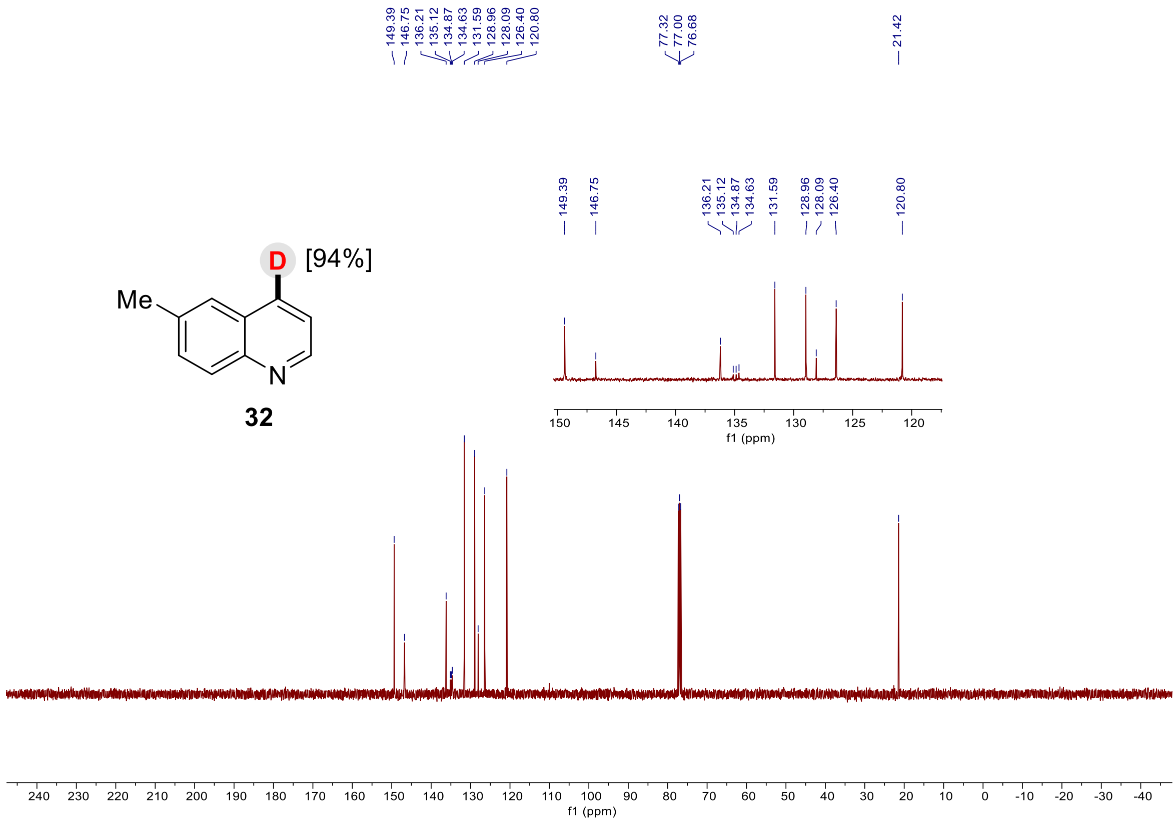

## $^{1}$H NMR and $^{13}$C NMR of 33

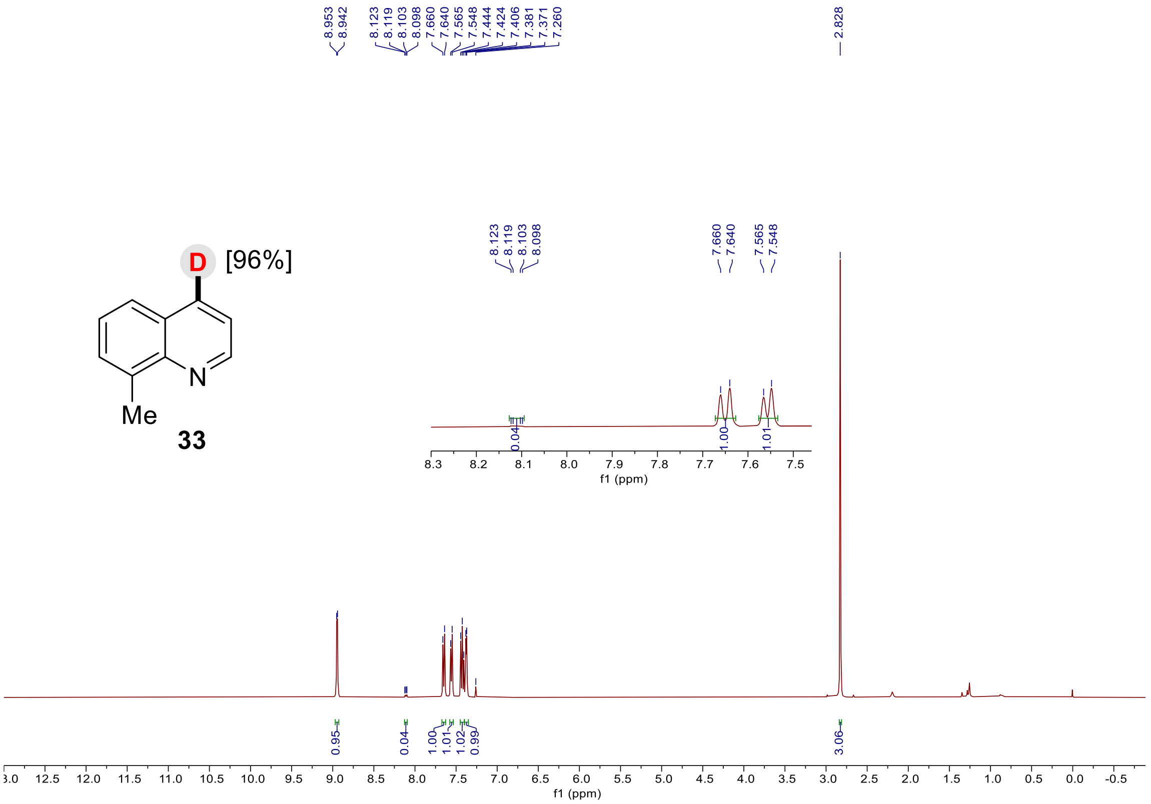


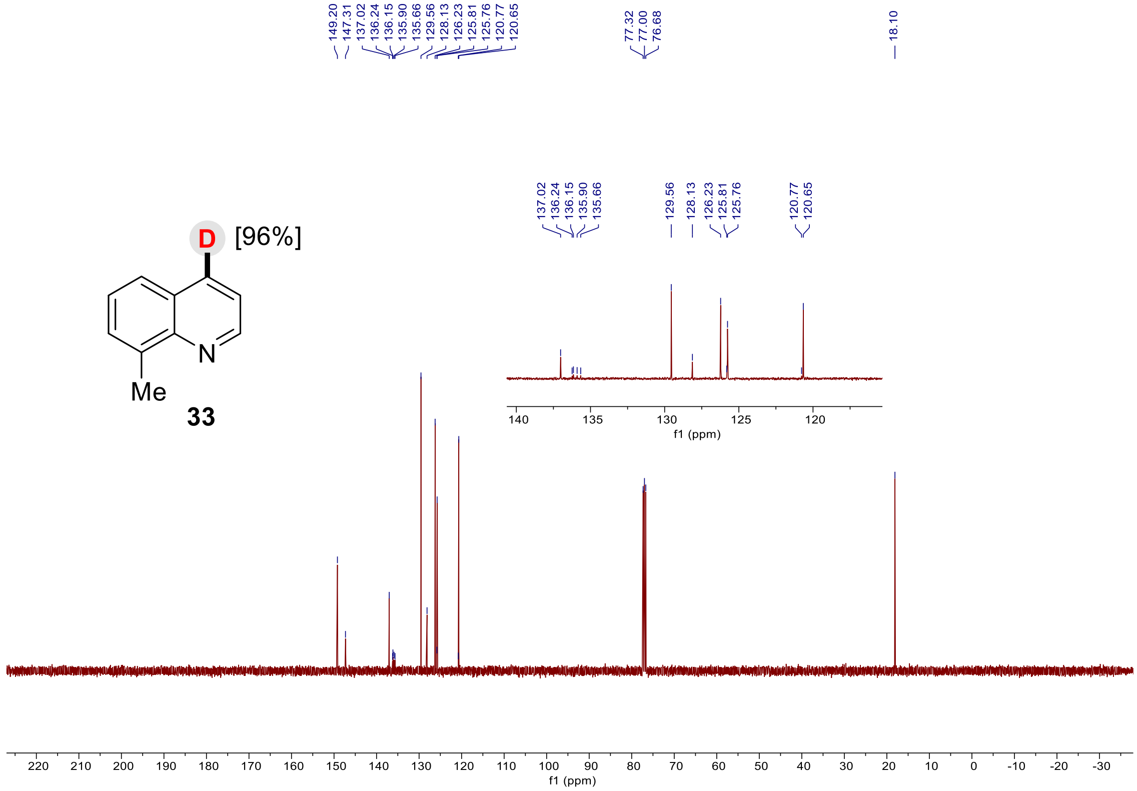

$^{1}$H NMR and $^{13}$C NMR of **34**

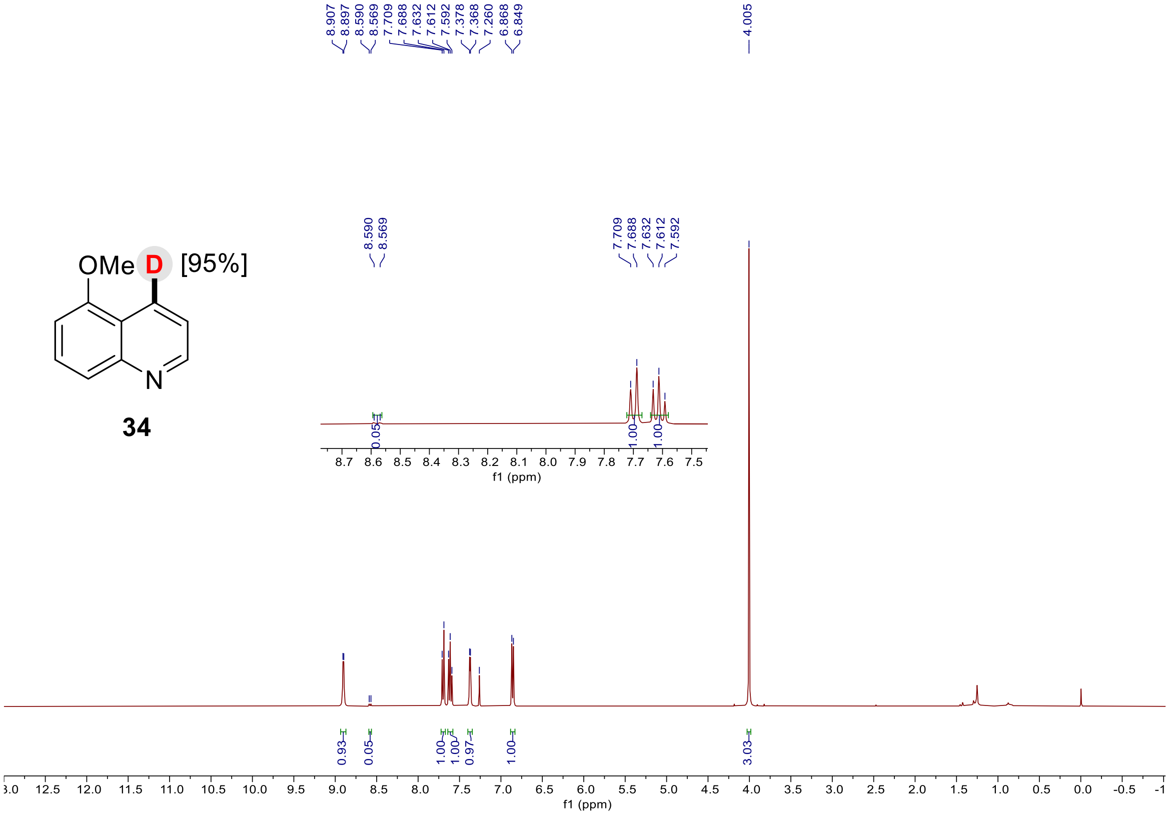


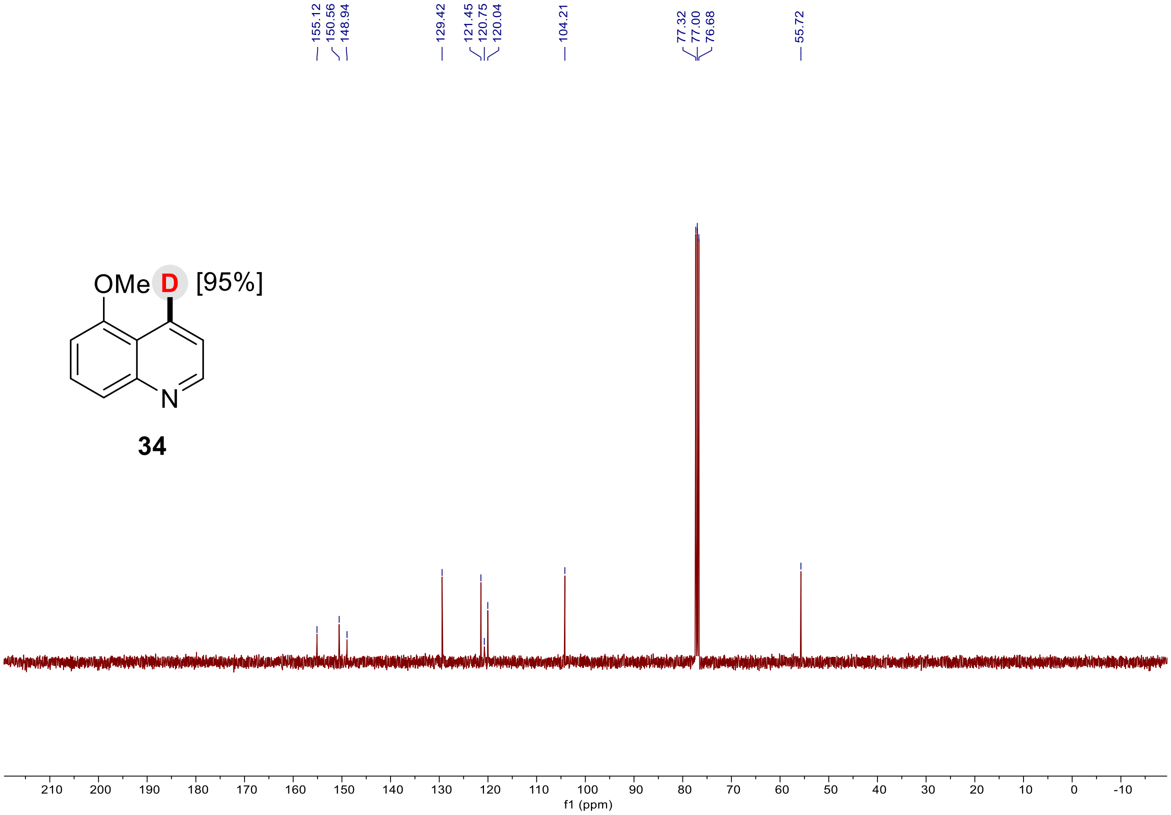

**$^{1}$H NMR** and **$^{13}$C NMR** of **35**

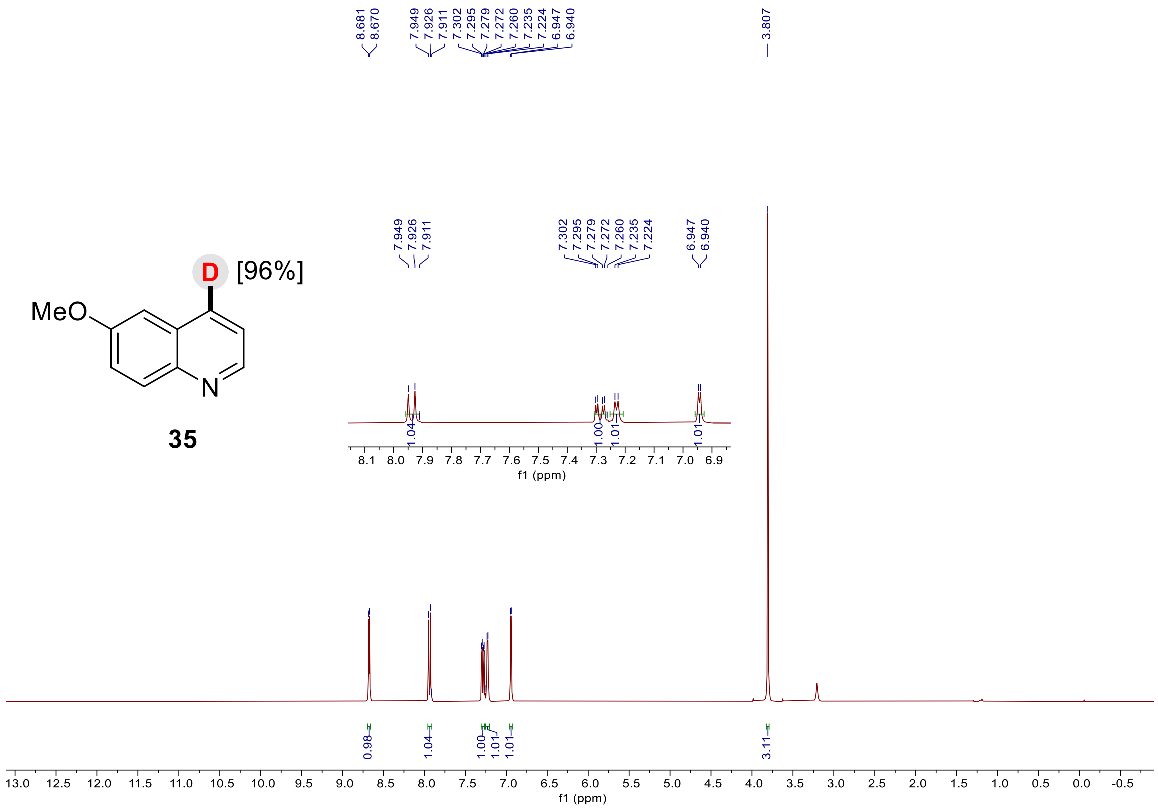


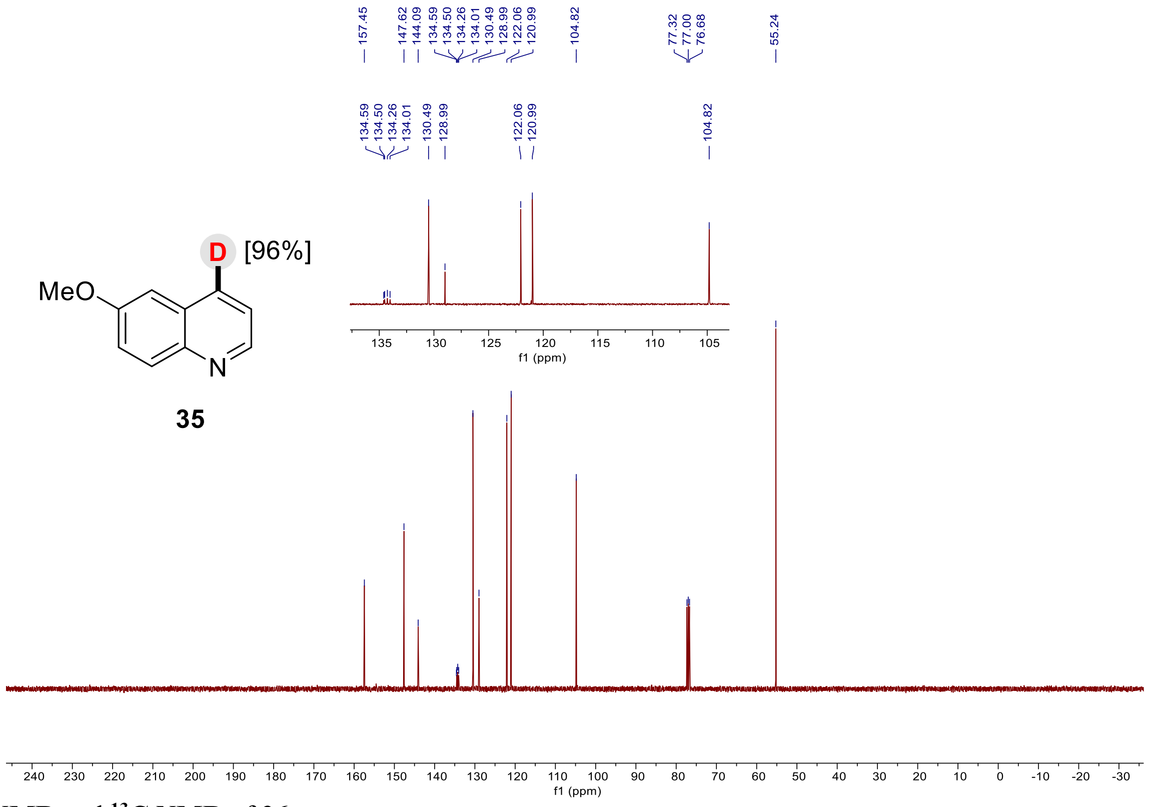


**$^{1}$H NMR** and **$^{13}$C NMR** of **36**

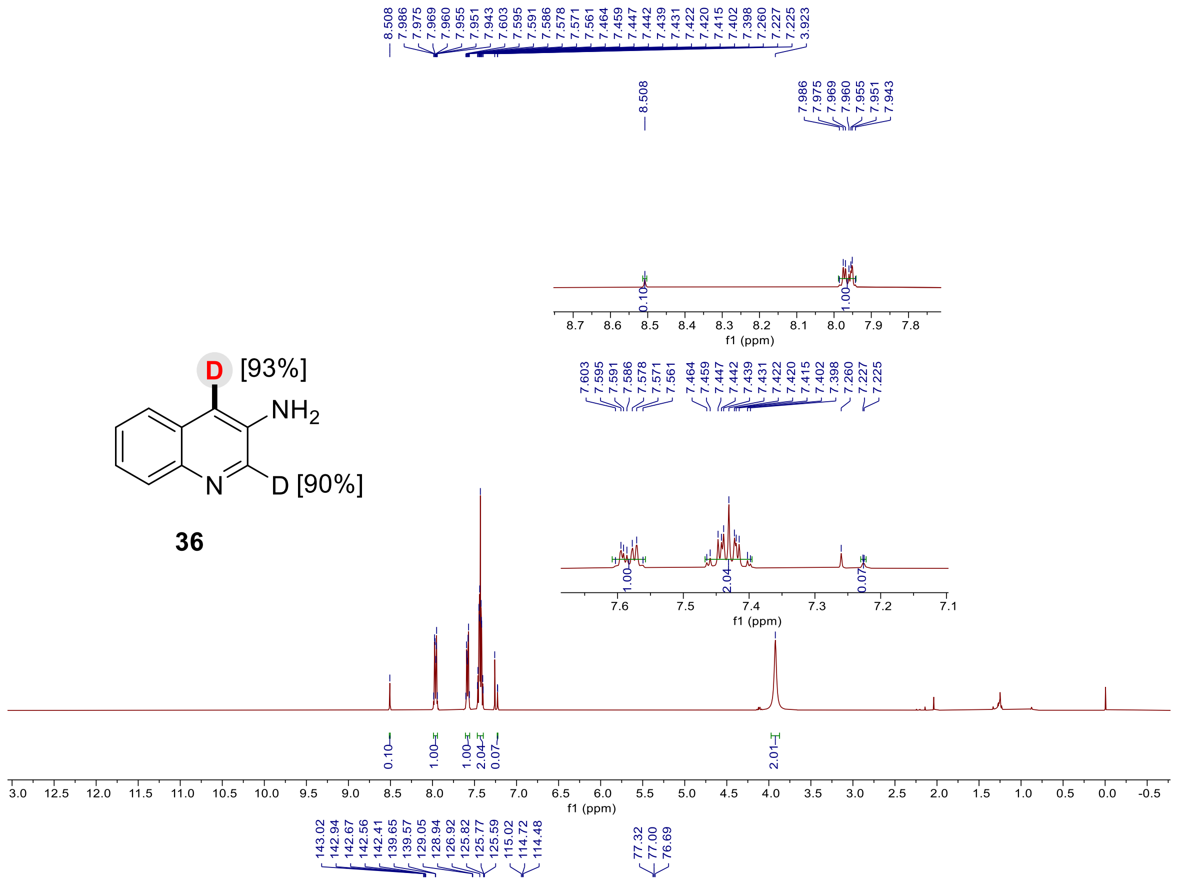


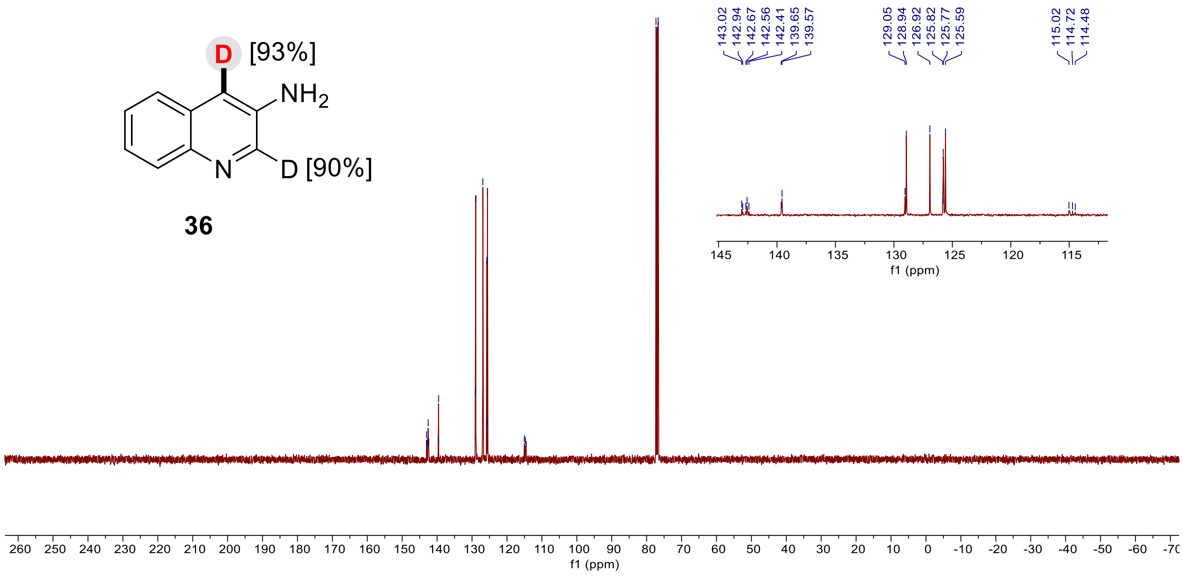


**$^{1}$H NMR** and **$^{13}$C NMR** of **37**

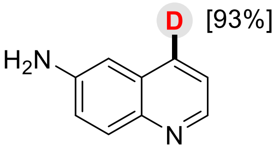


**37**

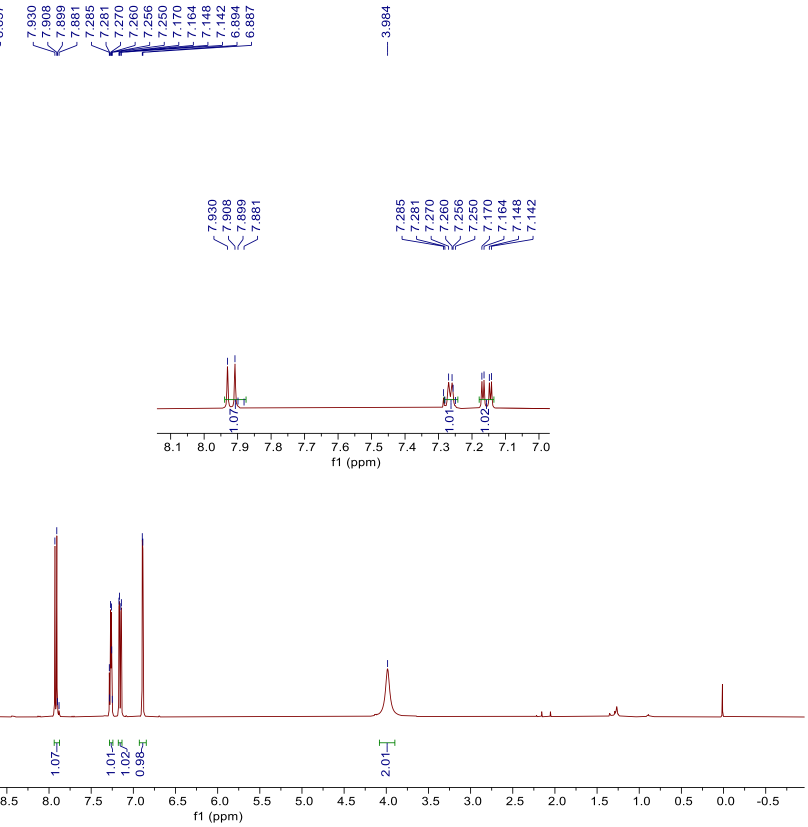


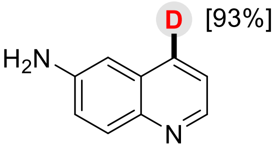


**37**

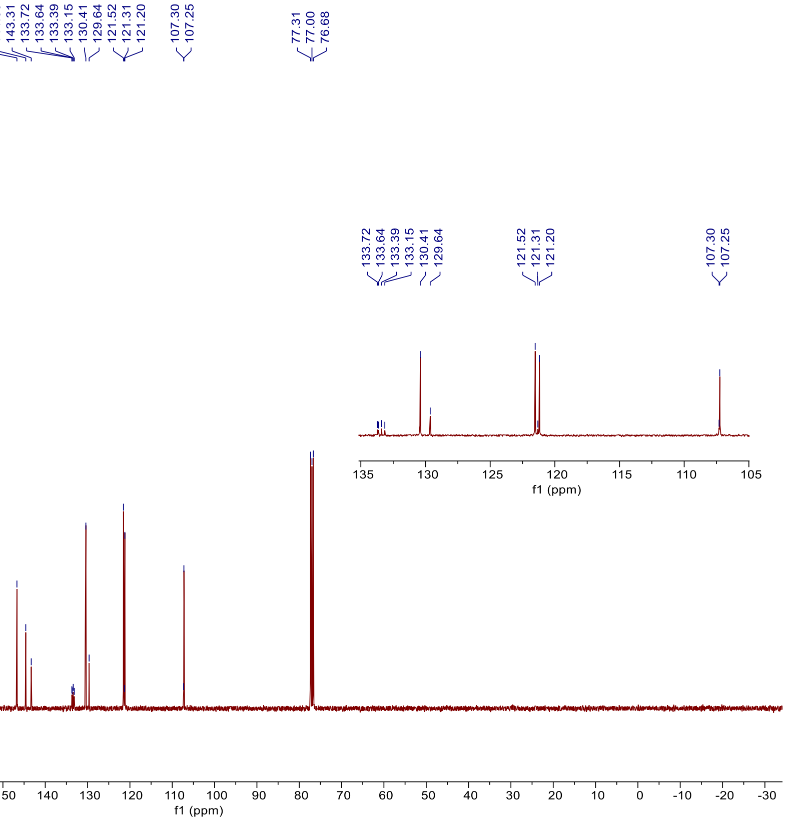


$^{1}$**H NMR** and $^{13}$**C NMR** of **38**

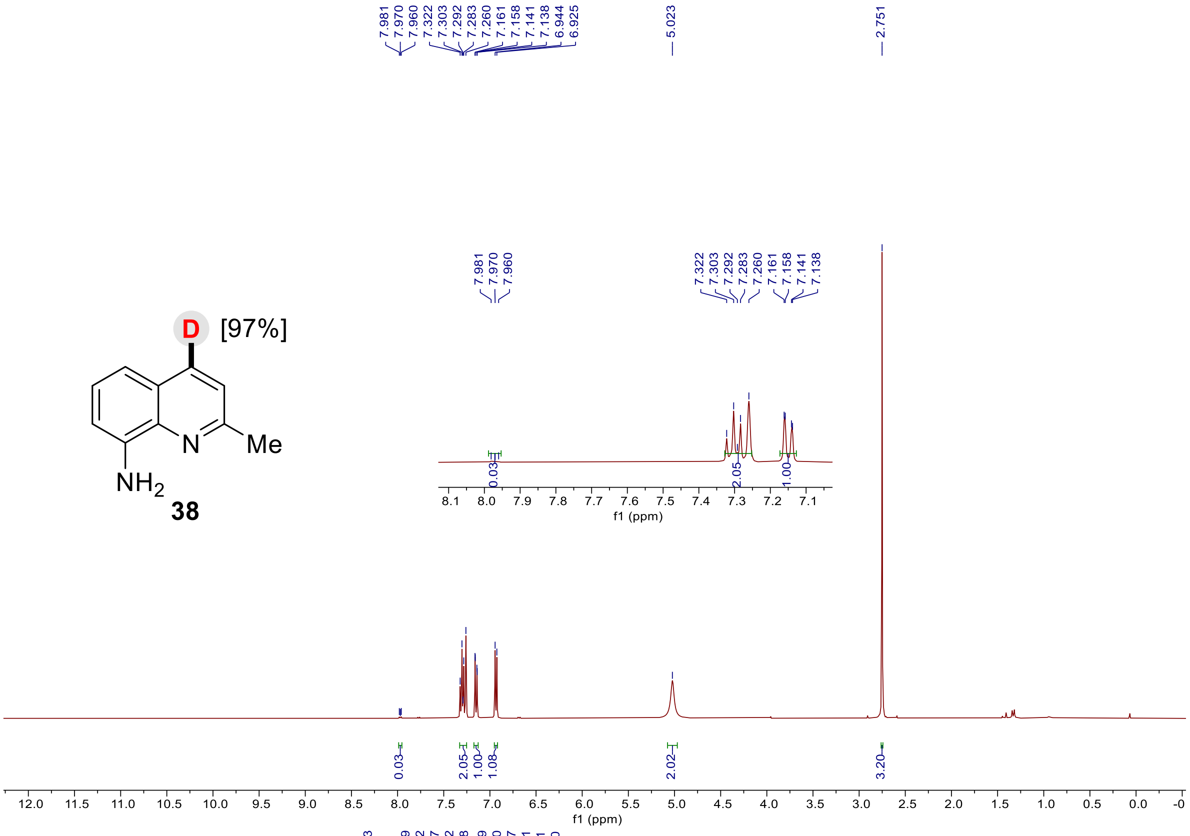

7.981
7.970
7.960
7.322
7.303
7.292
7.283
7.260
7.161
7.158
7.141
7.138
6.944
6.925
5.023
2.751
D [97%]
Me
N
NH2
38
0.03
2.05
1.00
f1 (ppm)
0.03
2.05
1.00
1.08
2.02
3.20
f1 (ppm)


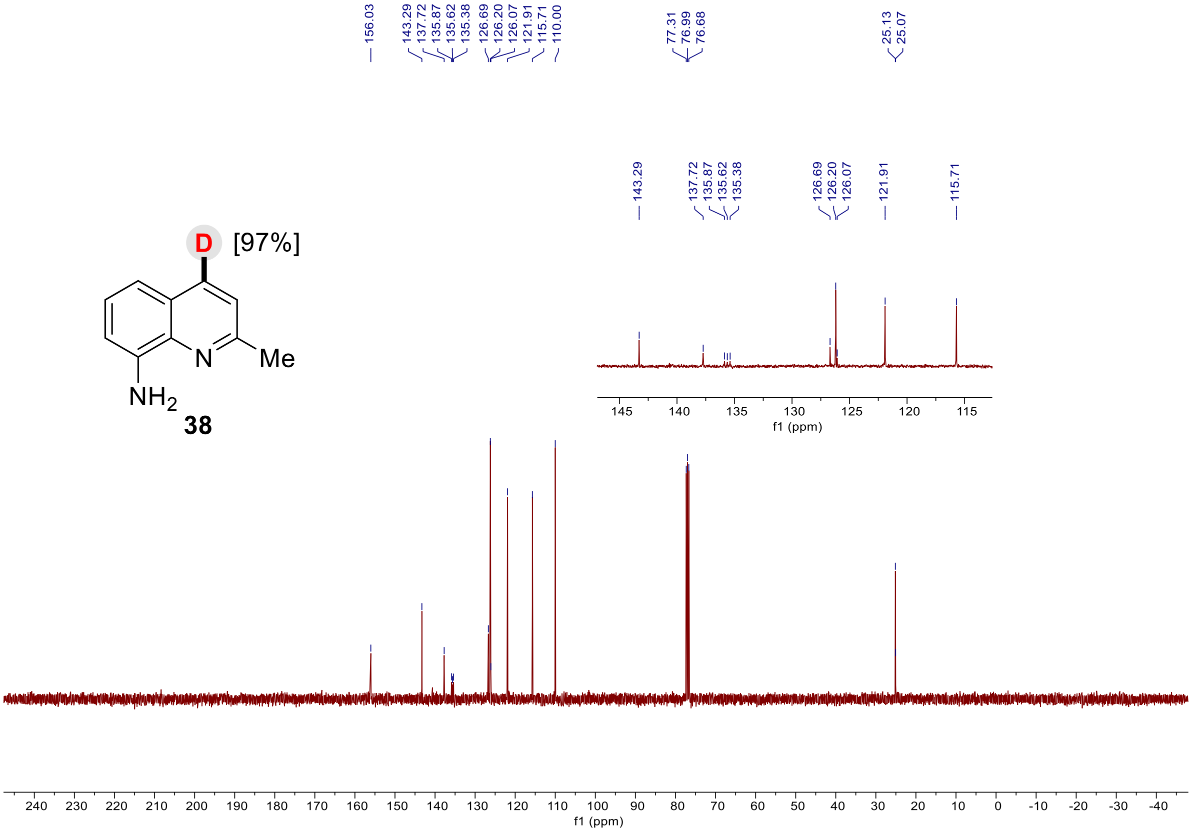

156.03
143.29
137.72
135.87
135.62
135.38
126.69
126.20
126.07
121.91
115.71
110.00
77.31
76.99
76.68
25.13
25.07
D [97%]
Me
N
NH2
38
f1 (ppm)
f1 (ppm)

**$^{1}$H NMR** and **$^{13}$C NMR** of **39**

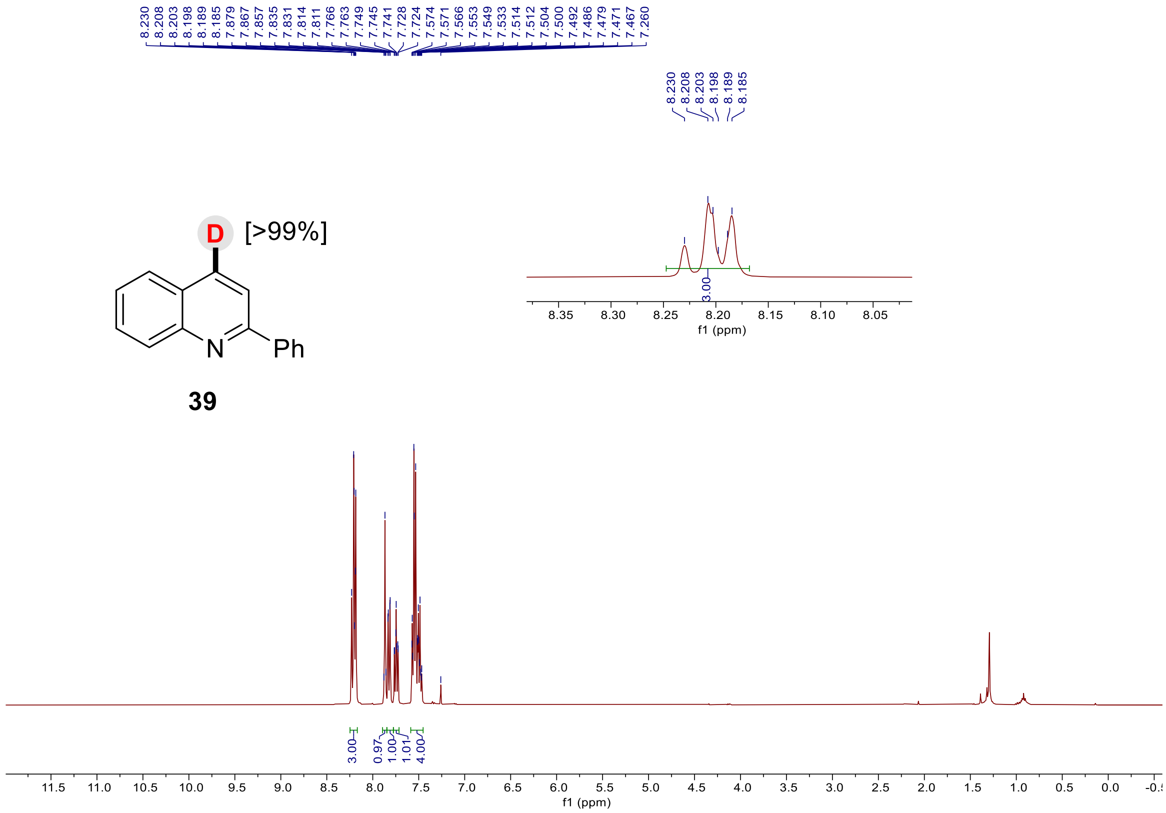


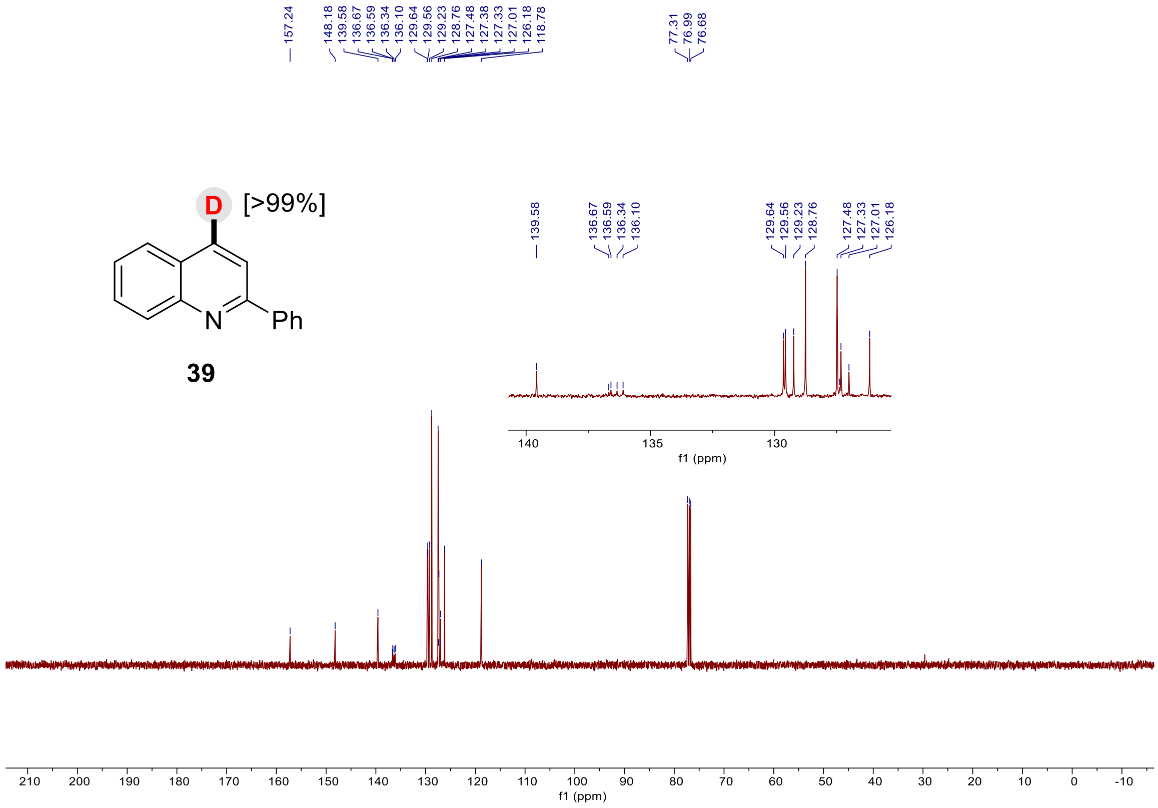

**$^{1}$H NMR** and **$^{13}$C NMR** of **40**

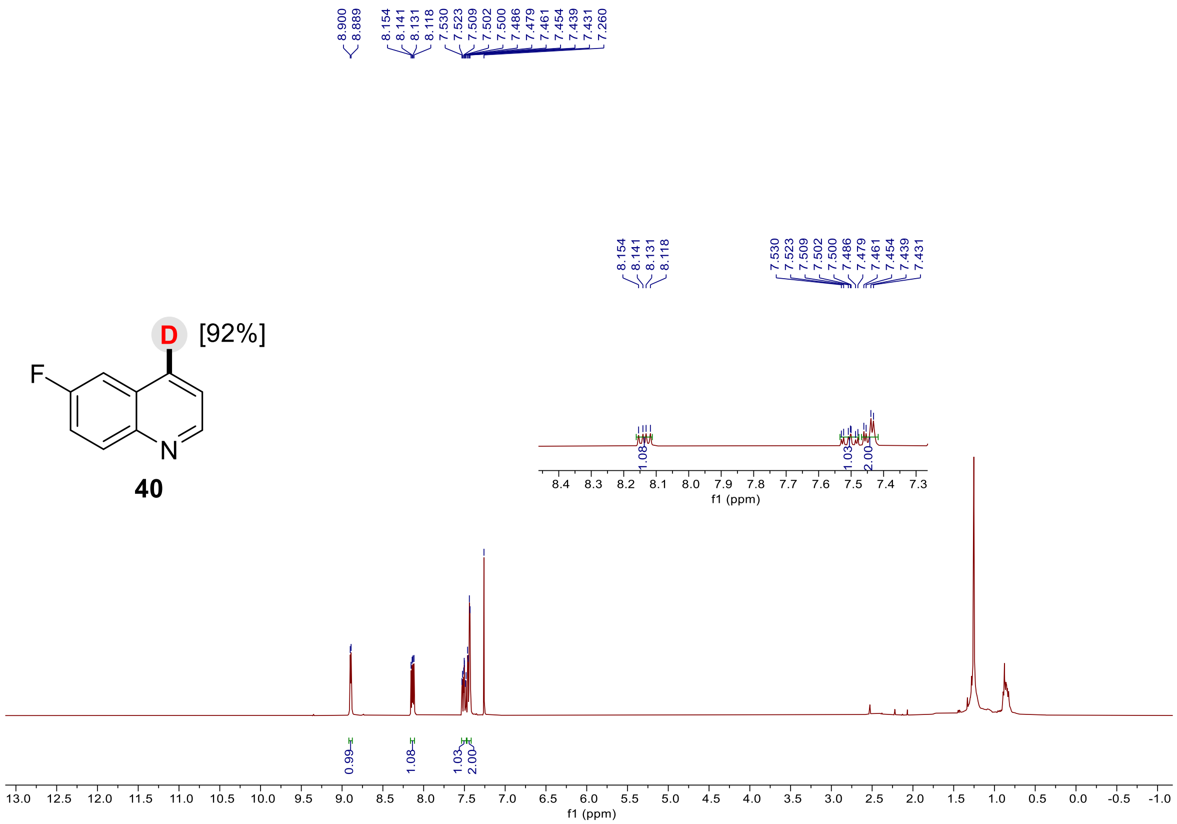


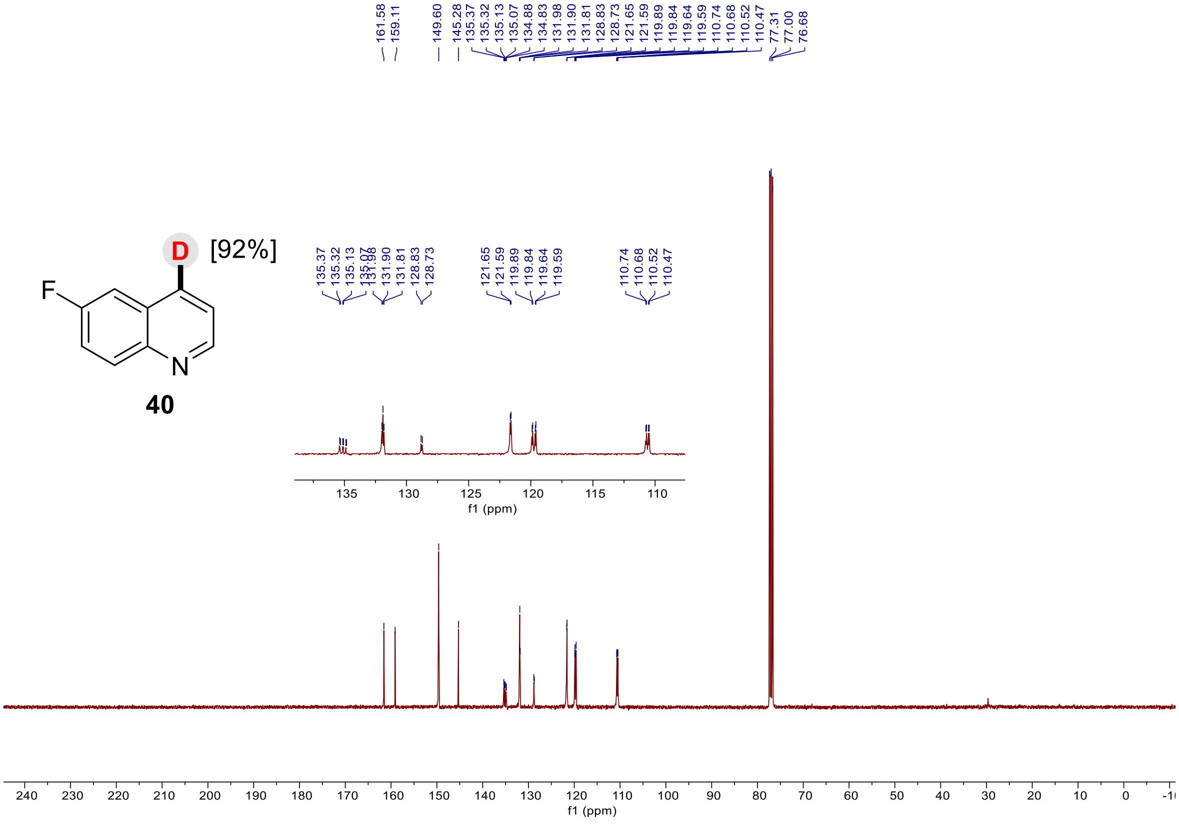

**$^{19}$F NMR** of **40**

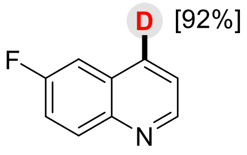


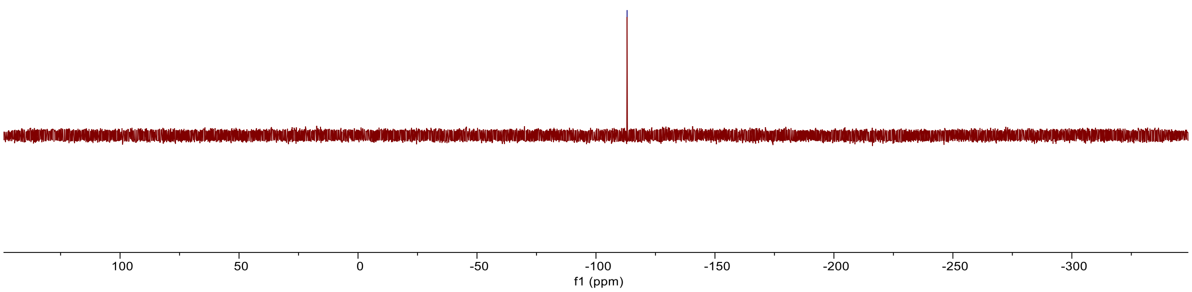

$^1H$ NMR and $^{13}C$ NMR of **41**

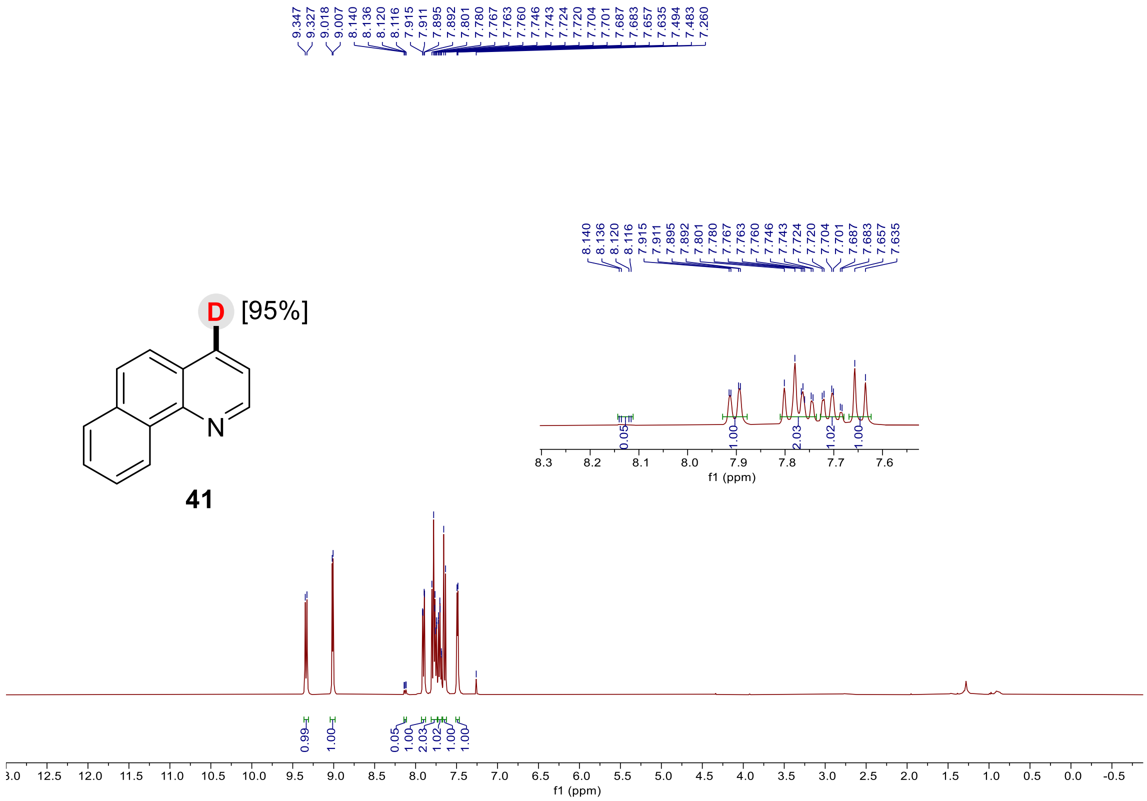


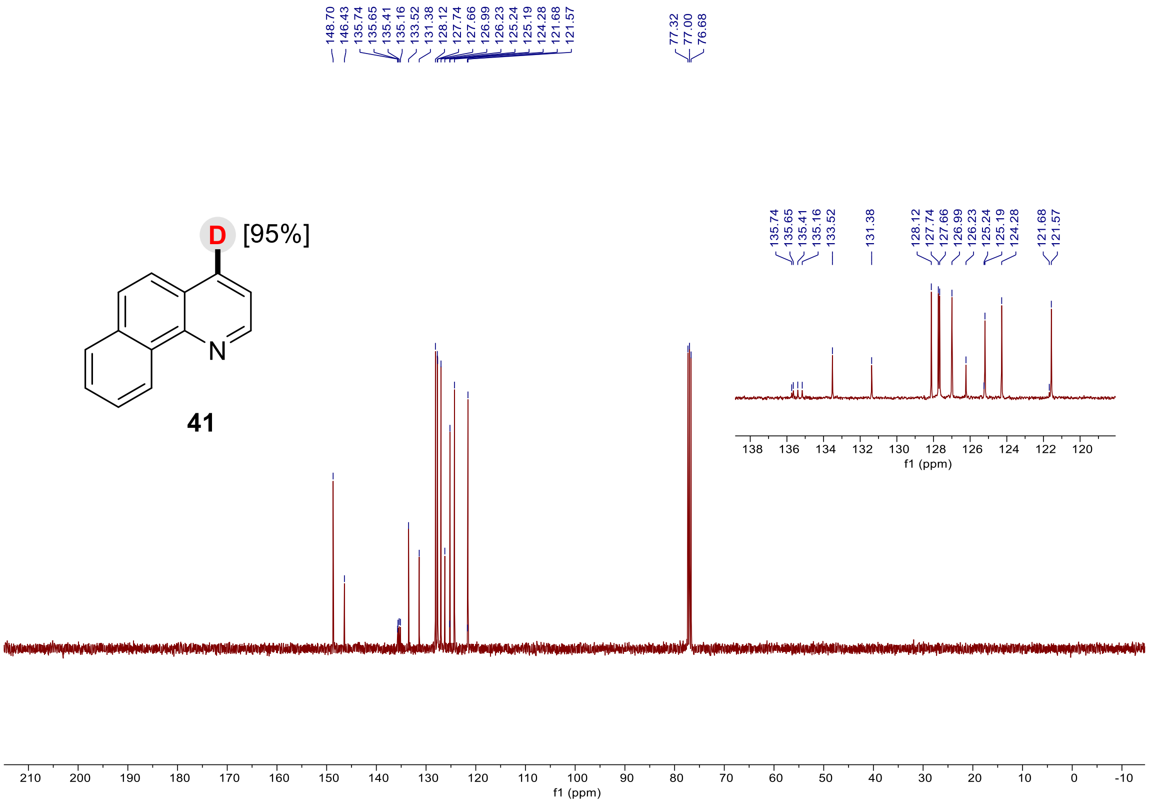

**$^{1}$H NMR** and **$^{13}$C NMR** of **42**

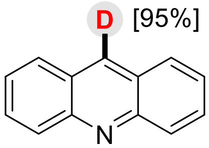


**42**

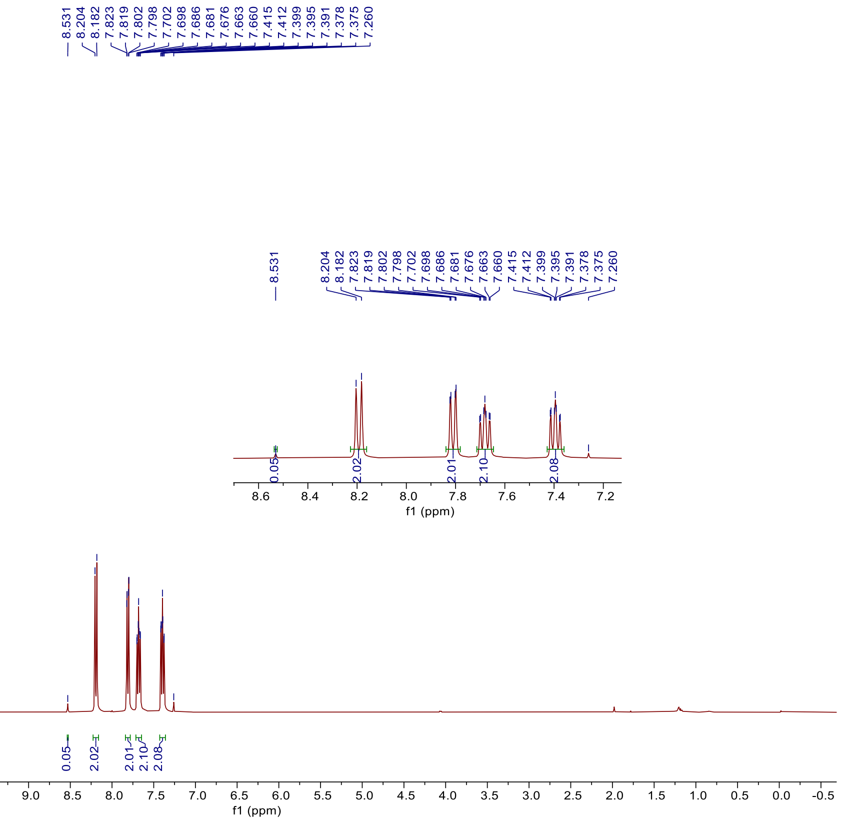


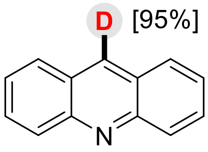


**42**

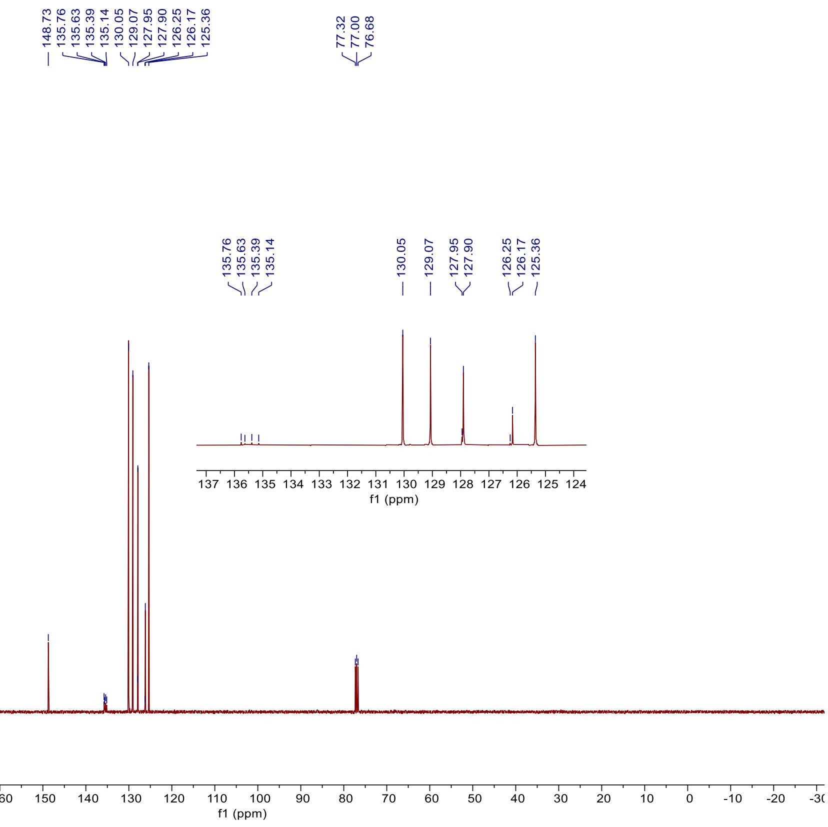

**$^{1}$H NMR** and **$^{13}$C NMR** of **43**

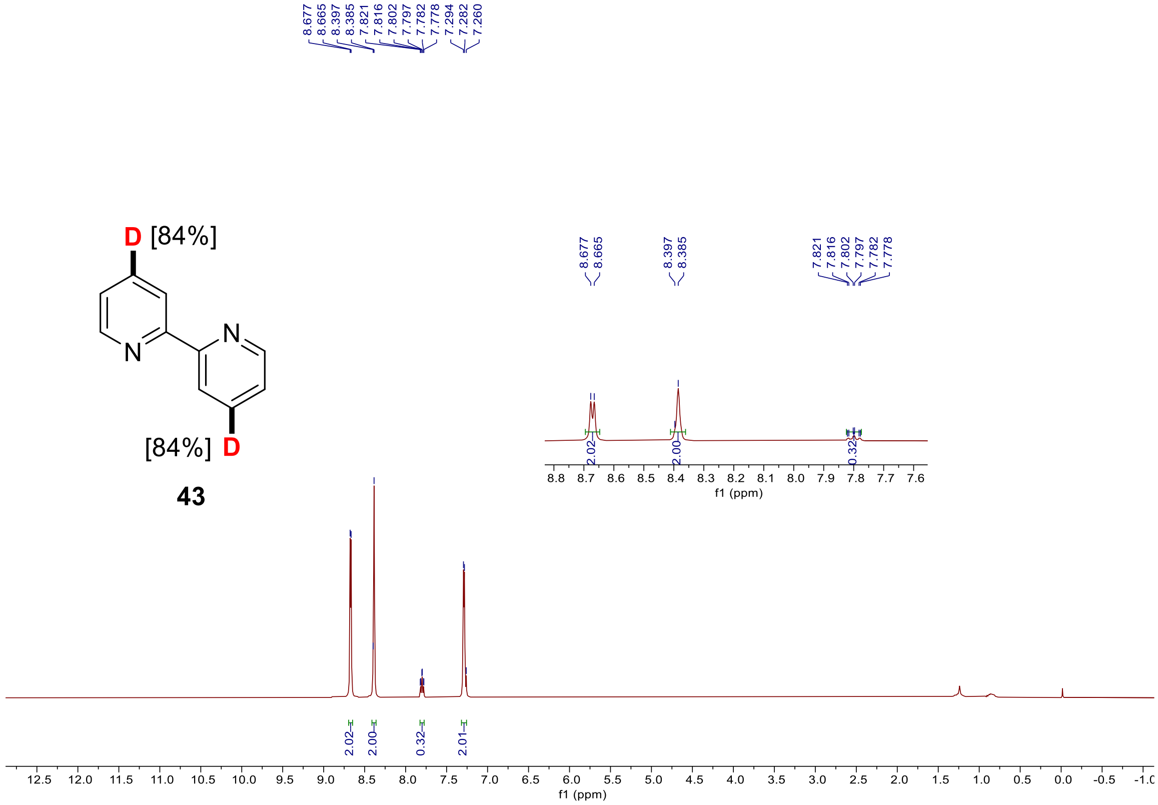


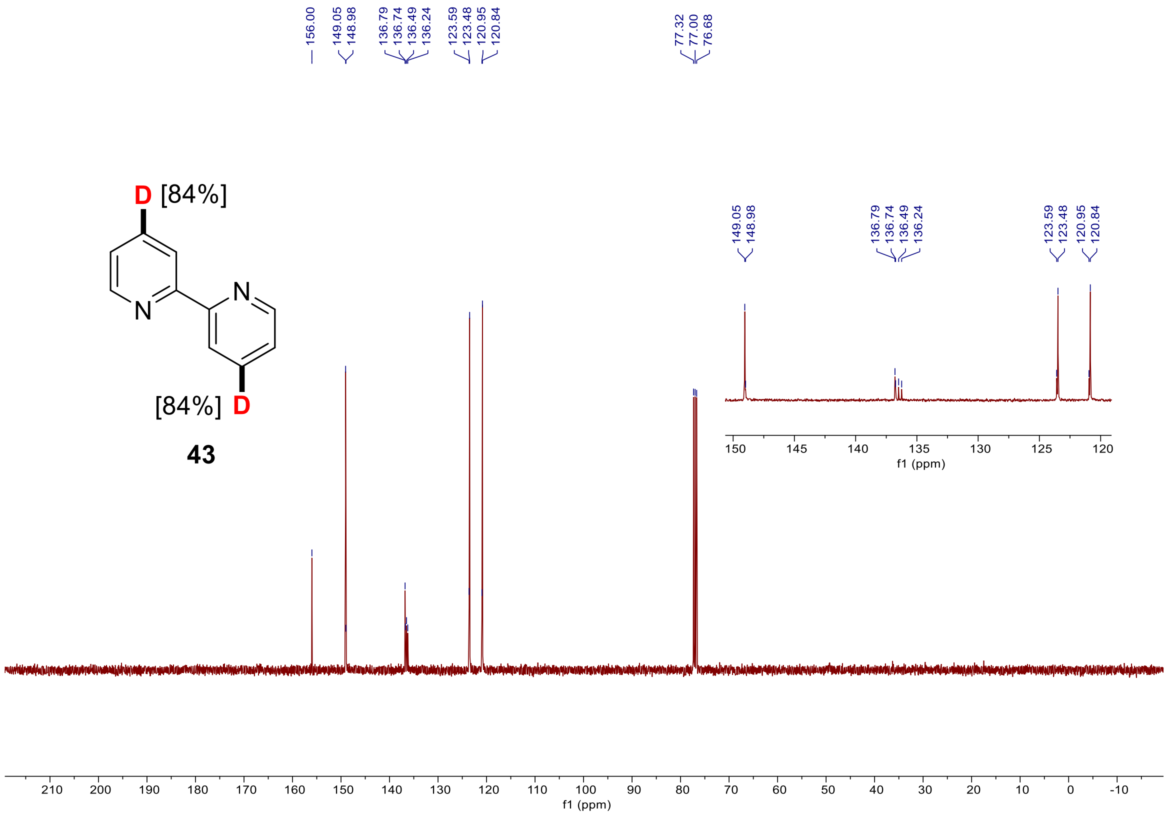

**$^{1}$H NMR** and **$^{13}$C NMR** of **44**

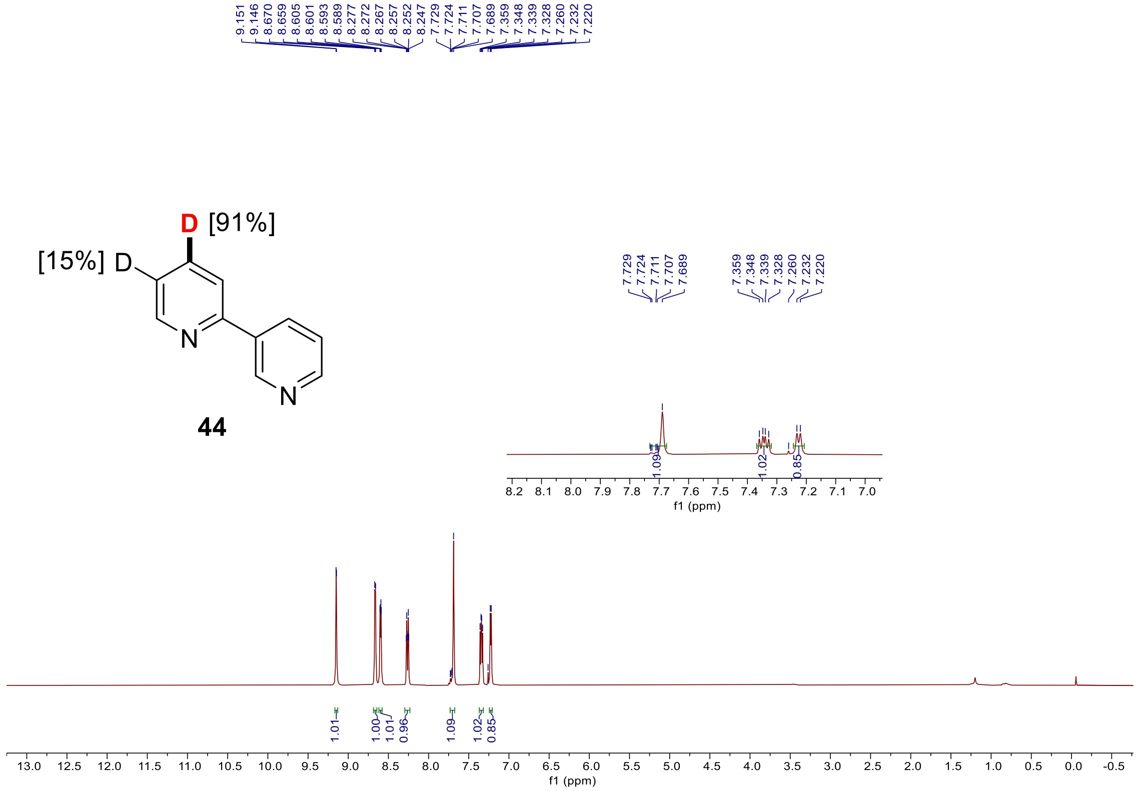


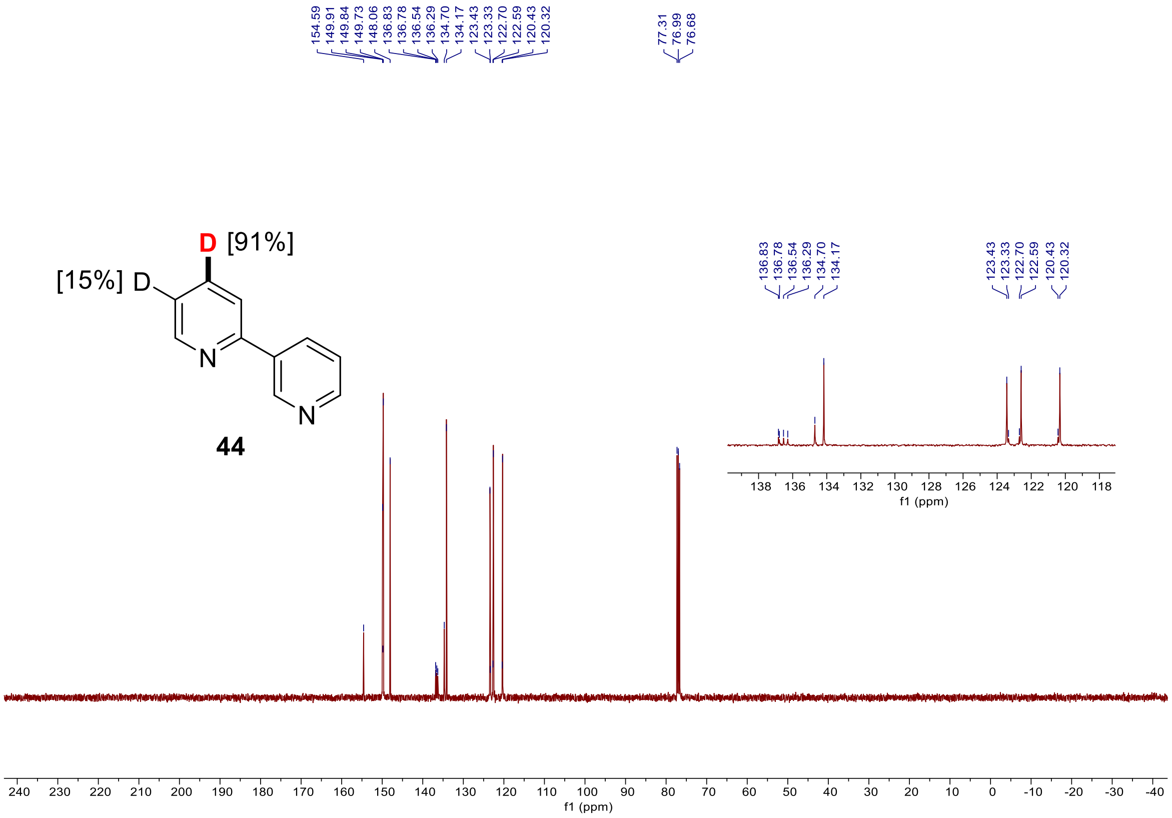

**$^{1}$H NMR and $^{13}$C NMR of 45**

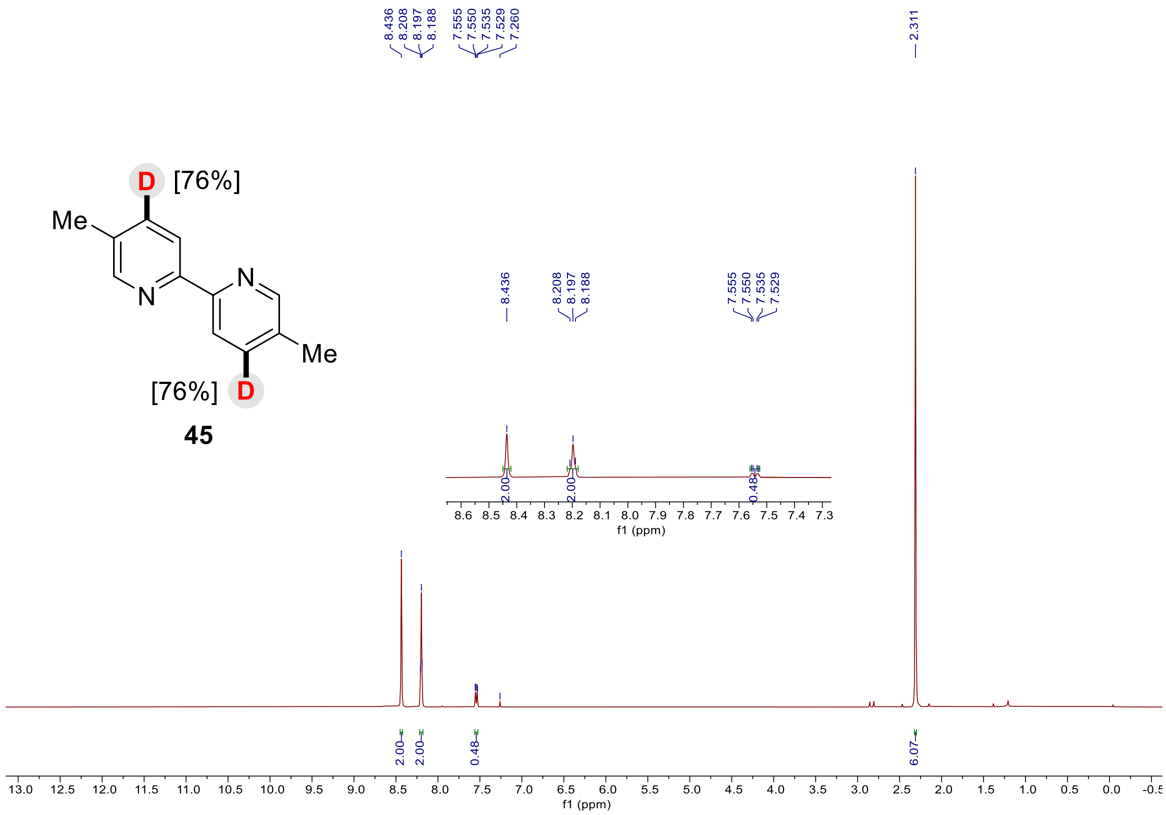


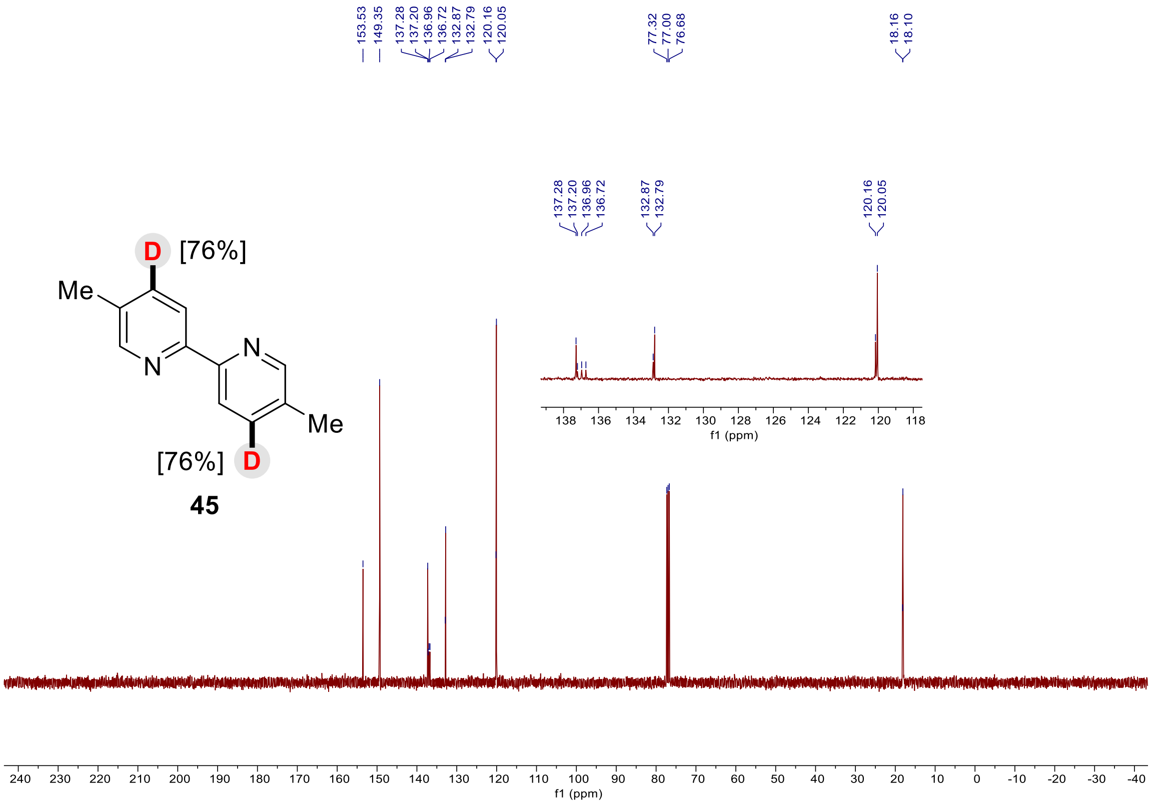

**$^{1}$H NMR** and **$^{13}$C NMR** of **46**

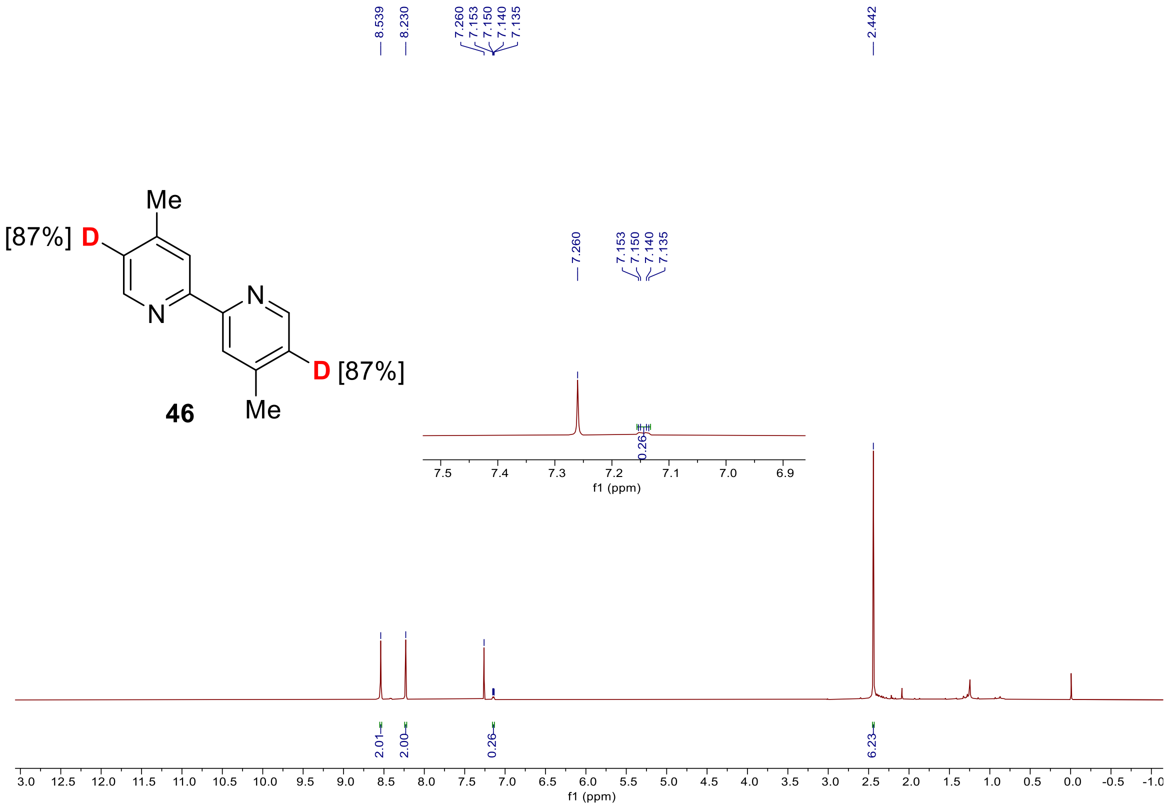


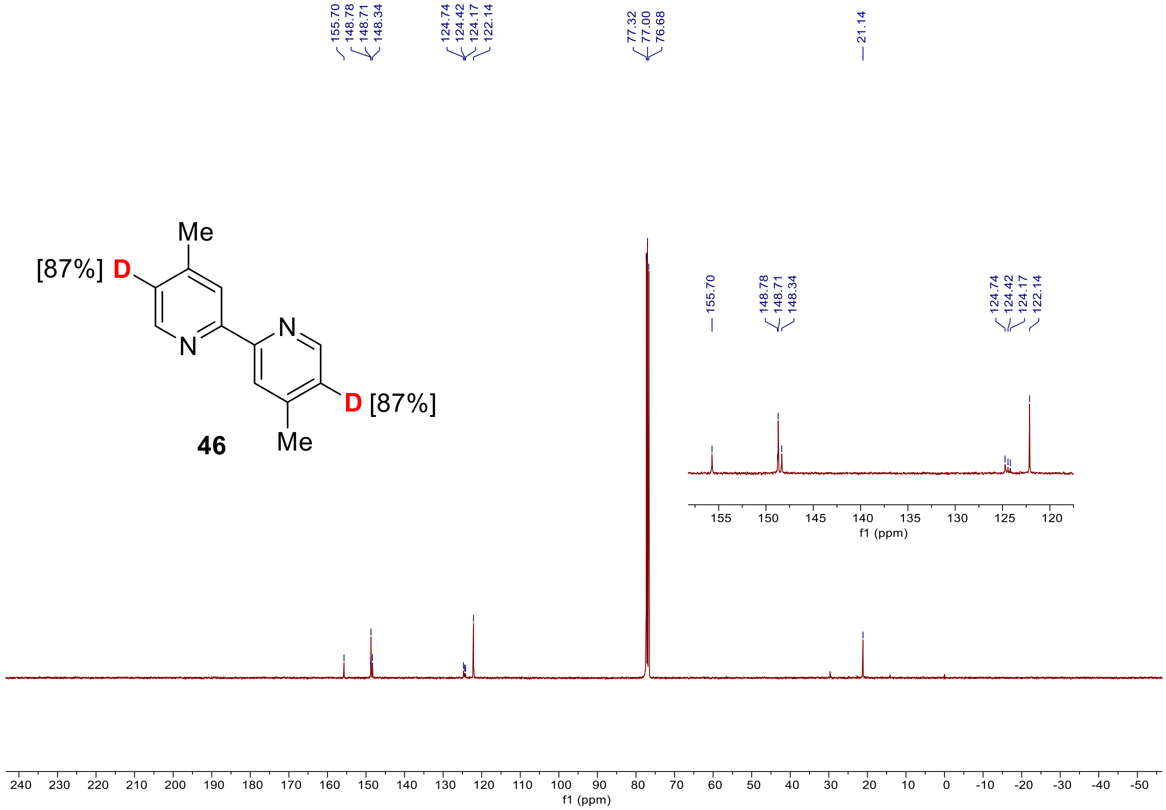

$^{1}H$ NMR and $^{13}C$ NMR of **47**

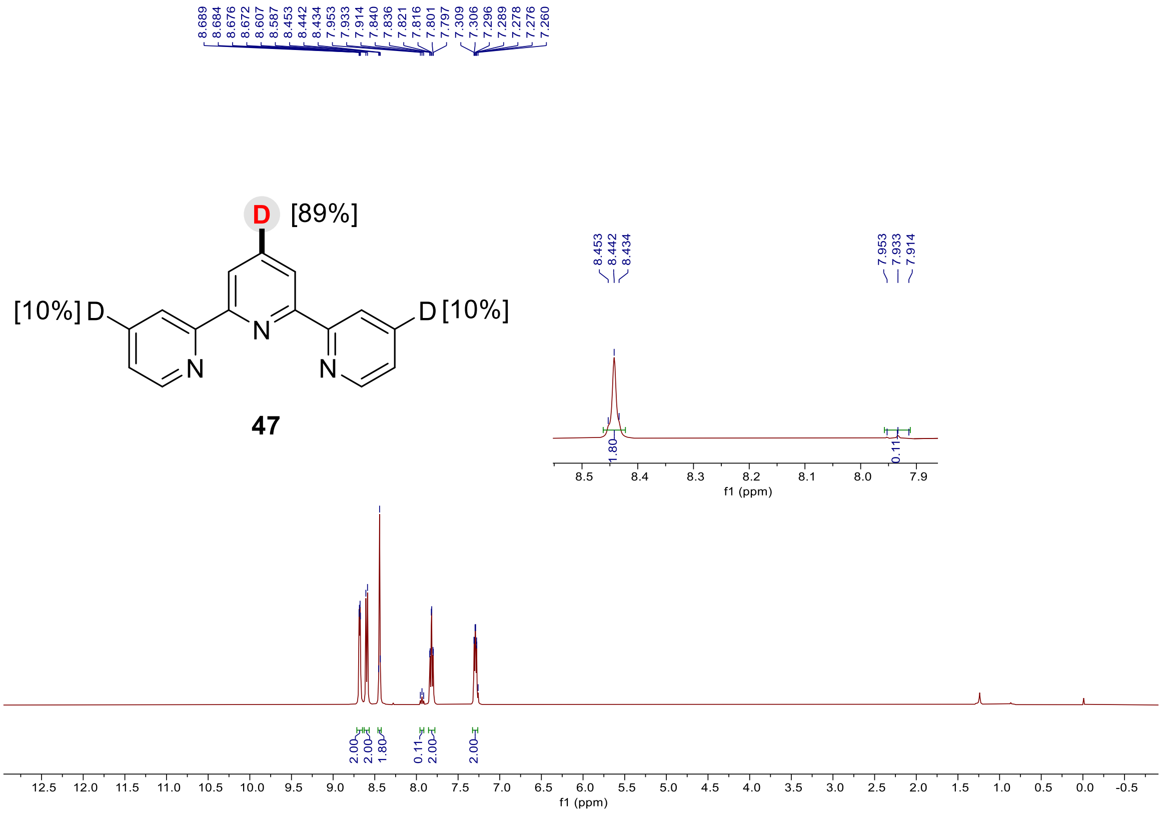


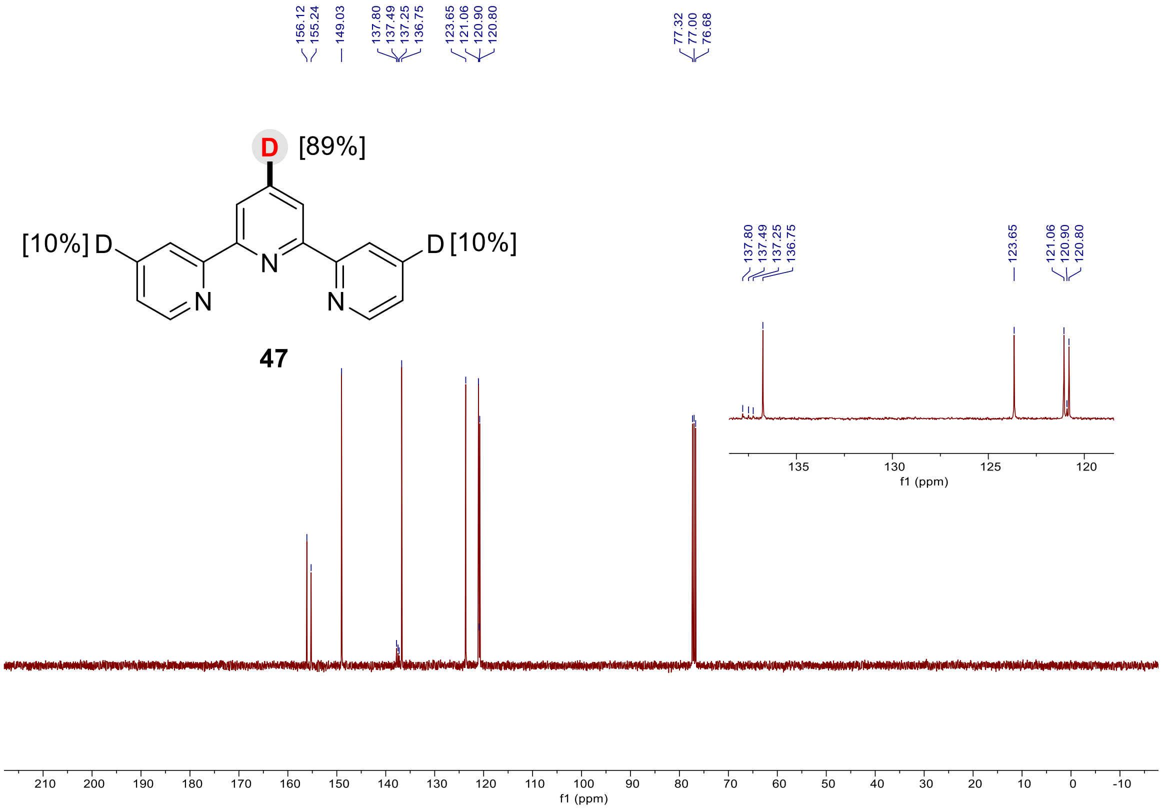

**$^{1}$H NMR** and **$^{13}$C NMR** of **48**

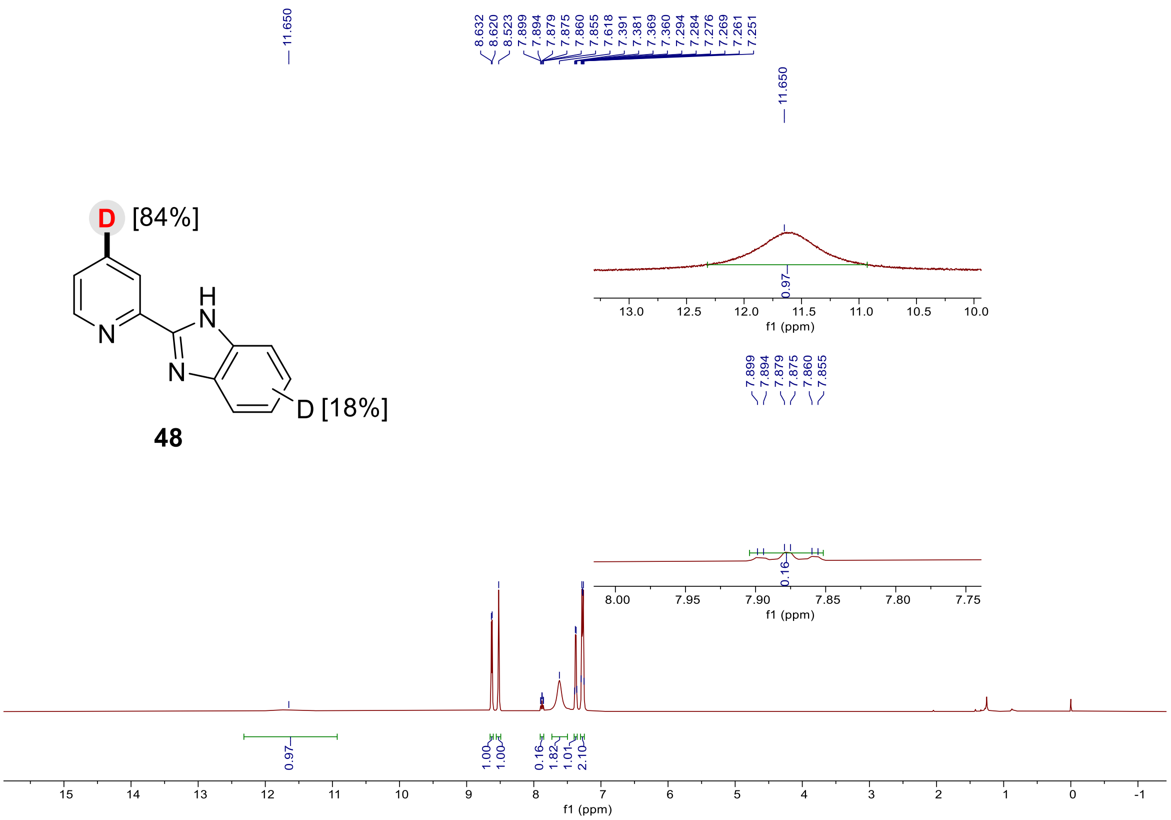


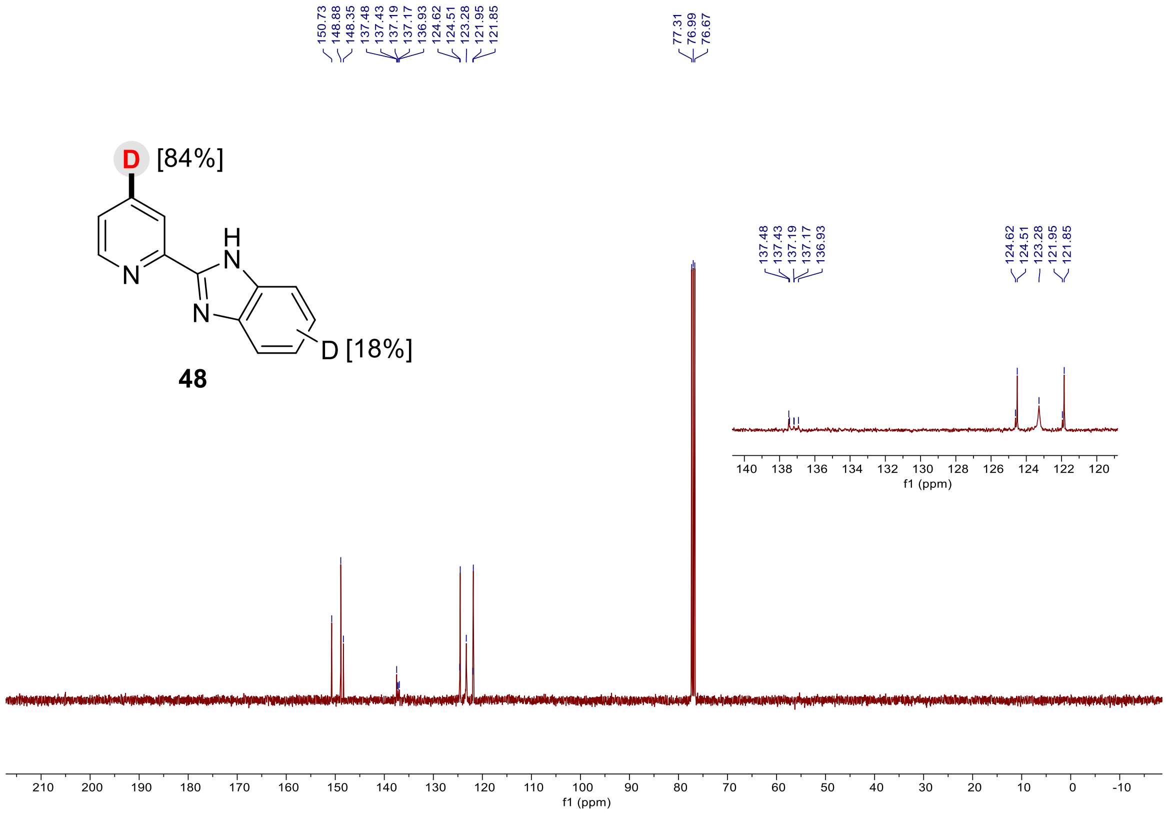

**$^{1}$H NMR** and **$^{13}$C NMR** of **49**

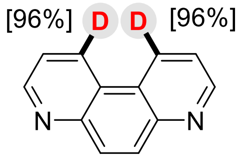


**49**

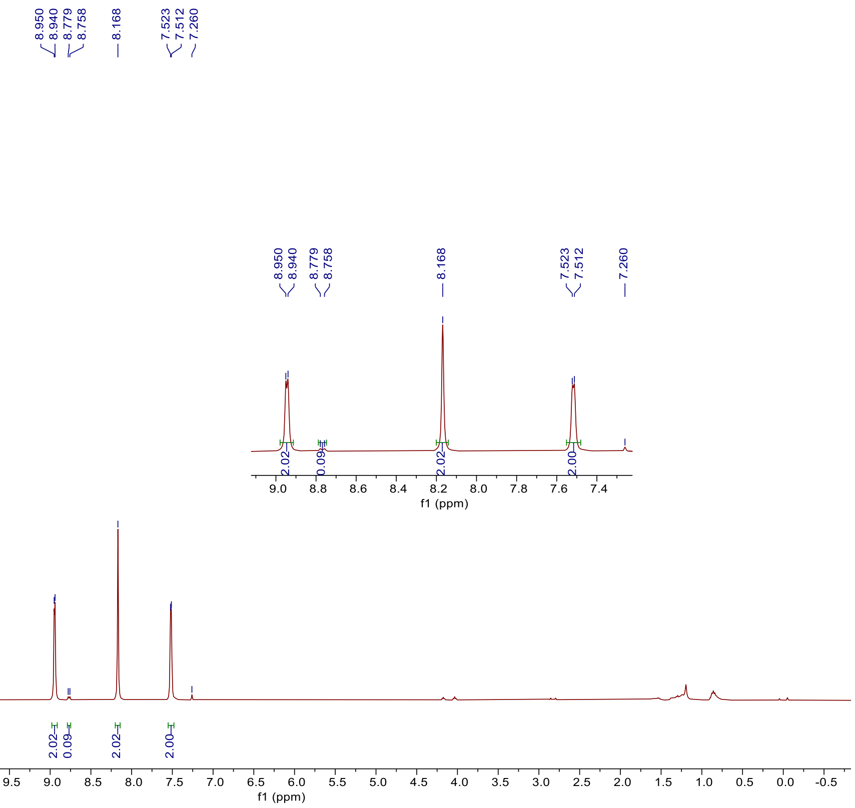


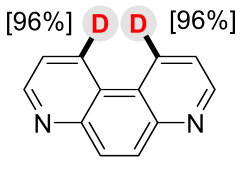


**49**

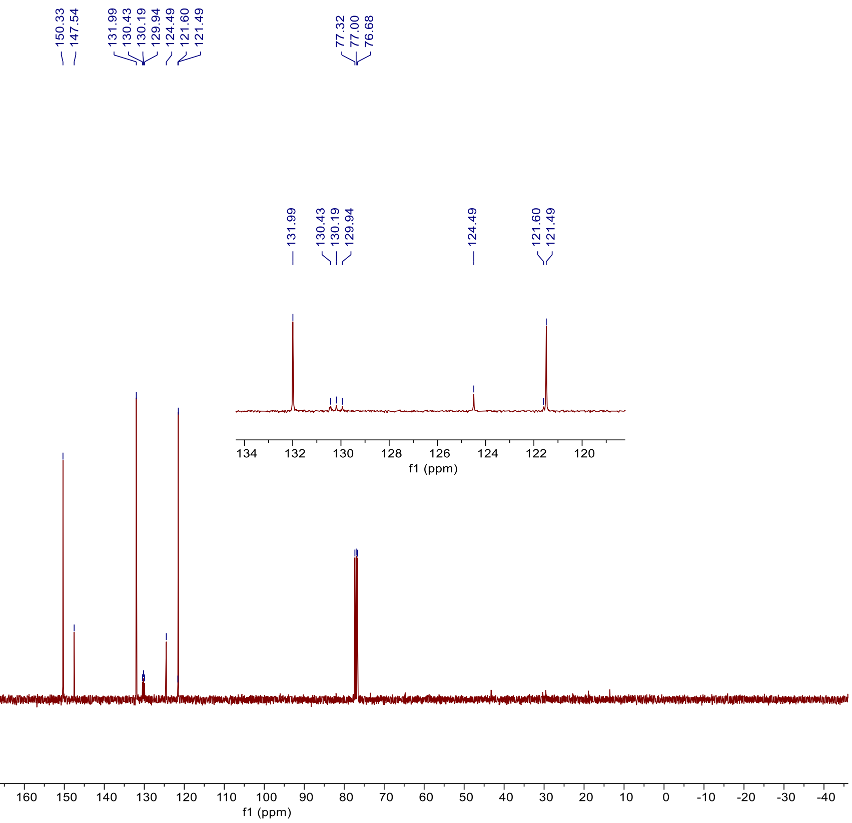

**$^{1}$H NMR** and **$^{13}$C NMR** of **50**

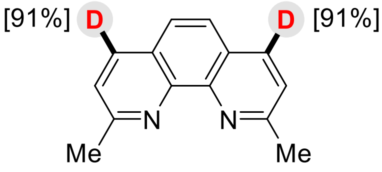


**50**

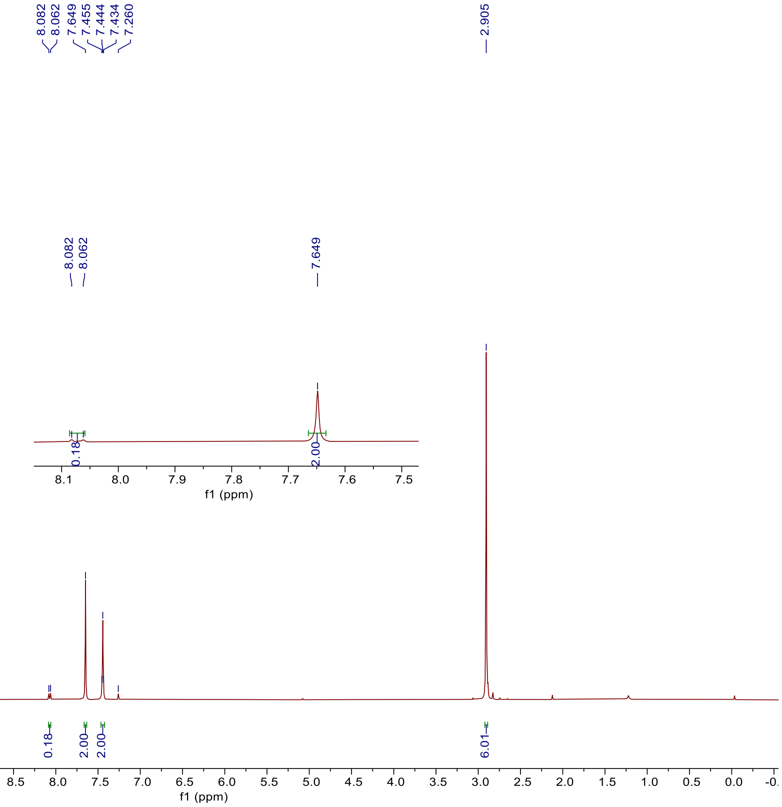


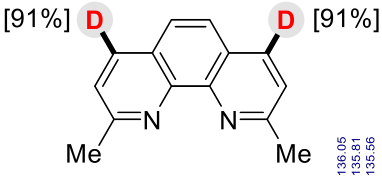


**50**

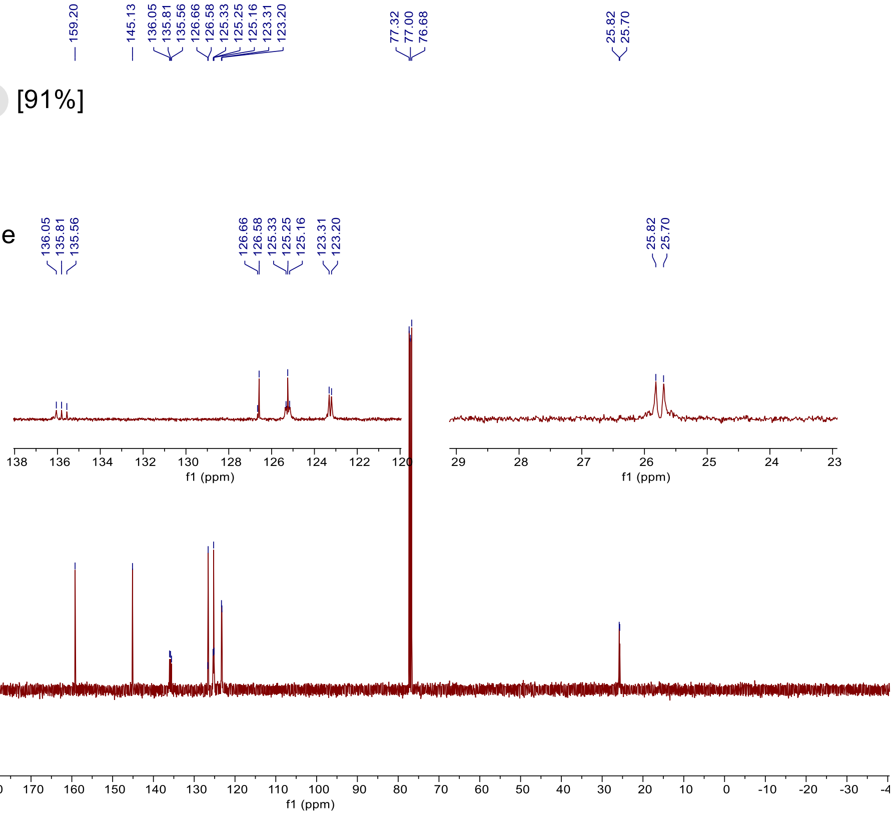

$^{1}$H NMR and $^{13}$C NMR of **51**

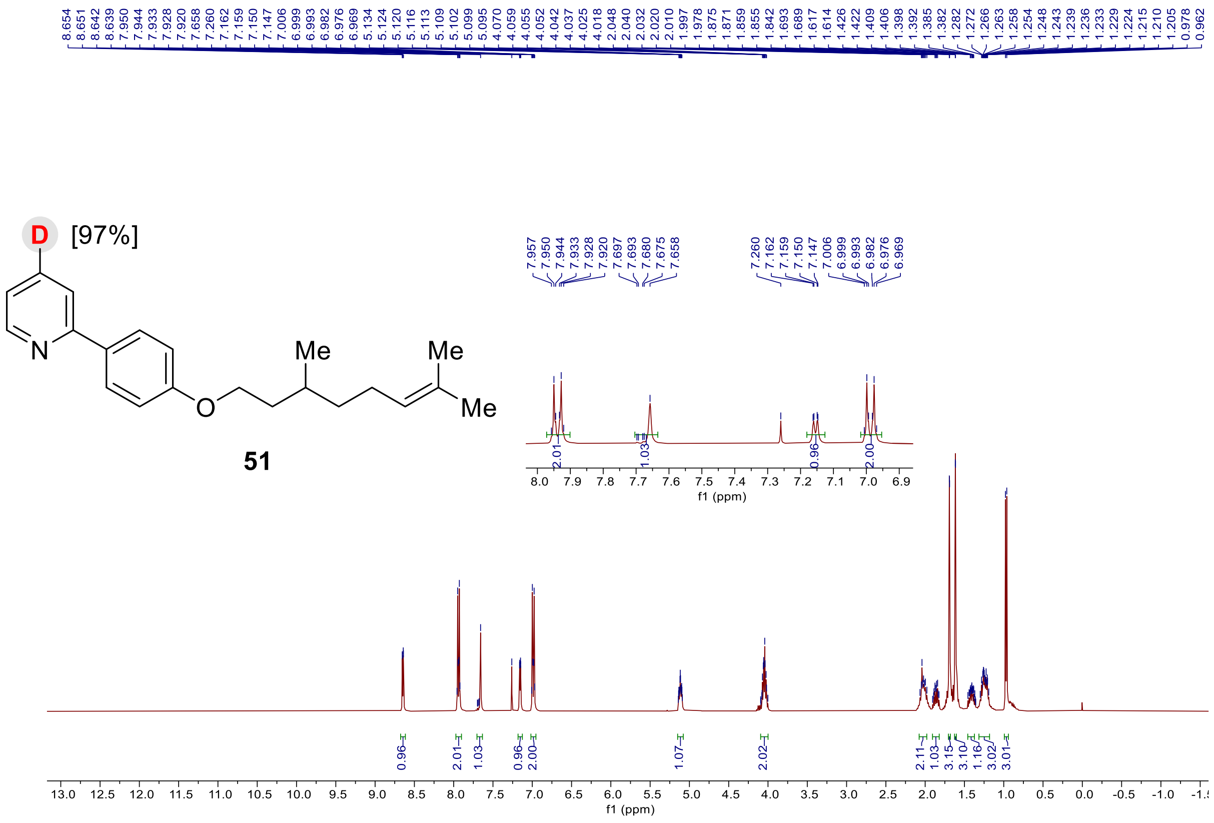

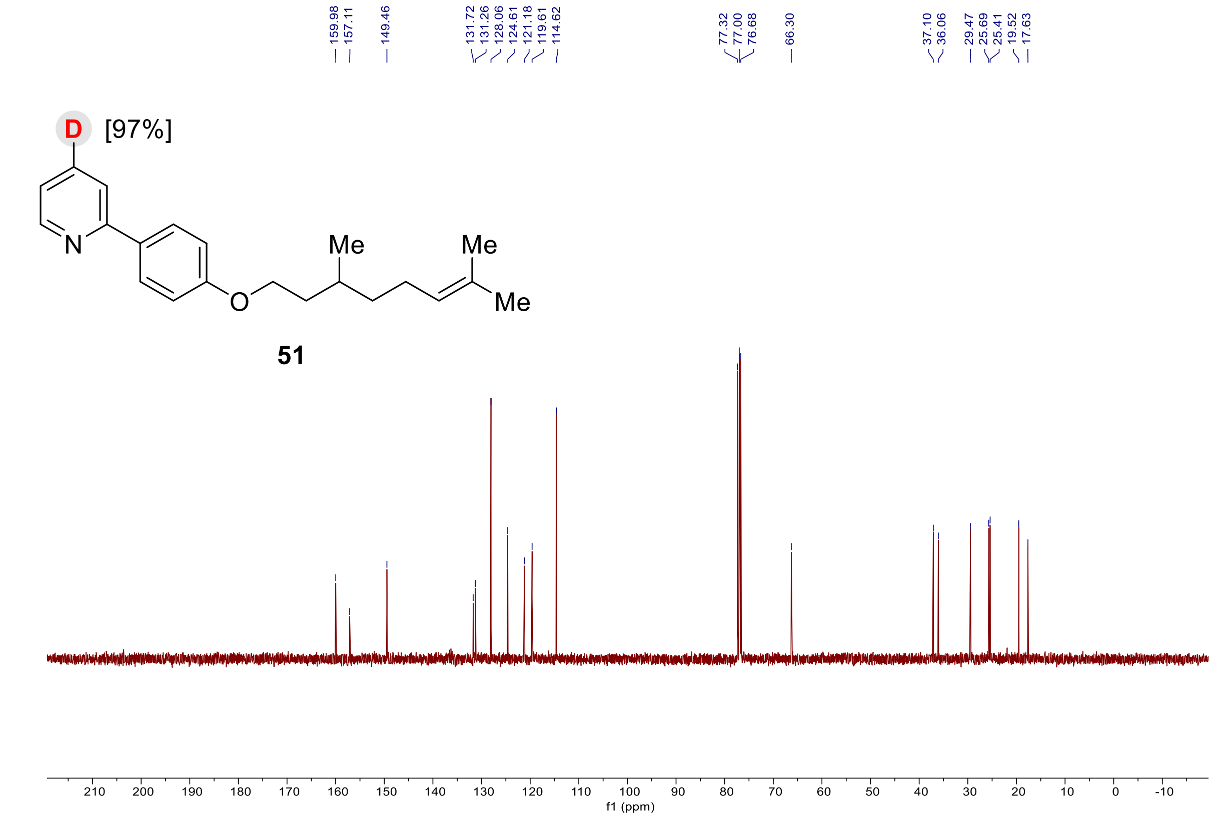

## $^{1}$H NMR and $^{13}$C NMR of **52**

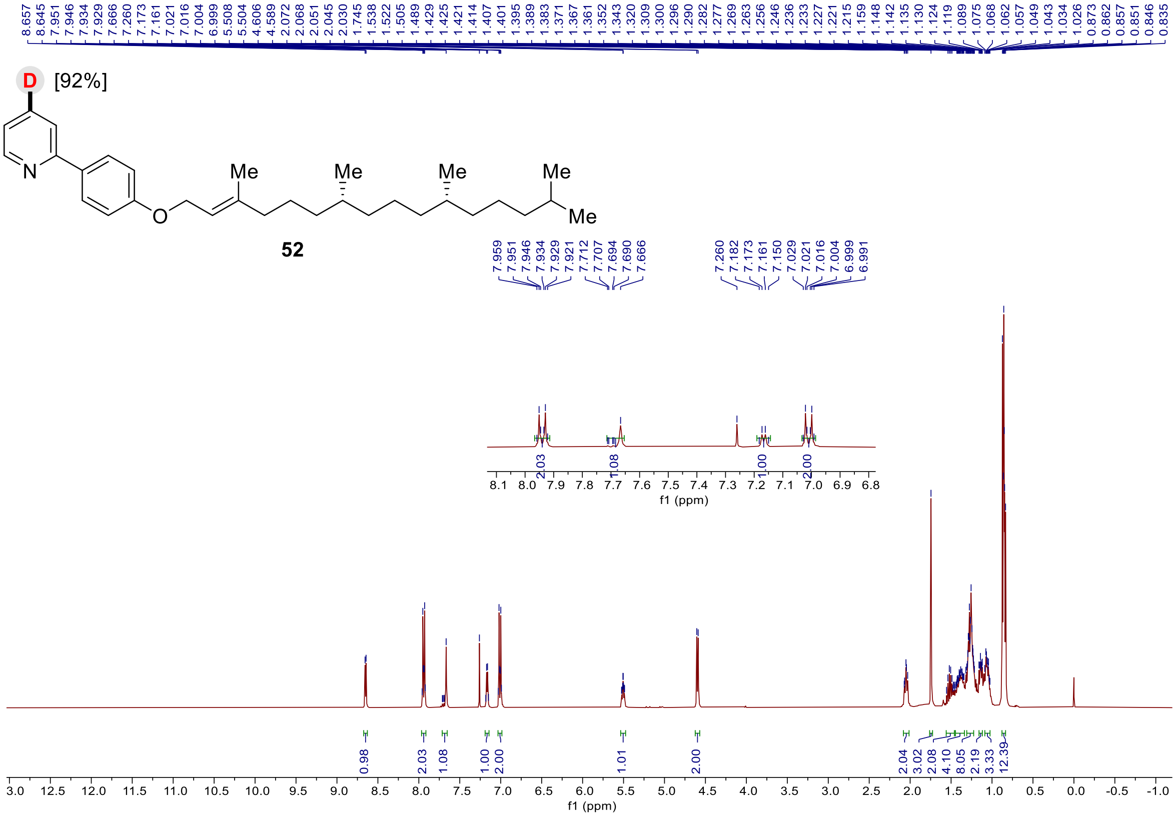


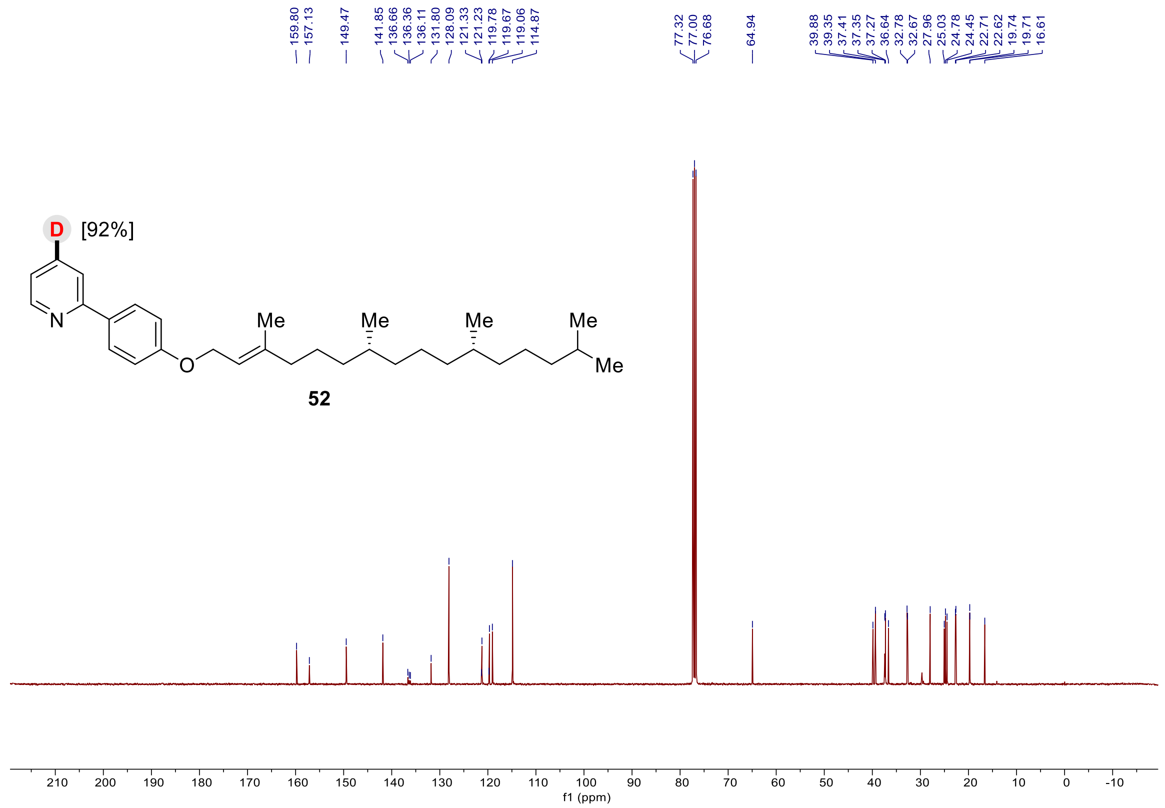

**$^1$H NMR and $^{13}$C NMR of 53**

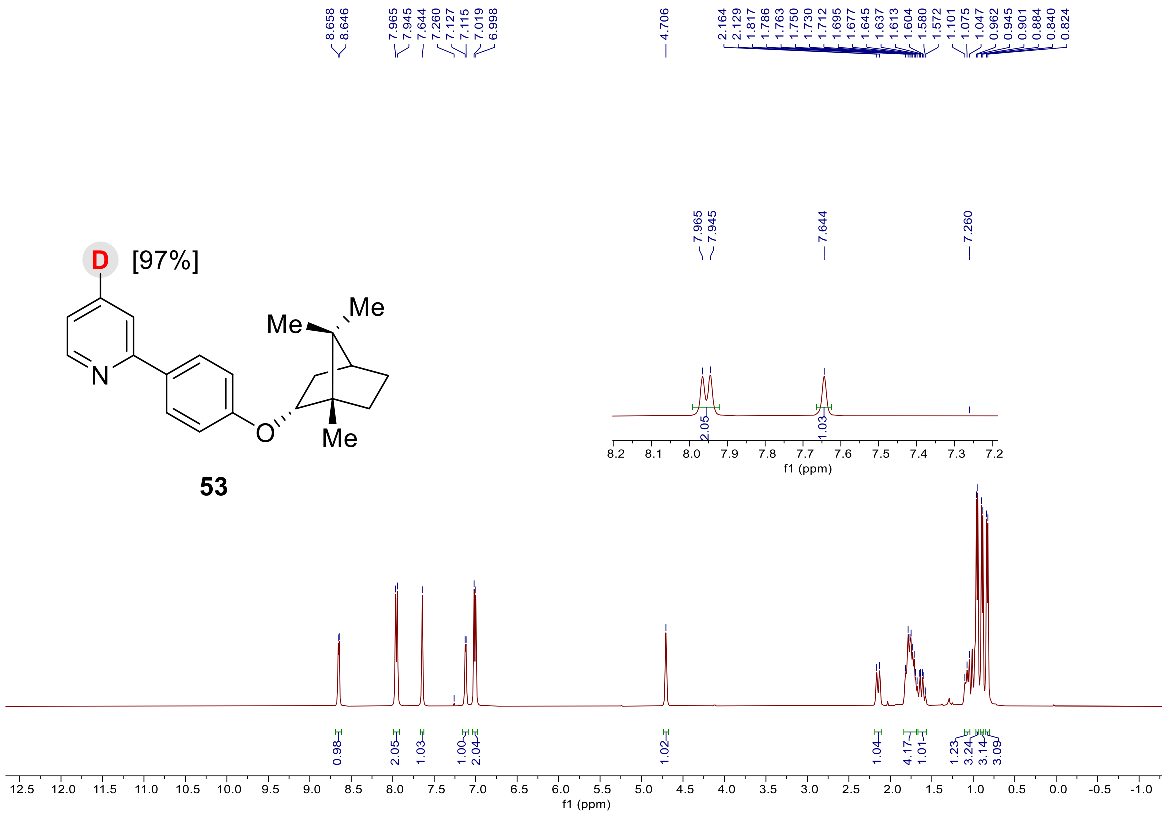

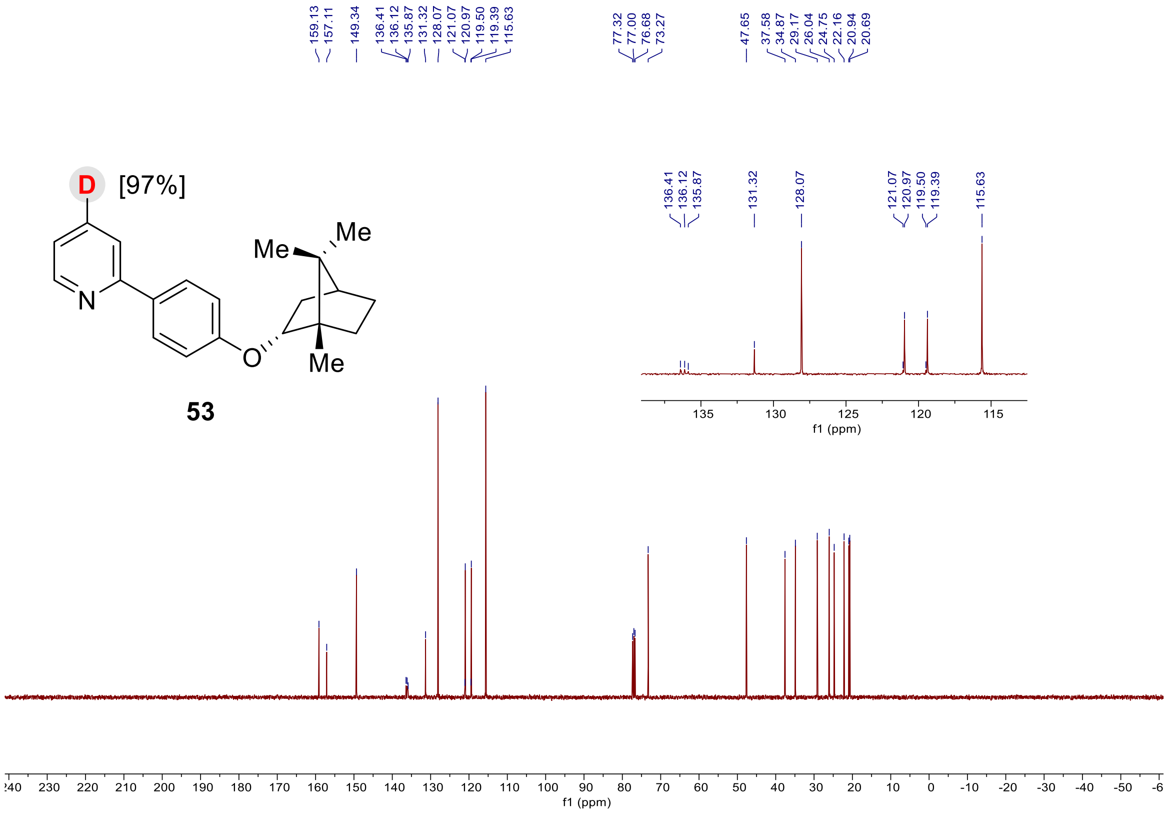

$^{1}$H NMR and $^{13}$C NMR of **54**

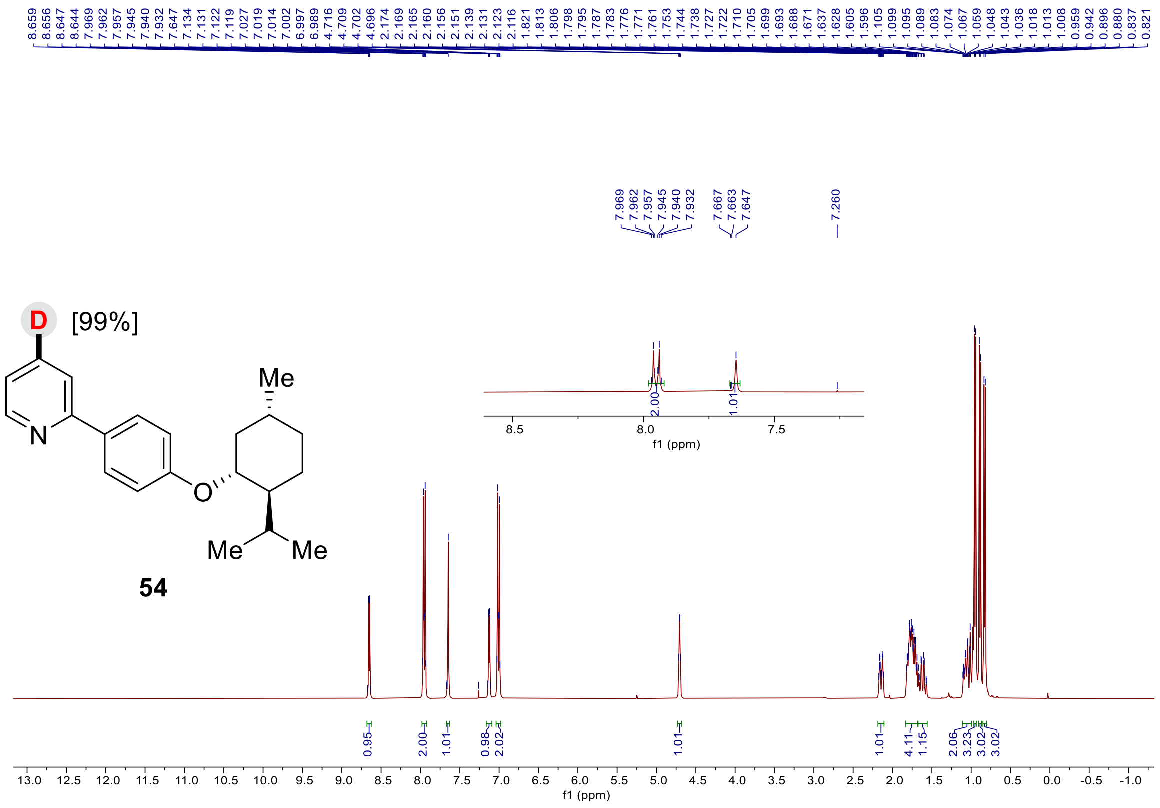


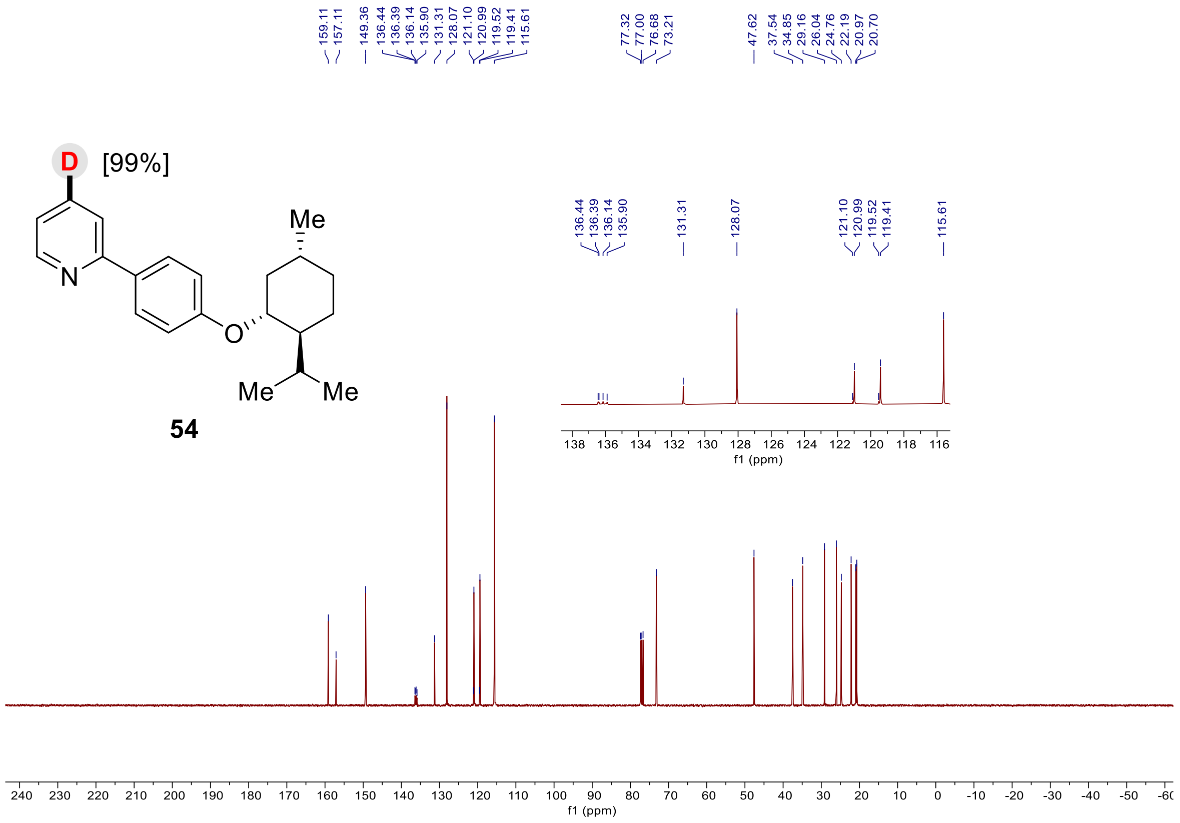

## $^{1}H$ NMR and $^{13}C$ NMR of 55

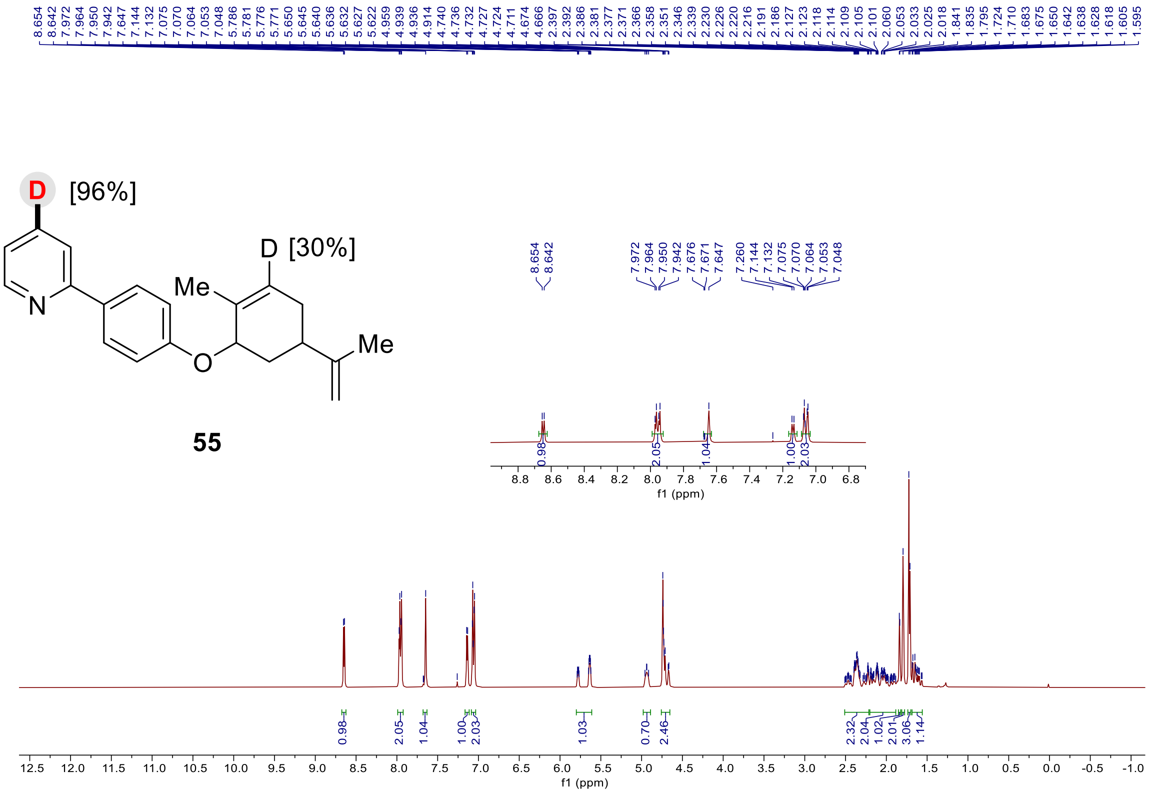


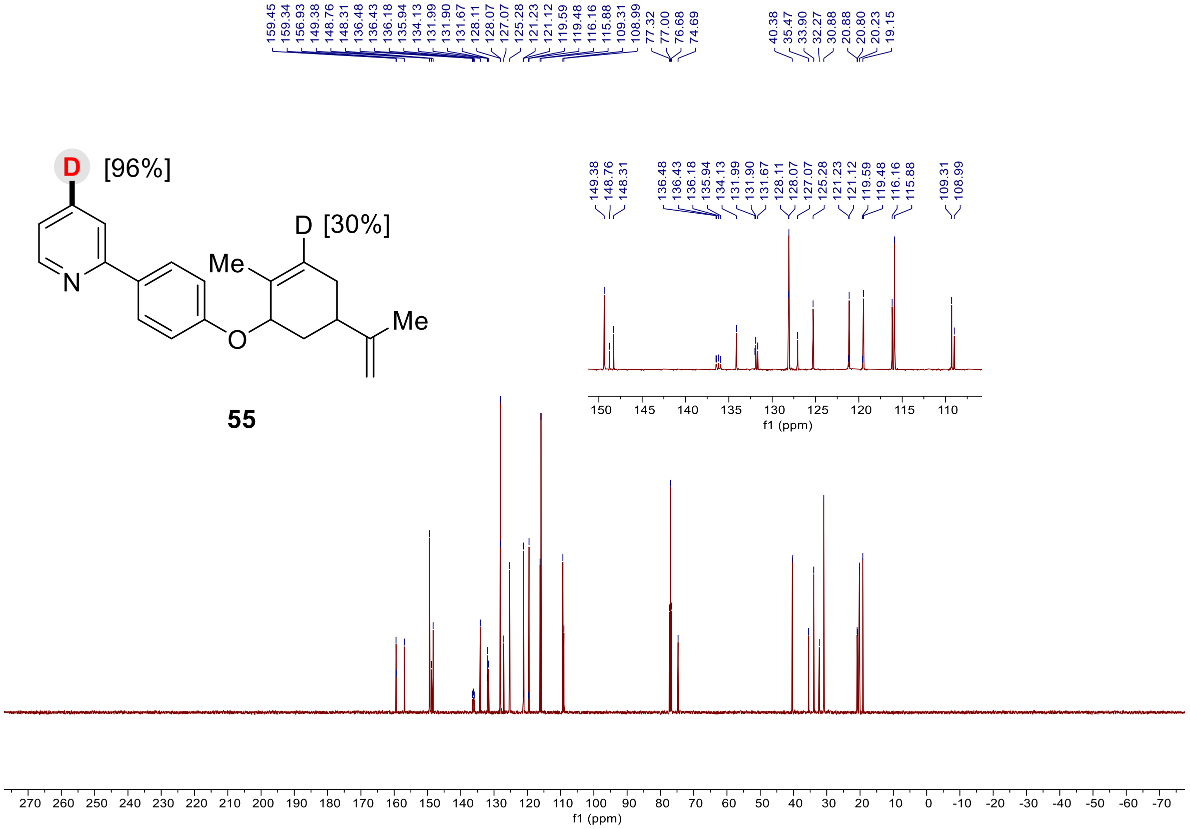

## $^{1}$H NMR and $^{13}$C NMR of 56

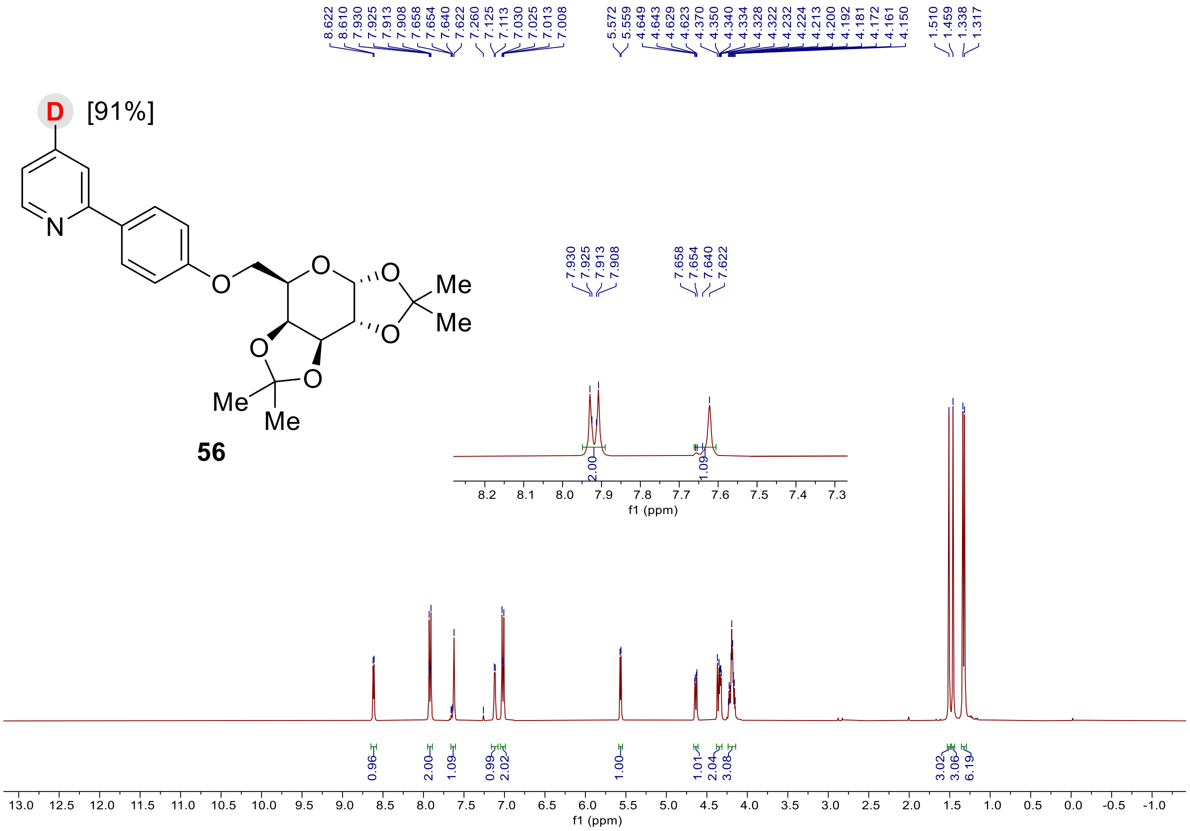


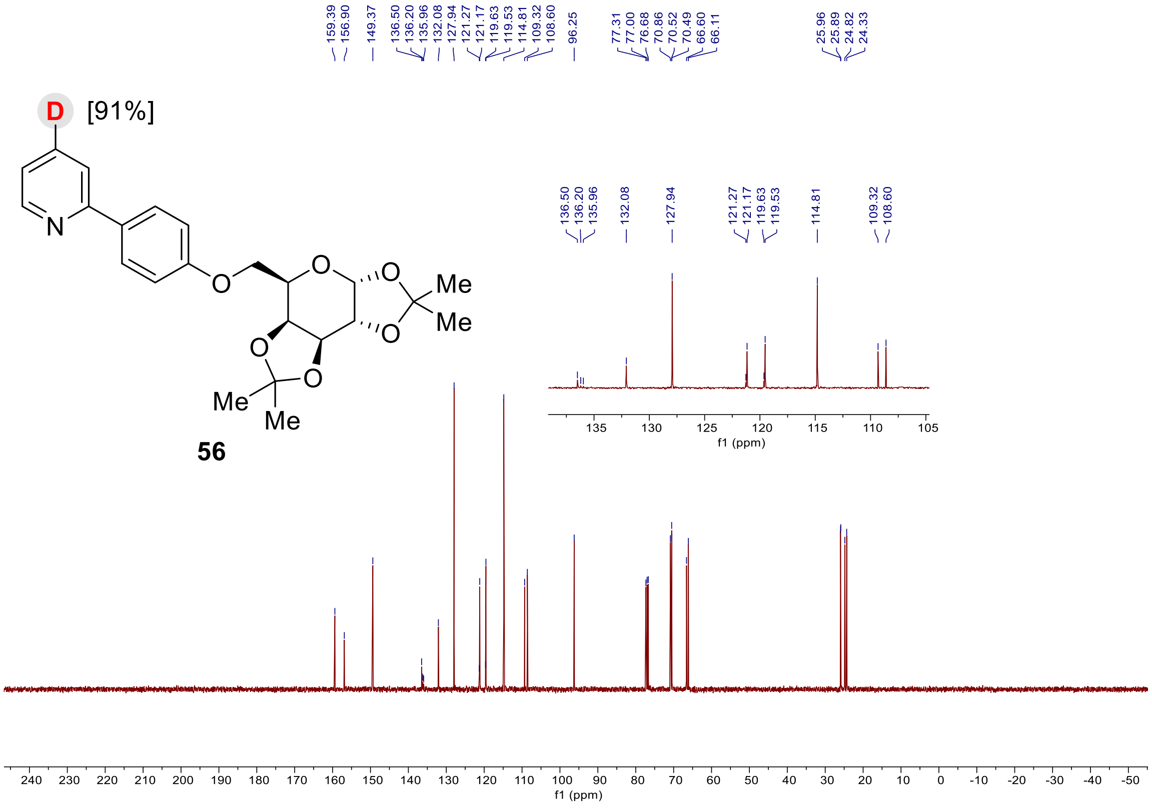

**$^{1}$H NMR** and **$^{13}$C NMR** of **57**

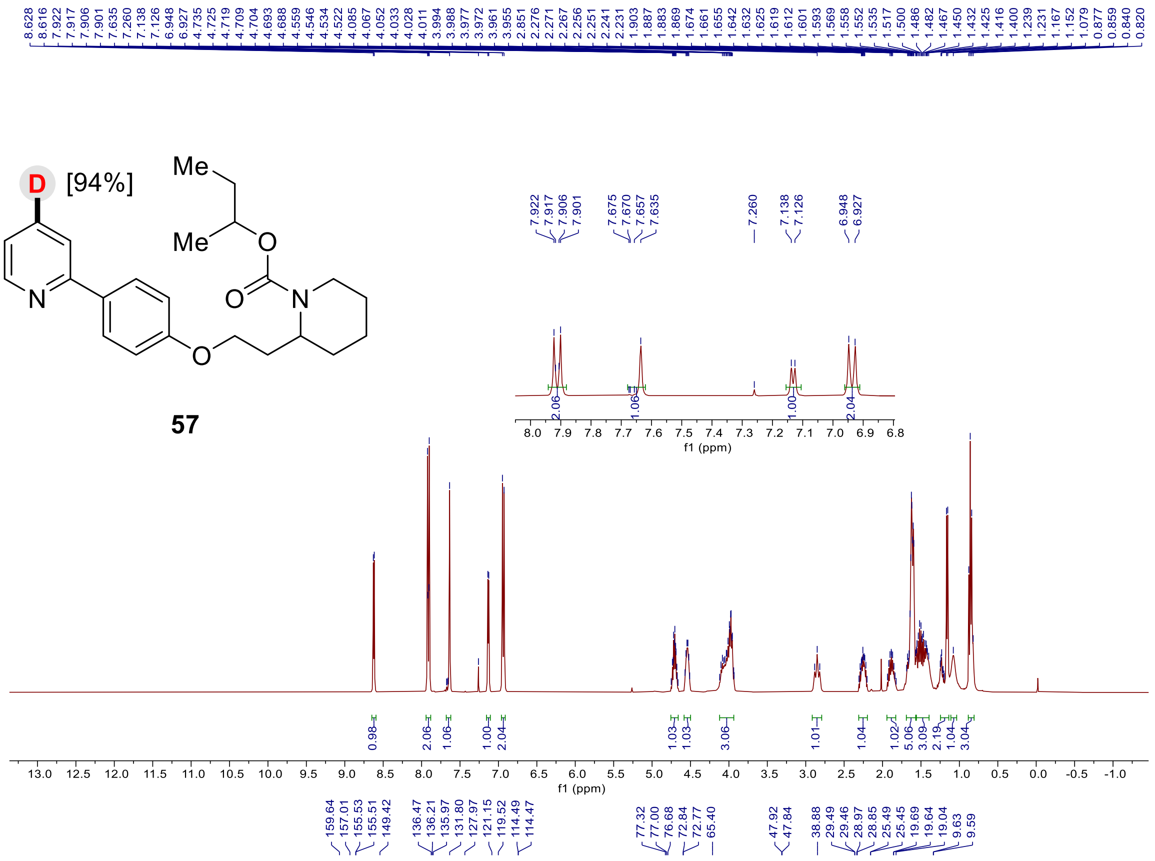


**57**

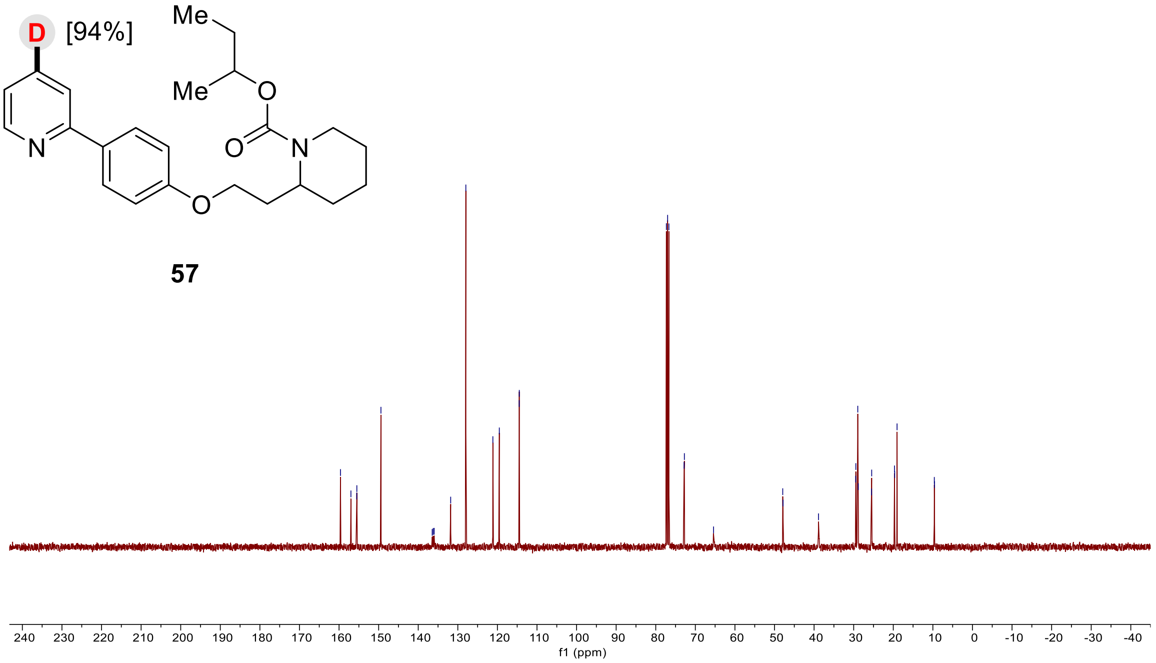


**57**

## $^{1}$H NMR and $^{13}$C NMR of 58

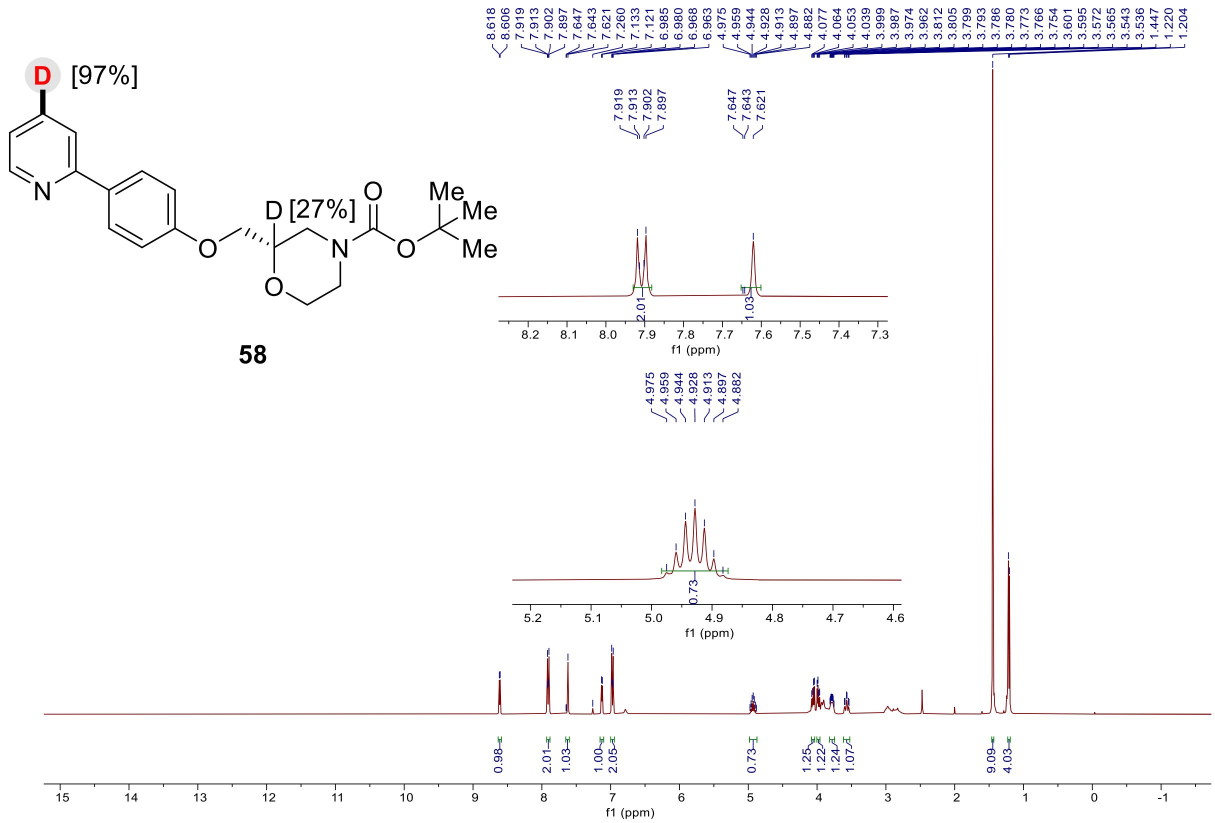


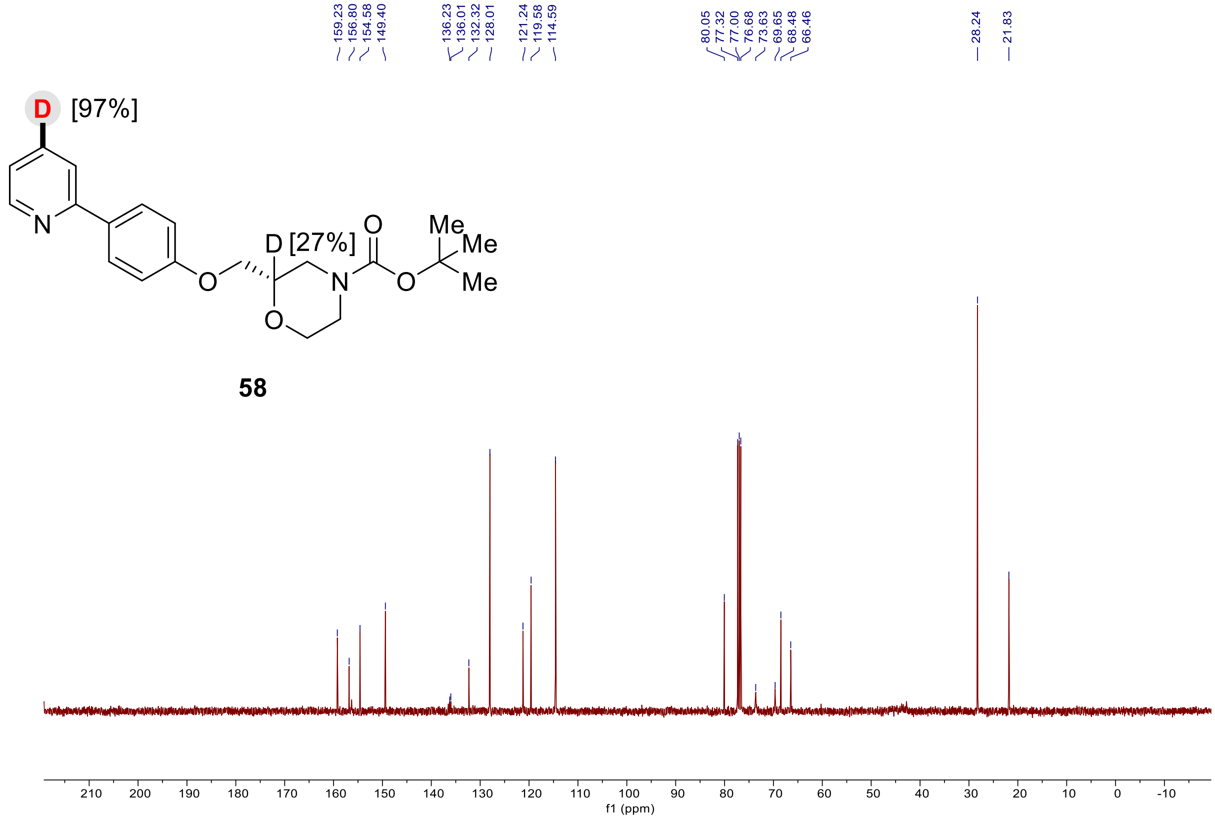

## $^{1}$H NMR and $^{13}$C NMR of 59

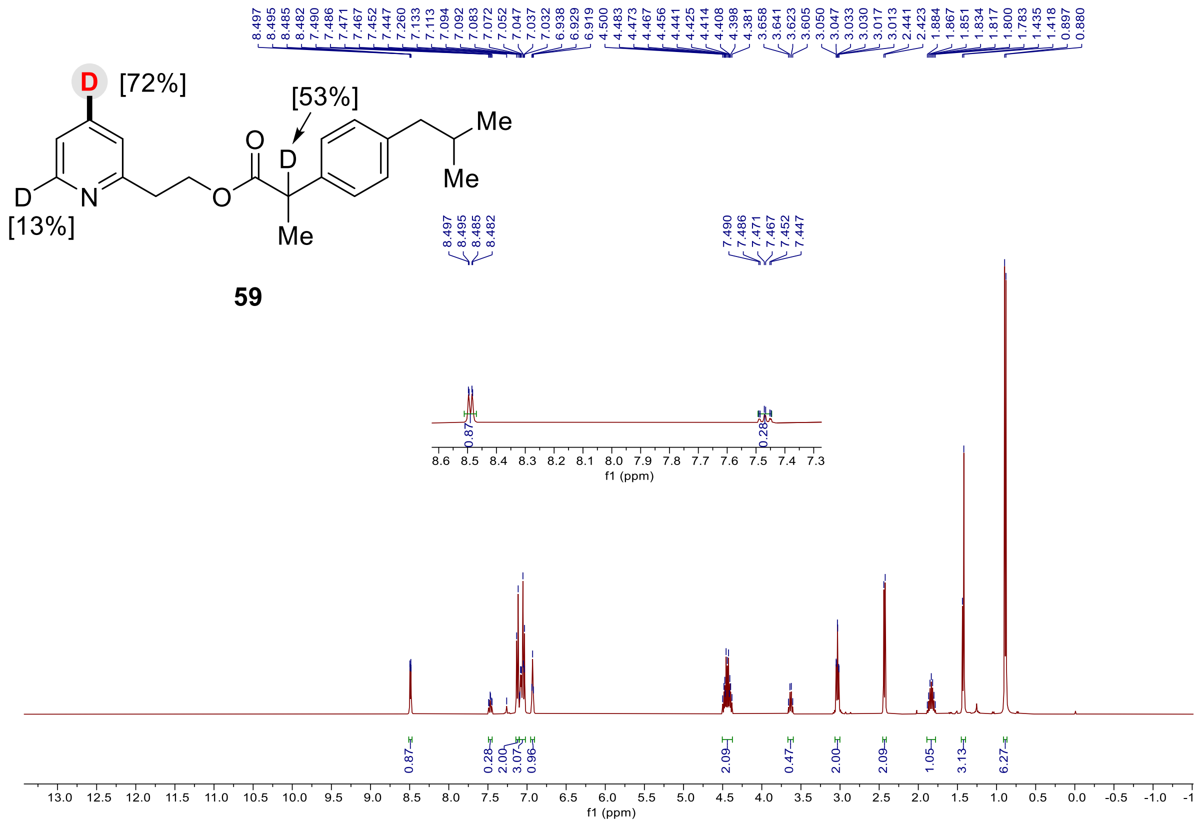


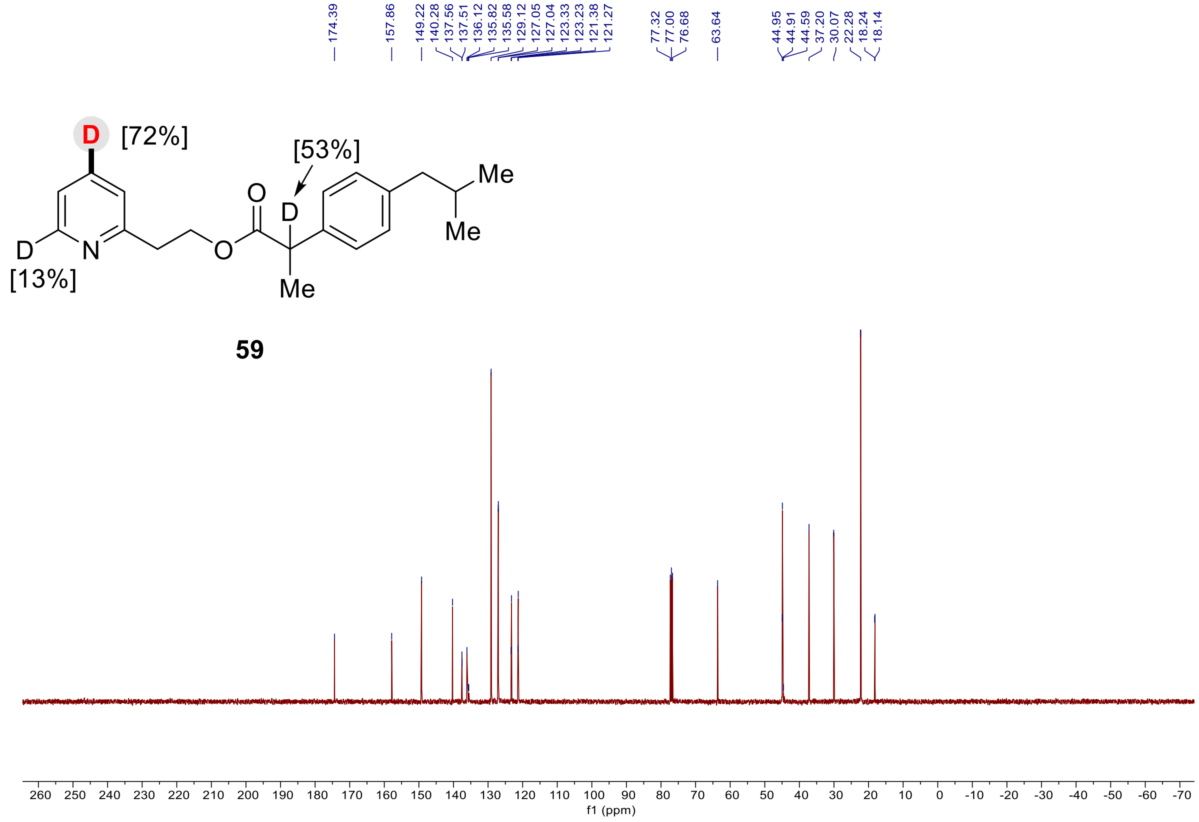

**$^{1}$H NMR** and **$^{13}$C NMR** of **60**

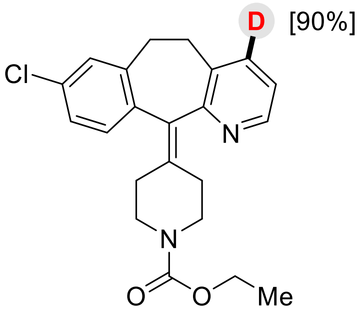


**60**

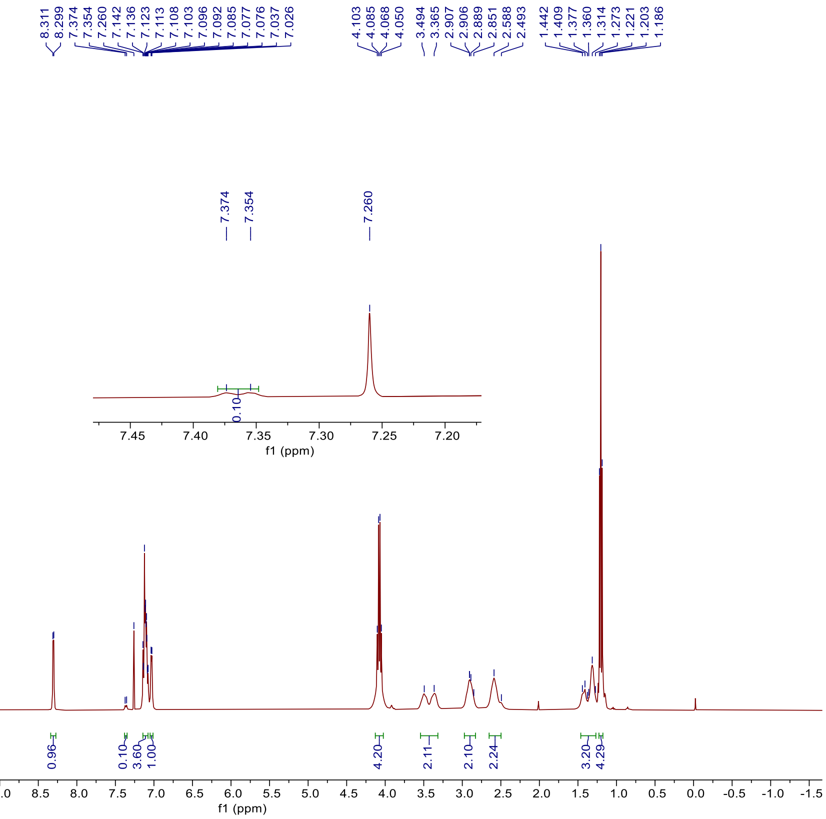


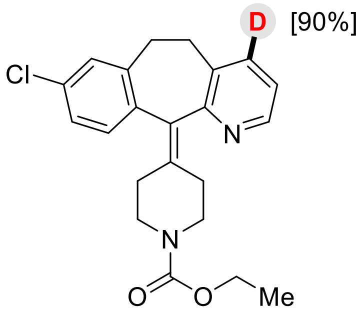


**60**

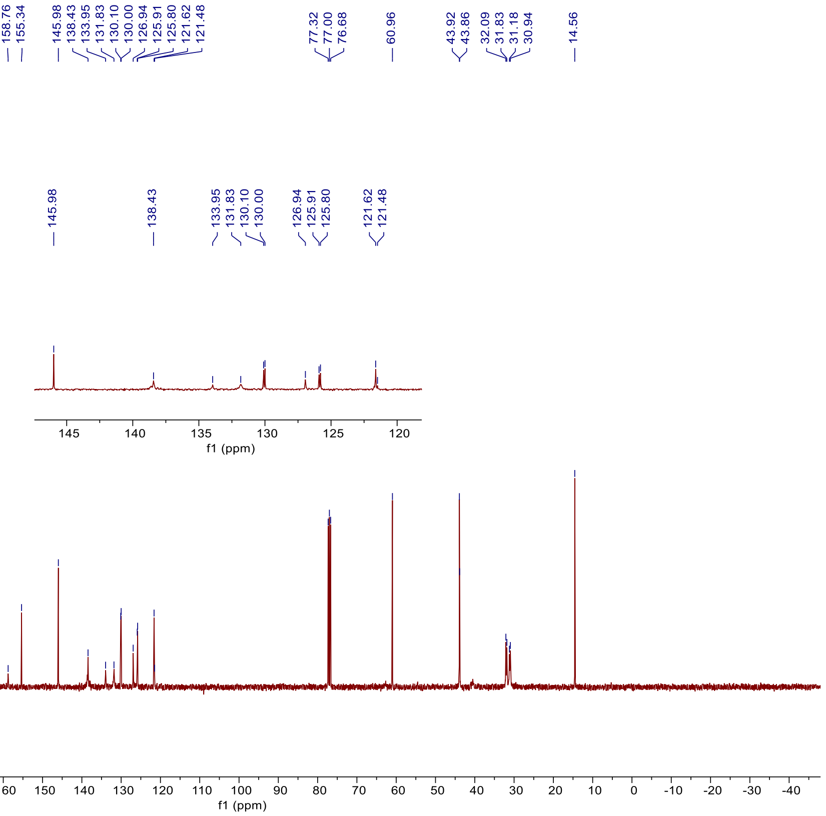

**$^{1}$H NMR** and **$^{13}$C NMR** of **61**

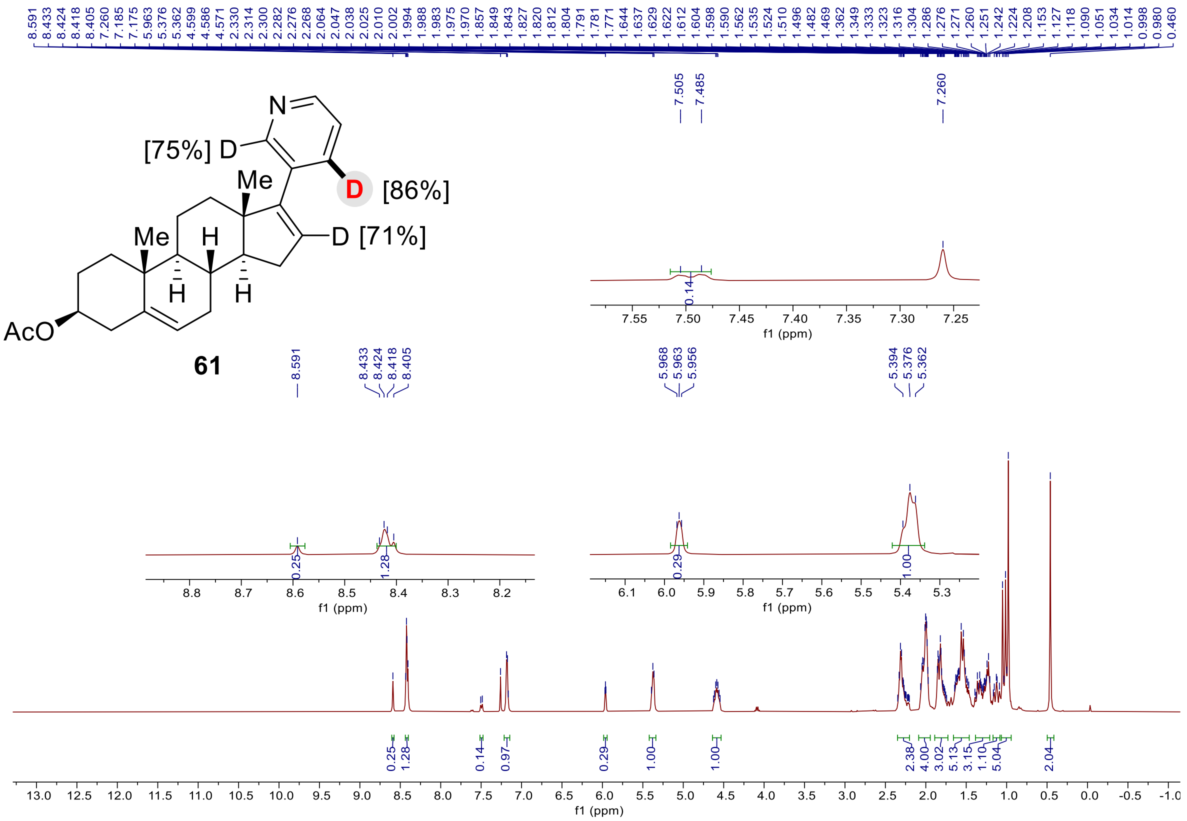


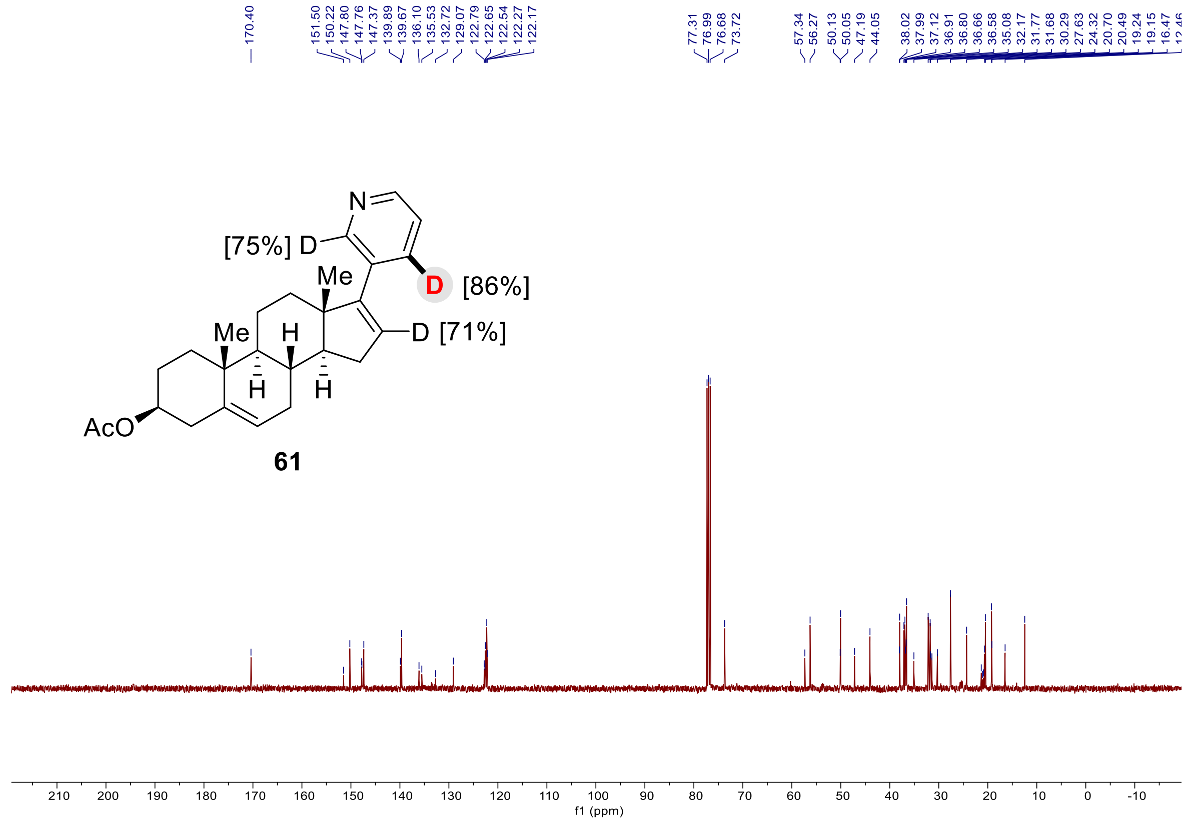

$^{1}H$ NMR and $^{13}C$ NMR of **62**

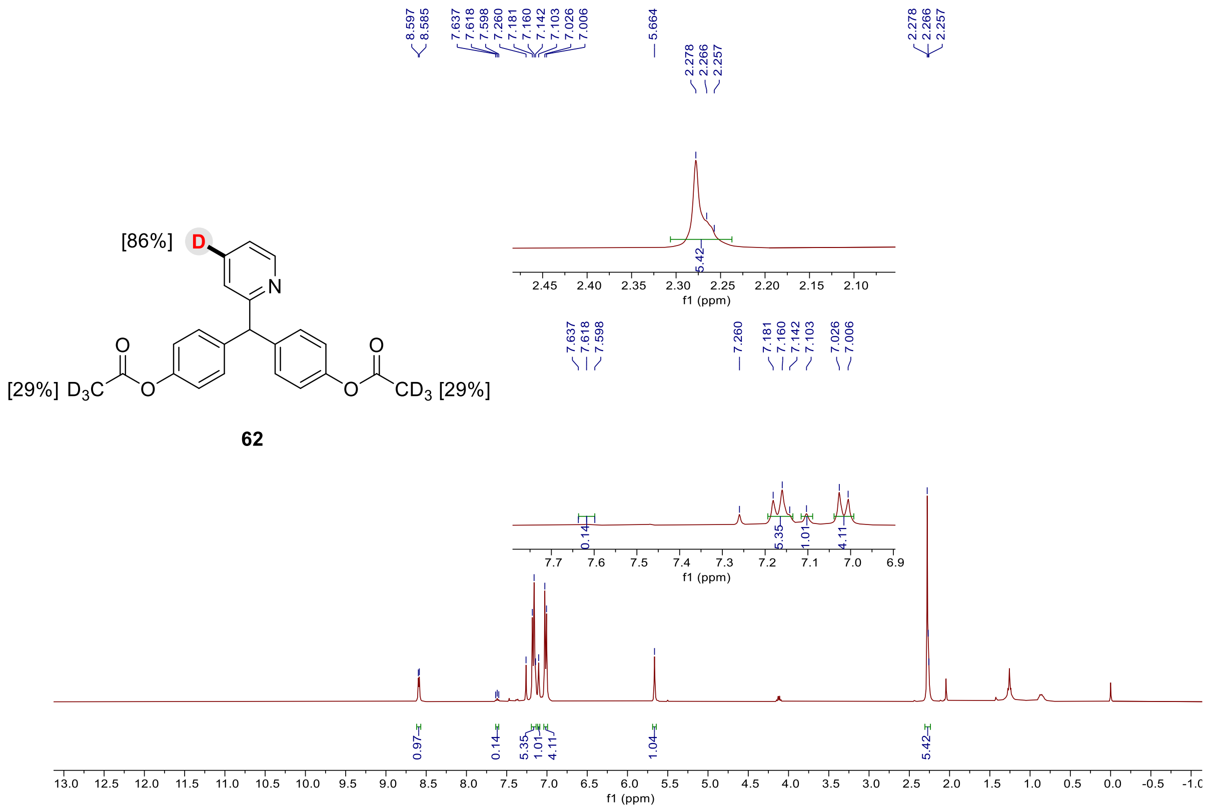


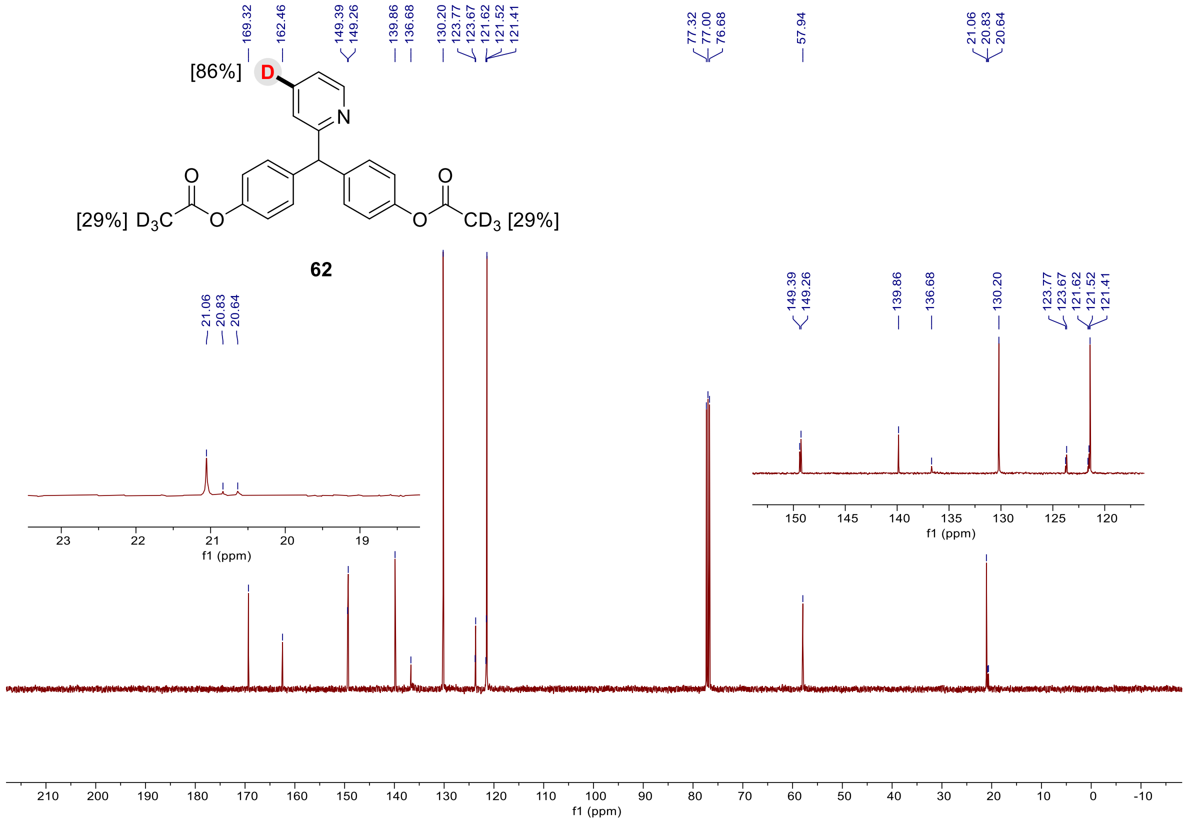

**$^{1}$H NMR** and **$^{13}$C NMR** of **63**

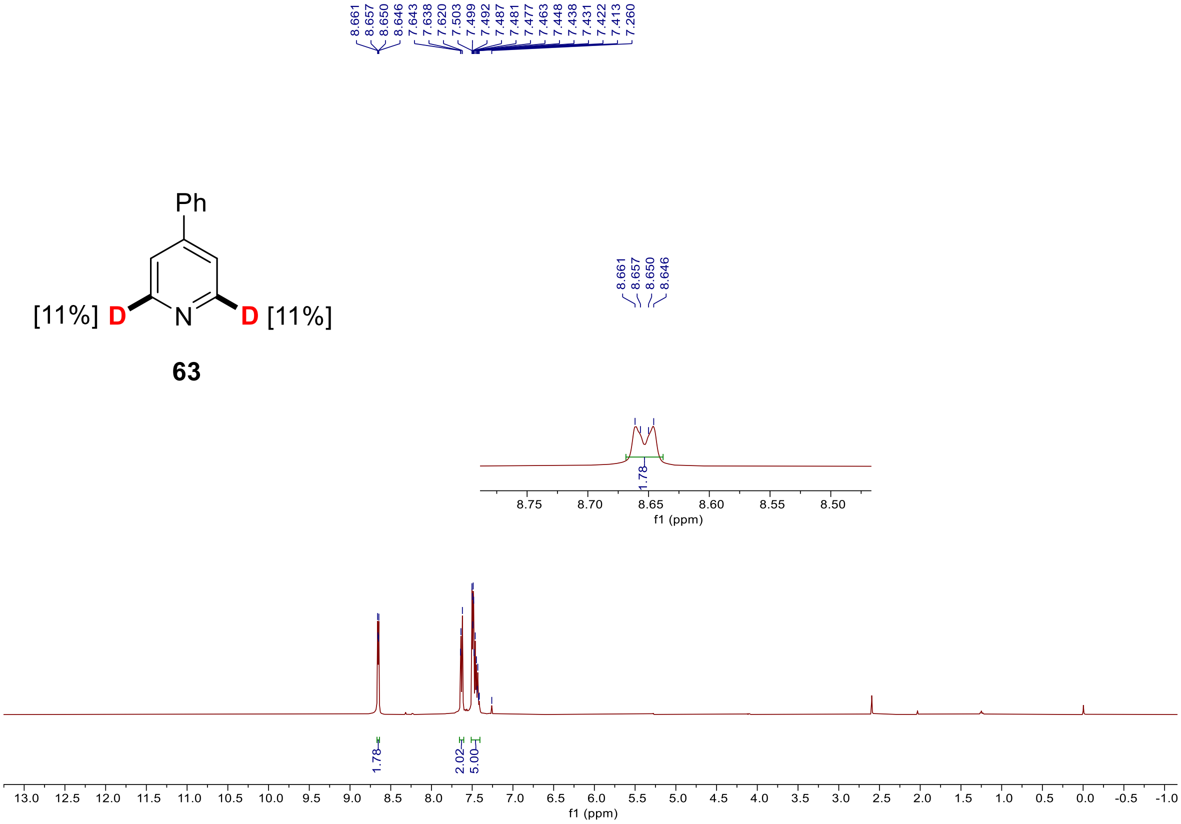


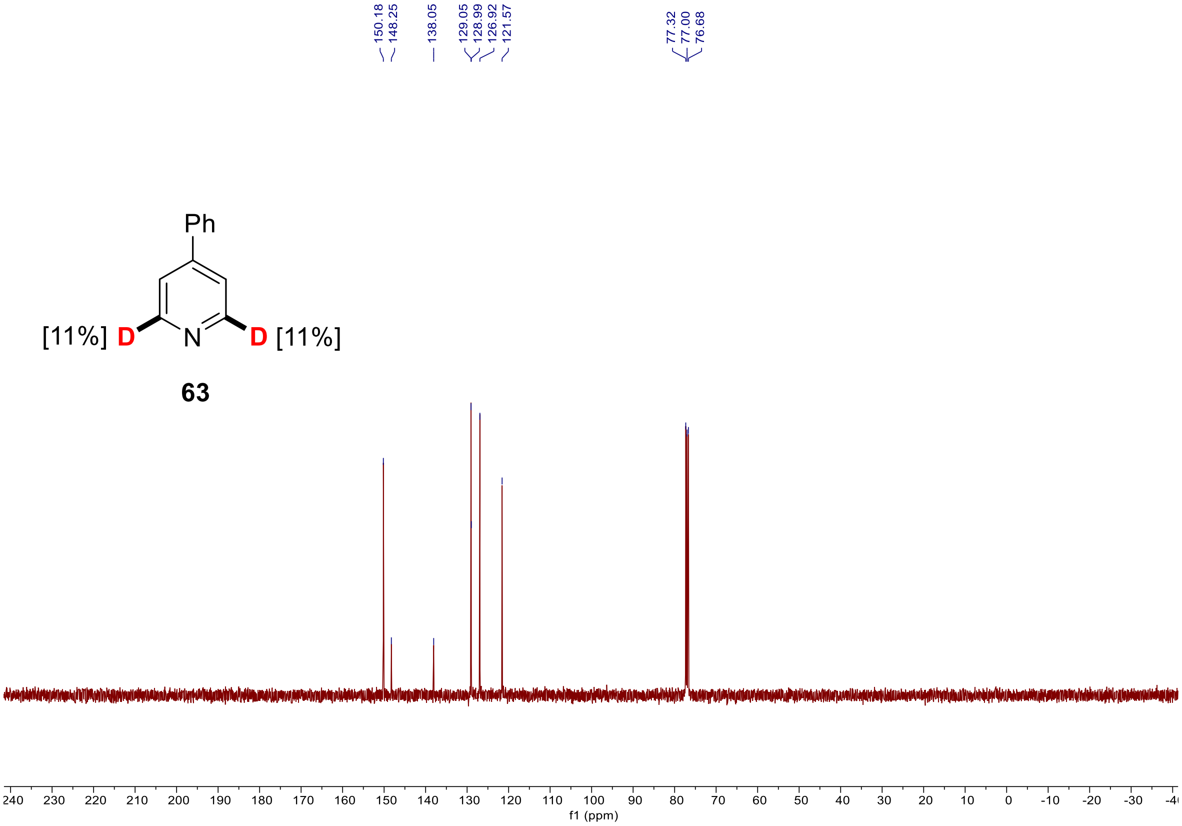

$^{1}$**H NMR** and $^{13}$**C NMR** of **64**

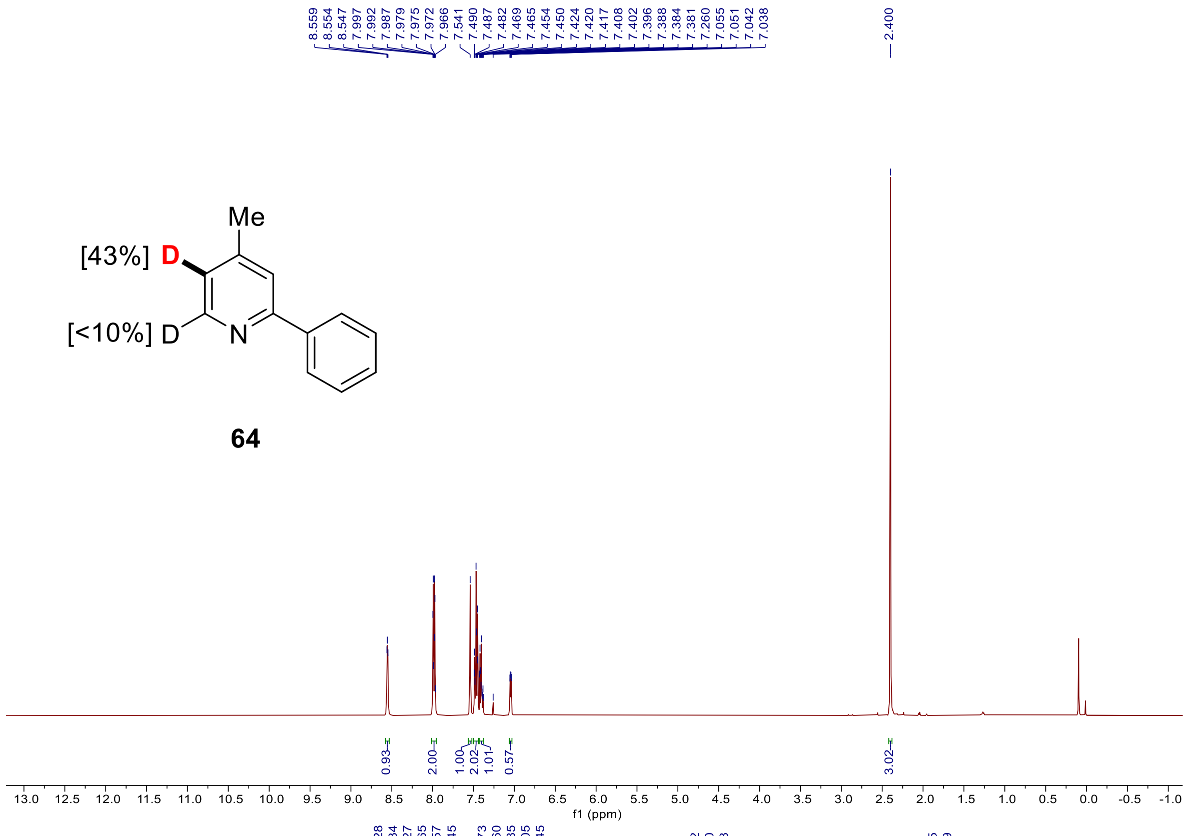

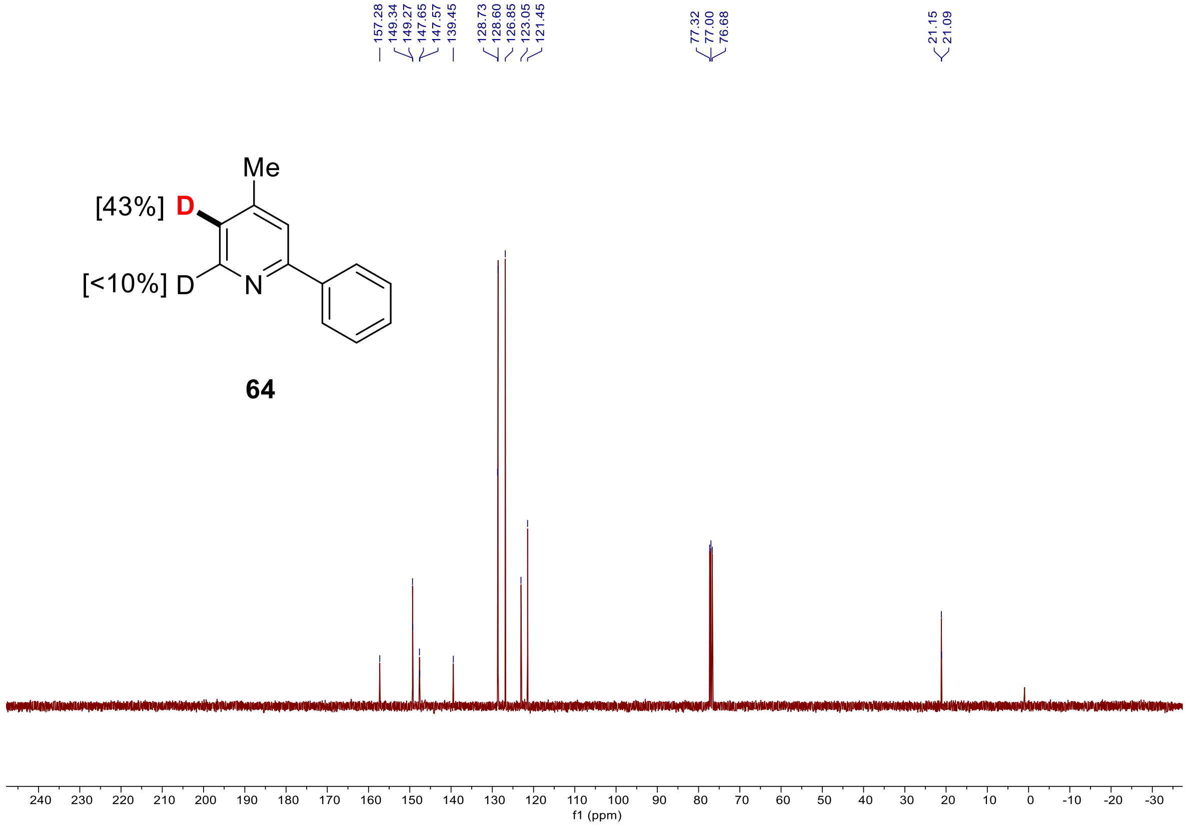

## $^{1}$H NMR and $^{13}$C NMR of **65**

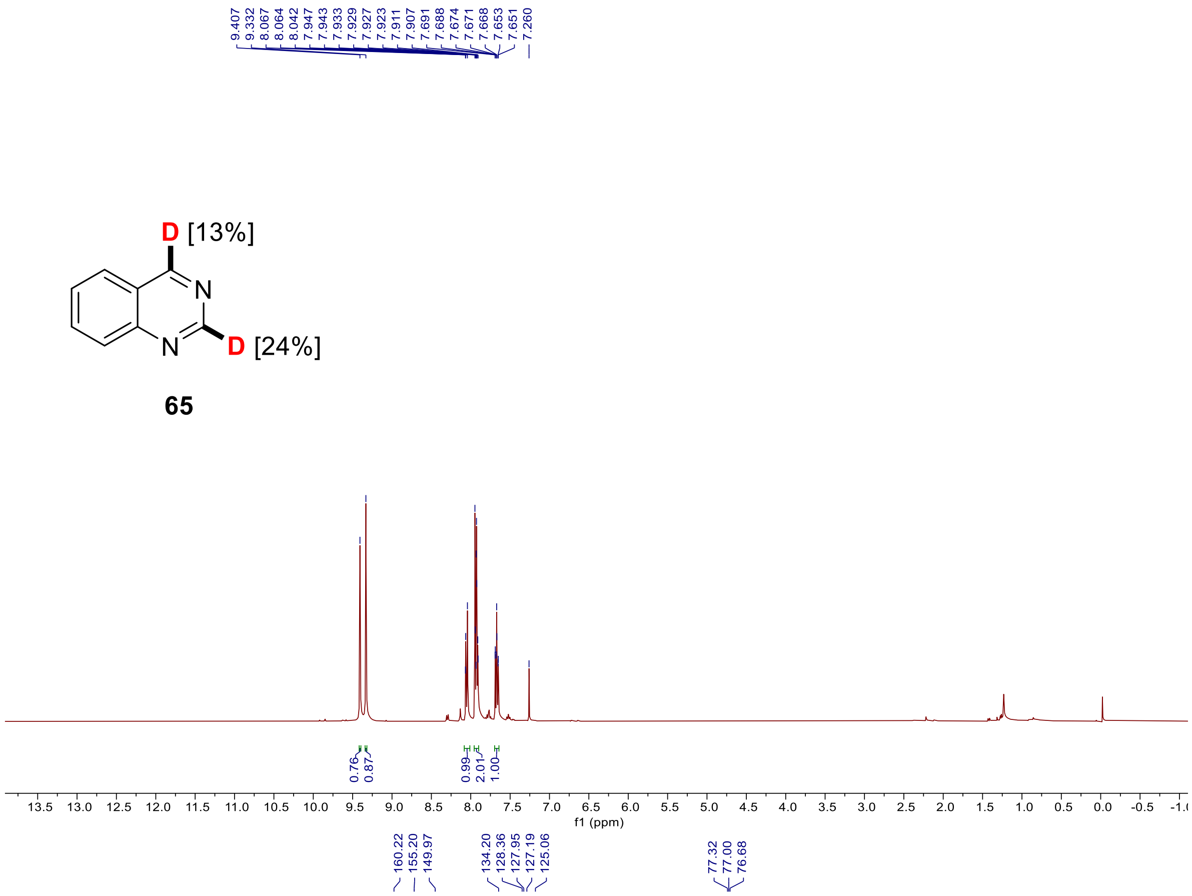


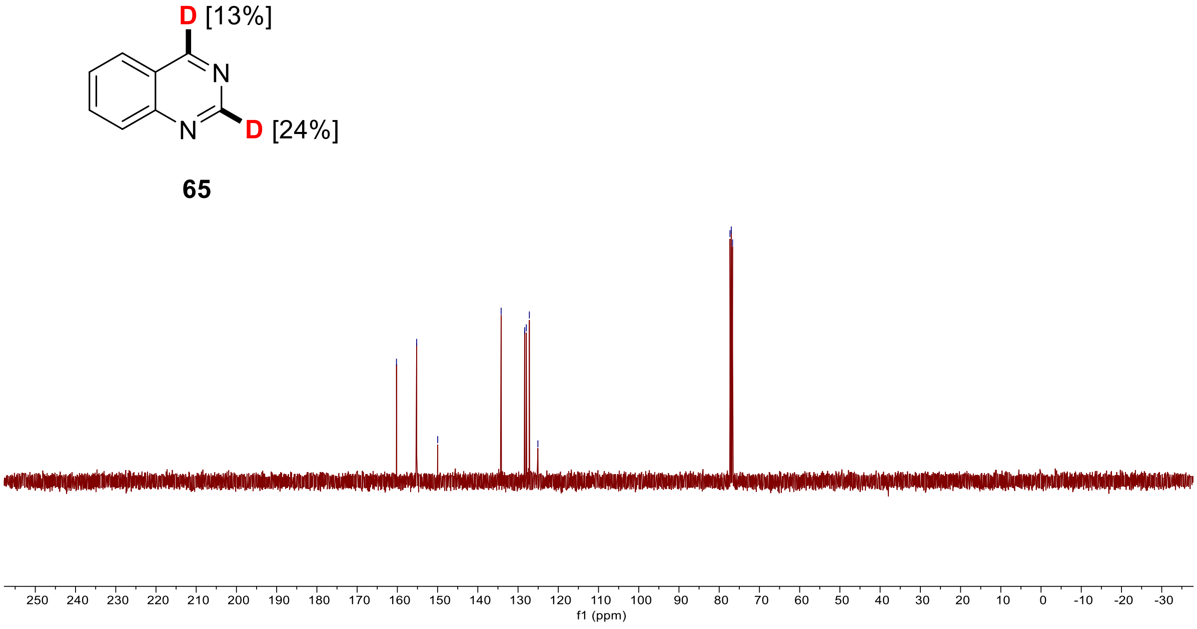

# $^{1}$H NMR and $^{13}$C NMR of 66

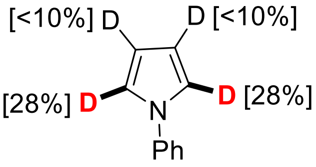


**66**

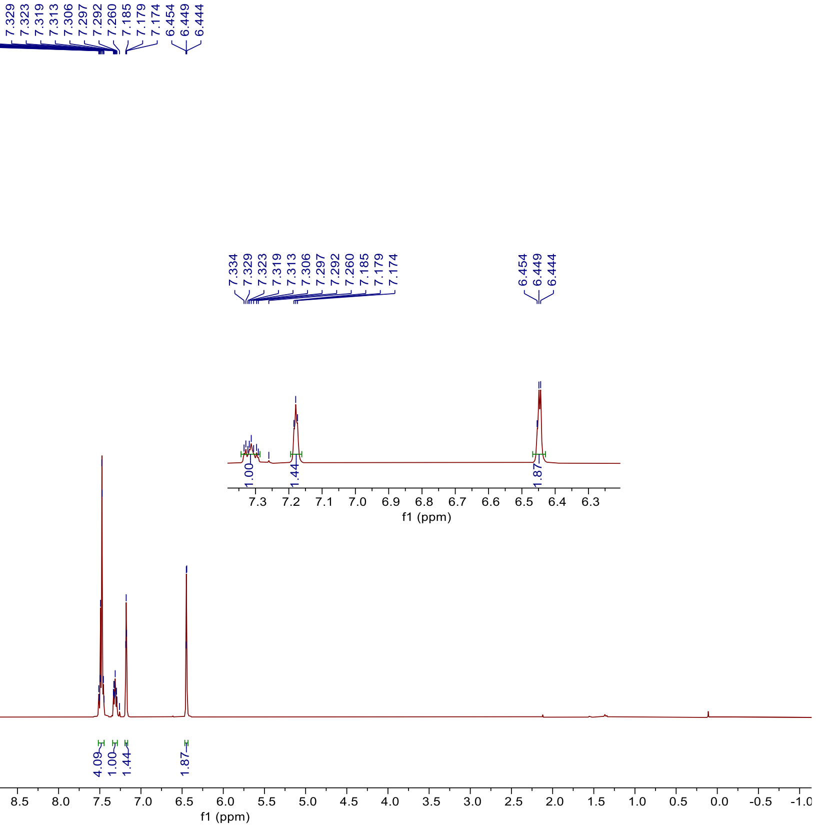


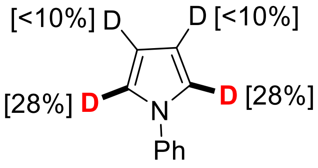


**66**

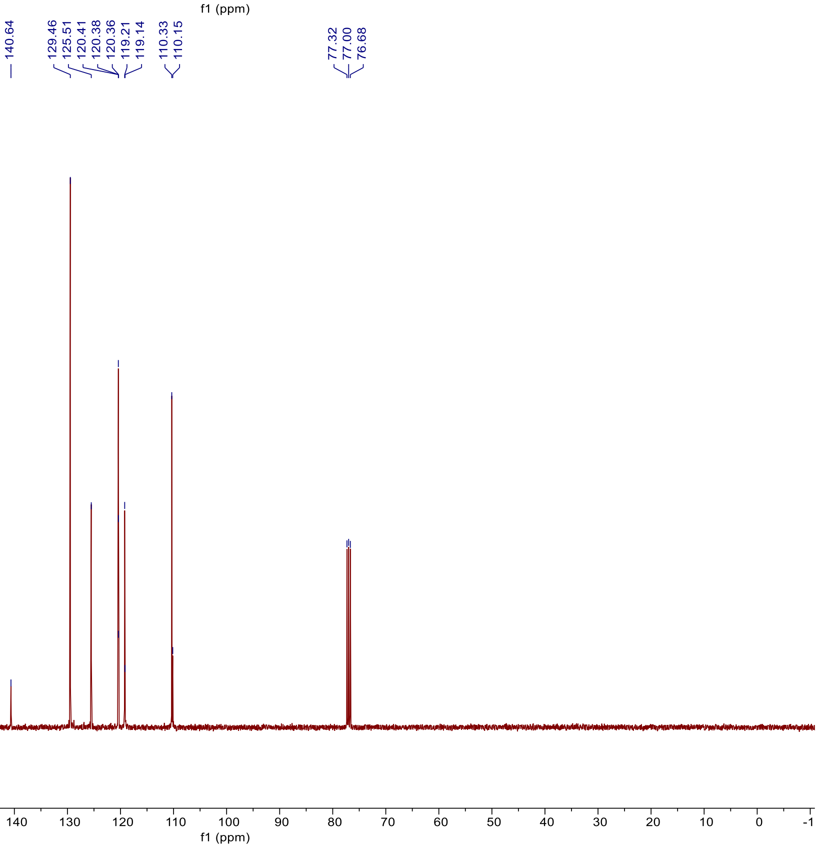

**$^1$H NMR** and **$^{13}$C NMR** of **67**

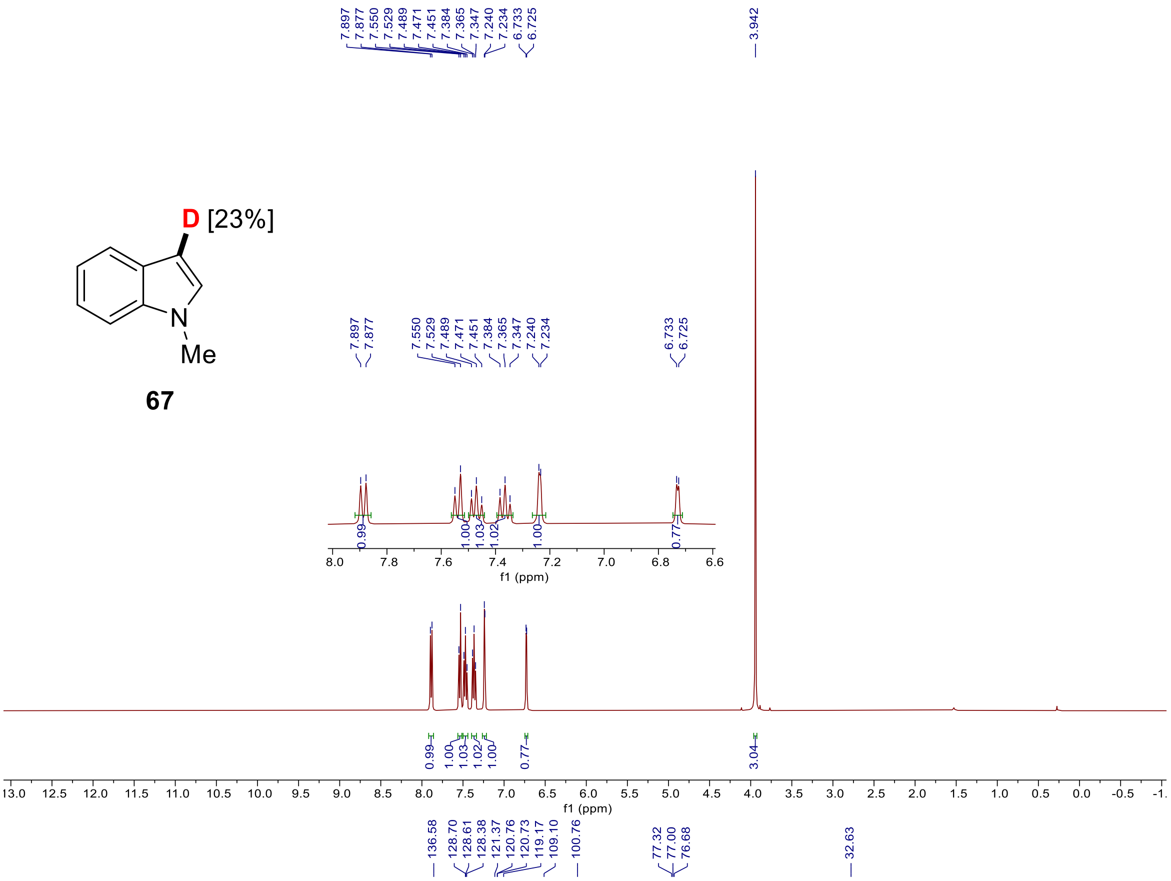

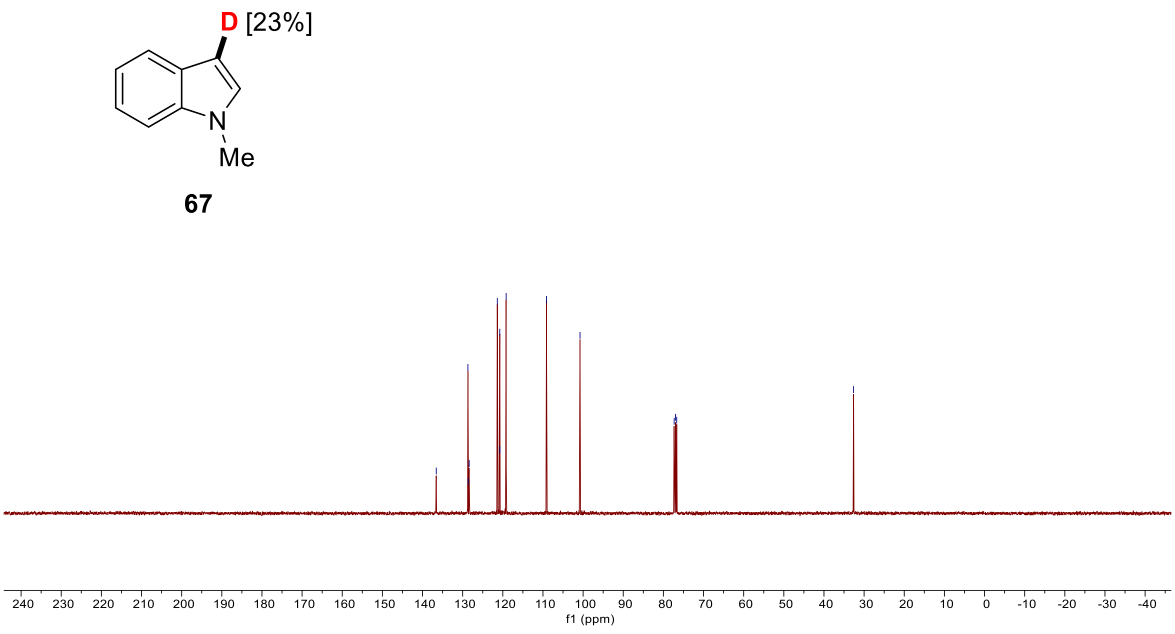

**$^{1}$H NMR** and **$^{13}$C NMR** of **68**

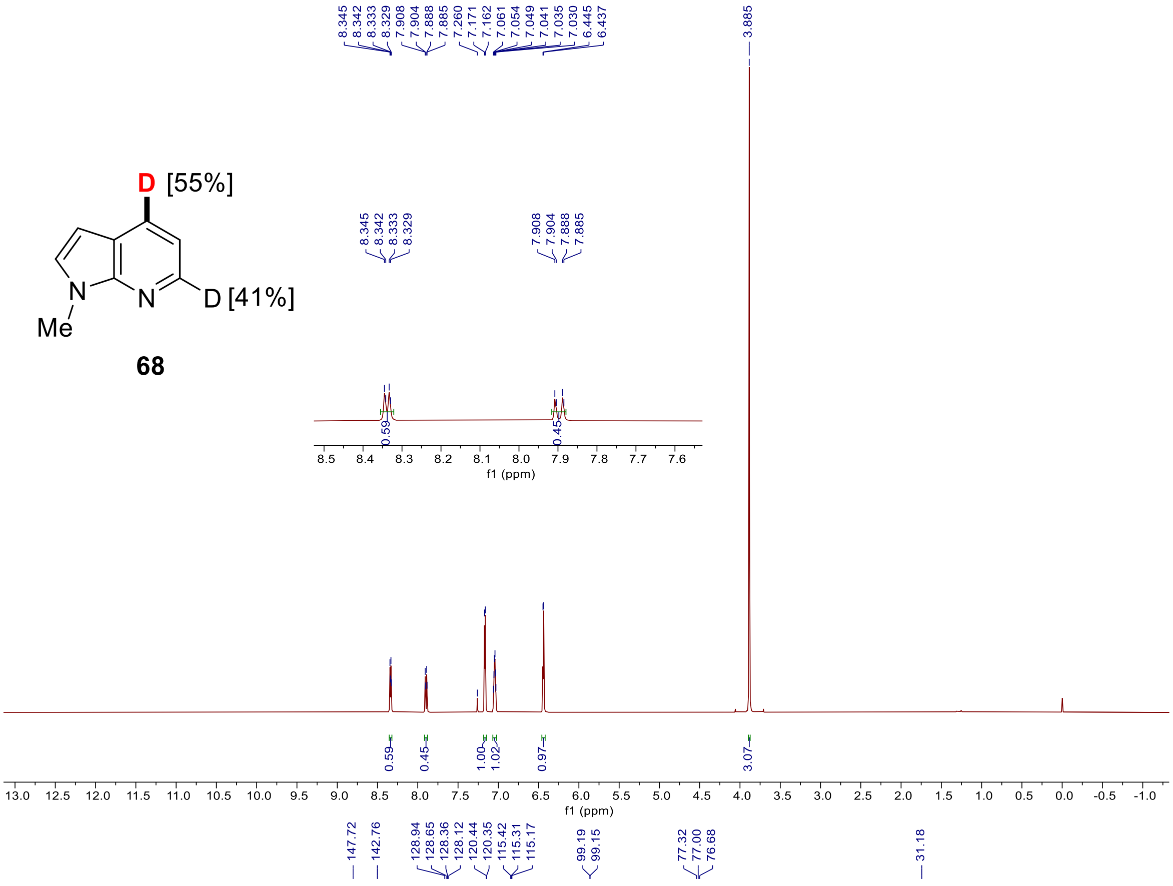

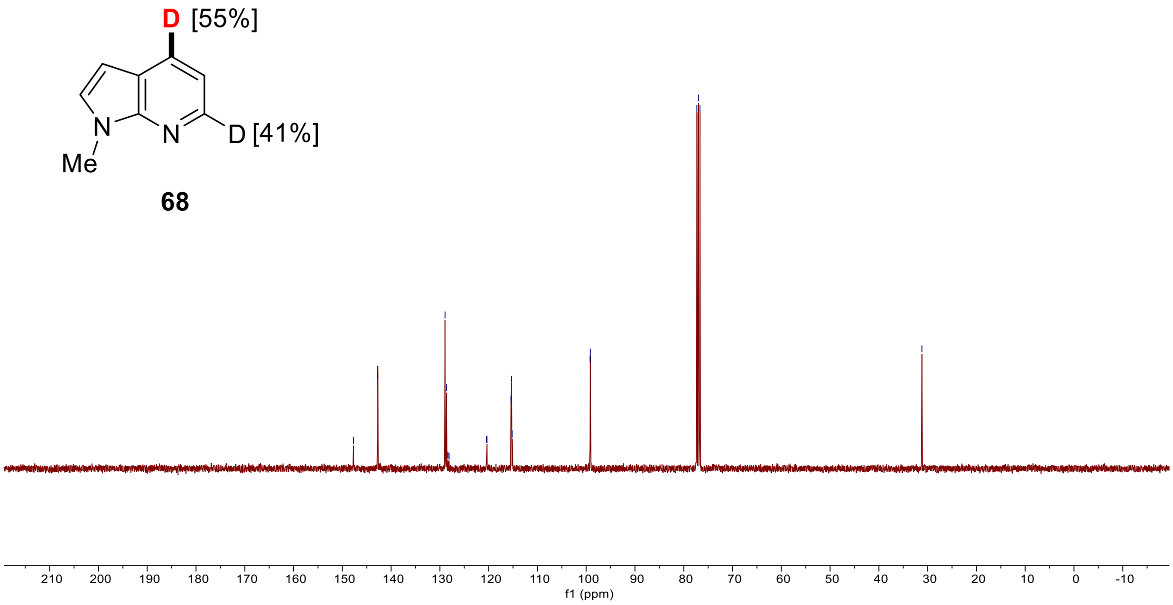

# $^{1}$H NMR and $^{13}$C NMR of 69

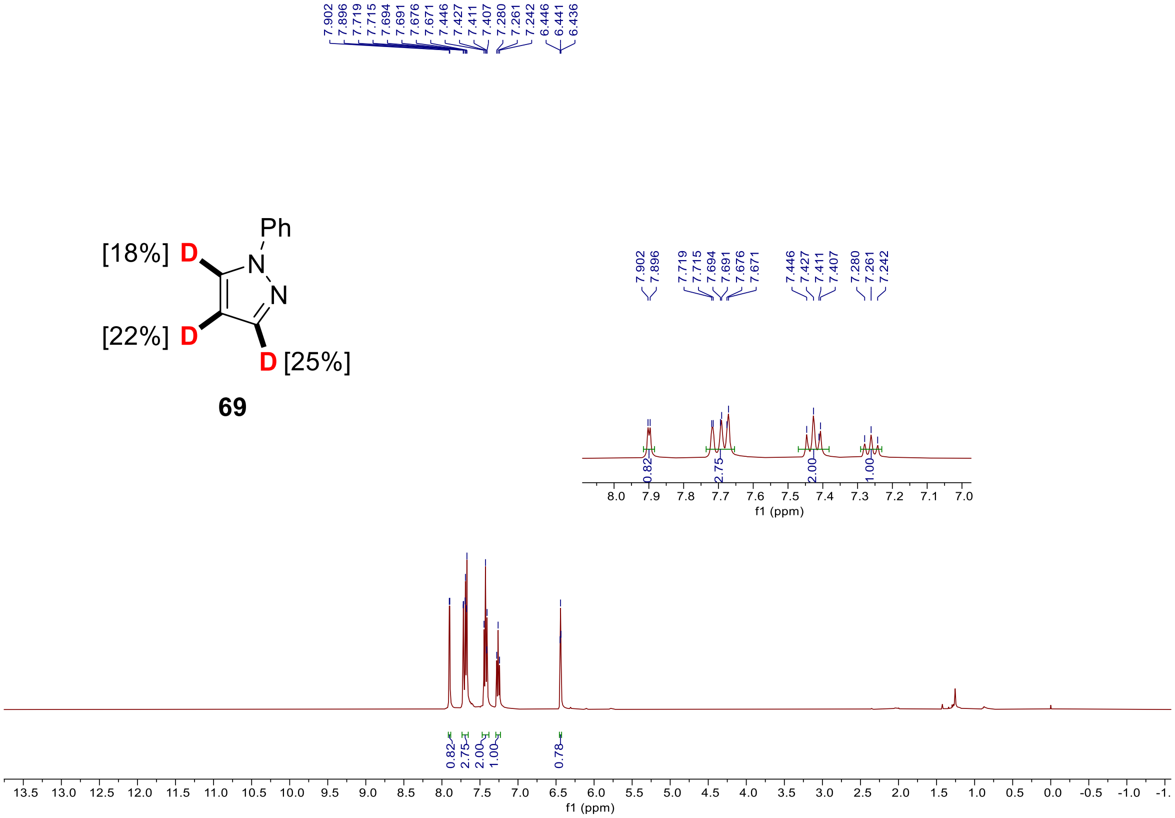


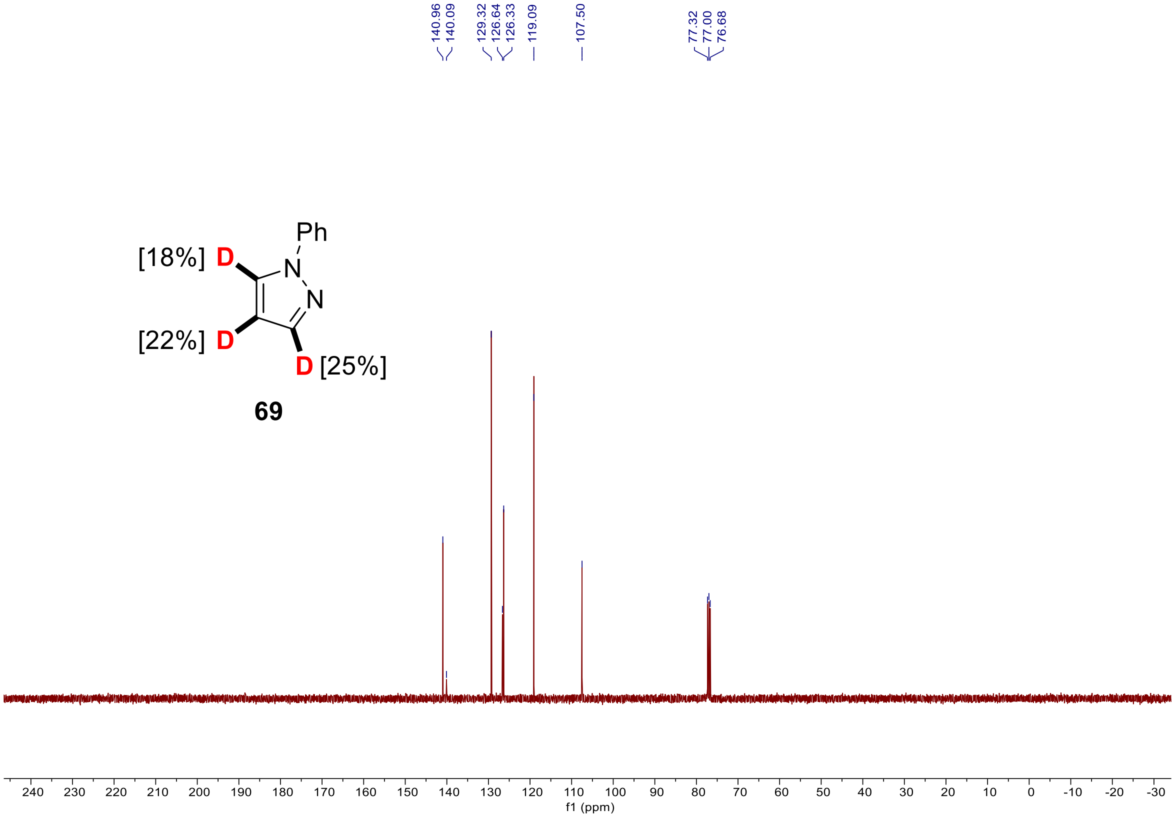

## $^{1}$H NMR and $^{13}$C NMR of **70**

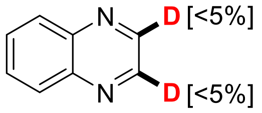


**70**

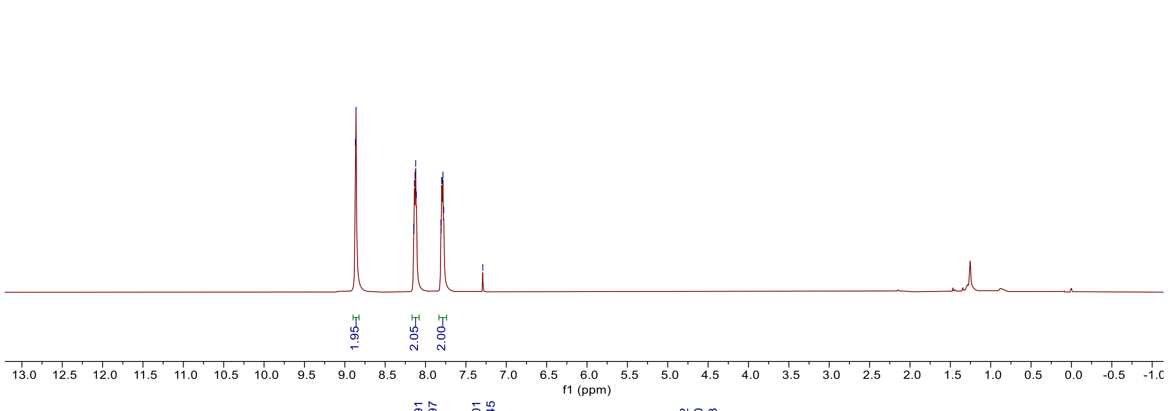


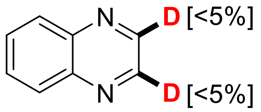


**70**

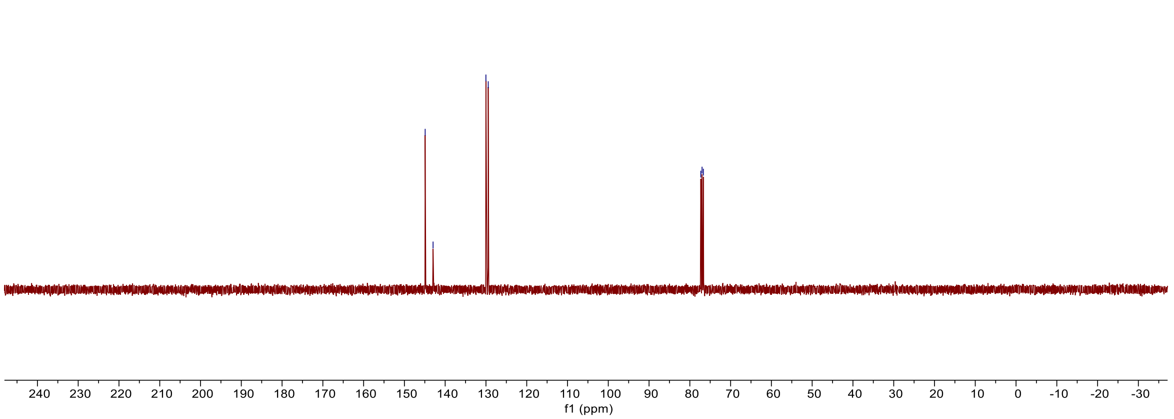

**$^{1}$H NMR** and **$^{13}$C NMR** of **71**

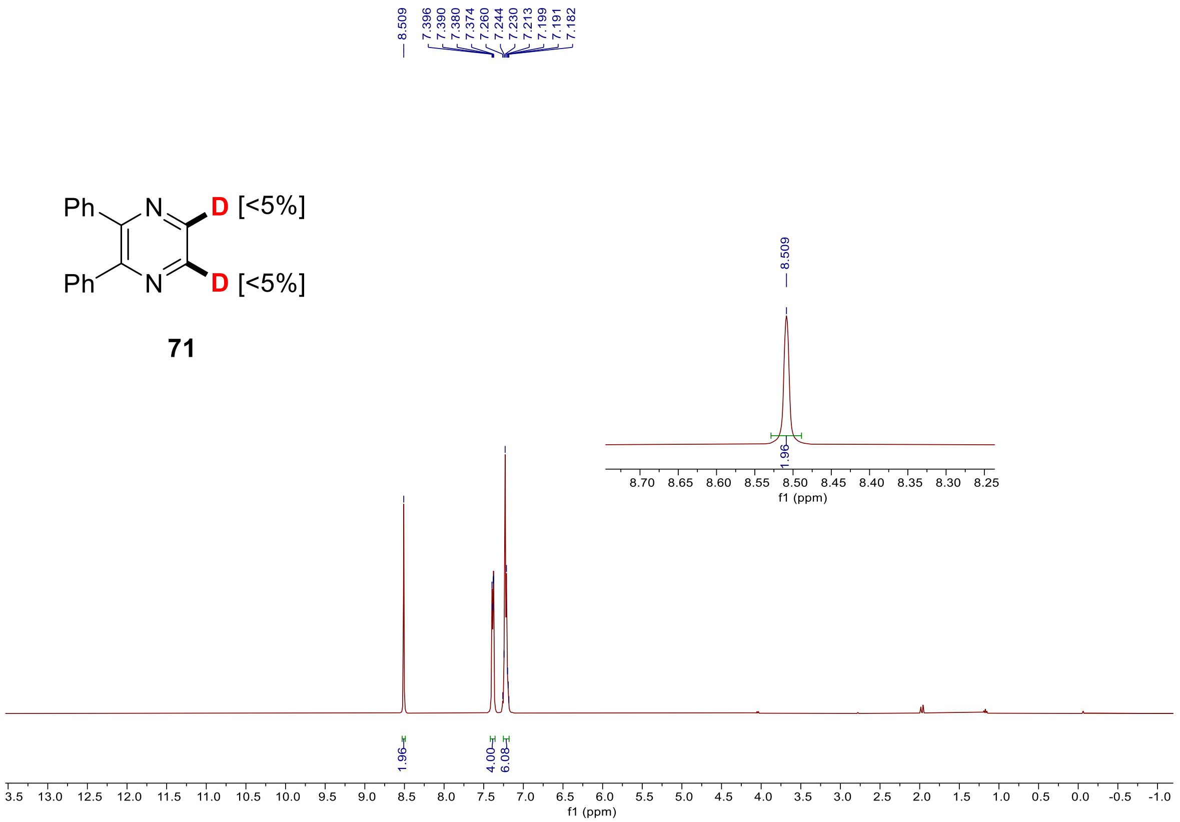


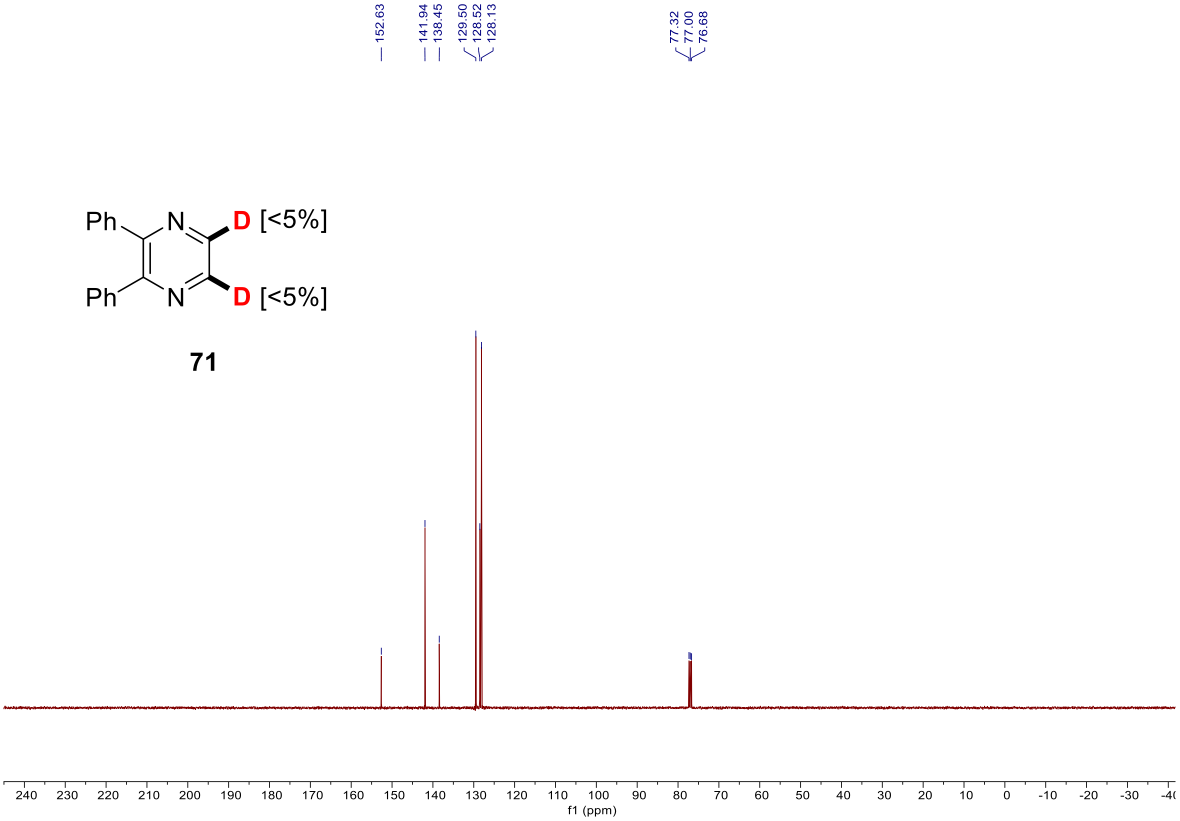

**$^{1}$H NMR** and **$^{13}$C NMR** of **72**

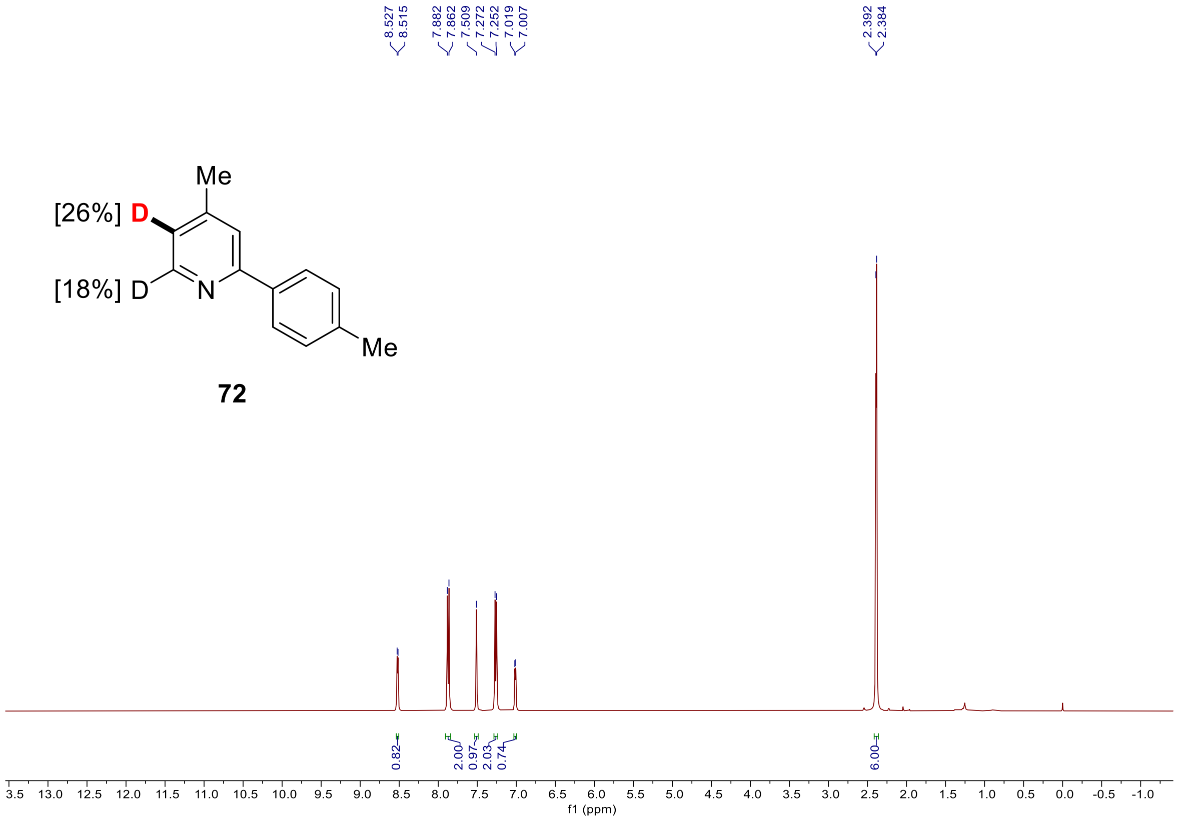


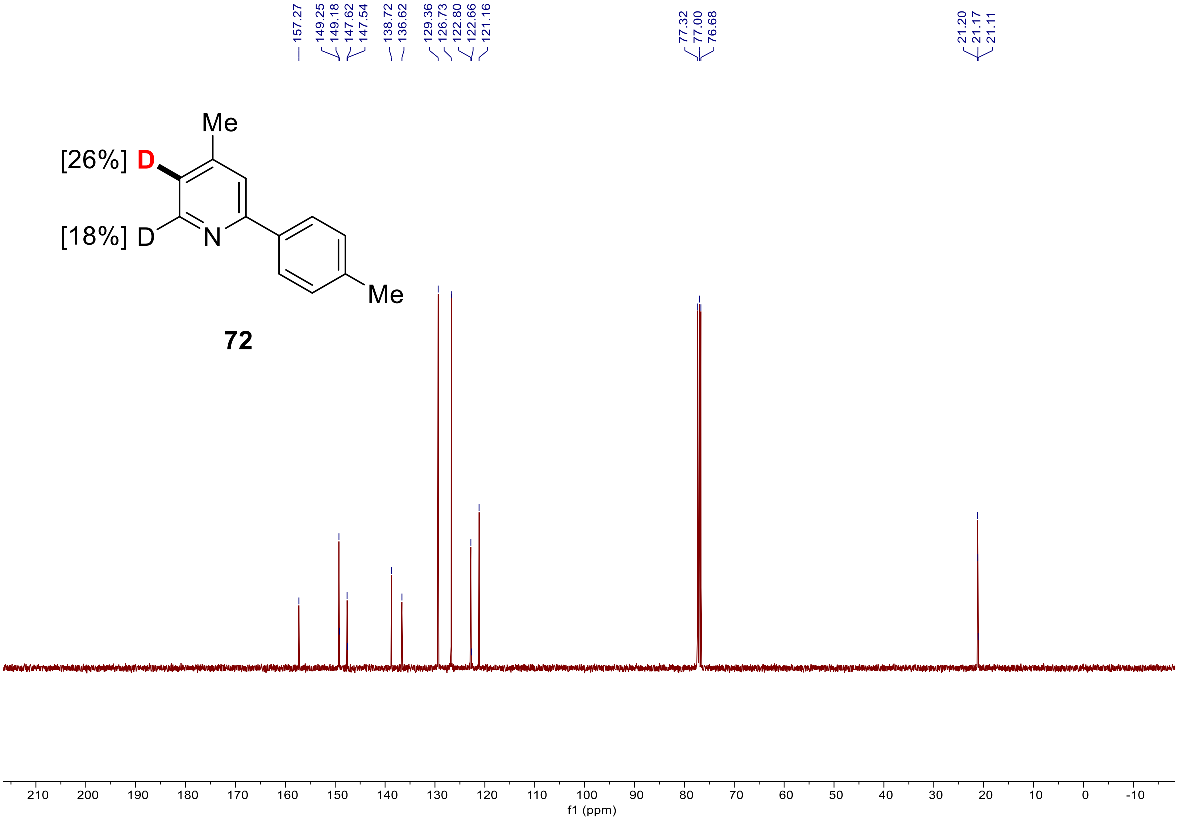

**$^{1}$H NMR** and **$^{13}$C NMR** of **73**

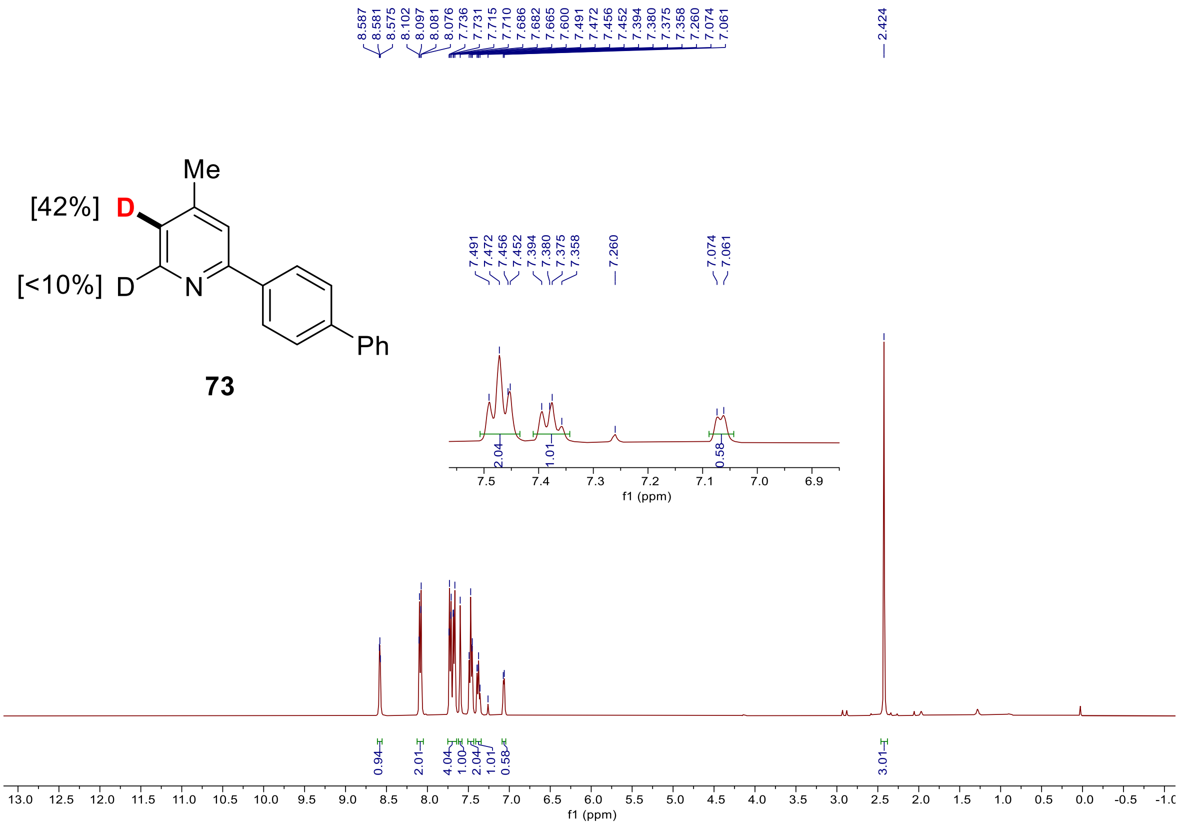


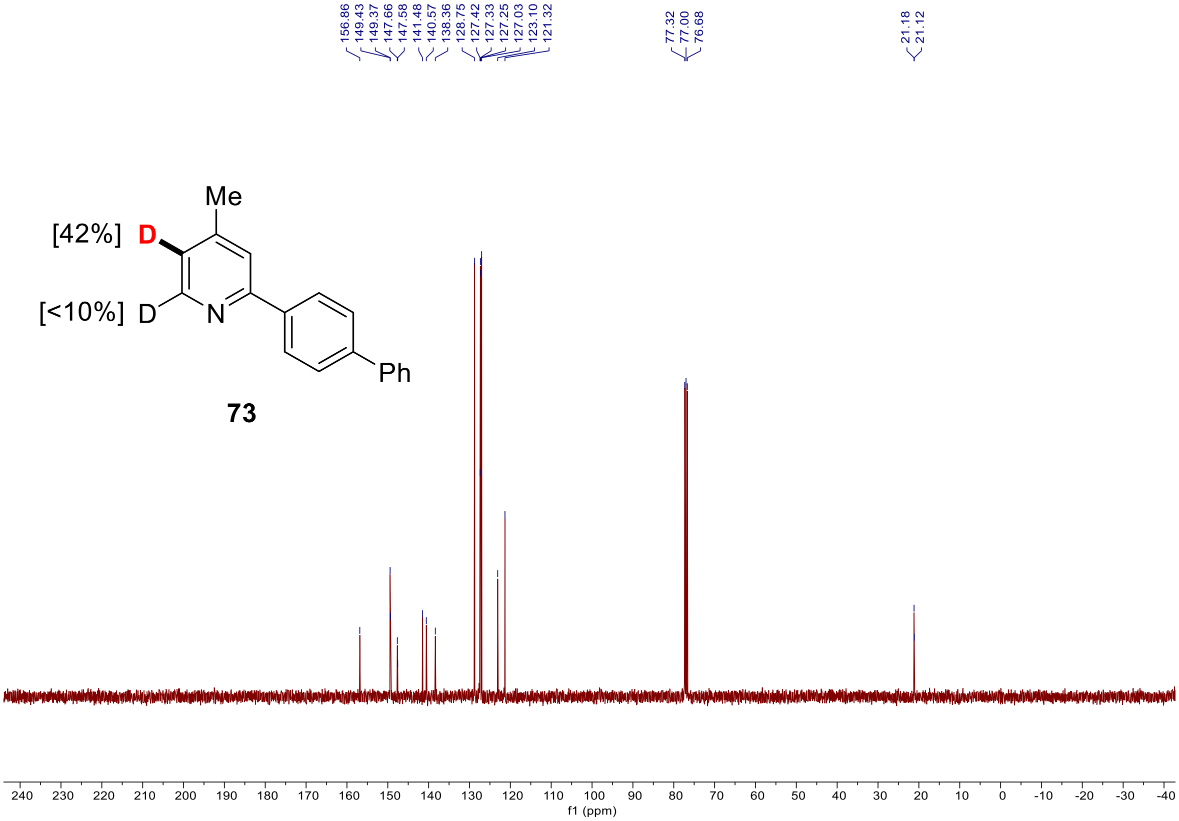

## $^{1}$H NMR and $^{13}$C NMR of 74

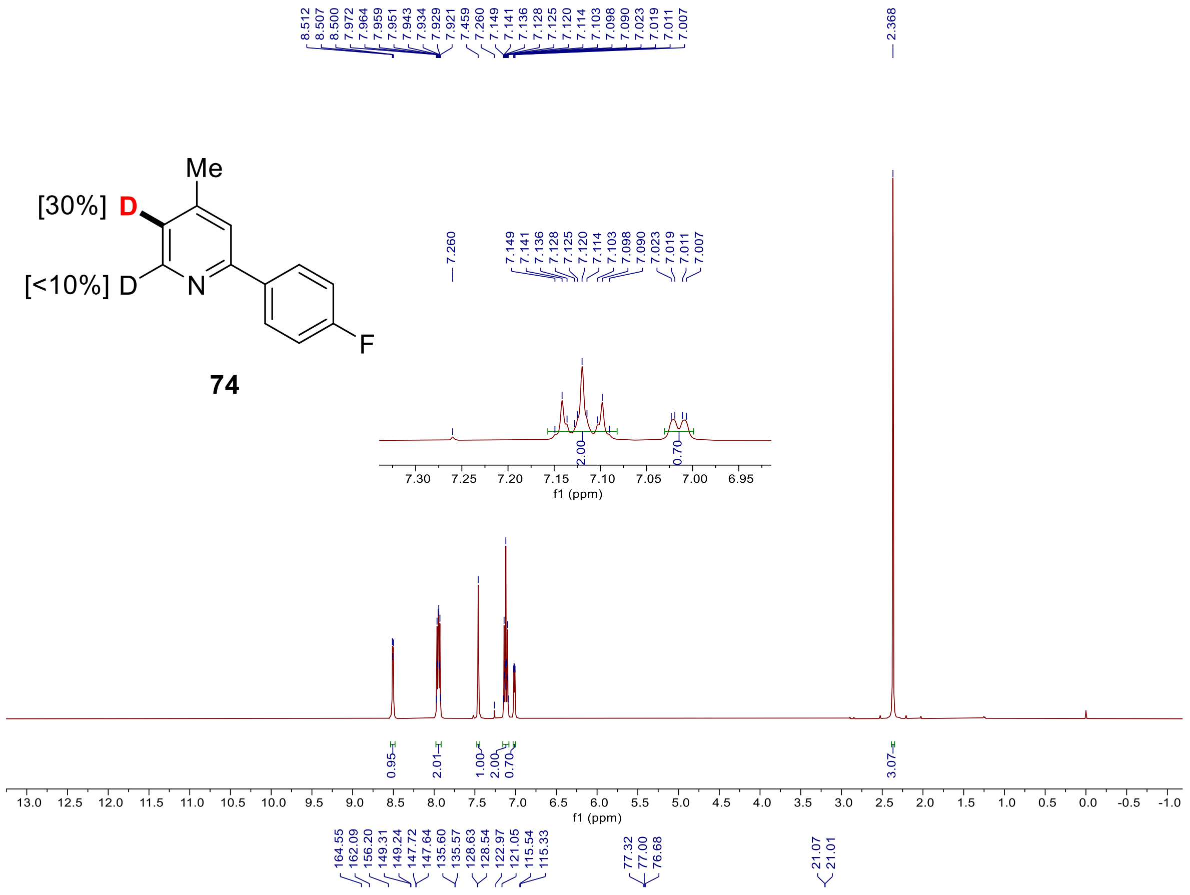

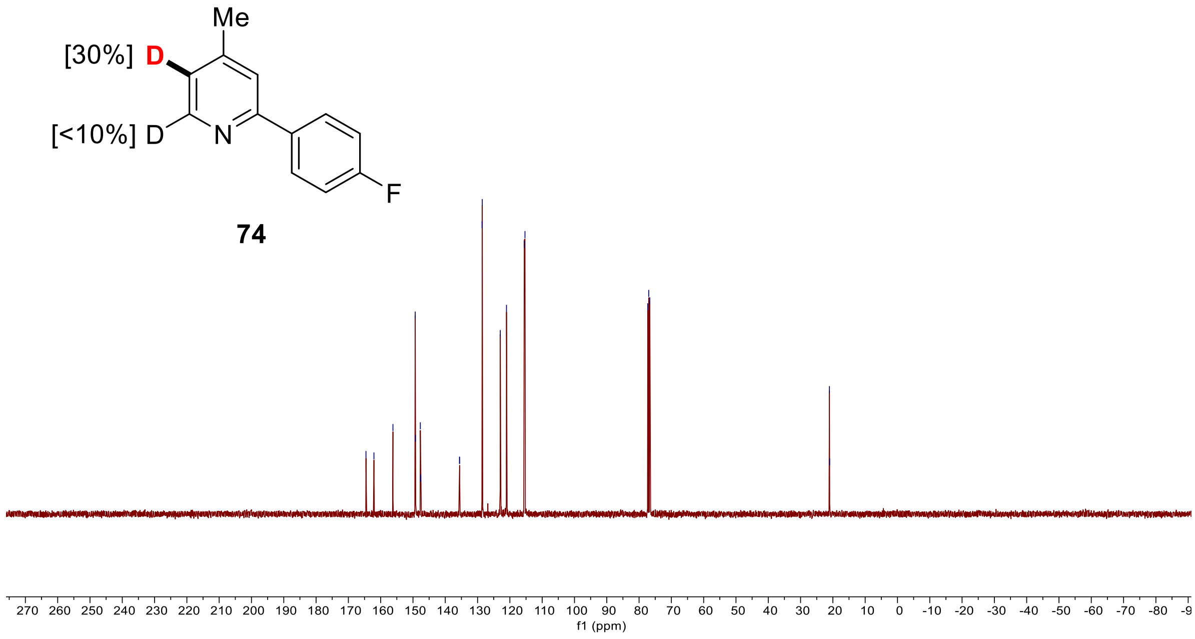

**$^{1}$H NMR** and **$^{13}$C NMR** of **75**

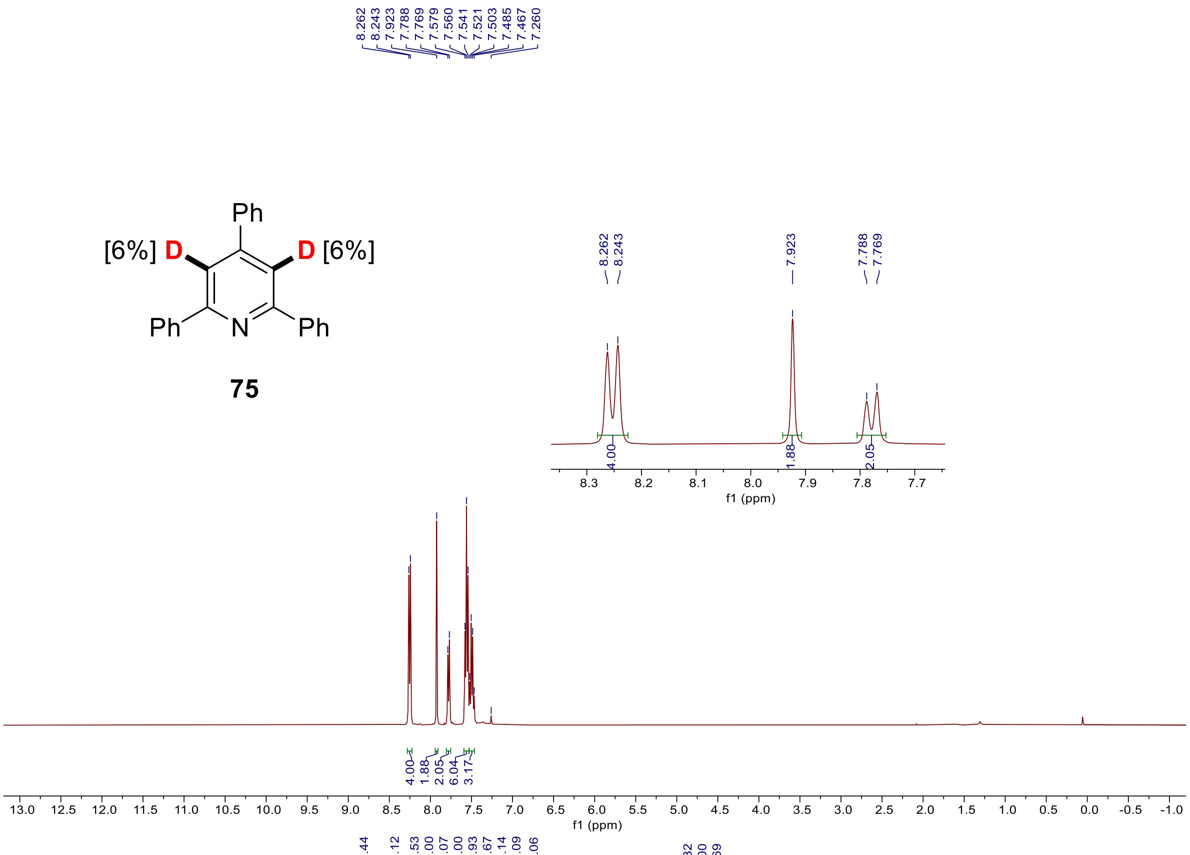


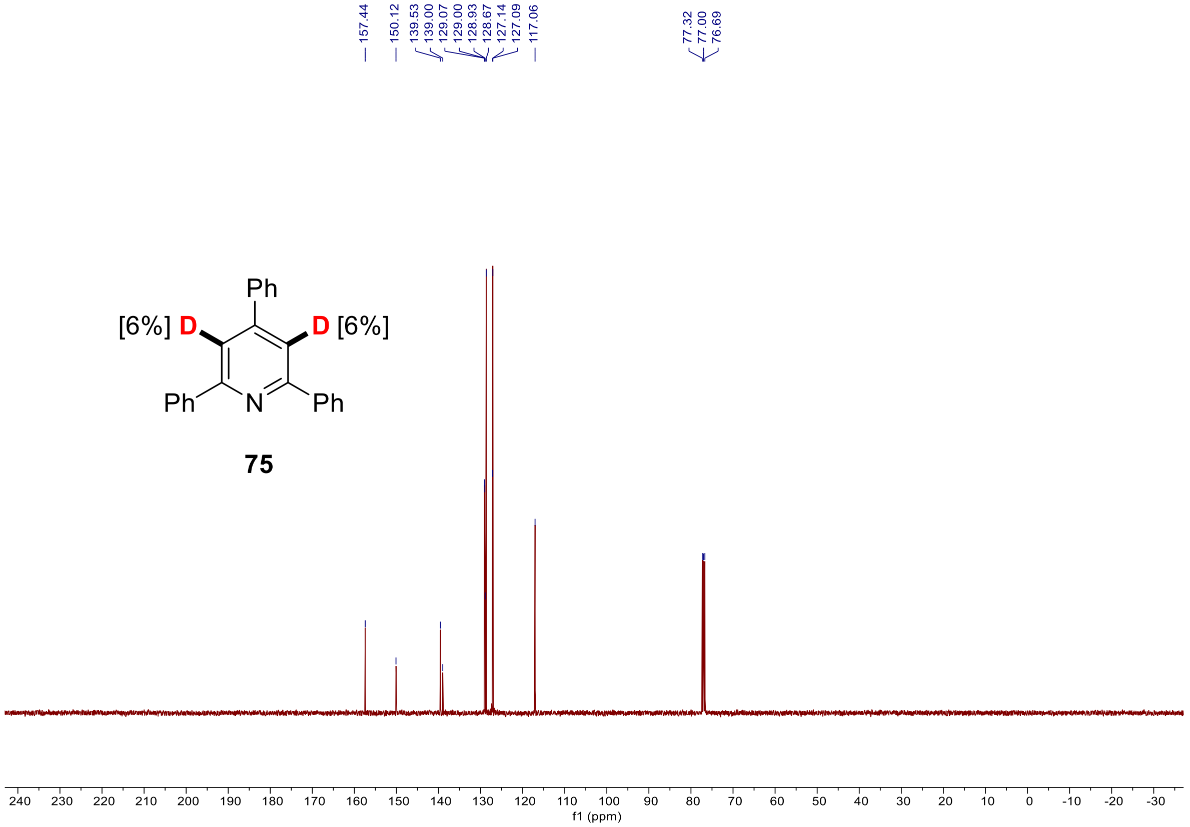

## $^{1}$H NMR and $^{13}$C NMR of S1-a (1-butyl-2-phenylpyridinium iodide)

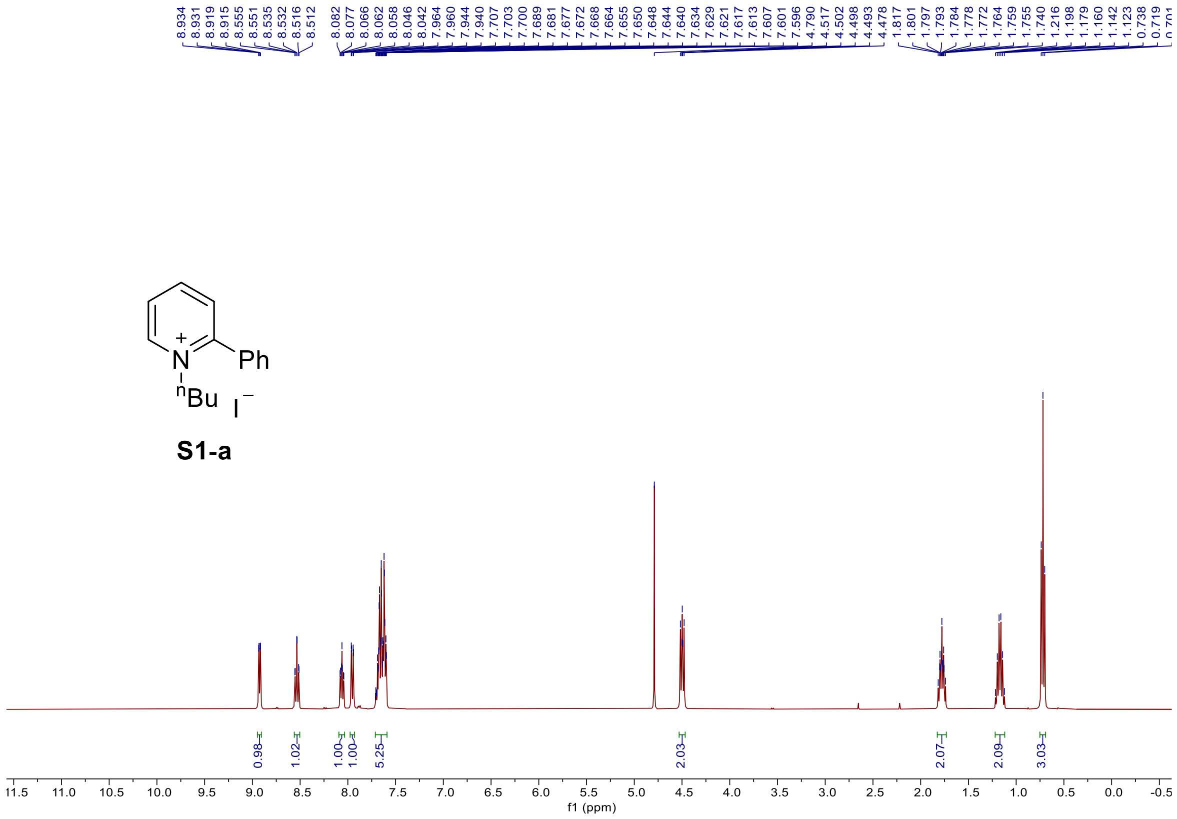


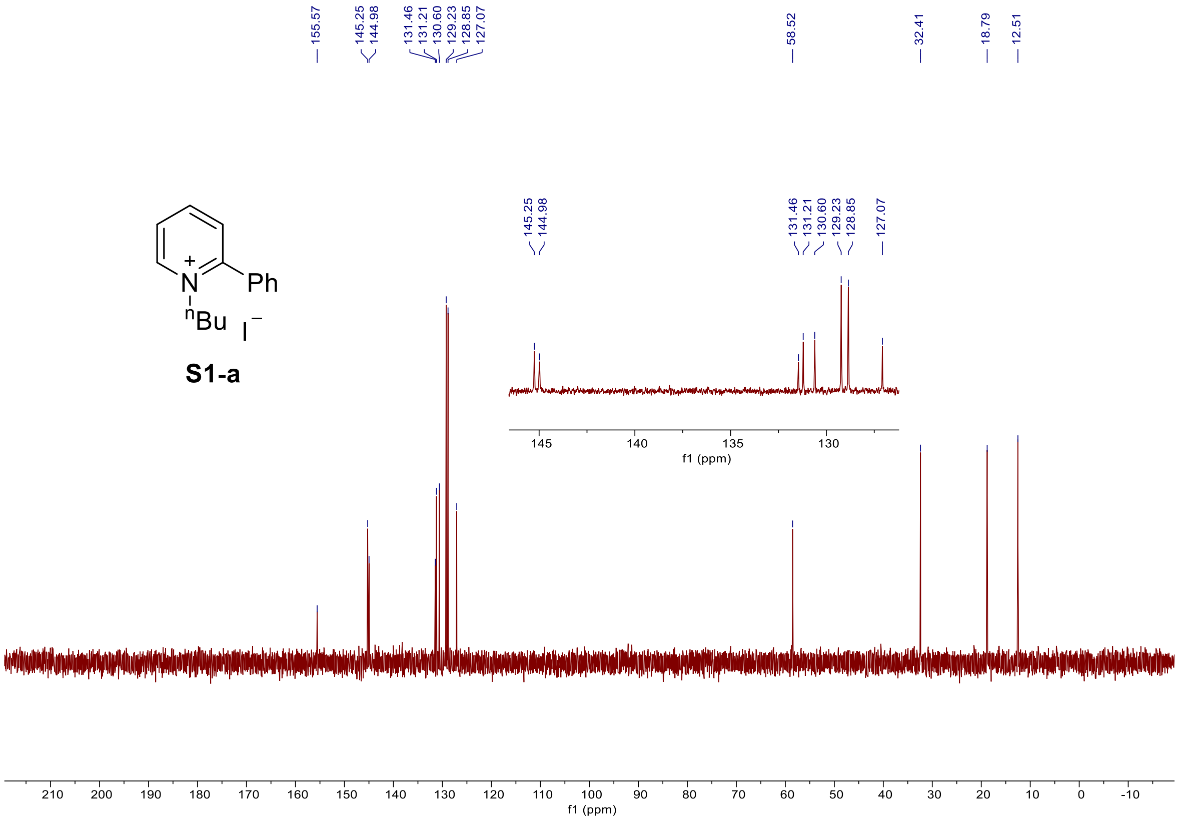

$^{1}H$ NMR and $^{13}C$ NMR of **S1-b (1-propyl-2-phenylpyridinium iodide)**

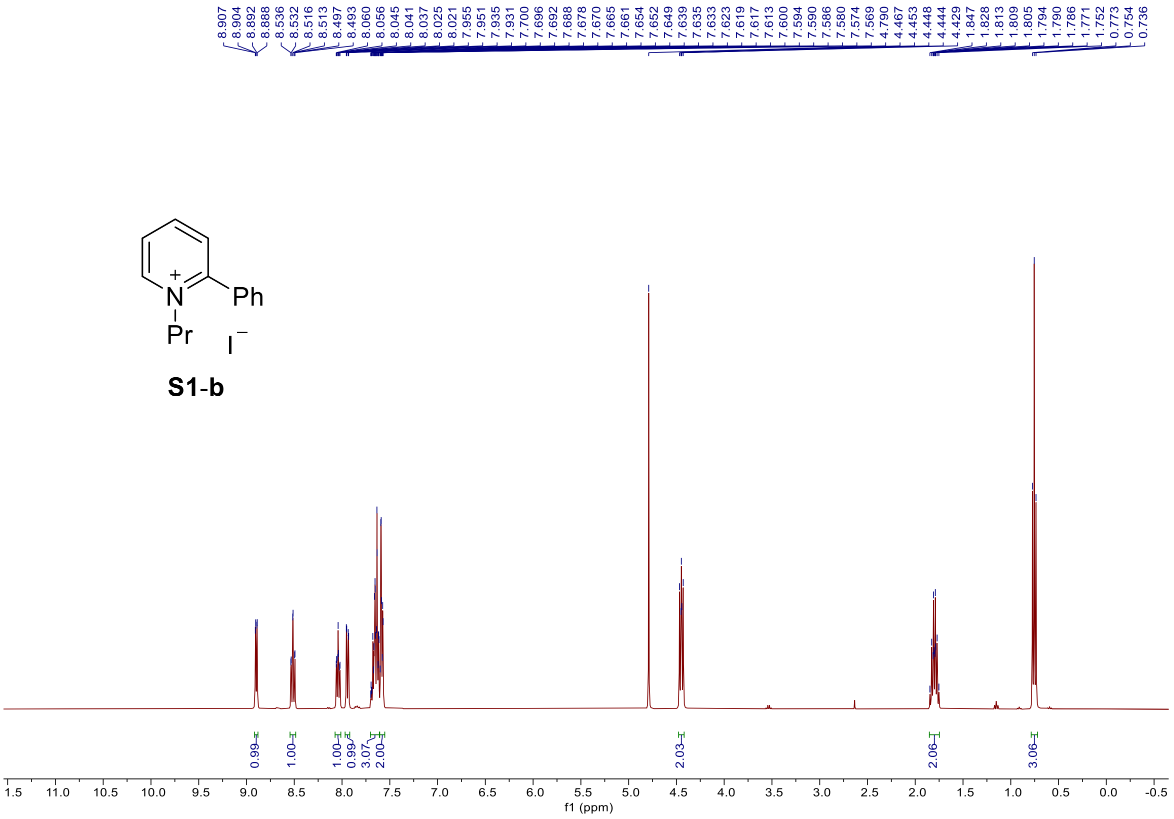

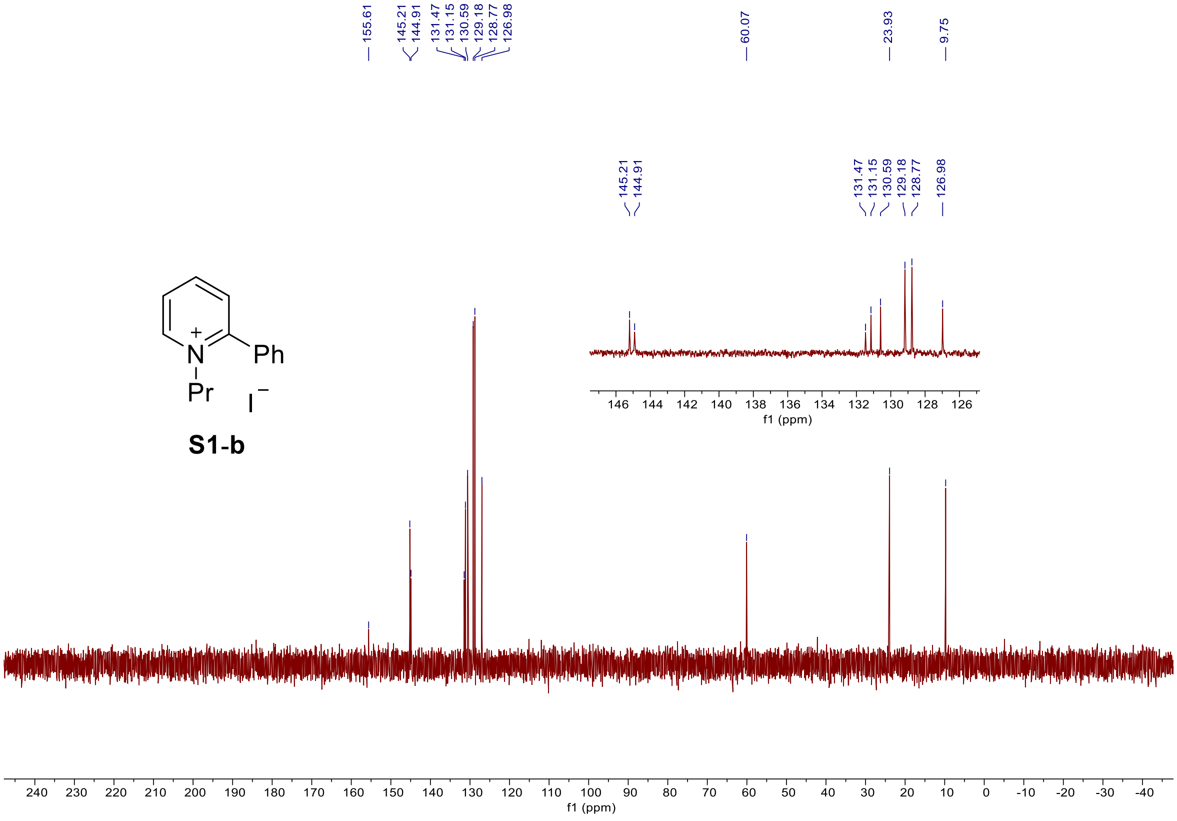

$^{1}$H NMR and $^{13}$C NMR of **S1-c (1-ethyl-2-phenylpyridinium-iodide)**

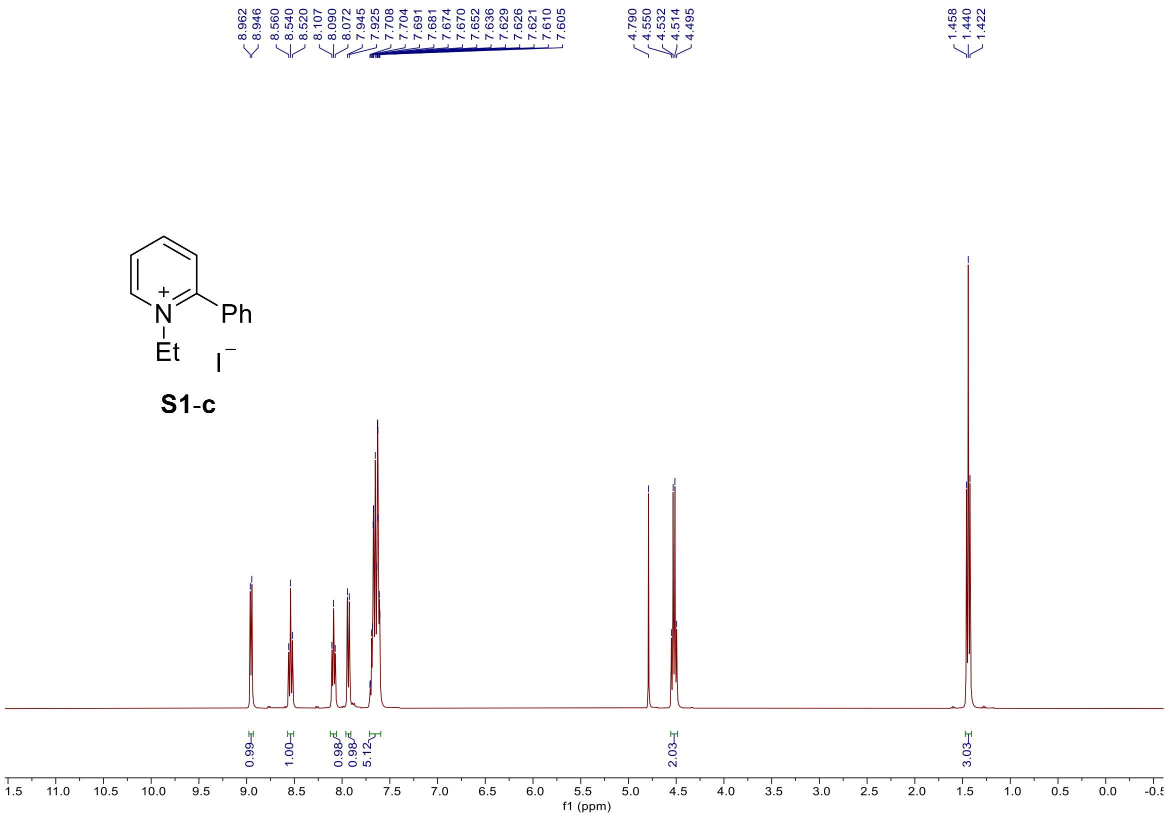


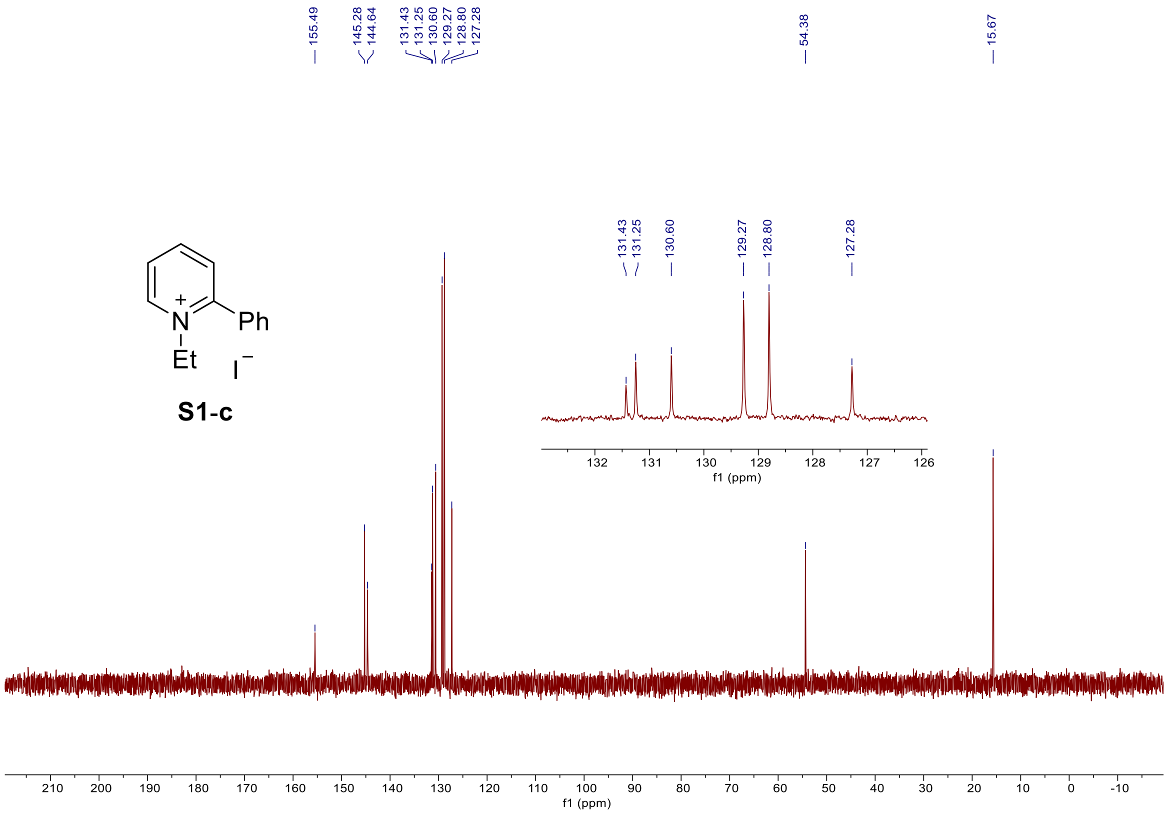

**$^{1}$H NMR** and **$^{13}$C NMR** of **S1-d (1-methyl-2-phenylpyridinium iodide)**

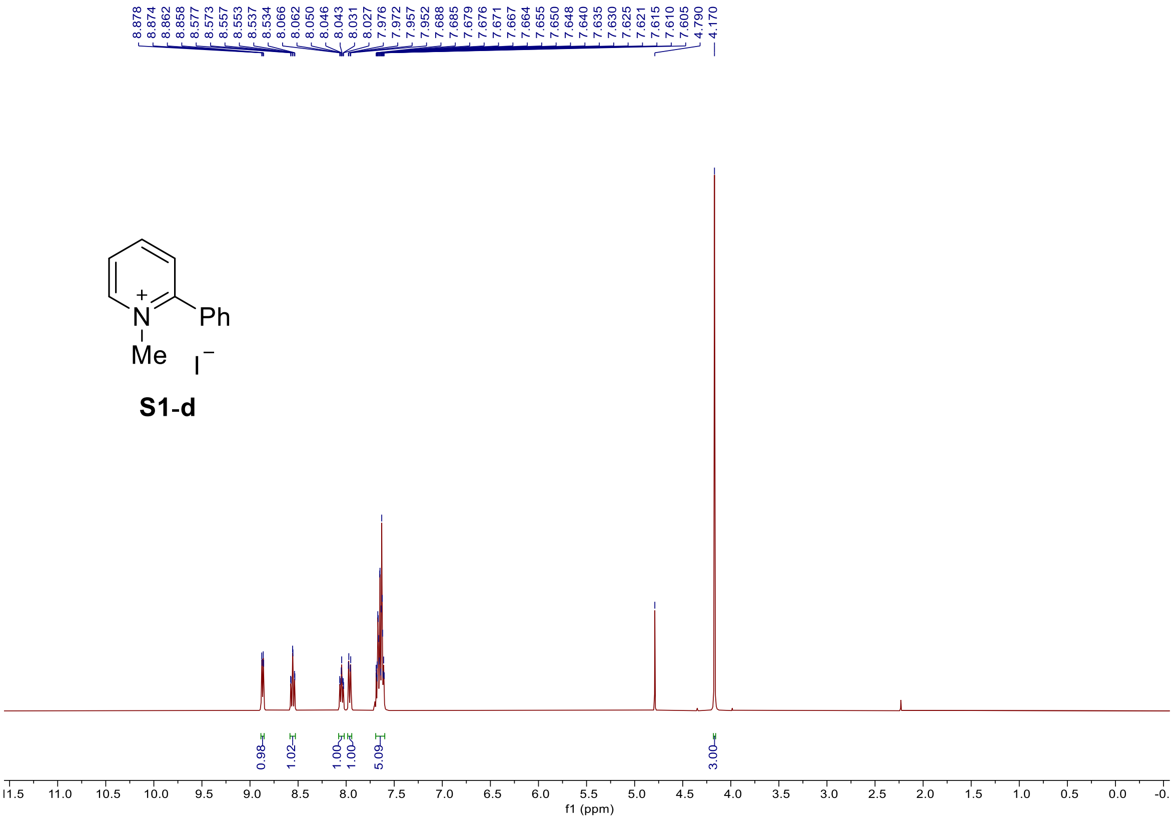

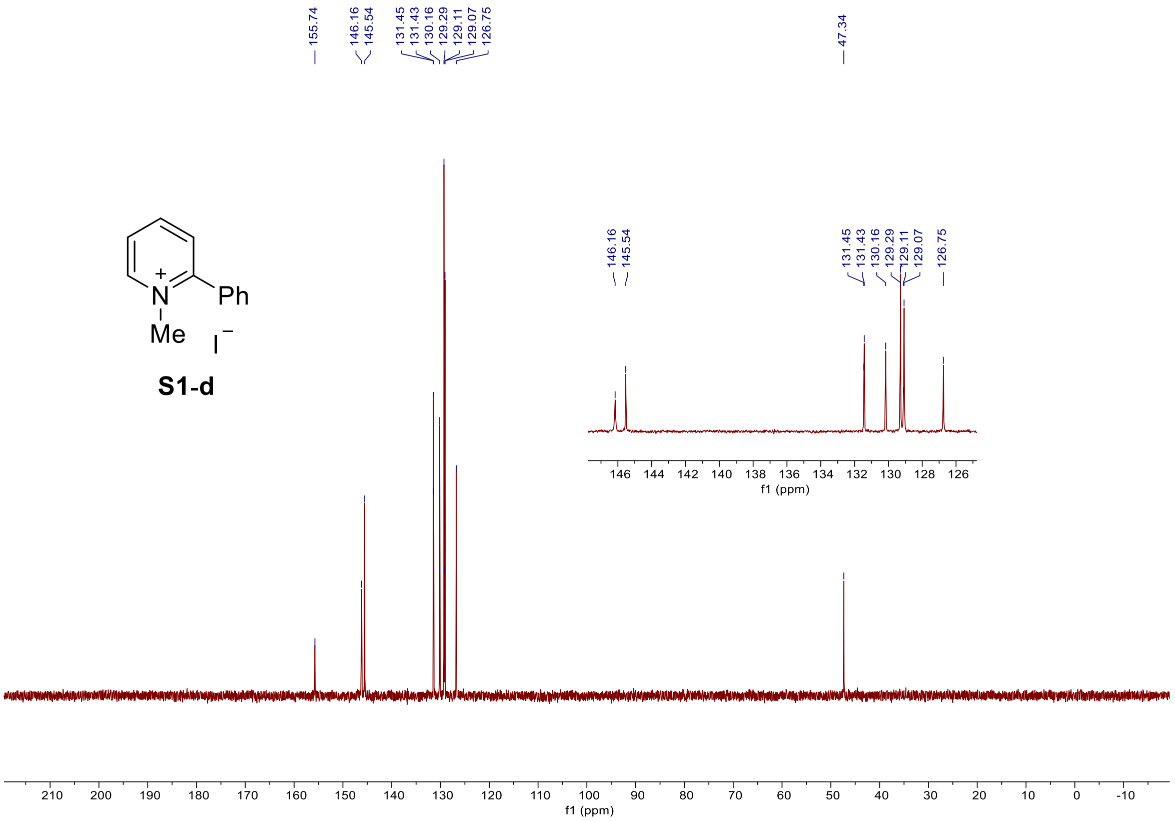

## 4. Supplemental references

1. Stivanin, M., et al. & Jurberga, I. D. Blue light-promoted *N*−H insertion of carbazoles, pyrazoles and 1,2,3-triazoles into aryldiazoacetates. *Adv. Synth. Catal*. **362**, 1106–1111 (2020).
2. Hu, F.-D., et al. & Xia, Y. Enantioselective hydroarylation or hydroalkenylation of benzo[b]thiophene 1,1-dioxides with organoboranes. *Org. Lett*. **23**, 896−901 (2021).
3. Yang, X., et al. & Li, R. Regioselective direct C–H trifluoromethylation of pyridine. *Org. Lett*. **22**, 7108−7112 (2020).
4. Koniarczyk, J. L., et al. & McNally, A. A general strategy for site-selective incorporation of deuterium and tritium into pyridines, diazines, and pharmaceuticals. *J. Am. Chem. Soc*. **140**, 1990−1993 (2018).
5. Kopf, S., et al. Beller, M. Base-mediated remote deuteration of *N*-Heteroarenes broad scope and mechanism. *Eur. J. Org. Chem*. **19**, e202200204 (2022).
6. Tang, J., et al. Chen, H. Copper-mediated and Palladium-catalyzed cross-coupling of indoles and *N*-methylpyridinium salts: a practical way to prepare 3-(pyridin-2-yl)indoles. *Org. Lett*. **25**, 5203–5208 (2023).
7. Gaussian 16, Revision A.03, Frisch, M. J. et.al. Gaussian, Inc. Wallingford CT. (2016).
8. Zhao Y. & Truhlar, D. G. The M06 suite of density functionals for main group thermochemistry, thermochemical kinetics, noncovalent interactions, excited states, and transition elements: two new functionals and systematic testing of four M06-class functionals and 12 other functionals. *Theor. Chem. Acc*. **120**, 215–241 (2008)
9. Grimme, S., Antony, J., et al. & Krieg, H. A consistent and accurate *ab initio* parametrization of density functional dispersion correction (DFT-D) for the 94 elements H-Pu. *J. Chem. Phys.* **132**, 154104 (2010).
10. Weigend, F. & Ahlrichs, R. Balanced basis sets of split valence, triple zeta valence and quadruple zeta valence quality for H to Rn: Design and assessment of accuracy. *Phys. Chem. Chem. Phys*. **7**, 3297–3305 (2005).
11. Marenich, A. V., Cramer, C. J. & Truhlar, D. G. Universal solvation model based on solute electron density and on a continuum model of the solvent defined by the bulk dielectric constant and atomic surface tensions. *J. Phys. Chem*. **113**, 6378–6396 (2009).

12. Tomasia, J., Mennuccia, B. & Cancèsb, E. The IEF version of the PCM solvation method: an overview of a new method addressed to study molecular solutes at the QM ab initio level. *J. Mol. Struct*. (Theochem), **464**, 211–226 (1999).
